# Alternative splicing broadens antiviral diversity at the human *OAS2* locus

**DOI:** 10.1038/s44318-026-00825-w

**Published:** 2026-06-03

**Authors:** Emma L Davies, Alegna Calderon Nuñez, Allison L Ward, Hanna Sowar, Eilidh Rivers, Arda Balci, Daniel Mair, Elliot Moorhouse, Jake Towers, Arthur Wickenhagen, Matthew L Turnbull, Massimo Palmarini, Sam J Wilson, Adam J Fletcher

**Affiliations:** 1 MRC-University of Glasgow Centre for Virus Research, University of Glasgow, Glasgow, UK; 2 Cambridge Institute of Therapeutic Immunology and Infectious Disease (CITIID), University of Cambridge, Cambridge, UK

**Keywords:** Immunology, Microbiology, Virology & Host Pathogen Interaction

## Abstract

Interferons (IFN) are cytokines that regulate the expression of hundreds of genes during viral infections to generate a broadly antiviral environment in the stimulated cell. Antiviral breadth is provided by the concurrent expression of many individual IFN-stimulated genes (ISG), each encoding a protein with often exquisite antiviral specificity. Here, we identify mechanistic plasticity at a single genetic locus as a novel mechanism to diversify the antiviral profile of human cells. Through alternative splicing, the OAS2 gene encodes two antiviral molecules with distinct target specificities. The shorter OAS2 p69 isoform restricts seasonal human coronavirus OC43 (HCoV-OC43), whereas the longer p71 isoform restricts picornavirus Cardiovirus A (EMCV). The restriction profile is determined by the variable length OAS2 C-terminal tails. Notably, these antiviral activities differ in their dependence on RNase L, suggesting that alternative splicing separates canonical restriction and virus sensing functions across two distinct OAS2 polypeptides. Together, these findings show how alternative splicing expands antiviral diversity at the human OAS2 locus.

## Synopsis

OAS2 is an interferon-induced antiviral factor that classically restricts viral replication through activation of RNase L. This manuscript shows that two distinct OAS2 isoforms restrict unrelated RNA viruses through divergent C-terminal tails that determine antiviral specificity and RNase L dependence.

- Two OAS2 isoforms restrict the replication of different viruses.
- OAS2 p69 restricts HCoV-OC43 independently of RNase L, whereas OAS2 p71 restricts EMCV through canonical RNase L-dependent signaling.
- Invariant N-terminal myristoylation promotes Golgi localisation and virus detection, while short C-terminal tails determine antiviral specificity and mechansim.

## Abstract (web summary)

Alternative splicing generates OAS2 isoforms that restrict EMCV or HCoV-OC43 through divergent RNase L-dependent and -independent mechanisms.

## Graphical abstract

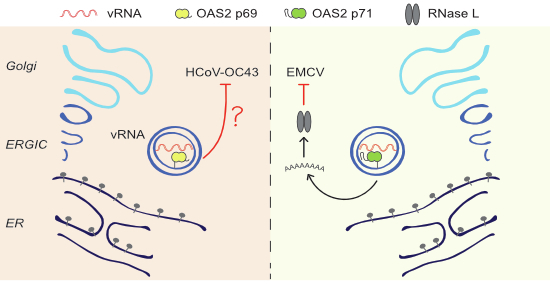

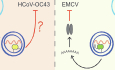

## Introduction

Across the tree of life, antiviral defence systems are often based on the recognition of viral RNA (vRNA). In eukaryotes, from cartilaginous fish to birds and mammals, vRNA detection leads to the secretion of cytokines called interferons (IFN) (Dalskov et al, 2023; Sa Ribero et al, 2020). IFNs regulate the transcription of hundreds of interferon-stimulated or interferon-repressed genes (ISG/IRG), many of which encode proteins with antiviral function (Schoggins, 2019; Shaw et al, 2017). The importance of a broad antiviral defence is evidenced by patients with severe viral disease, including herpes simplex encephalitis (HSE) and COVID-19, who harbour defects in components of the IFN pathway, including self-reactive antibodies against IFN or genetic errors in key IFN signalling components like vRNA sensors TLR3 and MDA5 (Bastard et al, 2020; Hambleton et al, 2013; Lamborn et al, 2017; Zhang et al, 2007).

The causative agent of COVID-19, SARS-CoV-2, is the third coronavirus to emerge into the human population since the turn of the century, along with SARS-CoV-1 and MERS-CoV (Drosten et al, 2003; Zaki et al, 2012; Zhu et al, 2020). Owing to the recent spillover, these emerging human coronaviruses can be highly pathogenic and cause more severe, lower respiratory tract infections than their seasonal counterparts (HCoV-229E, HCoV-NL63, HCoV-HKU1 and HCoV-OC43), which mainly cause common cold-like symptoms (Gaunt et al, 2010). HCoV-OC43 is thought to have diverged from bovine coronavirus (BCoV) in the late 19th century, when it jumped into humans via an intermediate livestock species (Shaw and Gatherer, 2023; Vijgen et al, 2005). Based on contemporary events, it is likely that HCoV-OC43 caused an epidemic upon its emergence but later evolved into an endemic respiratory virus (Gaunt et al, 2010). Understanding the cellular factors that regulate HCoV-OC43 replication in humans could contribute to our understanding of viral adaptation in new hosts.

To search for these factors, we employed large-scale cDNA screens and uncovered antiviral activity of 2’-5’ oligoadenylate (2-5A) synthetase 2 (OAS2) against the seasonal coronavirus HCoV-OC43. Of the two major OAS2 isoforms sharing the same enzymatic core, only the shorter p69 isoform is antiviral toward HCoV-OC43. Conversely, we find that the longer p71 isoform restricts picornavirus Cardiovirus A (encephalomyocarditis virus (EMCV)), in a manner dependent on the length of its long C-terminal tail. While p71 restriction of EMCV occurs via the classic OAS/RNase L pathway, p69 restriction of HCoV-OC43 is entirely RNase L-independent and requires two residues at the very C-terminus of the protein. Our data demonstrate that alterations in the OAS2 C-terminal tail affect target specificity, providing a mechanism to achieve antiviral diversity from a single genetic locus.

## Results

### Developing a high-throughput assay for dsRNA formed during HCoV-OC43 replication

**Figure EV1:**
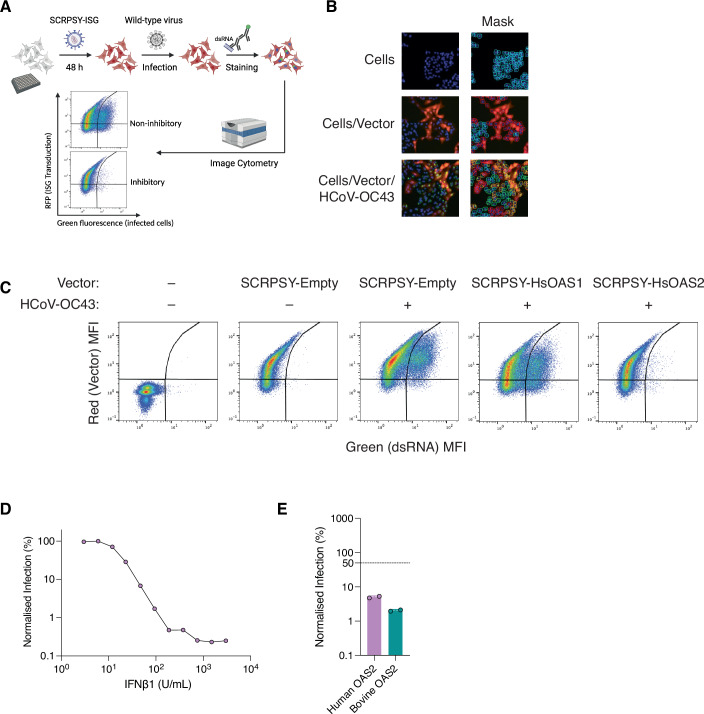
Quantifying RNA virus infection by immunostaining. (**A**) Schematic diagram of the screening method used for the arrayed ISG expression screens described in (Fig. 1D–G). (**B**) Representative images of control wells in the ISG screen (Fig. 1D) were gated for RFP and dsRNA expression levels using an image cytometer. (**C**) Cell populations from (Fig. 1D) were gated using cell-only and SCRPSY-Empty mock-infected controls. Representative FlowJo analysis plots are shown, including OAS1 and OAS2 as examples of a non-candidate and candidate ISG, respectively. MFI, mean fluorescence intensity. (**D**) A549 cells were pre-treated with IFNβ1 for 24 h, infected with HCoV-OC43 for 72 h, and stained for dsRNA prior to quantifying dsRNA+ cells by image cytometry. (**E**) A549 expressing Human or Bovine OAS2, from the ISG libraries in (Fig. 1D), were infected with HCoV-OC43 for 72 h and stained for dsRNA prior to quantifying dsRNA+ cells by image cytometry. Data normalised to SCRPSY-EMPTY controls.

**Figure 1:**
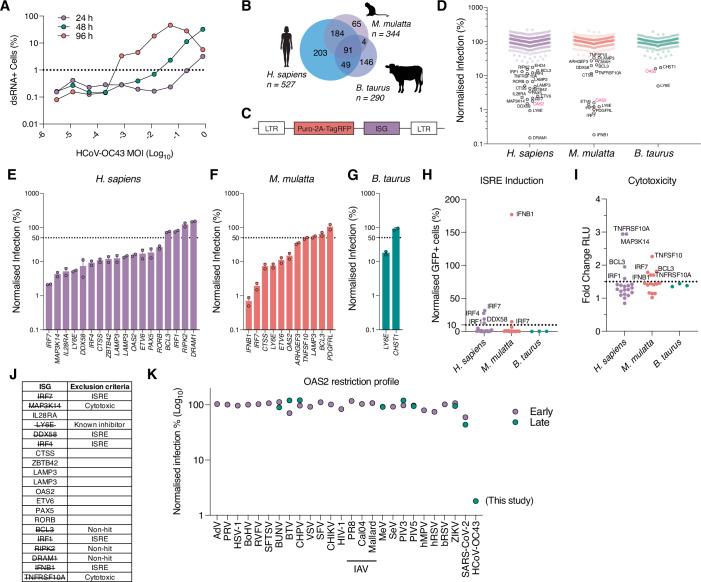
Identification of genes with antiviral activity against HCoV-OC43. (**A**) A549 cells were infected with serially diluted HCoV-OC43. The proportion of dsRNA+ cells at multiple timepoints was quantified in fixed cells by immunostaining for dsRNA followed by image cytometry. (**B**) Schematic of the human (*H. sapiens*), macaque (*M. mulatta*) and bovine (*B. taurus*) ISG libraries used in this study. (**C**) Schematic of the SCRPSY lentiviral vector, encoding an individual ISG and TagRFP. (**D**) A549 cells were transduced with hundreds of ISGs (**B**), infected with HCoV-OC43 (MOI 0.07) for 72 h, immunostained for dsRNA, and the level of infection quantified using image cytometry. Infection was normalised to the mean of the species library. (**E**–**G**) Miniscreens were performed to validate the ability of (**E**) human, (**F**) macaque or (**G**) bovine ISGs identified in (**D**) to restrict HCoV-OC43 (MOI 0.07) at 72 hpi. The LAMP3 gene is duplicated in the human ISG library. Shown is the mean of duplicate experiments. (**H**) The ability of candidate ISGs to stimulate the ISRE in A549-ISRE:GFP cells, assessing GFP expression by flow cytometry 120 h post-transduction. (**I**) The ability of candidate ISGs to cause cytotoxicity, determined using the CytoTox-Glo™ assay using supernatant harvested from the A549-ISRE:GFP cells in (**H**). (**J**) Shortlist of human ISGs that are candidate restriction factors of HCoV-OC43. (**K**) Normalised infection levels in cells expressing the p69 isoform for a variety of viruses, from 34 arrayed ISG expression screening datasets. Source data are available online for this figure.

Unlike HCoV-229E and SARS-CoV-2, which originated in bats (Latinne et al, 2024), rodents are thought to be the ancestral host of HCoV-OC43 (Corman et al, 2018), and there has been little research on genes with antiviral activity against this coronavirus. Understanding this evolutionary trajectory and the factors that influence HCoV-OC43-human interaction could aid in understanding how emerging coronaviruses adapt to human hosts. Like other positive-sense single-stranded RNA viruses, coronaviruses replicate their genomes via dsRNA replication intermediates (V’kovski et al, 2021). Since dsRNA is a potent immune agonist, endogenous dsRNA is typically expressed at low levels (Chen and Hur, 2022). A recent coronavirus screen monitored virus-derived dsRNA to score viral replication (Schneider et al, 2021), which we adapted here for HCoV-OC43 and plate-based image cytometry (Fig. EV1A–C). Benefits of this system include its high-throughput nature and utility, where neither a recombinant viral clone nor viral antigen-specific antibodies are available. We titrated HCoV-OC43 on A549 lung adenocarcinoma cells, fixing cells at intervals post-infection. An increase in signal over time confirmed that detected dsRNA was a robust correlate of viral infection (Fig. 1A). Pre-treatment of A549 cells with type I IFN substantially reduced the dsRNA signal, suggesting that these cells provide a suitable context in which to study anti-HCoV-OC43 machinery (Fig. EV1D).

### Identification of genes with antiviral activity against HCoV-OC43

To search for genes with anti-HCoV-OC43 activity, we undertook a multi-species cDNA screen by transducing A549 cells with ISG libraries encoding human (*Homo sapiens*), rhesus macaque (*Macaca mulatta*) and bovine (*Bos taurus*) ISGs, encoded in lentiviral vectors arrayed in 96-well format, with one ISG expressed per well. Although not implicated in the emergence of HCoV-OC43, we included the macaque library to increase the range of ISGs screened; the macaque library contains ISGs shared by both humans and macaques, which are not present in the human library. Combined, these libraries encoded >500 human genes, >300 macaque genes and >250 bovine genes (Fig. 1B). In addition to an ISG, the lentiviral vector expresses the fluorescent protein TagRFP as an independent ORF (Fig. 1C), to allow quantification of transduction efficiency (Fig. EV1A–C).

After library transduction, we infected cells in parallel with a single dose of HCoV-OC43, determined previously to infect 30–50% of cells by 96 h post-infection (hpi) (Fig. 1A). Infection was scored in each well and presented here as relative to the mean of the species library (Fig. 1D). The antiviral activity of candidate human, macaque and bovine ISGs were re-tested in secondary screens, using independently prepared ISG lentiviruses, newly transduced cells, and repeat HCoV-OC43 infections (Fig. 1E–G). Bovine OAS2 failed to reach the transduction threshold, but its antiviral behaviour was confirmed by generating a stable expression cell line (Fig. EV1E). The efficacy of our approach was reinforced by the identification of the known antiviral protein LY6E across all three libraries. LY6E inhibits the replication of multiple coronaviruses, including HCoV-OC43, by interfering with membrane fusion at virus entry (Pfaender et al, 2020).

Other antiviral candidates from our screen included the endosomal protease cathepsin S (CTSS), the vRNA sensors OAS2 and DDX58/RIG-I, and the transcription factors ETV6 and IRF7. CTSS was previously identified in a CRISPR-activation screen against SARS-CoV-2, suggesting broad anti-CoV behaviour (Danziger et al, 2022). Cathepsins B (CTSB) and L (CTSL) proteolyse SARS-CoV-2 Spike protein, promoting virus entry in endosomes (Jackson et al, 2022), suggesting that CTSS overexpression may modulate HCoV-OC43 entry pathways. OAS2 has appeared in several genetic screens against CoVs (Mac Kain et al, 2022; Pfaender et al, 2020; Wickenhagen et al, 2021), however its anti-CoV activity has not been well characterised. RIG-I is a key cellular sensor of vRNA, and several reports also describe a role for RIG-I in sensing SARS-CoV-2 RNA (Marx et al, 2022; Thorne et al, 2021). ETV6 is poorly studied, but the related protein ETV7 has been shown to regulate the expression of ISGs that inhibit influenza A virus (Froggatt et al, 2021). IRF7 is a key transcription factor driving IFN signalling; IRF7 overexpression might be expected to inhibit viral infections via non-specific ISG induction.

To distinguish effectors acting directly vs indirectly on HCoV-OC43 infection, we next screened our candidate ISGs for their ability to induce an IFN response independently of viral infection. To do this, we expressed each ISG in A549 cells containing an interferon-stimulated response element (ISRE)-GFP reporter construct, whereby GFP expression is a proxy for IFN pathway activity. In doing so, we found that IRF1, IRF4, IRF7, IFNβ1 and RIG-I all caused >10-fold ISRE activation (Fig. 1H). We also used a cytotoxicity assay, which pinpointed TNFRSF10A and MAP3K14 as reducing cell viability, likely explaining the antiviral effects detected (Fig. 1I). After removing off-target genes, we could shortlist our putative anti-HCoV-OC43 ISGs (Fig. 1J).

Of these, we were most intrigued by OAS2. OAS enzymes synthesise short 2’-5’ phosphodiester-linked polyA (oligoadenylate) RNAs (2-5A) upon binding dsRNA (Marié et al, 1997). 2-5A behaves as a second messenger, binding the dormant endoribonuclease RNase L, inducing its dimerisation and activation (Dong and Silverman, 1995). Once unleashed, RNase L degrades host and viral RNAs, stemming viral replication whilst inhibiting cell proliferation (Ghosh et al, 2000; Maitra et al, 1995). The human OAS gene family (OAS1, OAS2, OAS3 and OASL) undergoes alternative splicing, generating multiple isoforms for OAS1 (p42, p44, p46, p48 and p52), OAS2 (p69 and p71) and OASL (p30 and p56-59) (Koul et al, 2024). Interestingly, HCoV-OC43 encodes a 2’-5’-phosphodiesterase (PDE) in its *ns2* gene, a described antagonist of the RNase L pathway via 2-5A hydrolysis (Goldstein et al, 2017). This viral countermeasure was thought to explain the resistance of HCoV-OC43 to OAS1, which potently inhibits SARS-CoV-2 (Wickenhagen et al, 2021). We were also surprised to identify OAS2 because in 33 additional ISG screens at the University of Glasgow, against diverse RNA viral families including *Lentiviridae*, *Togaviridae*, *Peribunyaviridae*, *Orthomyxoviridae* and *Coronaviridae*, OAS2 is notable in having only been identified as a putative antiviral in one (SARS-CoV-2), and in that example, with only very modest antiviral activity (1.7- 2.1-fold inhibition) (Fig. 1K) (Wickenhagen et al, 2021). Combined, this suggested that by studying this specific combination of virus and host enzymes, we might uncover novel antiviral biology.

### The p69 isoform of OAS2 shows antiviral activity against HCoV-OC43

**Figure EV2:**
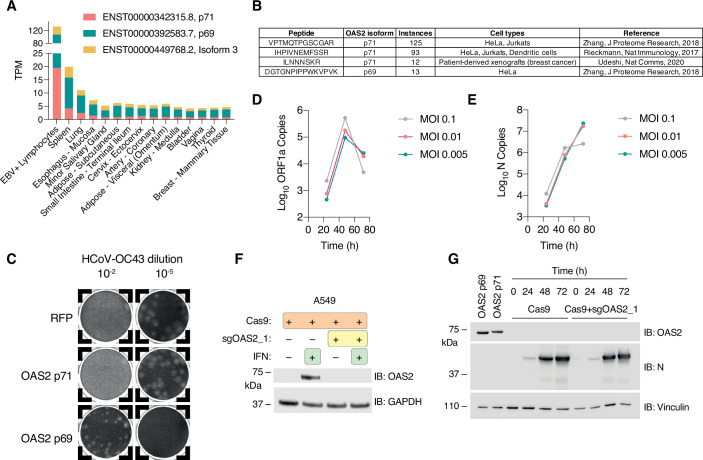
Presence of endogenous OAS2 isoforms. (**A**) Transcript expression of OAS2 isoforms across tissues analysed using the GTEx database. (**B**) The human peptide atlas (https://peptideatlas.org/) was searched for peptides from the OAS2 p71 and p69 C-terminus, generated by trypsin cleavage. (**C**) Representative images of HCoV-OC43 plaques formed in cell lines characterised in (Fig. 2C), 120 hpi. (**D**) HCoV-OC43 *ORF1a* transcript levels in A549 cells infected with HCoV-OC43 were quantified at multiple timepoints, by RT-qPCR. (**E**) HCoV-OC43 *nucleocapsid* transcript levels in A549 cells infected with HCoV-OC43 were quantified at multiple timepoints, by RT-qPCR. (**F**) Endogenous OAS2 levels in A549 cells transduced with a lentiviral vector-derived OAS2 sgRNA, with or without pre-treatment of 100 U/mL IFNβ for 24 h. (**G**) Expression of endogenous OAS2 was monitored in A549 cells from (**F**), infected with HCoV-OC43 (MOI 0.01), at multiple timepoints, by Western blotting.

**Figure 2:**
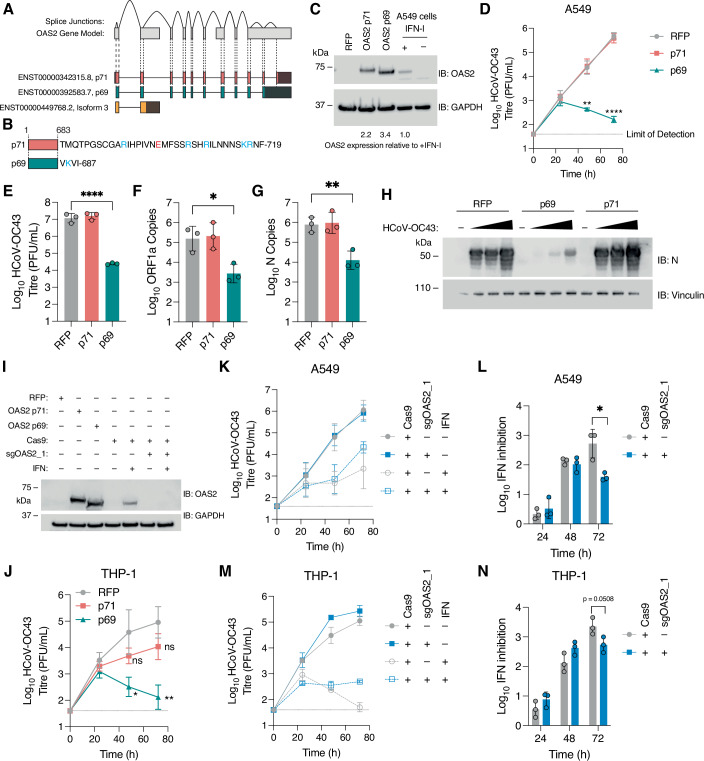
The p69 isoform of human OAS2 restricts HCoV-OC43 replication. (**A**) Representation of the OAS2 gene model including exon structures and splice junctions. The three major OAS2 transcripts are indicated, shaded boxes are non-coding, bright colouring indicates coding sequences. (**B**) Schematic of the OAS2 isoforms with the distinct C-termini sequences of p71 (NM_016817.3) and p69 (NM_002535.5) indicated. Basic residues are in blue, and acidic residues are in red. (**C**) A549 cells were modified to express RFP, OAS2-p71 or OAS2-p69, confirmed by Western blotting. A549 cells treated with 1000 U/mL IFNβ are additionally shown. (**D**) OAS2-expressing A549 cells from (**C**) were infected with HCoV-OC43 (MOI 0.01) and culture supernatant was collected at indicated timepoints to quantify infectious titre by plaque assay. (**E**) Infectious titre of HCoV-OC43 in cell lines generated in (**C**) were quantified by plaque assay at 120 hpi. (**F**) Quantification of HCoV-OC43 *ORF1a* transcripts in OAS2-expressing cells infected for 72 h (MOI 0.01) by RT-qPCR. (**G**) Quantification of HCoV-OC43 *nucleocapsid* (*N*) transcripts in OAS2-expressing cells infected for 72 h (MOI 0.01) by RT-qPCR. (**H**) N protein levels in A549 cells expressing OAS2 p69 or p71, or an RFP control, were compared by Western blotting, following infection with HCoV-OC43 (MOI of 0.005, 0.01 and 0.05) for 72 h (*n* = 1). (**I**) THP-1 cells were modified to express RFP, OAS2-p71 and OAS2-p69, or transduced with a lentiviral vector-derived OAS2 sgRNA, with or without pre-treatment of 100 U/mL IFNβ for 24 h. Expression of OAS2 was confirmed by Western blotting. (**J**) OAS2-expressing PMA-differentiated THP-1 macrophages from (**I**) were infected with HCoV-OC43 (MOI 0.01), and culture supernatant was collected at the indicated timepoints to quantify infectious titre by plaque assay. (**K**) OAS2-depleted A549 cells from (Fig. EV2F) were mock or pre-treated with 100 U/mL IFNβ for 24 h prior to infection with HCoV-OC43 (MOI 0.01). Culture supernatant was collected at indicated timepoints to quantify infectious titre by plaque assay. Statistics were performed as indicated in the data information for (**L**). (**L**) Fold IFNβ inhibition of HCoV-OC43 in OAS2-depleted A549 cells at the indicated timepoints measured in (**K**). (**M**) OAS2-depleted PMA-differentiated THP-1 macrophages from (**l**) were mock or pre-treated with 100 U/mL IFNβ for 24 h prior to infection with HCoV-OC43 (MOI 0.01). Culture supernatant was collected at indicated timepoints to quantify infectious titre by plaque assay. (**N**) Fold IFNβ inhibition of HCoV-OC43 in OAS2-depleted PMA-differentiated THP-1 cells at the indicated timepoints measured in (**M**). Data information: Data were presented as the mean ± SD of 3 biological replicates. (**D**, **J**, **L**, **N**) Data were analysed using pairwise Welch’s *t*-tests, where *p < 0.05, **p < 0.01, ***p < 0.001, and ****p < 0.0001. (**E**–**G**) Data were analysed by one-way ANOVA with Dunnett’s multiple comparison test (vs. RFP), where *p < 0.05, **p < 0.01, ***p < 0.001, and ****p < 0.0001. (**D**) **p = 0.0066, ****p < 0.0001. (**E**) ****p < 0.0001. (**F**) *p = 0.0193. (**G**) **p = 0.0060. (**J**) *p = 0.0364, **p = 0.0035. (**L**) *p = 0.0447. Source data are available online for this figure.

Our human ISG library only contained the p69 isoform, whereas the literature generally references two OAS2 transcripts, namely p69 and p71 (Koul et al, 2024; Kristiansen et al, 2010; Marié et al, 1999; Schwartz and Conn, 2019; Silverman, 2007). To investigate whether other OAS2 isoforms are also restrictive toward HCoV-OC43, we sought a panel of OAS2 transcripts to test. NCBI lists three OAS2 transcripts, while Ensembl lists fifteen, seven of which are predicted to encode protein. To corroborate this larger Ensembl dataset, we first referred to the Genotype-Tissue Expression Project (GTEx), which quantifies Ensembl transcript abundance across 54 tissues (Consortium, 2015). Despite Ensembl’s large collection of transcripts, only three transcripts were significantly expressed across a range of tissues (although notably these data derive from donors rather than IFN-treated cells, potentially underestimating the transcript repertoire of an immune response) (Fig. EV2A) – ENST00000342315.8, p71 (genbank NM_016817.3); ENST00000392583.7, p69 (NM_002535.3); and ENST00000449768.2, transcript 3, isoform 3 (NM_001032731.2) (Fig. 2A). The p71 and p69 transcripts encode the same core 683 residues but owing to an alternate splicing event near to the end of exon 10, an additional exon (exon 11) is formed, which includes a short in-frame coding sequence and stop codon, to give p71. Consequently, while p69 has a 4-residue C-terminal tail, p71 has a longer, 36-residue tail (Fig. 2B). Conversely, isoform 3 shares the first 150 residues with p69 and p71 but, by missing a splice site, has an extended exon 2 resulting in a unique 22-residue tail. Isoform 3 lacks key residues required for RNA binding and 2-5A synthesis, suggesting its role might be regulatory rather than the detection of viral RNA. Moreover, while the PeptideAtlas project (Desiere et al, 2006) provided empirical evidence for the expression of both p69 and p71 isoforms in diverse cell types, there are no recorded peptides unique for isoform 3, suggesting this isoform might not be expressed (Fig. EV2B). Thus, we decided to exclude OAS2 isoform 3 from further investigation.

We next stably expressed the p69 or p71 isoforms, or an RFP control, in A549 cells; Western blots confirmed expression, and the isoforms exhibited modest differential electrophoretic mobility (Fig. 2C). Parallel electrophoresis of lysates from A549 cells stimulated with IFN suggested that p69 was the predominant endogenous isoform expressed in these cells (Fig. 2C). We next infected our OAS2 overexpression cells with a low multiplicity of HCoV-OC43 and performed multi-step growth curves to monitor viral replication. Strikingly, while HCoV-OC43 replicated with comparable kinetics in the RFP-expressing and OAS2 p71-expressing cells, replication in OAS2 p69-expressing cells was markedly impaired, exhibiting a ~2500-fold drop in released viral titre by 72 hpi (Fig. 2D). The effect was also observed by directly measuring plaque formation in RFP- or OAS2-expressing cells at 120 hpi (Figs. 2E and EV2C). Next, we developed a qPCR assay to quantify relative HCoV-OC43 genome copies using probes targeting either the 5’ end of the genome (ORF1a/b) or the 3’ subgenomic region (Nucleocapsid, N). Levels of these targets were examined over 72 h in parental A549 cells; ORF1a amplicons peaked at 48 hpi at all MOIs tested (Fig. EV2D), whereas N amplicons increased continuously at all MOIs tested (Fig. EV2E). Supporting the reduction in replicating virus, in p69-expressing cells at 72 hpi, we observed a 60-fold and 50-fold decrease in genomic (ORF1a/b) and genomic+subgenomic (N) transcripts, respectively, compared to RFP- or p71-expressing cells (Fig. 2F,G). In alignment, we also observed substantial inhibition of HCoV-OC43 N protein expression at 72 hpi in p69-expressing cells compared to RFP- or p71-expressing cells (Fig. 2H). Although genomes, subgenomes or proteins are not measures of infectious virus, the agreement between these surrogate markers and infectious titres (Fig. 2E) demonstrates they serve as useful correlates of replication. Combined, these data reveal a potent antiviral activity of OAS2 p69, but not p71, against HCoV-OC43 at the transcript, protein, and infectious particle level.

To test whether this restriction pattern was unique to A549, we also expressed RFP, p71 and p69 in THP-1 monocytes (Fig. 2I). Following their differentiation into macrophage-like cells, we infected at low MOI for multi-step growth, quantifying virus release by plaque assay on A549 cells. In THP-1 as in A549 cells, p69 induced the most robust inhibition of HCoV-OC43, exhibiting a 200- and 860-fold inhibition at 48 and 72 hpi, respectively (Fig. 2J). Unlike in A549 cells, in THP-1 we also observed a consistent and significant 9-fold inhibition of viral growth by p71 at 72 hpi, indicating that the microenvironment can influence restriction specificity and potency (Fig. 2J). Nonetheless, in this background, p69 displayed ~100-fold more potent anti-HCoV-OC43 restriction than p71.

### Endogenous OAS2 contributes to IFN-mediated suppression of HCoV-OC43 replication

The antiviral effect of IFNs against any single virus can be the cumulative activity of a small subset of ISGs (McDougal et al, 2023). A549 cells express the restrictive p69 isoform upon IFN stimulation (Fig. 2C), so we hypothesised that OAS2 could contribute to IFN’s inhibition of HCoV-OC43 in this setting (Fig. EV1D). We used CRISPR/Cas9 to reduce OAS2 expression in A549 cells, confirming depletion by Western blot (Fig. EV2F). We then pre-treated control or OAS2-depleted cells with 100 U/mL IFN-I before performing HCoV-OC43 growth curves and measuring released infectious titres. As expected, IFN-I treatment of Cas9-only control cells caused substantial inhibition of viral replication, manifest as 715-fold inhibition at 72 hpi (Fig. 2K,L). In cells depleted of OAS2, IFN was less antiviral, inducing a 40-fold inhibition of replication at 72 hpi (Fig. 2K,L). In the absence of IFN treatment, OAS2 depletion had no effect on HCoV-OC43 replication, likely explained by low baseline OAS2 expression in either unstimulated cells (Fig. 2C) or during HCoV-OC43 replication (Fig. EV2G). To further investigate a role for endogenous OAS2 in HCoV-OC43 replication, we depleted its expression in differentiated THP-1 cells (Fig. 2I), before treating cells with or without IFN and performing HCoV-OC43 replication experiments. As in A549, IFN treatment caused strong inhibition of HCoV-OC43 replication, manifest as 2,500-fold inhibition at 72 hpi (Fig. 2M,N). Also, as in A549 cells, OAS2 depletion reduced IFN efficacy at later timepoints, by approximately threefold in this setting (Fig. 2M,N). Unlike in A549 cells, we also observed a modest but reproducible rescue in HCoV-OC43 infectious titre upon OAS2 depletion, in the absence of IFN stimulation (Fig. 2M,N). A key difference between these two cell types is that THP-1 cells likely express higher levels of IFIH1/MDA5 (an interferon-inducing RIG-I-like receptor) at baseline compared to A549 (Lama et al, 2026). It is therefore possible that THP-1 cells mount an endogenous interferon response that induces OAS2 in wild-type cells, which in turn controls the virus. However, the lack of a complete rescue to viral replication in OAS2-depleted cells is in keeping with studies demonstrating that IFN inhibits viral replication via a cohort of ISGs; only by synchronously removing a complete ‘ISG syndicate’ is IFN rendered ineffective against a specific virus (McDougal et al, 2023). Moreover, we note that depletion of the related enzyme OAS1, in some contexts, has no effect on the replication of SARS-CoV-2 in vitro but has a substantial effect on SARS-CoV-2 replication in vivo (Lee et al, 2023; Wickenhagen et al, 2021). Combined, these experiments support a role for endogenous OAS2 in IFN-mediated restriction of HCoV-OC43 in A549 cells.

### *N*-myristoylation is necessary for OAS2 p69 antiviral activity

**Figure 3:**
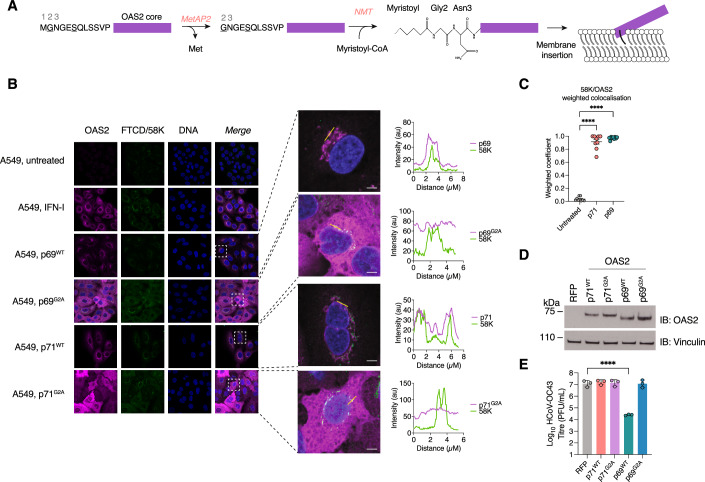
Myristoylation is required for the antiviral activity of OAS2 p69. (**A**) *N*-myristoylation of OAS2 proteins. The initiator methionine is removed by methionine aminopeptidase 2 (MetAP2) prior to N-myristoyltransferase (NMT) catalysing the addition of the myristoyl group to the glycine residue at the N-terminus. (**B**) A549 cells stimulated with 1000 U/mL IFNβ, or A549 cells expressing p71, p69, p71^G2A^ or p69^G2A^, were immunostained for OAS2 (magenta), anti-FTCD/58 K (green) and DNA (blue). Signal intensity profiles along the lines of the magnified boxes are shown. (**C**) Weighted coefficients between p71 and p69 isoforms and Golgi marker 58 K; Each data point represents a separate region of interest from a representative experiment, *n* = 2. (**D**) A549 cells were modified to express the G2A variants of OAS2, confirmed by Western blotting. (**E**) The infectious titre of HCoV-OC43 in cell lines generated in (**D**) were determined by plaque assay at 120 hpi; data were presented as the mean ± SD of three biological replicates. Data information: (**C**, **E**) Data were analysed by one-way ANOVA with Dunnett’s multiple comparison test (vs. untreated (**C**) or RFP (**E**)), where ****p < 0.0001. Source data are available online for this figure.

**Figure EV3:**
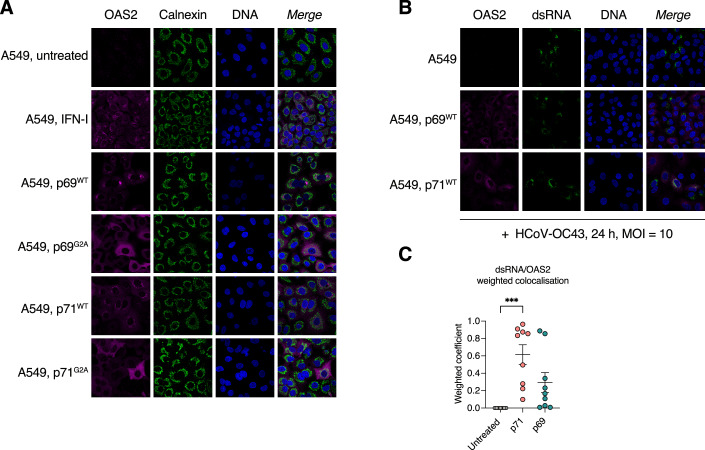
N-terminal myristoylation activity is required for antiviral activity. (**A**) A549 cells stimulated with 1000 U/mL IFNβ or A549 cells expressing p71, p69, p71^G2A^ or p69^G2A^, were immunostained for OAS2 (magenta), calnexin (green) and DNA (blue). (**B**) Representative immunofluorescence images of A549 cells expressing p71 or p69 infected with HCoV-OC43 (MOI = 10) for 24 h and immunostained for OAS2 (magenta), dsRNA (green) and DNA (blue). (**C**) Weighted coefficients between p71 and p69 isoforms and dsRNA. Each data point represents a separate region of interest from a representative experiment, *n* = 2. Data were presented as the mean ± SEM and analysed by one-way ANOVA with Dunnett’s multiple comparison test, ***p = 0.0008.

The p46 isoform of OAS2-related enzyme OAS1 requires anchoring to intracellular membranes to sense coronavirus and picornavirus replication, achieved via post-translational prenylation at its C-terminal CaaX motif (Soveg et al, 2021; Wickenhagen et al, 2021). Early characterisation of OAS2 p69 showed the protein was *N*-myristoylated in vitro, and this was hypothesised to facilitate its association with membranes (Marié et al, 1990). *N*-myristoylation is a distinct, co-translational lipid modification catalysed by the enzyme *N*-myristoyltransferase (NMT) and can direct protein membrane targeting (Fig. 3A) (Tate et al, 2024). Certain features of the OAS2 N-terminal peptide, including a glycine immediately after the initiator methionine, and a serine at position 5 (Fig. 3A), make OAS2 an ideal NMT substrate (Johnson et al, 1994). We first investigated the subcellular appearance of endogenous OAS2 using A549 cells treated with or without type I IFN for 24 h, which induces expression of the p69 isoform (Fig. 2C). OAS2 formed a reticular pattern that did not localise with ER marker calnexin (Figs. 3B and EV3A) but did with the Golgi marker formiminotransferase cyclodeaminase FTCD/58 K (Bloom and Brashear, 1989) (Fig. 3B,C). This distribution was similar to that of the ectopic OAS2 isoforms (Figs. 3B and EV3A). To investigate a role for lipidation in OAS2 function, we substituted glycine at position 2 with alanine, which inhibits lipidation by NMT (Borgese et al, 1996; Deichaite et al, 1988), and stably expressed these proteins in A549 cells (Fig. 3D). In contrast to their WT counterparts, G2A variant OAS2 proteins were diffusely expressed and lost specific Golgi localisation (Fig. 3B), supporting a role for *N*-myristoylation in OAS2 membrane association.

To look for OAS2-dsRNA interaction in cells, we infected A549 cells expressing p69 or p71 with HCoV-OC43 for 24 h and stained cells with the same dsRNA-specific antibody used in our initial screen. In this way, we observed that both p69 and p71 colocalised with dsRNA, although only the correlation for p71 achieved statistical significance (*p* < 0.05), perhaps indicative of aborted viral replication in the presence of p69 (Fig. EV3B,C). To test whether OAS2 N-myristoylation contributes to viral restriction, we infected A549 cells expressing the WT or G2A-substituted p69 or p71 variants with HCoV-OC43 and measured viral replication by plaque assay. As before, p69 robustly inhibited HCoV-OC43 replication, but this was completely reversed by G2A substitution (Fig. 3E). Importantly, in vitro studies demonstrate that p69 myristoylation is not necessary for enzymatic activity per se (Sarkar et al, 1999a). Thus, these data support a model where myristoylation facilitates OAS2 recruitment to membranes proximal to HCoV-OC43 replication compartments, where it can bind viral RNA and inhibit viral replication.

### RNA binding correlates with OAS2 p69 antiviral activity

**Figure EV4:**
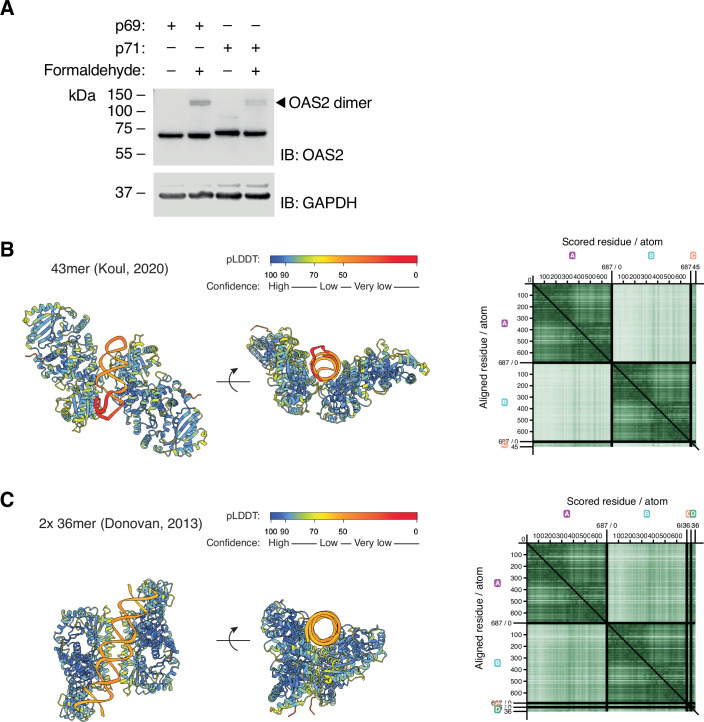
AlphaFold 3 models of OAS2:dsRNA. (**A**) A549 cells expressing OAS2 p71 or p69, treated with or without 0.5% formaldehyde. OAS2 expression assessed by Western blotting. (**B**, **C**) Top-ranking AlphaFold3 predictions of human OAS2 p69 in complex with dsRNA 43mer palindromic RNA (Koul et al, 2020a) (**B**) or 2x36mer RNA (Donovan et al, 2013) (**C**), coloured according to pLDDT scores as indicated by key. Predicted Aligned Error (PAE) plots for the top-ranking structures are shown, which indicate high confidence in the conformation of individual OAS2 structures, and lower confidence in the relative position of one OAS2 chain to the other.

To test that interaction with RNA is necessary for the observed restriction activity, we sought to reduce p69 binding to viral RNA. In the absence of an experimentally determined OAS2-dsRNA structure, we used the AlphaFold3 (AF3) Server to generate a series of structural predictions (Abramson et al, 2024). As RNA ligands, we used two experimentally optimised ligands: one copy of a palindromic 43mer that activated OAS2 in vitro (Koul et al, 2020a), or two copies of an 18mer that co-crystallised with OAS1 (GGCUUUUGACCUUUAUGC), repeated once (2x36mer) (Donovan et al, 2013). We reasoned that comparing complexes with distinct RNAs could help in defining the interface. OAS2 forms a homodimer in solution (Koul et al, 2020b; Merold et al, 2025; Sarkar et al, 1999a), which we corroborated by chemical cross-linking in cells (Fig. EV4A), so we included two copies of OAS2 in all predictions. Confidence metrics of the individual OAS2 protomers were high (pLDDT >70) (Fig. EV4B,C), although the relative arrangement of opposing molecules was variable, as indicated by predicted aligned error (PAE) plots of the top-ranked structures (Fig. EV4B,C). In contrast, expected position error values between OAS2 monomers and RNA were between 0 and 5 Å, suggesting higher confidence, particularly for the complex with 2x36mer RNA (Fig. EV4C). In both structures, RNA was sandwiched between antiparallel OAS2 protomers. The singlet palindromic 43mer appeared as a hairpin, contacting only the catalytic OAS domains (DII), while the helical 2x36mer appeared as a linear duplex running along a surface of the catalytic OAS DII domain as well as a corner of the inactive DI domain (Fig. EV4B,C). While residues in DI, including R165, D168, N278, H282 and Q285, sit within 5 Å of dsRNA in these predictions, for the purposes of ablating RNA binding, we focused on the conserved residues in DII that are important for OAS1-dsRNA binding (Donovan et al, 2013).

**Figure 4:**
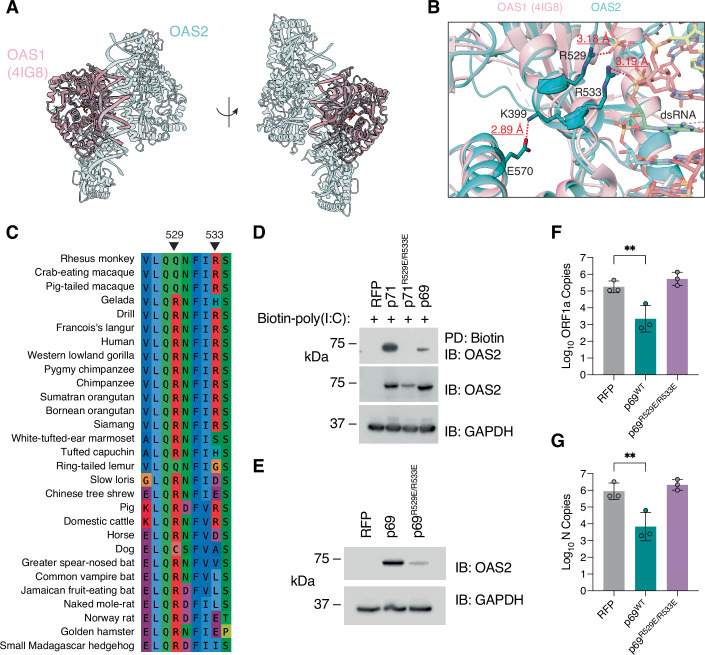
RNA binding residues are necessary for OAS2 antiviral activity. (**A**) Superposition of OAS1:18mer RNA (Donovan et al, 2013) (PDB: 4IG8) with OAS2 p69:2x36mer (Fig. EV4C). (**B**) Close up of the E570:K399 salt bridge, analogous to that identified in OAS1:18mer (Donovan et al, 2013), and displaced R529 and adjacent R533, in proximity of the RNA phosphate backbone; predicted structure superimposed with OAS1:18mer crystal structure (PDB: 4IG8) (Donovan et al, 2013). (**C**) OAS2 multiple sequence alignments across primate and mammalian orthologs, focusing on human R529 and R533. (**D**) Biotin-poly(I:C) pulldown from cell extracts expressing RFP, p71, p71^R529E/R533E^ or p69, eluates and input sample probed for OAS2 expression by Western blot. (**E**) A549 cells were modified to express p69 with substitutions predicted to disrupt RNA binding (p69^R529E/R533E^), confirmed by Western blotting. (**F**) HCoV-OC43 *ORF1a* levels were measured in cells characterised in (**E**), infected with HCoV-OC43 (MOI 0.01) for 72 h, by RT-qPCR. (**G**) HCoV-OC43 *nucleocapsid* levels were measured in cells characterised in (**E**), infected with HCoV-OC43 (MOI 0.01) for 72 h, by RT-qPCR. Data information: In (**F**, **G**), data were presented as mean ± SD of three biological replicates. Data were analysed by one-way ANOVA with Dunnett’s multiple comparison test (vs. RFP), where **p < 0.01. (**F**) **p = 0.0096. (**G**) **p = 0.0086. Source data are available online for this figure.

Focussing on the OAS2-2x36mer prediction, we observed that this superimposed well with the OAS1-dsRNA crystal structure (root mean square deviation (RMSD) value between 305 pruned atom pairs was 0.685 Å) (Fig. 4A), with several conserved side chains stabilising interaction with RNA. In particular, the prediction captured a key conformational change that occurs upon dsRNA binding in OAS1 (Donovan et al, 2013). In an apo structure of porcine OAS1 (Hartmann et al, 2003), residue R195 (human OAS1 numbering) forms a salt bridge with an internal glutamate (E233). Upon dsRNA binding, distant residue K66 moves 18 Å to exchange places with R195, displacing R195 by 11 Å and causing it to rotate and form hydrogen bonds with RNA backbone phosphates (Donovan et al, 2013). In the predicted OAS2-2x36mer structure, the mobile K66-equivalent K399 is already seen forming a salt bridge with the same glutamate (E570), allowing R529 (porcine OAS1 R195) to interact with RNA (Fig. 4B). Nearby OAS2 residue R533, also conserved in OAS1, similarly forms hydrogen bonds with RNA phosphates (Fig. 4B). Charge swapping these two basic residues in porcine OAS1 (R194E/R198E) caused a ~10,000 reduction in dsRNA-dependent 2-5A catalysis (Hartmann et al, 2003). Of note, in OAS2, the displaced R529 and, to a lesser extent, the nearby R533, are conserved in bovine, equine, rodent, porcine and primate OAS2 sequences, although interestingly, human R529 is a glutamine in the *Macaca* sequences analysed (Fig. 4C). R529E/R533E substitution in human OAS2 was recently shown to ablate function in a cellular reporter assay for 2-5A synthesis (Merold et al, 2025).

To test dsRNA binding between WT and variant OAS2 proteins, we exploited the observation that OAS2 proteins can bind to and be stimulated by poly(I:C) (Koul et al, 2020a; Li et al, 2016). We incubated biotinylated poly(I:C) in cell extracts expressing these OAS2 variants or their parental isoforms, enriching against streptavidin and resolving eluates by SDS-PAGE and Western blot. By this approach, we were able to capture both parental p71 and, to a lesser extent, p69 isoforms with poly(I:C) (Fig. 4D). Owing to this weaker p69 binding, we evaluated the R529E/R533E substitution in the context of p71, finding it strongly inhibited RNA binding (Fig. 4D), supporting the prior studies that used this variant.

We next infected cells expressing p69 and p69^R529E/R533E^ with HCoV-OC43 (Fig. 4E) and used qPCR to monitor *ORF1a* and *N* transcript copies following infection. As before, p69 caused a significant 35-fold reduction in *ORF1a* relative to the RFP control, which was abolished in p69^R529E/R533E^ cells (Fig. 4F). Similarly, we measured a 60-fold reduction in *N* transcripts in p69^WT^ cells, but no reduction in p69^R529E/R533E^ cells, compared to the RFP control (Fig. 4G). While the lower expression level of the RNA-binding mutant might also contribute to the absence of measurable restriction, the ability of OAS2 p69 to interact with dsRNA in vitro tracks with restriction potency in cells.

### OAS2 p69 inhibits HCoV-OC43 independently of RNase L

**Figure 5:**
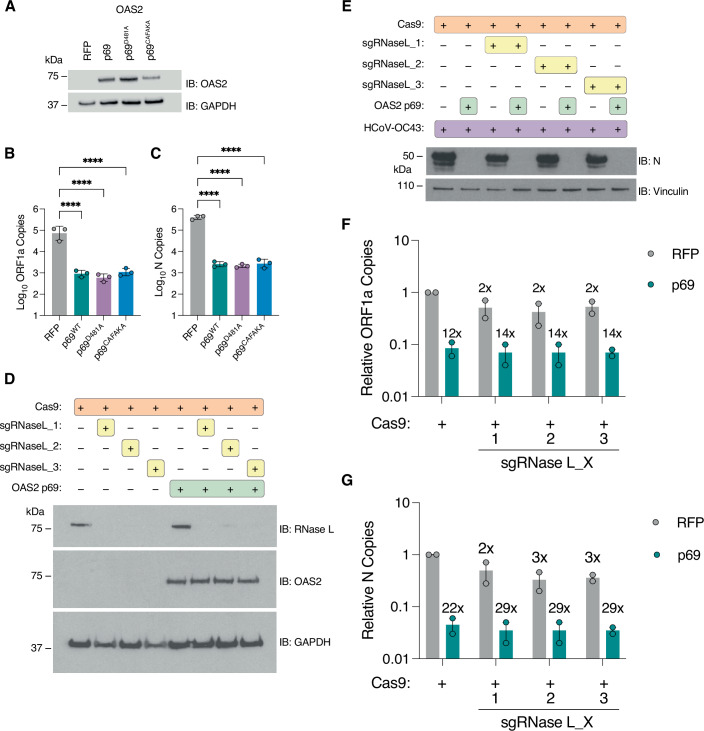
OAS2 p69 restriction of HCoV-OC43 is RNase L-independent. (**A**) A549 cells were modified to express a catalytic variant of p69 (D481A) or a CFK motif variant (CAFAKA), confirmed by Western blotting. (**B**, **C**) A549 cells from (**A**) were infected with HCoV-OC43 for 72 h, and *ORF1a* (**B**) or *nucleocapsid* (**C**) transcripts were quantified by RT-qPCR; data were presented as the mean ± SD of three biological replicates. Data were analysed by one-way ANOVA with Dunnett’s multiple comparison test (vs. RFP), where ****p < 0.0001. (**D**) A549 cells expressing RFP or OAS2 p69 were depleted of RNase L using three sgRNAs, and levels of RNase L and OAS2 were confirmed by Western blotting. (**E**) Cells from (**D**) infected with HCoV-OC43 for 72 h and levels of N protein determined by Western blotting. (**F**, **G**) Cells from (**D**) were infected with HCoV-OC43 for 48 h, and *ORF1a* (**F**) or *nucleocapsid* (**G**) transcripts were quantified by RT-qPCR; data were presented as the mean of two biological replicates. Source data are available online for this figure.

As described above, the canonical antiviral activity of OAS proteins occurs via synthesis of 2-5A, which activates RNase L. Because OC43 encodes a phosphodiesterase that degrades 2-5A (Goldstein et al, 2017), we were intrigued as to how OAS2 p69 could inhibit HCoV-OC43 replication. Accordingly, we introduced a mutation to disrupt the catalytic triad of aspartic acids (D408, D410 and D481), which collectively coordinate two catalytic metal ions in all enzymes of the nucleotidyl transferase superfamily, including OASes, and are required for 2-5A synthesis (Donovan et al, 2013; Koul et al, 2020b; Sarkar et al, 1999b). OAS2 D481 is also in proximity to the site of the donor ATP molecule (Sarkar et al, 2002), and the D481A variant in OAS2, or equivalent D148A in OAS1, are both catalytically inactive (Donovan et al, 2013; Koul et al, 2020b). In addition, we generated a variant substituted at the previously proposed CFK dimerisation residues (CAFAKA) (Ghosh et al, 1997). We expressed these variants in A549 cells; p69^CAFAKA^ expression was lower than that of p69^WT^, whereas p69^D481A^ expression was equivalent to that of p69^WT^ (Fig. 5A). We then infected these cells with HCoV-OC43 and measured viral transcripts at 72 hpi. As expected, HCoV-OC43 *ORF1a* transcripts were significantly lower in the cells expressing p69^WT^ compared to the RFP control. However, surprisingly, the same restriction was observed in cells expressing the catalytically dead variant p69^D481A^, as well as the p69^CAFAKA^ variant (Fig. 5B). Similarly, a significant reduction was observed in HCoV-OC43 *N* transcripts in the p69^WT^, p69^D481A^, p69^CAFAKA^ cells compared to the RFP control (Fig. 5C). The lack of effect of CAFAKA substitution is supported by recent in vitro studies where CAFAKA mutation was insufficient to disrupt OAS2 oligomerisation and activity (Koul et al, 2020b), as well as the recently published OAS2 cryo-EM structure, which shows these residues are not located at the dimer interface (Merold et al, 2025). However, the conservation of viral restriction by p69^D481A^ supports a model whereby p69 restricts HCoV-OC43 despite the viral PDE, via a mechanism independent of 2-5A synthesis and RNase L function.

To test this directly, we stably depleted RNase L from A549 cells expressing RFP or OAS2 p69 using CRISPR/Cas9, assessing RNase L depletion by Western blot (Fig. 5D). Despite robust RNase L depletion, the ability of OAS2 p69 to inhibit viral Nucleocapsid translation was unaltered compared to control cells (Fig. 5E). We also measured viral *ORF1a* and *N* transcripts at 48 hpi, which confirmed robust RNase L-independent p69 restriction of viral transcription (Fig. 5F,G). Combined with the full activity of the catalytically dead p69^D481A^ variant, these data suggest that OAS2 p69 restricts HCoV-OC43 in a manner independent of the canonical 2-5A/RNase L pathway.

### OAS2 p69 restricts HCoV-OC43 independently of IFN

**Figure 6:**
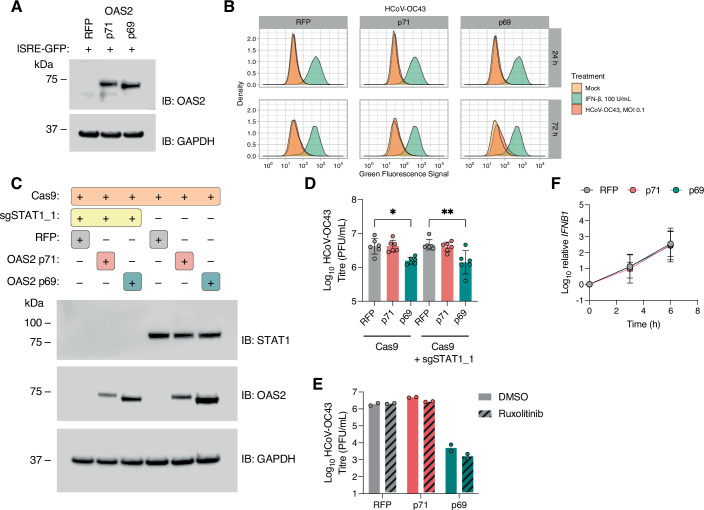
OAS2 operates independently of IFN signalling. (**A**) A549-ISRE:GFP cells were modified to express RFP, OAS2-p71 or OAS2-p69, confirmed by Western blotting. (**B**) A549-ISRE:GFP cells from (**A**) were treated with 100 U/mL IFNβ or infected with HCoV-OC43 (MOI 0.1). Stimulation of the ISRE was assessed by GFP expression, using flow cytometry at 24 h and 72 h. The fluorescence signal is presented as a representative histogram. (**C**) A549 cells were transduced with a lentiviral vector-derived STAT1 sgRNA, before modification to express RFP, OAS2-p71 and OAS2-69. OAS2 and STAT1 were confirmed by Western blotting. (**D**) Infectious titre of HCoV-OC43 in STAT1-depleted A549 cells from (**C**), was measured by plaque assay at 120 hpi. Data is presented as the mean ± SD of 3 biological replicates. Data were analysed by one-way ANOVA with Tukey’s multiple comparison test, where *p < 0.05, **p < 0.01, ***p < 0.001 and ****p < 0.0001. *p = 0.0129, **p = 0.0017. (**E**) OAS2-expressing A549 cells from (Fig. 2C) were treated with DMSO or 0.5 μM Ruxolitinib for 1 h prior to infection with HCoV-OC43. Infectious titre was determined by plaque assay at 120 hpi. Data is presented as the mean of two biological replicates. (**F**) OAS2-expressing A549 cells from (Fig. 2C) were transfected with 1 μg/mL poly(I:C) followed by quantification of IFNβ transcripts at the indicated timepoints by RT-qPCR. Data were presented as the mean ± SD of three biological replicates. Data were analysed using pairwise Welch’s *t*-tests, and no statistical significance was found. Source data are available online for this figure.

To elucidate the mechanism by which OAS2 p69 restricts viral replication independently of RNase L, we first asked whether this isoform stimulated IFN production upon virus detection, causing IFN-dependent HCoV-OC43 inhibition (Fig. EV1D). However, several subsequent experiments failed to provide evidence to support this model. First, we monitored the activity of an interferon-sensitive response element (ISRE) promoter coupled to GFP—wherein GFP expression serves as a readout for IFN signalling—in A549 cells expressing RFP, OAS2 p71 or OAS2 p69 (Fig. 6A). While exogenous IFN-I treatment (100 U/mL) caused robust activation of the ISRE reporter in all cell lines, HCoV-OC43 infection induced only weak ISRE activation in the presence of p69, at 72 hpi, and not in cells expressing p71 or RFP (Fig. 6B). This suggested that IFN had indeed been induced – albeit weakly – during p69-mediated restriction. Second, to ask whether IFN was functionally relevant to the p69 restriction phenotype, we ablated IFN signalling genetically, via STAT1 depletion using CRISPR/Cas9 (Fig. 6C), or pharmacologically, using JAK inhibitor ruxolitinib during infections. Neither approach blunted the antiviral activity of OAS2 p69 (Fig. 6D,E), suggesting its restriction occurred independently of IFN signalling. Third, we considered the recently proposed model that OAS1 can bind and stabilise *IFNB1* mRNA following poly(I:C) transfection (Harioudh et al, 2024). However, in cells overexpressing RFP, OAS2 p71 or OAS2 p69, we observed no difference in *IFNB1* transcript stability following poly(I:C) transfection (Fig. 6F), suggesting p69-mediated inhibition of HCoV-OC43 is distinct from OAS1 inhibition of West Nile Virus. Combined, these experiments suggest that OAS2 p69 restricts HCoV-OC43 via a mechanism unrelated to IFN-mediated inhibition of virus replication.

### The p71 isoform of OAS2 restricts EMCV in an RNase L-dependent manner

We were intrigued by the RNase L-independence of OAS2 p69, which contradicts reports that OAS2 operates via 2-5 A synthesis (Lee et al, 2023; Merold et al, 2025). A recent report revealed that human OAS1 can exhibit both RNase L-dependent and independent restriction mechanisms, in a virus-specific manner (Harioudh et al, 2024). To explore whether OAS2 might execute RNase L-dependent restriction under alternative scenarios, we tested its ability to restrict the picornavirus cardiovirus A (EMCV). Like HCoV-OC43, EMCV replicates in membranous organelles (Romero-Brey and Bartenschlager, 2014) and was shown to be sensitive to OAS2 (Marié et al, 1999) and OAS1 (Wickenhagen et al, 2021).

**Figure 7:**
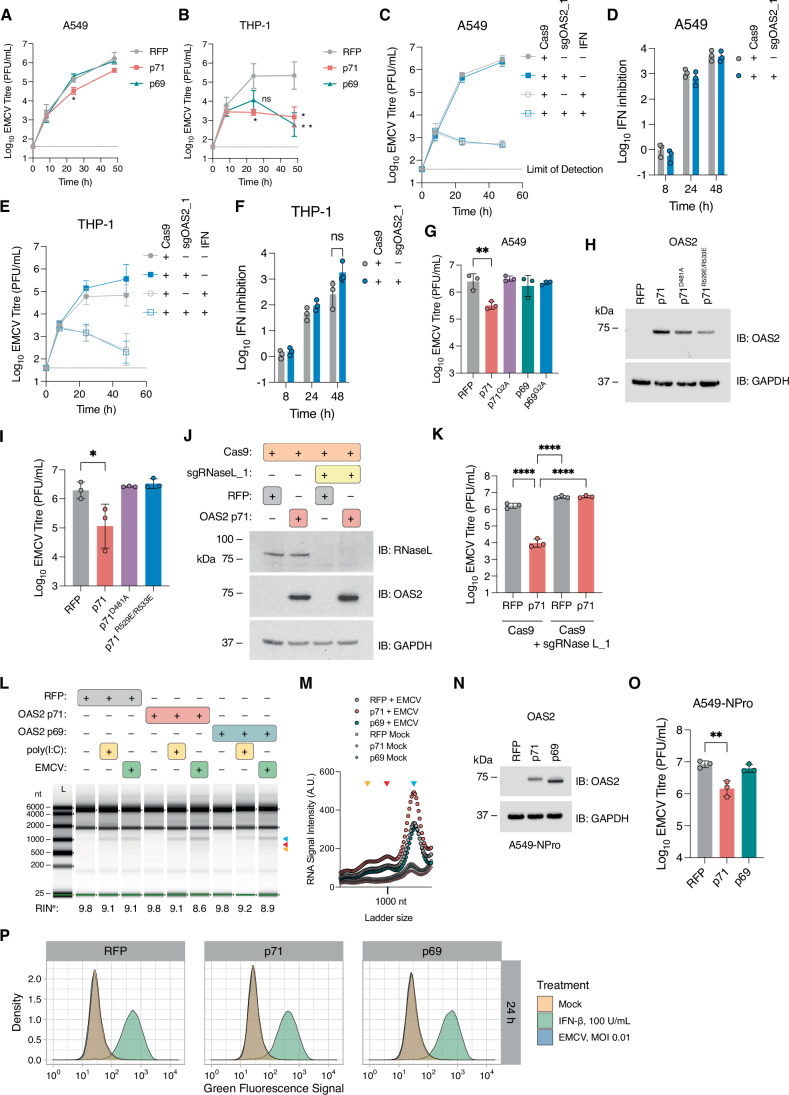
EMCV is restricted by OAS2 p71 in an RNase L-dependent manner. (**A**) A549 cells expressing RFP, p71 or p69 from (Fig. 2C) were infected with EMCV (MOI 0.005) and culture supernatant was collected at indicated timepoints to quantify infectious titre by plaque assay. (**B**) PMA-differentiated THP-1 macrophages expressing RFP, p71 or p69 from (Fig. 2I) were infected with EMCV (MOI 0.005) and culture supernatant was collected at indicated timepoints to quantify infectious titre by plaque assay. (**C**) OAS2-depleted A549 cells from (Fig. EV2F) were mock or pre-treated with 100 U/mL IFNβ for 24 h prior to infection with EMCV (MOI 0.005). Culture supernatant was collected at indicated timepoints to quantify infectious titre by plaque assay. (**D**) Fold IFNβ inhibition of EMCV in OAS2-depleted A549 cells at indicated timepoints measured in (**C**). (**E**) PMA-differentiated THP-1 macrophages from (Fig. 2I) were mock or pre-treated with 100 U/mL IFNβ for 24 h prior to infection with EMCV (MOI 0.005). Culture supernatant was collected at indicated timepoints to quantify infectious titre by plaque assay. (**F**) Fold IFNβ inhibition of EMCV in OAS2-depleted PMA-differentiated THP-1 macrophages at the indicated timepoints measured in (**E**). (**G**) Infectious titre of EMCV in cell lines expressing OAS2 isoforms or their respective G2A variants (Fig. 3D), determined by plaque assay at 30 hpi. (**H**) A549 cells were modified to express p71 with substitutions that disrupt catalytic activity (p71^D481A^) and RNA binding (p71^R529E/R533E^), confirmed by Western blotting. (**I**) Infectious titre of EMCV in A549 cells expressing p71^D481A^ and p71^R529E/R533E^characterised in (**H**), determined by plaque assay at 30 hpi. (**J**) A549 cells expressing RFP or p71 were transduced with a lentiviral vector-derived RNase L sgRNA, and expression levels of RNase L and OAS2 were confirmed by Western blotting. (**K**) Infectious titre of EMCV in the RNase L-depleted A549 cells characterised in (**J**), determined by plaque assay at 30 hpi. (**L**) A549 cells expressing RFP, p71 and p69 were transfected with 1 μg/mL poly(I:C) or infected with EMCV (MOI 2). Cells were lysed 24 hpi and RNA integrity analysed using a Tapestation. Blue, red and yellow arrows indicate the three RNA degradation peaks that are elevated in p71-expressing cells relative to p69 and RFP-expressing cells, when infected with EMCV compared to mock infection. L = ladder. (**M**) RNA signal intensity from (L) at the region indicated by blue, red and yellow arrows. Traces for A549 cells expressing RFP, p71 and p69, infected with EMCV or mock-infected. (**N**) A549-NPro cells were modified to express RFP, OAS2-p71 and OAS2-p69, confirmed by Western blotting. (**O**) Infectious titre of EMCV in A549-NPro characterised in (**N**), determined by plaque assay at 30 hpi. (**P**) A549-ISRE:GFP cells from (Fig. 6A) were treated with 100 U/mL IFNβ or infected with EMCV (MOI 0.01). Stimulation of the ISRE was assessed by GFP expression, using flow cytometry at 24 h. The fluorescence signal is presented as a representative histogram. Data information: Data were presented as the mean ± SD of three biological replicates. (**A**, **B**, **D**, **F**) Data were analysed using pairwise Welch’s *t*-tests, where *p < 0.05, **p < 0.01, ***p < 0.001, and ****p < 0.0001. (**G**, **I**, **O**) Data were analysed by one-way ANOVA with Dunnett’s multiple comparison test (vs. RFP), where *p < 0.05, **p < 0.01, ***p < 0.001 and ****p < 0.0001. (**K**) Data were analysed by one-way ANOVA with Tukey’s multiple comparison test, where *p < 0.05, **p < 0.01, ***p < 0.001 and ****p < 0.0001. (**A**) *p = 0.0132. (**B**) *p = 0.0302 (24 h), *p = 0.0165 (30 h), **p = 0.0100. (**G**) **p = 0.0030. (**I**) *p = 0.0164. (**K**) ****p < 0.0001. (**O**) **p = 0.0026. Source data are available online for this figure.

We first performed EMCV growth curve experiments in A549 cells expressing RFP, OAS2 p69 or p71. Surprisingly, in this case, OAS2 p71, but not p69, inhibited EMCV replication (Fig. 7A). We also performed the same EMCV replication experiments in THP-1 cells expressing RFP, OAS2 p71 or p69. Again, OAS2 p71 was strongly inhibitory to EMCV replication, although in this case OAS2 p69 was also antiviral (Fig. 7B). Notably, both isoforms were also shown to restrict EMCV when expressed in murine cells (Marié et al, 1999), indicating that context can also influence OAS2 antiviral responses. In these two cell types, endogenous OAS2 appeared to play only a minimal role in IFN-mediated inhibition of EMCV (Fig. 7C–F), which correlates with the apparent induction of OAS2 p69, and not p71, by IFN in both of these cell types (Fig. 2C,I). Indeed, the modest reduction in IFN efficacy against EMCV in OAS2-depleted THP-1 cells is supported by the ability of p69 to restrict EMCV when overexpressed in THP-1 (Fig. 7B). As observed with HCoV-OC43 replication in THP-1 cells (Fig. 2M), EMCV replication in THP-1 cells was modestly rescued by OAS2 depletion in the absence of exogenous IFN (Fig. 7E), again likely reflecting a higher baseline expression of RLRs like MDA5 in these cells compared to A549 cells (Lama et al, 2026). Thus, the use of a different RNA virus revealed that each OAS2 isoform possesses antiviral function, but against different sets of viruses.

We next explored the determinants of p71-mediated inhibition of EMCV. As with p69 against HCoV-OC43, p71 function was ablated by N-terminal G2A mutation (Fig. 7G), confirming that p71 myristoylation–and p71’s observed Golgi localisation (Fig. 3B,C)–were also important for its antiviral function. We also tested whether RNA binding or catalytic activity were important for p71-mediated restriction of EMCV. Strikingly, disruption of the p71 catalytic centre (D481A) ablated its antiviral behaviour towards EMCV (Fig. 7H,I), in marked contrast to the same mutation in p69 when tested against HCoV-OC43 (Fig. 5B,C), suggesting a switch in mechanism between the two OAS2 isoforms. Like p69, p71 restriction required RNA binding for antiviral function (Fig. 7H,I). These data align p71 with the canonical mechanism of OAS protein function, via 2-5A synthesis and RNase L activation. Supporting this, p71 restriction of EMCV was entirely RNase L dependent (Fig. 7J,K). We also observed a specific reduction in total cellular RNA integrity in cells expressing p71 and infected with EMCV, supporting the notion that RNase L is activated following p71 engagement (Fig. 7L,M). Arguing against a role for IFN in the p71 restriction mechanism, co-expression of the interferon antagonist NPro from bovine viral diarrhoea virus, which induces IRF3 degradation and ablates IFN induction (Hilton et al, 2006) (Appendix Fig. S1A,B), had no impact on p71 function (Fig. 7N,O). Moreover, EMCV infection of A549 co-expressing ISRE-GFP and either RFP, p69 or p71, failed to induce a GFP signal (Fig. 7P). Together, these data demonstrate that OAS2 p71 restricts EMCV via the canonical RNase L-dependent pathway (Fig. 7K), indicating that the two major OAS2 isoforms operate via distinct antiviral mechanisms.

### OAS2 C-terminal tails shape antiviral specificity

**Figure 8:**
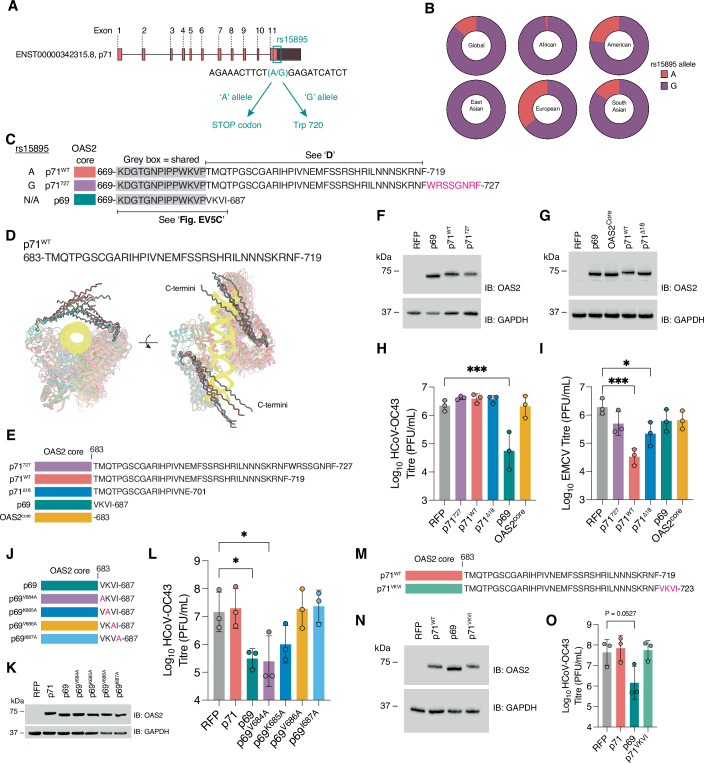
OAS2 C-terminal tails shape antiviral specificity. (**A**) Representation of the exon structure of OAS2 p71 with SNP rs15895 resulting in a premature stop codon occurring in the canonical p71 sequence (NM_016817.3). Bright colours are coding sequence, shaded region is non-coding sequence. (**B**) Frequency of the ‘A’ and ‘G’ allele of SNP rs15895 recorded for different human populations, obtained from the 1000 Genomes Project study data on the Ensembl database. (**C**) Schematic showing the C-terminal tail sequences of OAS2, including the p71 C-termini resulting from SNP rs15895. Additional C-terminal residues in the p71^727^ are highlighted in pink. Note that p71^WT^ refers to the canonical p71 isoform studied in previous figures. (**D**) Superposition of all 5 OAS2 p71 AlphaFold3 structure predictions in complex with dsRNA (Donovan et al, 2013). The highlighted tail peptides begin at residues T684 for OAS2 p71, as indicated. (**E**) Schematic of the OAS2 C-terminus of natural isoforms and OAS2 variants with tail truncations. (**F**) A549 cells were modified to express both p71 isoforms, resulting from SNP rs15895, confirmed by Western blotting. (**G**) A549 cells expressing the OAS2 C-terminal variants indicated in (**E**) were assessed by Western blot. (**H**) Infectious titre of HCoV-OC43 in A549 cells expressing OAS2 C-terminal variants (**F**, **G**), determined by plaque assay at 120 hpi. (**I**) Infectious titre of EMCV in A549 cells expressing OAS2 C-terminal variants (**F**, **G**), determined by plaque assay at 30 hpi. (**J**) Schematic of OAS2 p69 C-terminal variants generated, where each residue of the VKVI tail was individually substituted to alanine. (**K**) A549 cells expressing the OAS2 variants indicated in (**J**) were confirmed by Western blotting. (**L**) Infectious titre of HCoV-OC43 in cells expressing p69 C-terminal variants described in (**K**), determined by plaque assay at 120 hpi. (**M**) Schematic of p71^VKVI^, where the ‘VKVI’ motif of p69 has been transplanted onto the C-terminus of the canonical p71 isoform. (**N**) A549 cells were modified to express p71^VKVI^, as described in (**M**), confirmed by Western blotting. (**O**) Infectious titre of HCoV-OC43 in cells characterised in (**N**), determined by plaque assay at 120 hpi. Data information: Data is presented as the mean ± SD of three biological replicates. (**H**, **I**, **L**, **O**) Data were analysed by one-way ANOVA with Dunnett’s multiple comparison test (vs. RFP), where *p < 0.05, **p < 0.01, ***p < 0.001 and ****p < 0.0001. (**H**) ***p = 0.0004. (**I**) ***p = 0.0003, *p = 0.0281. (**L**) *p = 0.0429 (p69), *p = 0.307 (p69^V684K^). Source data are available online for this figure.

To understand their divergent antiviral profiles, we next considered the OAS2 isoform C-terminal tails–the only structural difference between p69 and p71–speculating that these must harbour determinants of restriction specificity. Interestingly, an OAS2 single-nucleotide polymorphism (SNP)–rs15895–extends the OAS2 p71 tail by eight residues. An ‘A’ allele encodes a stop codon after residue 719 (p71^WT^), whereas a ‘G’ allele encodes a tryptophan at position 720, followed by an additional seven residues (p71^727^). The original identification of the p71 isoform isolated cDNA from Daudi cells, which harbour the ‘G’ allele and encode p71^727^ rather than the p71^WT^ cloned in our study (Marié and Hovanessian, 1992) (Fig. 8A,C). Daudi cells originate from a Burkitt’s lymphoma patient of African origin, where the rs15895 ‘A’ allele occurs at a lower frequency (1.2%) than in European populations (35.6%) (Gokul et al, 2023) (Fig. 8B). In fact, globally, the ‘G’ SNP is present at the greater depth (86.4%) (Fig. 8B), suggesting the longer p71 tail has conferred a selective advantage in most locations. We speculate that European sampling bias has resulted in the ‘A’ allele, encoding the shorter p71^WT^ isoform, to become annotated as the canonical p71 sequence by both NCBI and Ensembl–and was therefore the isoform characterised in our study. While less well studied, the presence of the ‘G’ allele at rs15895–p71^727^–is associated with differential susceptibility to DENV infection and severe TBEV infection in vivo (Alagarasu et al, 2013; Barkhash et al, 2010). This suggests the precise length and/or composition of the p71 tail could influence viral replication in vivo.

**Figure EV5:**
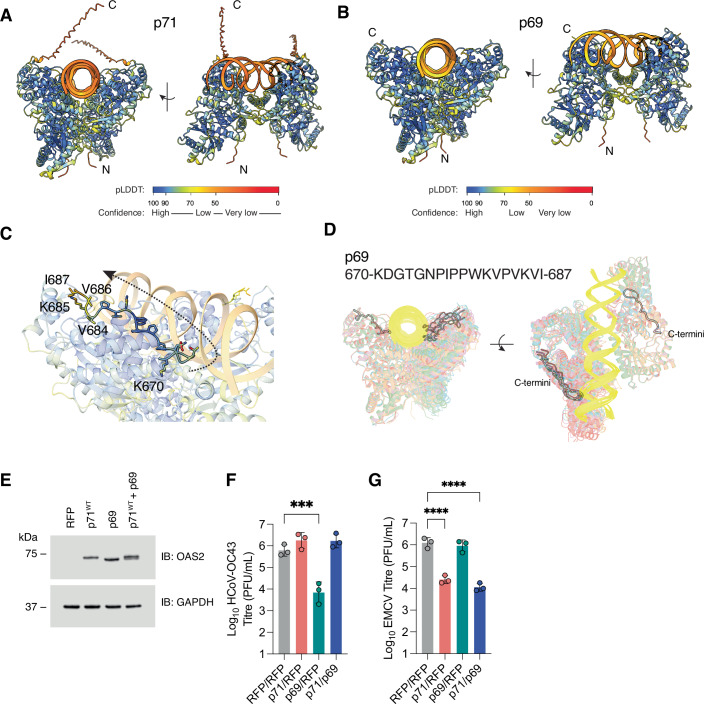
OAS2 C-terminal tails shape antiviral activity. (**A**, **B**) AlphaFold3 top-ranked models from (Fig. EV4) of p71 (**A**) or p69 (**B**) with dsRNA (Donovan et al, 2013) coloured by pLDDT score, indicated in colour key. OAS2 N- and C-termini are labelled N and C, respectively. (**C**) Close up of OAS2 p69 from structure presented in (**B**), showing side chains of peptide beginning at K670, until terminal residue I687, coloured as in (**B**). The dashed arrow indicates the sequence direction from 670-687. pLDDT scores are high between residues 670-683, and low between residues 684–687. (**D**) Superposition of all 5 OAS2 p69 AlphaFold3 structure predictions in complex with dsRNA (Donovan et al, 2013). The highlighted tail peptides begin at residues K670, as indicated; note that sequence K670-P683 is shared between p69 and p71 isoforms, thus the region highlighted for p69 is also partly present in p71. (**E**) A549 were modified to co-express both p71^WT^ and p69 isoforms. Single isoforms were co-expressed with RFP to control for transgene dosage. OAS2 expression was confirmed by Western blotting. (**F**) Infectious titre of HCoV-OC43 in cells characterised in (**E**), determined by plaque assay at 120 hpi. (**G**) Infectious titre of EMCV in cells characterised in (**E**), determined by plaque assay at 30 hpi. Data information: Data were presented as the mean ± SD. (**F**,** G**) Data were analysed by one-way ANOVA with Dunnett’s multiple comparison test (vs. RFP), where *p < 0.05, **p < 0.01, ***p < 0.001 and ****p < 0.0001. (**F**) ***p = 0.0005. (**G**) ****p < 0.0001.

To understand how OAS2 tails might affect antiviral specificity, we first compared the AF3 structural predictions of p69 and p71 in complex with the 2x36mer RNA. In contrast to most of the OAS scaffold, pLDDT scores for both isoforms’ tails were very low (Fig. EV5A,B), suggesting structural disorder. In the case of p71, the proximity of the tails to dsRNA suggests they could even contact nucleic acid (Fig. 8D). Although significantly shorter, the p69 4-residue tail was also not confidently modelled (Fig. EV5B–D). Thus, structural predictions that accurately modelled OAS2:dsRNA interfaces also suggest that the OAS2 C-terminal tails are surface exposed, disordered and sit in proximity to nucleic acid, perhaps explaining their ability to influence target specificity.

To test this, we first removed either half (p71^∆18^) or all (OAS2^core^) of the p71 tail–OAS2^core^ represents the 683 residues shared between both isoforms (Fig. 8E). We expressed these variants in A549 cells alongside the parental isoforms, p71^727^, or the RFP control (Fig. 8F,G), then infected these cells with either HCoV-OC43 or EMCV, enumerating infectious titres at 120 or 30 hpi, respectively. Remarkably, OAS2^core^ was unable to restrict either virus, revealing that the presence of a C-terminal tail is essential for all antiviral mechanisms (Fig. 8H,I). Compared to control cells, we observed a 59-fold decrease in EMCV titre in p71 cells which reduced to eightfold in p71^∆18^ cells–an “intermediate” antiviral phenotype that suggests p71-mediated restriction of EMCV is dependent on tail length (Fig. 8I). Moreover, the p71^727^ variant, abundant in African populations, lacked antiviral activity toward EMCV (Fig. 8I), indicating that natural variation in OAS2 tails can affect antiviral potency.

We were surprised by the absolute requirement for the four-residue tail of p69 in the restriction of HCoV-OC43 (Fig. 8H). To explore this, we used alanine scanning to identify any specific residue in this short motif necessary for activity (Fig. 8J,K). Surprisingly, substitution of either of the two terminal residues, V686 or I687, was sufficient to ablate p69-mediated antiviral activity against HCoV-OC43 (Fig. 8L). We also transplanted the p69 tail ‘VKVI’ motif to the end of p71 (p71^VKVI^) to ask whether, as a functional unit, it might confer HCoV-OC43 antagonism to p71^WT^ (Fig. 8M,N). However, p71^VKVI^ was unable to restrict HCoV-OC43 (Fig. 8O), indicating that VKVI context is critical for function.

The ability of OAS2 to homodimerize in cells (Fig. EV4A)(Merold et al, 2025) suggests that isoform heterodimerisation might occur should the two be co-expressed. To understand the antiviral potential of any such heterodimer, we co-expressed both isoforms stably before infecting cells with either HCoV-OC43 or EMCV. To control for increased transgene dosage, single isoforms were co-expressed with RFP, and all cell lines were compared to a double RFP/RFP-expressing control (Fig. EV5E). As previously, p71/RFP and p69/RFP cells restricted either EMCV or HCoV-OC43, respectively (Fig. EV5F,G). Interestingly, dual p71/p69-expressing cells did not display a broader restriction profile; EMCV was restricted, while HCoV-OC43 replication was unaffected (Fig. EV5F,G), mirroring the effect of p71 alone. Thus, the longer p71 tail has a dominant-negative effect on the function of p69.

## Discussion

In this study, we identify a collection of genes with antiviral activity against the seasonal coronavirus HCoV-OC43. Focusing on *OAS2*, we find that the p69 isoform is antiviral towards HCoV-OC43, while the longer p71 isoform is not. Interestingly, the antiviral activity of p69 toward HCoV-OC43 occurred independently of the canonical 2-5A/RNase L axis, aligning OAS2 with other OAS systems recently reported to act independently of RNase L (Harioudh et al, 2024; Zhu et al, 2014). In the context of HCoV-OC43, this alternative antiviral mechanism might provide immune defence in the face of the viral PDE NS2, which is an effective OAS antagonist in related betacoronaviruses like mouse hepatitis virus (Goldstein et al, 2017) and was presumed to explain the lack of restriction by OAS1 toward HCoV-OC43 (Wickenhagen et al, 2021). We also found that the p71 isoform is antiviral toward the unrelated positive-sense cardiovirus EMCV, and, as its activity was RNase L-dependent, suggests that p71 acts as a canonical OAS sensor in this role. Our data therefore suggest that a single *OAS2* locus provides antiviral breadth through alternative splicing, and that mechanistic plasticity underlies this phenomenon. The concept of ‘sensor’/’restriction factor’ duality first emerged with the discovery that TRIM5 and tetherin–already well-characterised restriction factors–also stimulated NF-κB signalling pathways upon virus detection (Galao et al, 2012; Pertel et al, 2011). Since then, several other proteins have been shown to exhibit both activities (Fletcher et al, 2015; McEwan et al, 2013), extending even to the prototypical sensors like RIG-I (Sato et al, 2015). In this context, OAS2 represents an alternative solution to achieving restriction/sensor duality, whereby the distinct functions are not coordinated but rather split across two polypeptides derived from the same gene. Both isoforms are bona fide 2-5A synthetases in vitro (Marié et al, 1999; Merold et al, 2025), suggesting RNase L independence is a functional adaptation rather than a catalytic defect. However, understanding whether restriction versus sensing are strictly split (can p69 restrict a virus via 2-5A synthesis, and p71 via RNase L-independent mechanisms?) will provide a clearer picture of how malleable the *OAS2* locus is in humans. Testing the replication of an NS2-deleted HCoV-OC43 (Diefenbacher et al, 2024) in p69- and p71-expressing cells could address whether any residual RNase L-dependent mechanism is indeed antagonised by this viral PDE.

Importantly, the C-terminal tails harbour critical determinants for virus specificity, in vitro and in vivo. Substitution of either of the final two residues from p69 was sufficient to abolish its antiviral activity toward HCoV-OC43, while deletion of its tail also rendered p71 inactive toward EMCV. This was surprising, as the expected RNA binding site, while not empirically modelled in OAS2, is predicted to lie along an exposed surface encompassing both DI and DII domains, supported by structural predictions, molecular dynamics simulations (Merold et al, 2025), and alignments with the determined OAS1-dsRNA interface (Donovan et al, 2013). A role for the C-terminal tails in viral restriction has not yet been considered, but is strongly supported by our data. Moreover, we show that an abundant OAS2 p71 snp that generates a slightly longer tail–p71^727^–also interferes with EMCV restriction, further supporting the model that tails influence target binding. Of note, the p71 C-terminal tails are characterised by periodic spacing of basic residues (and a paucity of acidic residues) (Fig. 8C), which might interact with the negatively charged phosphate backbone of dsRNA. Whether the short VKVI motif of the p69 tail influences RNA binding is less clear.

The evolution of RNase L-independent mechanisms for OAS enzymes is perhaps unsurprising considering the diversity of viral PDE enzymes, which have been recurrently acquired by both nidoviruses and rotaviruses via horizontal gene transfer from host to virus (Goldstein et al, 2024). Moreover, *OAS* genes are evolutionarily ancient antiviral loci, appearing across eukaryotic taxa–more deeply so than the related cGAS-like receptors (cGLRs) (Culbertson and Levin, 2023; Li et al, 2023). Being able to restrict viral replication by diverse enzymatic mechanisms might afford a more robust defence in the face of rapidly evolving RNA viruses, particularly where OAS-RNA binding is sequence or structure specific, as indicated by OAS1 p46 with SARS-CoV-2 (Wickenhagen et al, 2021), West Nile Virus (WNV) (Koul et al, 2020a), or adenovirus-associated virus RNA (Desai et al, 1995). As for the precise antiviral mechanism behind p69 restriction of HCoV-OC43, it seemed not to involve *IFNB1* mRNA stabilisation as reported for human OAS1 during WNV replication (Harioudh et al, 2024). Unlike its single human OAS1 orthologue, murine Oas1b is devoid of 2-5A synthetase activity (Elbahesh et al, 2011), yet remains potently antiviral towards WNV (Perelygin et al, 2002). Many mouse lab strains are highly susceptible to WNV because they encode a truncated Oas1b protein lacking the C-terminal transmembrane domain that localises Oas1b to the endoplasmic reticulum (Courtney et al, 2012; Perelygin et al, 2002). While the details of the RNase L-independent Oas1b restriction mechanism remain unclear, it is thought to involve interaction with the ATP-binding cassette protein 3, subfamily F (ABCF3) (Courtney et al, 2012). Human OASL is a catalytically inactive paralogue, but via its C-terminal ubiquitin-like domains, OASL binds to RIG-I by mimicking di-ubiquitin, enhancing RIG-I signalling (Zhu et al, 2014). Human OAS2 also localises to stress granules (Reineke and Lloyd, 2015), which can coordinate antiviral defence against diverse RNA viruses. Thus, OAS2 p69 might operate by any of these or yet to be discovered pathways against HCoV-OC43.

Irrespective of antiviral mechanism, we find that the restriction activity of both OAS2 isoforms is wholly dependent on membrane association, aligning it with the related OAS1 genes, including human OAS1 p46 (Soveg et al, 2021; Wickenhagen et al, 2021), horseshoe bat OAS1 (Lytras et al, 2023; Wickenhagen et al, 2021), and murine Oas1b (Courtney et al, 2012). Across these three examples, the method of membrane attachment is distinct, exemplifying the plasticity of the OAS system in achieving a similar goal. In the case of OAS2, N-terminal lipidation rather than C-terminal prenylation could have allowed the evolution of additional function in the C-terminal tail, as we hypothesise above. The question of precisely where and how membrane-bound OAS2 senses viral RNA remains unclear, although, whilst this manuscript was in revision, the requirement of OAS2 anchoring to the Golgi network was confirmed by Merold et al (Merold et al, 2025). Coronaviruses and cardioviruses such as EMCV have been reported to replicate in structures resembling single or double-membrane vesicles (DMV) (Knoops et al, 2008; Melia et al, 2018). Although the ER has been proposed to be the donor organelle for such structures, OAS1 similarly localises to a Golgi compartment, and can potently restrict other human coronaviruses and EMCV (Soveg et al, 2021; Wickenhagen et al, 2021). Therefore, across OAS systems, the topology of dsRNA sensing remains unclear, although at the resolution provided by our own and others’ experiments so far, OAS2 does not appear to localise to the ER. We note that vRNA can transit through exit pores (Chen et al, 2024), which could facilitate exposure to OAS enzymes anchored to the cytosolic face of the vesicle. Alternatively, anchored OASes could infiltrate the DMV during its biogenesis from the endomembrane system. It is possible that the two OAS2 isoforms exhibit subtly different distribution patterns, which better enable the detection of their respective targets, however we cannot discern this at the resolution of our imaging.

Antiviral breadth is hardwired into the IFN response. The parallel expression of multiple ISGs provides the cell with a broad palette of antiviral activities against many viral families (McDougal et al, 2023; Schoggins et al, 2011), even where some ISG products, like TRIM5 (Ganser-Pornillos and Pornillos, 2019), are highly target-specific. Some ISGs independently achieve antiviral breadth by virtue of generic restriction mechanisms; BST-2/tetherin prevents the release of enveloped viral particles belonging to diverse virus families including *Retroviridae*, *Flaviviridae*, *Filoviridae*, *Orthoherpesviridae*, *Peribunyaviridae* and *Coronaviridae* (Hagelauer et al, 2023; Neil, 2013; Varela et al, 2017), while arguably, OAS enzymes achieve this through the indiscriminate endoribonuclease RNase L. Gene duplication in theory provides another mechanism to diversify an antiviral portfolio, exemplified by the *OAS1* and *TRIM5* loci in rodents (Elkhateeb et al, 2016; Tareen et al, 2009), although this remains speculative. Here, we reveal a mechanism where, through alternative splicing and mechanistic plasticity, a single *OAS* gene expands antiviral breadth. We speculate that this might be an evolutionarily economical method for achieving functional diversity, compared to whole gene duplication. As the majority of multi-exon genes undergo alternative splicing (Kjer-Hansen and Weatheritt, 2023), this is likely to be a more common event in antiviral defence than currently appreciated.

## Methods

**Table:** Reagents and tools table

Reagent/resource	Reference or source	Identifier or catalogue number
**Experimental models**		
HEK-293T (*H. sapiens*)	Prof. Paul Bieniasz	N/A
A549 (*H. sapiens*)	Prof. Ben Hale (ATCC)	CCL-185
A549-ISRE:GFP (*H. sapiens*)	Stewart et al (2014)	N/A
A549-N^Pro^ (*H. sapiens*)	Stewart et al (2014)	N/A
Vero E6 (*C. aethiops*)	Prof. Michelle Bouloy (ATCC)	CRL-1586
Huh7.5 (*H. sapiens*)	Prof. Richard M. Elliot	N/A
THP-1 (*H. sapiens*)	Prof. Paul Bieniasz	N/A
HCoV-OC43	ATCC	VR-1558
EMCV	ATCC	VR-129B
**Recombinant DNA**		
pSCRPSY	Kane et al (2016)	N/A
pLV-EF1a-IRES-*SfiI*-Puro-TagRFP	Wickenhagen et al (2021)	N/A
pLV-EF1a-IRES-*SfiI*-Neo-TagRFP	Wickenhagen et al (2021)	N/A
lentiCRISPRv2-PuroR	Addgene	52961
lentiCRISPRv2-BlastR	Addgene	83480
pLV-*Hs*.OAS2-p71-Puro	This study	N/A
pLV-*Hs*.OAS2-p71-Neo	This study	N/A
pLV-*Hs*.OAS2-p69-Puro	This study	N/A
pLV-*Hs*.OAS2-p69-Neo	This study	N/A
pLV-*Hs*.OAS2-p71^G2A^-Puro	This study	N/A
pLV-*Hs*.OAS2-p69^G2A^-Puro	This study	N/A
pLV-*Hs*.OAS2-p71^D481A^-Puro	This study	N/A
pLV-*Hs*.OAS2-p69^D481A^-Puro	This study	N/A
pLV-*Hs*.OAS2-p69^CAFAKA^-Puro	This study	N/A
pLV-*Hs*.OAS2-p71^R529E/R533E^-Puro	This study	N/A
pLV-*Hs*.OAS2-p69^R529E/R533E^-Puro	This study	N/A
pLV-*Hs*.OAS2^core^-Puro	This study	N/A
pLV-*Hs*.OAS2-p71^△18^-Puro	This study	N/A
pLV-*Hs*.OAS2-p71^VKVI^-Puro	This study	N/A
pLV-*Hs*.OAS2-p69^V684A^-Puro	This study	N/A
pLV-*Hs*.OAS2-p69^K685A^-Puro	This study	N/A
pLV-*Hs*.OAS2-p69^V686A^-Puro	This study	N/A
pLV-*Hs*.OAS2-p69^I687A^-Puro	This study	N/A
pLV-*Hs*.OAS2-p71^727^-Puro	This study	N/A
**Antibodies**		
Anti-GAPDH, mouse monoclonal	Thermo Fisher Scientific	AM4300
Anti-vinculin, rabbit monoclonal;	Cell Signaling Technologies	13901
Anti-OAS2, rabbit polyclonal	Proteintech	19279-1-AP
Anti-OAS2, mouse monoclonal	Santa Cruz Biotechnology	sc-271117
Anti-RNase L, rabbit monoclonal	Cell Signaling Technologies	27281
Anti-HCoV-OC43 Nucleocapsid, sheep polyclonal	MRC Protein Phosphorylation and Ubiquitylation Unit	DA116
Anti-IRF3, rabbit monoclonal	Cell Signaling Technologies	4302
Anti-STAT1, rabbit polyclonal	Cell Signaling Technologies	9172
Anti-dsRNA, mouse monoclonal	Nordic-MuBio	10010500
Anti-58K, mouse monoclonal	Abcam	ab27043
Anti-calnexin, mouse monoclonal	Thermo Fisher Scientific	MA3-027
Donkey anti-sheep IgG, HRP-conjugated	Thermo Fisher Scientific	A16041
Goat anti-rabbit IgG, HRP-conjugated	Cell Signaling Technologies	7074
Horse anti-mouse IgG, HRP-conjugated	Cell Signaling Technologies	7076
Goat anti-mouse IgG2a, Alexa Fluor^TM^ 488	Thermo Fisher Scientific	A-21131
Goat anti-rabbit IgG, Alexa Fluor™ 488	Thermo Fisher Scientific	A32731
Goat anti-rabbit IgG, Alexa Fluor™ 568	Thermo Fisher Scientific	A11011
Goat anti-mouse IgG, Alexa Fluor™ 488	Thermo Fisher Scientific	A11001
Hoechst 33342	Thermo Fisher Scientific	H3570
**Oligonucleotides and other sequence-based reagents**		
DNA Primers	This study	Table S1
HCoV-OC43 qPCR Primers and Probes	This study	Table S1
OAS2 sgRNAs	This study	Table S1
RNase L sgRNAs	Wickenhagen et al (2021)	Table S1
STAT1 sgRNAs	Xu et al (2023)	Table S1
ACTB TaqMan® GE assay	Thermo Fisher Scientific	Hs01060665_g1
IFNβ TaqMan® GE assay	Thermo Fisher Scientific	Hs01077958_s1
**Chemicals, enzymes and other reagents**		
DMEM high glucose	Thermo Fisher Scientific	31966021
10X MEM	Thermo Fisher Scientific	11430030
RPMI 1640	Thermo Fisher Scientific	21875034
Phosphate-buffered saline (PBS)	Thermo Fisher Scientific	14190094
Foetal bovine serum (FBS)	Thermo Fisher Scientific	10082147
Tryspin-EDTA 0.05%	Thermo Fisher Scientific	25300054
Trypsin-EDTA 0.5%	Thermo Fisher Scientific	15400054
L-Glutamine (200 mM)	Thermo Fisher Scientific	25030024
Sodium Bicarbonate 7.5%	Thermo Fisher Scientific	25080094
Gentamicin	Melford Laboratories	G0124
Penicillin/Streptomycin	Thermo Fisher Scientific	15140122
Puromycin	Melford Laboratories	P330020
Blasticidin S	Melford Laboratories	B12150
G418	Melford Laboratories	G64000
Phorbol 12-myristate 13-acetate (PMA)	Invivogen	tlrl-pma
Linear polyethylenimine (PEI), 25 kDa	Polysciences	23966
Bovine Serum Albumin	Sigma-Aldrich	A7638
Avicel RC-591	FMC Biopolymer	N/A
TRIzol Reagent	Thermo Fisher Scientific	15596026
Ruxolitinib	Stratech Scientific	S1378
Human interferon beta 1b	Stratech Scientific	11415-1
Human interferon alpha H2	Stratech Scientific	11145-1
Poly(I:C) HMW	Invivogen	tlrl-pic
Poly(I:C) HMW Biotin	Invivogen	tlrl-picb
Pierce^TM^ Streptavidin Magnetic beads	Thermo Fisher Scientific	88817
Herculase II Polymerase	Agilent Technologies	600675
NEB® 5-alpha competent *E. coli*	New England Biolabs	C2987H
FastDigest DpnI	Thermo Fisher Scientific	FD1703
SfiI	New England Biolabs	R0123S
HindIII-HF®	New England Biolabs	R3104S
4X Bolt™ LDS Sample Buffer	Thermo Fisher Scientific	B0007
NuPAGE™ Bis-Tris Gel, 4–12%, 10-well	Thermo Fisher Scientific	NP0322BOX
iBlot^TM^ Nitrocellulose Transfer Stacks	Thermo Fisher Scientific	IB23002
Pierce™ ECL Western Blotting Substrate	Thermo Fisher Scientific	32209
SuperScript IV Reverse Transcriptase	Thermo Fisher Scientific	18090010
Random Hexamers	Thermo Fisher Scientific	N8080127
TaqMan Fast Universal Master Mix	Thermo Fisher Scientific	4352042
**Software**		
AlphaFold 3	www.alphafoldserver.com	
UCSF ChimeraX v1.8	Meng et al (2023)	
Clustal Omega	Madeira et al (2024)	
FlowJo v10.8	https://www.flowjo.com/	
GraphPad Prism v10	www.graphpad.com	
BioRender	https://app.biorender.com/	
Adobe Illustrator	https://adobe.com/products/illustrator	
**Other**		
CytoTox-Glo™ Cytotoxicity assay	Promega	G9290
PhenoPlate 96-well	Revvity	6055302
Celigo^TM^ Imaging Cytometer	Revvity	200-BFFL-5C
Guava EasyCyte Flow Cytometer	Millipore	
iBlot^TM^ 2 Gel Transfer Device	Thermo Fisher Scientific	IB21001
Odyssey® XF scanner	LICOR	
SRX-101A Medical Film Processor	Konica Minolta	
QuantStudio^TM^ 3 Real-Time PCR system	Thermo Fisher Scientific	A28567
4200 Tapestation	Agilent Technologies	G2991BA
High Sensitivity RNA Screentape	Agilent Technologies	5067-5579
Zeiss LSM 880 with Airyscan	ZEISS	

### Cells and viruses

HEK-293T, A549, Vero E6 and Huh7.5 cells were propagated in Dulbecco’s Modified Eagle Medium (DMEM; Thermo Fisher Scientific) supplemented with 10% foetal bovine serum (FBS; Thermo Fisher Scientific) and 10 μg/mL gentamicin (Melford Laboratories) or 100 U/mL penicillin/100 μg/mL streptomycin (Thermo Fisher Scientific). A549-NPro and A549-ISRE:GFP cells were kind gifts from R.E. Randall (Stewart et al, 2014). THP-1 cells were propagated in RPMI 1640 Medium (Thermo Fisher Scientific), supplemented with 10% FBS and 100 U/mL penicillin/100 μg/mL streptomycin. Betacoronavirus HCoV-OC43 (VR-1558) was purchased from ATCC and propagated on Vero E6 cells (Fig. 1) or Huh7.5 cells (all other figures) at 33 °C. Encephalomyocarditis virus (VR-129B) was purchased from ATCC and propagated on Vero E6 cells at 37 °C.

### Retroviral vectors and plasmids

The screening lentiviral vector pSCRPSY (GenBank accession no. KT368137.1) and the modified plasmids pLV-EF1a-IRES-*SfiI*-Puro-TagRFP and pLV-EF1a-IRES-*SfiI*-Neo-TagRFP have been described previously (Wickenhagen et al, 2021). cDNA for OAS2 p71 (NM_016817.3) and OAS2 p69 (NM_002535.3) were ordered as gene blocks (IDT DNA) with flanking *SfiI* sites, and subcloned into the pLV plasmids; pLV-EF1a-IRES-*SfiI*-Neo was used for the dual isoform restriction and STAT1 KO experiments. Primer sequences for the following mutagenesis are found in (Appendix Table S1). The OAS2-p71^G2A^ and OAS2-p69^G2A^ variants were generated by PCR amplifying the relevant gene blocks with primers that mutate Gly2 to Ala. OAS2-p71^∆18^ and OAS2^core^ constructs were generated by PCR amplification from OAS2-p71 templates, then introduced into pLV-EF1a-IRES-SfiI-Puro using Gibson Assembly (NEB), following the manufacturer’s protocols. OAS2-p69^V684A^, OAS2-p69^K685A^, OAS2-p69^V686A^ and OAS2-p69^I687A^ were similarly generated by PCR amplification from OAS2-p69 templates.

The OAS2^D481A^, OAS2^CAFAKA^, OAS2^R529E/R533E^ and OAS2-p71^VKVI^ mutants were generated by in vivo assembly (IVA) using overlapping primers. IVA reactions were performed using 1 ng template, 100 nM primers, 200 μM dNTPs, 3% DMSO, 0.5 μL Herculase II Fusion DNA polymerase (Agilent Technologies). Cycle parameters were 95 °C 30 s, 18 cycles of 95 °C 10 s, 60 °C 30 s and 72 °C 3 min. A final hold of 72 °C for 10 min was performed. 1 μL FastDigest *DpnI* (Thermo Fisher Scientific) was added and incubated at 37 °C for 15 min. 1 μL reaction was added to 50 μL NEB® 5-alpha cells (NEB C2987H), incubated on ice 15 min, heat shocked at 42 °C 30 s, incubated on ice for 2 min, then 200 μL SOC media (NEB) added and cells recovered at 37 °C, 200 rpm, for 45 min. The complete mixture was plated on LB agar containing 200 μg/mL ampicillin. Colonies were screened by analytical restriction enzyme digest with *HindIII* (NEB), followed by Sanger sequencing.

LentiCRISPRv2-Puro and lentiCRISPRv2-Blast plasmids were used to knock down the expression of OAS2 and RNase L, respectively (Sanjana et al, 2014). CRISPR guides were designed using the CHOPCHOP tool (https://chopchop.cbu.uib.no) (Labun et al, 2019). Seven guides were subcloned into the lentiCRISPRv2 system, and the three best guides that depleted target protein expression were used for subsequent experiments (Appendix Table S1).

Retroviral vectors used for cell modification were produced by transient transfection in HEK-293T cells using the vector plasmid, HIV-1 gag-pol (pNLGP) and vesicular stomatitis virus glycoprotein expression plasmid (pVSVg), as described previously (Rihn et al, 2019). A549 cells were transduced with vector-containing supernatants filtered using a 0.45 μm-pore-size filter. Transduced cells were selected with 2 μg/mL puromycin, 5 μg/mL blasticidin or 2 mg/mL G418 (Melford Laboratories).

### Arrayed ISG expression screening

ISG lentiviral libraries encoding human, macaque and bovine genes have been described previously (Hardy et al, 2023; Wickenhagen et al, 2021). Briefly, libraries were generated in HEK-293T seeded in 96-well plates at a density of 3.5 × 10^4^ cells/well. HEK-293T were transfected with pSCRPSY, pNLGP and pVSVg at a ratio of 125 ng:25 ng:5 ng in the presence of Polyethylenimine (PEI; Polysciences). Supernatant was collected 48, 72 and 96 h post-transfection and replaced with fresh medium.

To perform the multi-species library screen, A549 cells were seeded in 96-well PhenoPlates (Revvity) at a density of 5 × 10^3^ cells/well and grown overnight. Cells were transduced with 50 μL of the ISG lentiviral libraries (one ISG per well) and spinoculated at 500×*g* for 1 h. Forty-eight hours post-transduction, cells were infected with HCoV-OC43 at MOI 0.07, in DMEM supplemented with 2% FBS. After incubation at 33 °C for 72 h, cells were fixed with a final concentration of 2% formaldehyde for 30 min, washed with PBS, and then permeabilised with 0.2% Triton X-100 (Sigma-Aldrich) for 5 min. After cells were washed and blocked with 1% Bovine Serum Albumin (BSA; Sigma-Aldrich) for 1 h, cells were incubated with 1 μg/mL of mouse anti-dsRNA IgG2a (Nordic-MuBio) overnight at 4 °C. After repeating washing and blocking steps, cells were incubated with 2 μg/mL goat anti-mouse IgG2a AlexaFluor488 (Thermo Fisher Scientific) and 5 μg/mL Hoechst 33342 (Thermo Fisher Scientific) for 1 h. Screening plates were scanned and analysed using the Celigo Imaging Cytometer (Nexcelom Biosciences). Transduced and infected cells were gated using FlowJo v10.8. Data were normalised to the mean of each species library, and z-scores for each gene were calculated. ISGs with <10% transduction efficiency were excluded from hit selection.

Miniscreen libraries were generated as described above. To measure cytotoxicity and ISRE induction, A549-ISRE:GFP cells were transduced with 50 μL miniscreen library supernatant and spinoculated for 1 h at 500×g. Supernatant was collected 120 h post-transduction and tested with the CytoTox-Glo™ Cytotoxicity assay (Promega), following the manufacturer’s protocols. Cells were disassociated using 0.5% trypsin-EDTA (Thermo Fisher Scientific) before fixation with formaldehyde. GFP+ cells were measured using the Guava EasyCyte flow cytometer (MilliPore); 10000 events/well were acquired, and data were analysed using FlowJo v10.8. Data was normalised to SCRPSY-EMPTY controls.

### Virus infections

For HCoV-OC43 plaque assays, transduced A549 cells were seeded at 4 × 10^5^ cells/well in a 12-well plate and grown to confluency overnight. Cells were inoculated with 250 μL HCoV-OC43 serially diluted tenfold in serum-free DMEM and incubated at 33 °C for 1 h. An overlay of 1:1 Avicel (1.2%, FMC) and 2x MEM (Thermo Fisher Scientific), supplemented with L-glutamine, sodium bicarbonate 7.5% (Thermo Fisher Scientific), gentamicin and 4% FBS was then added. Cells were incubated for 5 days, fixed with 4% Formaldehyde solution, washed twice with PBS (Thermo Fisher Scientific), and stained with Coomassie Blue for plaque visualisation. The same conditions were used for EMCV plaque assays but were incubated for 30 h at 37 °C.

For infections for RT-qPCR and Western blot analysis, MOIs were calculated using virus titre (PFU/mL) determined by plaque assay on A549 cells. Unless otherwise stated, transduced A549 cells were infected at an MOI of 0.01 and incubated for 72 h prior to cell collection. Concentrations and incubations with human interferon beta 1b (IFNβ), alpha H2 (IFNɑ14) and Ruxolitinib (RUX; Stratech Scientific) are described in the figure legends.

For growth curve experiments, transduced cells were seeded at 4 × 10^5^ (A549) or 8 × 10^5^ (THP-1) cells/well in a 12-well plate; THP-1 cells were differentiated into macrophages using 100 ng/mL phorbol 12-myristate 13-acetate (PMA, Invivogen), with the media changed 24 h post-PMA addition. For KO experiments, cells were pre-treated with 100 U/mL IFNβ 24 h (A549) or 120 h (THP-1) post-seeding. The following day, cell supernatant was replaced with fresh media containing HCoV-OC43 (MOI 0.01) or EMCV (MOI 0.005) and incubated at 33 or 37 °C, respectively. About 100 μL supernatant was harvested at specific timepoints—HCoV-OC43 (24 h, 48 h and 72 hpi) and EMCV (8 h, 24 h, and 48 hpi)—stored at −70 °C and replaced with 100 μL fresh media. Infectious titre was calculated by plaque assay, as described above, on A549 cells.

For ISRE-GFP experiments, transduced cells were seeded at 2 × 10^4^ cells/well in a 96-well plate. The following day, cells were treated with 100 U/mL IFNβ, or infected with HCoV-OC43 (MOI 0.1) or EMCV (MOI 0.01) and incubated at 37 °C, except HCoV-OC43-infected cells, which were incubated at 33 °C. At specified timepoints post-infection, cells were disassociated and fixed with formaldehyde, as described for the ISRE induction miniscreens.

### Western blot analyses

Cells were lysed on ice in a 1% NP-40 buffer containing protease and phosphatase inhibitors. After centrifugation, NuPAGE™ LDS Sample buffer containing 2-Mercaptoethanol (βME) was added. Proteins were separated on NuPAGE^TM^ 4–12% Bis-Tris polyacrylamide gels and transferred onto nitrocellulose membranes using the iBlot 2 (Thermo Fisher Scientific). After blocking in 5% milk, membranes were probed against GAPDH (Thermo Fisher), vinculin (Cell Signalling Technologies), OAS2 (Proteintech), RNase L (Cell Signalling Technologies), IRF3 (Cell Signalling Technologies), STAT1 (Cell Signalling Technologies) or HCoV-OC43 nucleocapsid (MRC Protein Phosphorylation and Ubiquitylation Unit). Membranes were then stained with species IgG-specific secondary antibodies conjugated to horseradish peroxidase: donkey anti-sheep (Thermo Fisher Scientific), goat anti-rabbit (Cell Signalling Technologies) and horse anti-mouse (Cell Signalling Technologies). After the addition of Pierce ECL substrate (Thermo Fisher Scientific), membranes were scanned using the LICOR Odyssey XF scanner or on the SRX-101A Medical Film Processor (Konica Minolta).

### Protein crosslinking

Cells were treated with 0.5% formaldehyde or not, for 10 min at room temperature. Reactions were quenched with glycine (0.25 M) for 5 min. Cells were lysed in RIPA buffer on ice for 15 min, and sonicated (6 cycles × 30 s). Lysates were normalised and collected for Western blot analysis.

### Biotin-poly(I:C) pulldown

Cells were lysed in ice-cold 1% NP-40 buffer containing protease and phosphatase inhibitors for 20 min. Clarified lysates were normalised, and input samples were collected. Lysates were incubated with biotin-poly(I:C) (Invivogen) for 1 h at 4 °C. Streptavidin magnetic beads (Thermo Fisher Scientific) were added, and samples were incubated for 1 h at 4 °C. Beads were washed four times with 1% NP-40 buffer containing protease and phosphatase inhibitors, resuspended in LDS buffer with βME and boiled at 90 °C for 5 min prior to Western blot analysis.

### RT-qPCR

Infected cells were lysed with TRIzol (Thermo Fisher Scientific), and the aqueous RNA layer was collected following chloroform precipitation. RNA was purified using the RNeasy mini kit (Qiagen), with on-column DNase treatment (Qiagen), following the manufacturer’s instructions. cDNA was synthesised using SuperScript IV with random hexamer primers (Thermo Fisher Scientific). Host and viral gene expression was measured using TaqMan Fast Universal Master Mix (Applied BioSystems) and specific TaqMan probes on the QuantStudio 3 Real-Time PCR machine (Thermo Fisher Scientific). Using the 2^−△△Ct^ method, viral transcript levels were normalised to ACTB (Hs01060665_g1, Thermo Fisher Scientific) and then normalised to input viral transcripts at 2 h in the respective cell line or 48/72 h in the control cell line. Primers and probes for viral transcripts can be found in (Appendix Table S1). For poly(I:C) stimulation experiments, IFNβ transcript levels (Hs01077958_s1, Thermo Fisher Scientific) were normalised to ACTB, then normalised to the 0 h timepoint of the respective cell line.

### RNA integrity assay

A549-RFP, A549-OAS2-p71 and OAS2-p69 cells were seeded at 4 × 10^5^ cells/well in 12-well plates. The following day, cells were mock-treated, stimulated with 1 μg/mL poly(I:C) (Invivogen) or infected with EMCV (MOI 2). Nucleic acids, extracted as described above, were quantified using a Qubit 4 fluorometer with High Sensitivity RNA and High Sensitivity dsDNA reagents (Thermo Fisher Scientific) to determine RNA and DNA concentrations in ng/µL. Measured RNA concentrations were used to normalise sample inputs for sizing and quality scoring using a Tapestation 4200 system with High Sensitivity RNA Screentape and Reagents (Agilent Technologies). Quality scores were recorded in the form of RIN and DV_200_ scores generated by the Agilent Tapestation Analysis software. These scores infer levels of RNA degradation based on relative proportions of 18S and 28S ribosomal RNA, and on the proportion of all material which falls within a range of 200–10,000 bp, respectively.

### Immunofluorescence

A549 parental or transduced cells were seeded at a density of 5 × 10^4^ cells/well onto glass coverslips. Cells were pre-treated with 1000 U/mL IFNβ or infected with HCoV-OC43 at MOI 10 for 24 h. After washing with PBS and fixing with 4% formaldehyde for 15 min at room temperature, cells were permeabilised in buffer containing 5% BSA and 0.2% Triton X-100 in PBS for 20 min at room temperature. The following antibodies, diluted in permeabilisation buffer, were used: rabbit polyclonal anti-OAS2 (Proteintech), mouse monoclonal anti-dsRNA (Nordic-MuBio), mouse monoclonal anti-58K (abcam), mouse monoclonal anti-calnexin, goat anti-mouse IgG2a Alexa Fluor™ 488, goat anti-rabbit IgG (H + L) Alexa Fluor™ 568, goat anti-rabbit IgG (H + L) Alexa Fluor™ 568, goat anti-mouse IgG (H + L) AlexaFluor™ 488 (Thermo Fisher Scientific). Coverslips were incubated for 1 h with primary antibody, followed by secondary antibody and Hoechst for 1 h. Maximum intensity projection images of cell monolayers were acquired with an Airyscan Fast detector fitted to a Zeiss LSM 880 confocal microscope. Acquired images were analysed using ZEN software.

### Software

Image and flow cytometry data were gated using FlowJo v10.8 (https://www.flowjo.com/). OAS2-dsRNA complex structural predictions were generated using the AlphaFold3 public server (www.alphafoldserver.com) (Abramson et al, 2024). Structural analyses and molecular graphics were performed with UCSF ChimeraX (version 1.8) (Meng et al, 2023). Protein sequences were analysed using Clustal Omega (Madeira et al, 2024) and AliView (Larsson, 2014). OAS2 sequence orthologs were obtained from the NCBI orthologs server. Graphs were generated using GraphPad Prism v10 (www.graphpad.com). Figures were prepared in Adobe Illustrator 2025. The schematic illustration in Fig. EV1 was generated using BioRender software (https://app.biorender.com/).

## Supplementary information

Peer Review File

Source data Fig. 1

Source data Fig. 2

Source data Fig. 3

Source data Fig. 4

Source data Fig. 5

Source data Fig. 6

Source data Fig. 7

Source data Fig. 8

Expanded View Source Data

Appendix

Expanded View Figures

## Data Availability

All source data were provided. Any additional information required to analyse data reported in this paper is available from Adam Fletcher upon request. The source data of this paper are collected in the following database record: biostudies:S-SCDT-10_1038-S44318-026-00825-w.
