# Supplementary figures and images for: Alternative splicing broadens antiviral diversity at the human OAS2 locus
Source: EMBO J. 2026 Jun 3;45(14):5164–91. doi: 10.1038/s44318-026-00825-w (PMC13372824; doi:10.1038/s44318-026-00825-w)

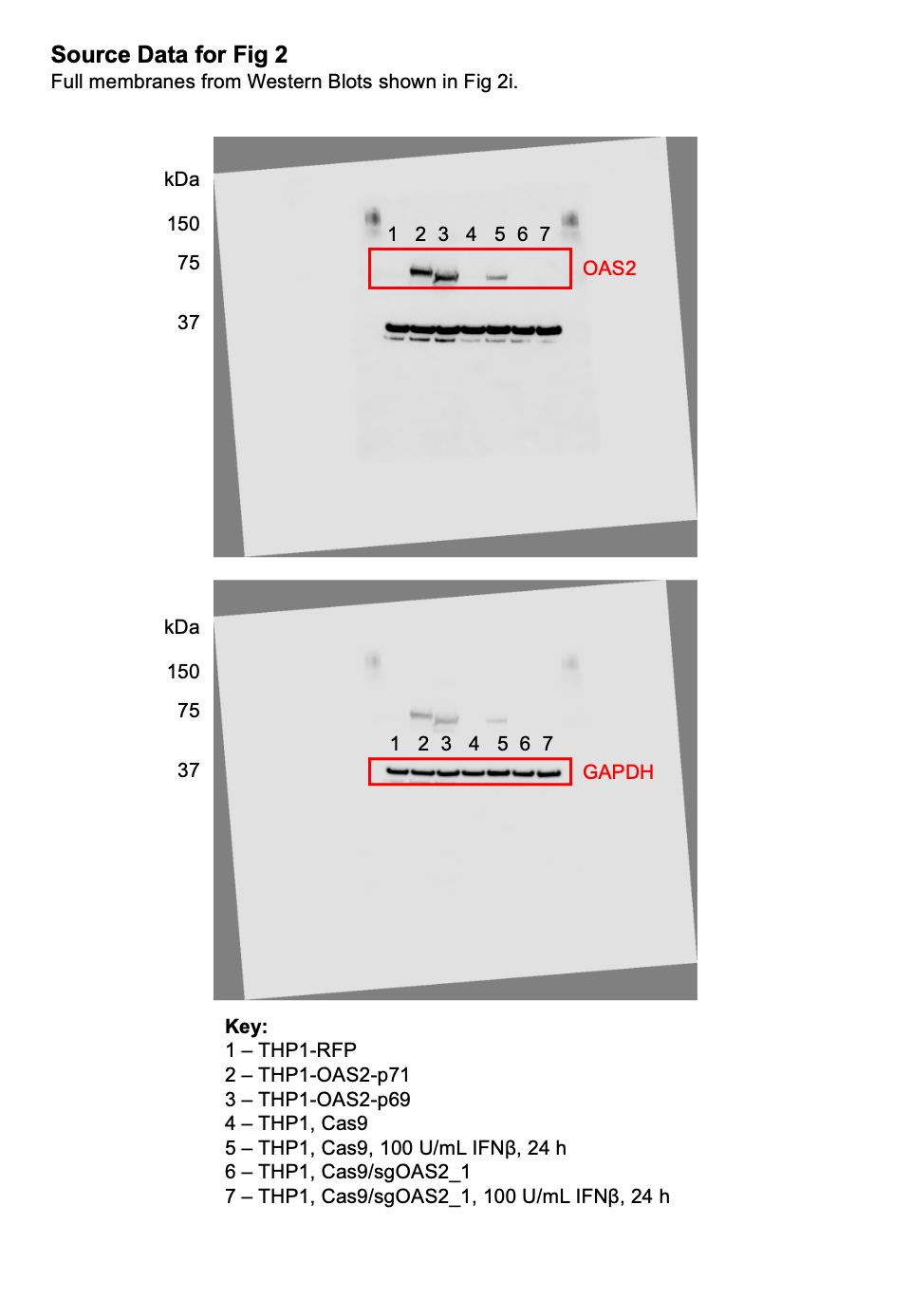

Supplement: Supplementary file 3 — Source data Fig. 2 [file 44318_2026_825_MOESM3_ESM.zip › Figure 2/2I/Figure_2I_WesternBlot.tiff]

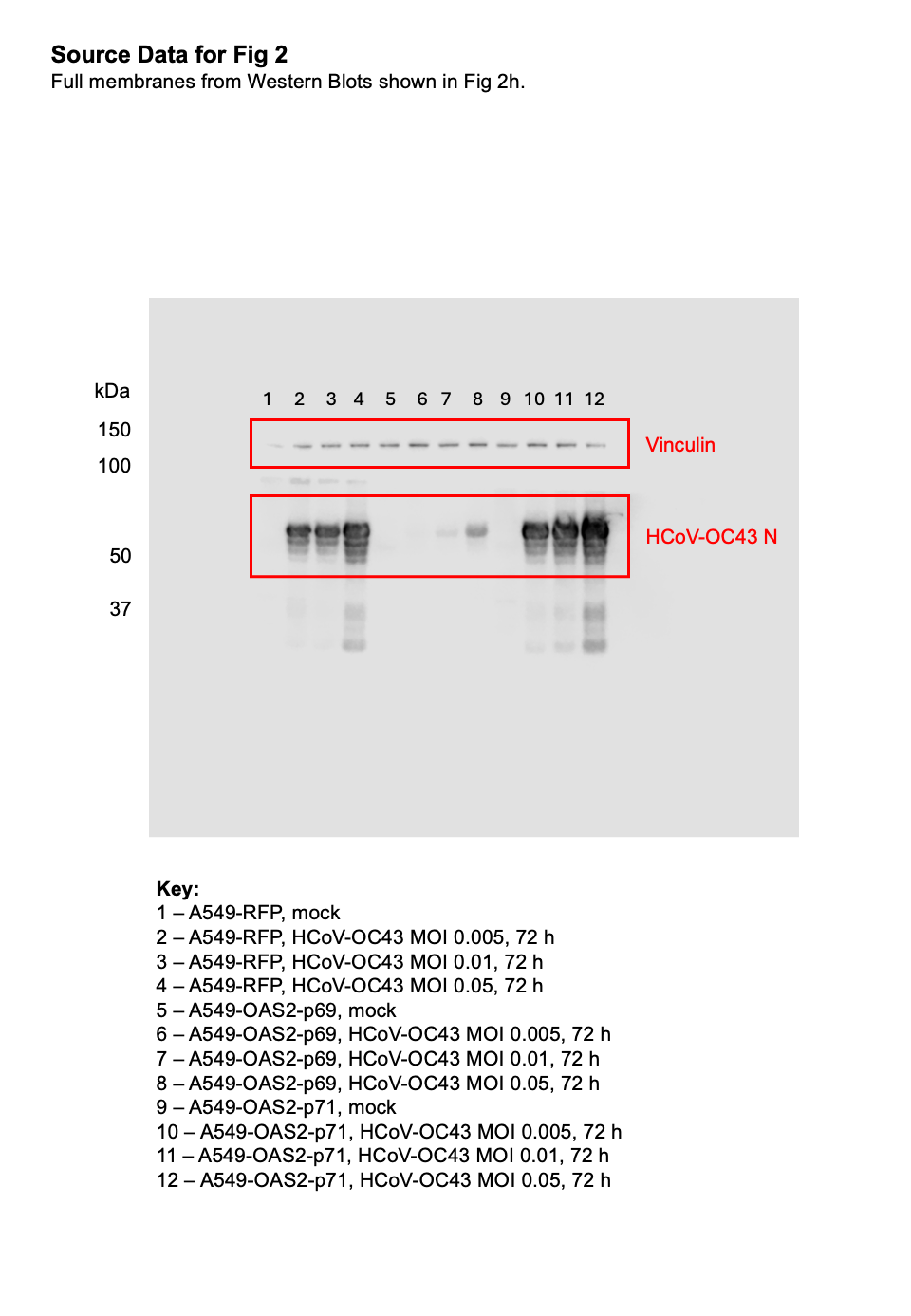

Supplement: Supplementary file 3 — Source data Fig. 2 [file 44318_2026_825_MOESM3_ESM.zip › Figure 2/2H/Figure_2H_WesternBlot.tiff]

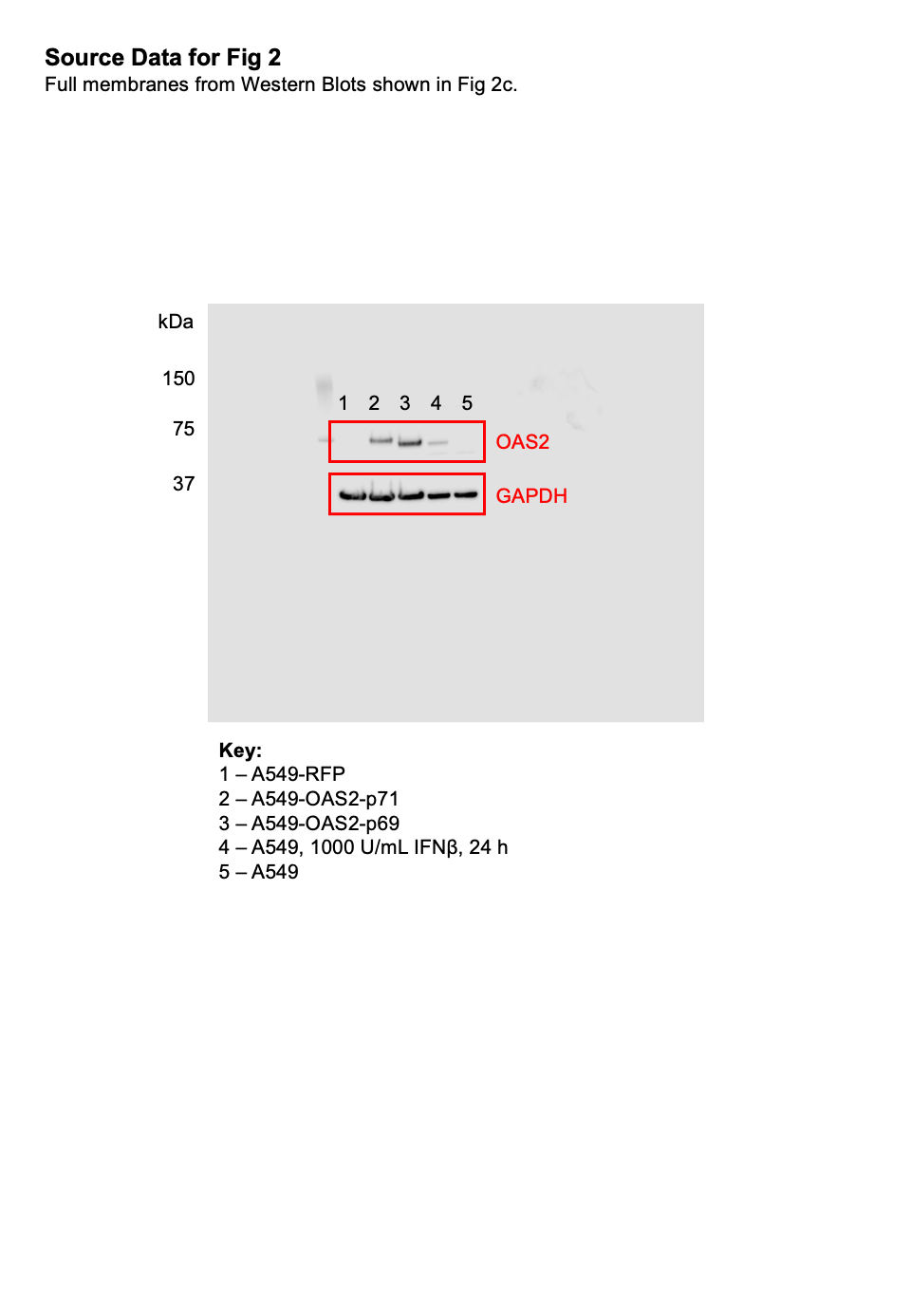

Supplement: Supplementary file 3 — Source data Fig. 2 [file 44318_2026_825_MOESM3_ESM.zip › Figure 2/2C/Figure_2C_WesternBlot.tiff]

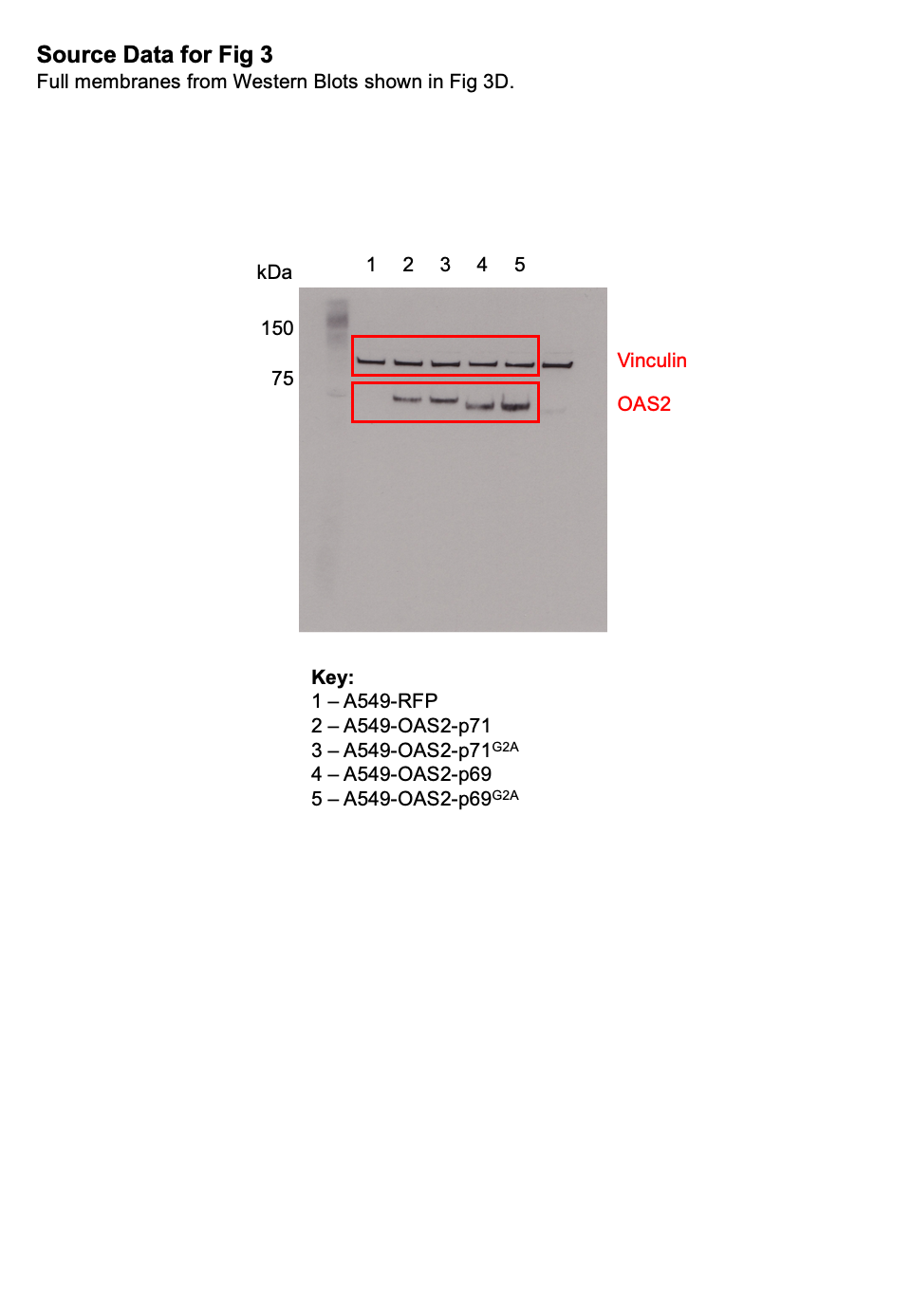

Supplement: Supplementary file 4 — Source data Fig. 3 [file 44318_2026_825_MOESM4_ESM.zip › Figure 3/3D/Figure_3D_WesternBlot.tiff]

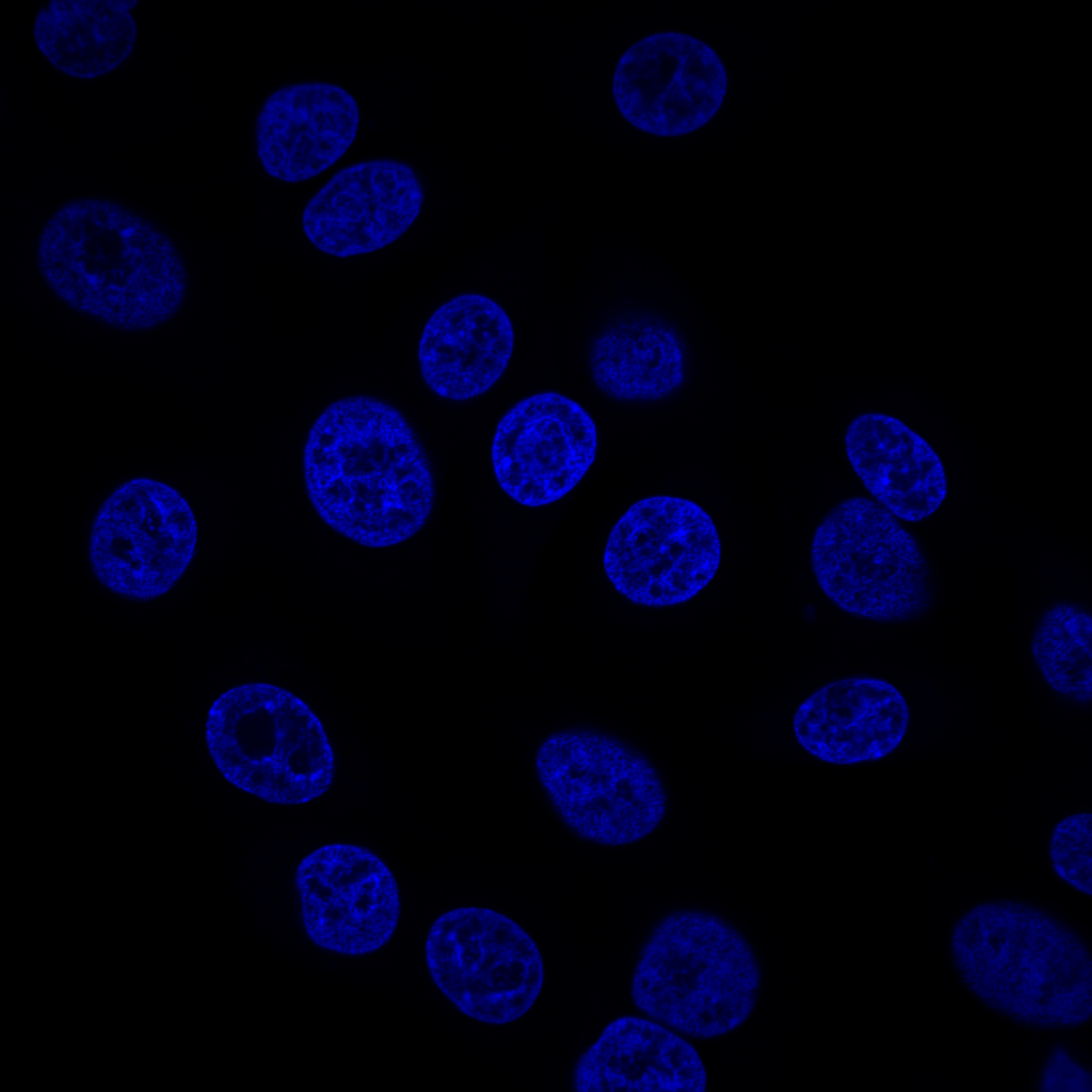

Supplement: Supplementary file 4 — Source data Fig. 3 [file 44318_2026_825_MOESM4_ESM.zip › Figure 3/3B/Figure_3B_IF/A549_IFN/A549_IFN_DNA_blue.tif]

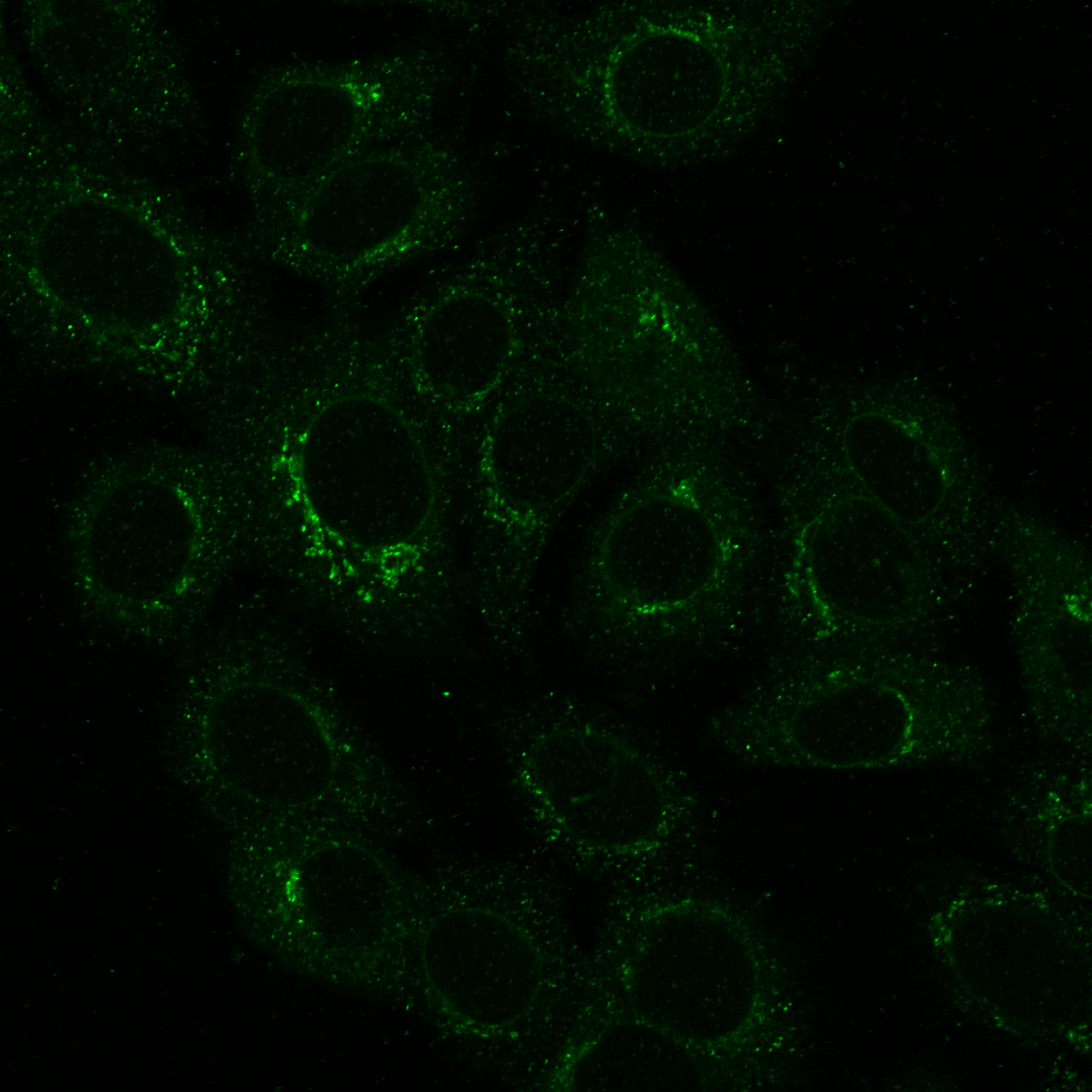

Supplement: Supplementary file 4 — Source data Fig. 3 [file 44318_2026_825_MOESM4_ESM.zip › Figure 3/3B/Figure_3B_IF/A549_IFN/A549_IFN_58K_green.tif]

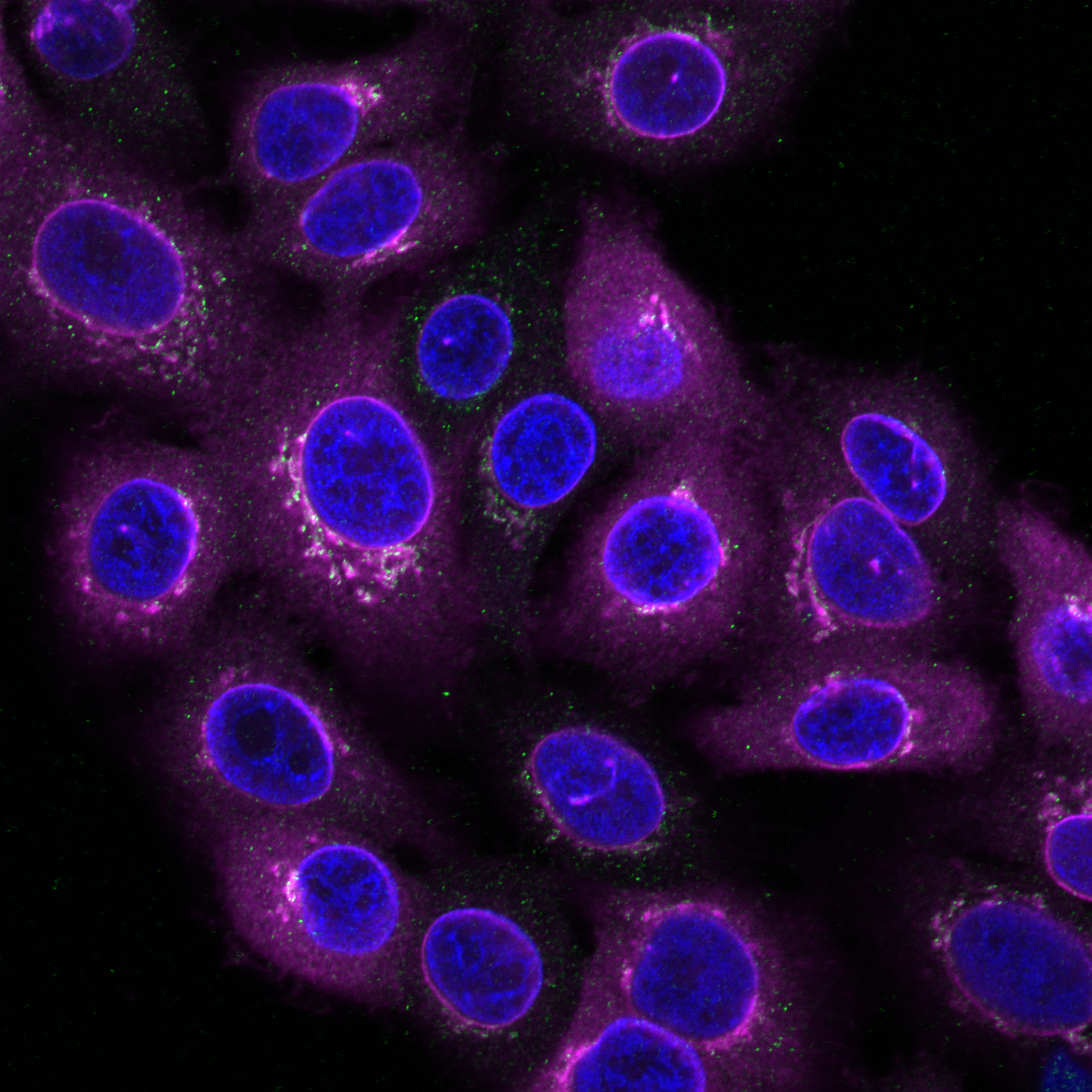

Supplement: Supplementary file 4 — Source data Fig. 3 [file 44318_2026_825_MOESM4_ESM.zip › Figure 3/3B/Figure_3B_IF/A549_IFN/A549_IFN_merge.tif]

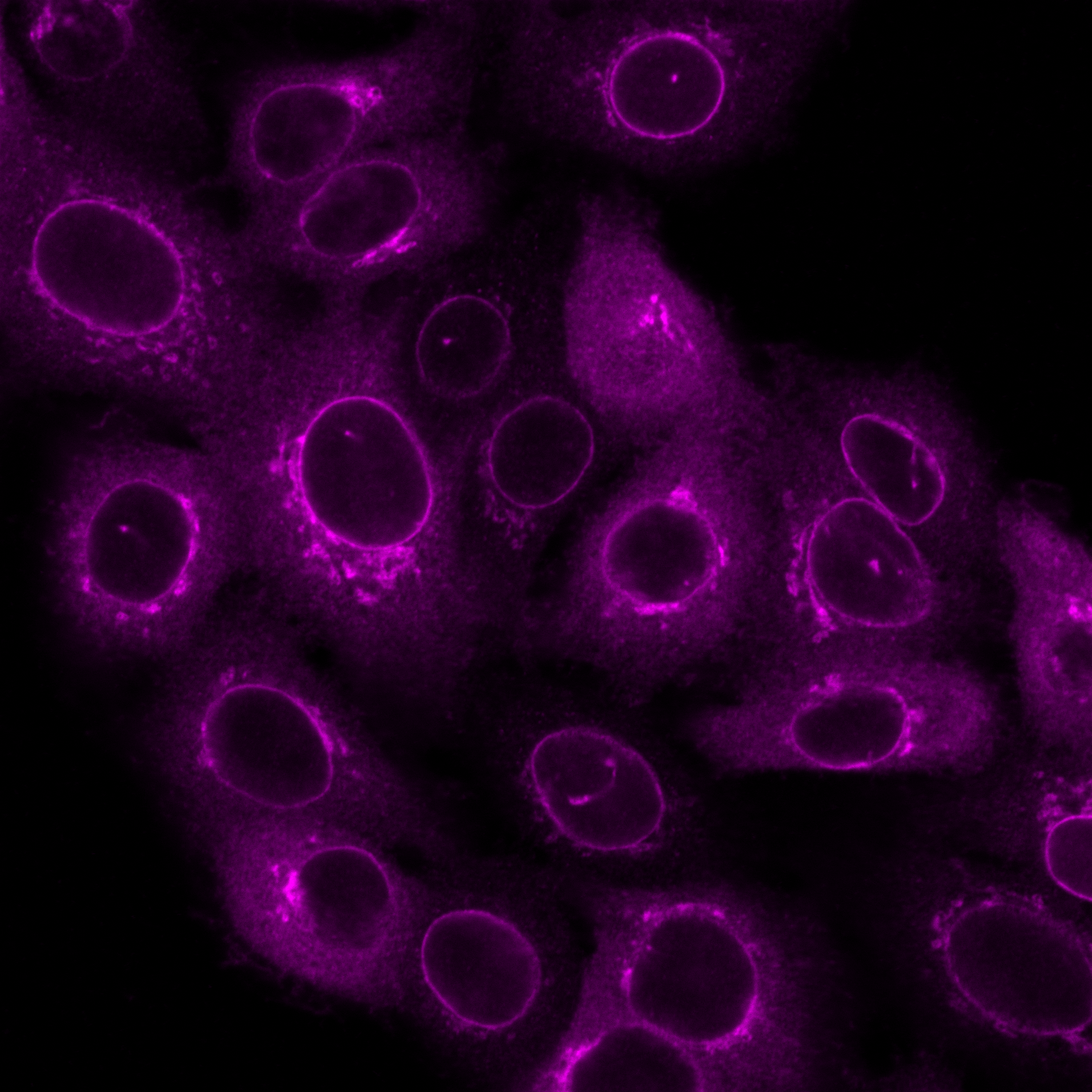

Supplement: Supplementary file 4 — Source data Fig. 3 [file 44318_2026_825_MOESM4_ESM.zip › Figure 3/3B/Figure_3B_IF/A549_IFN/A549_IFN_OAS2_Magenta.tif]

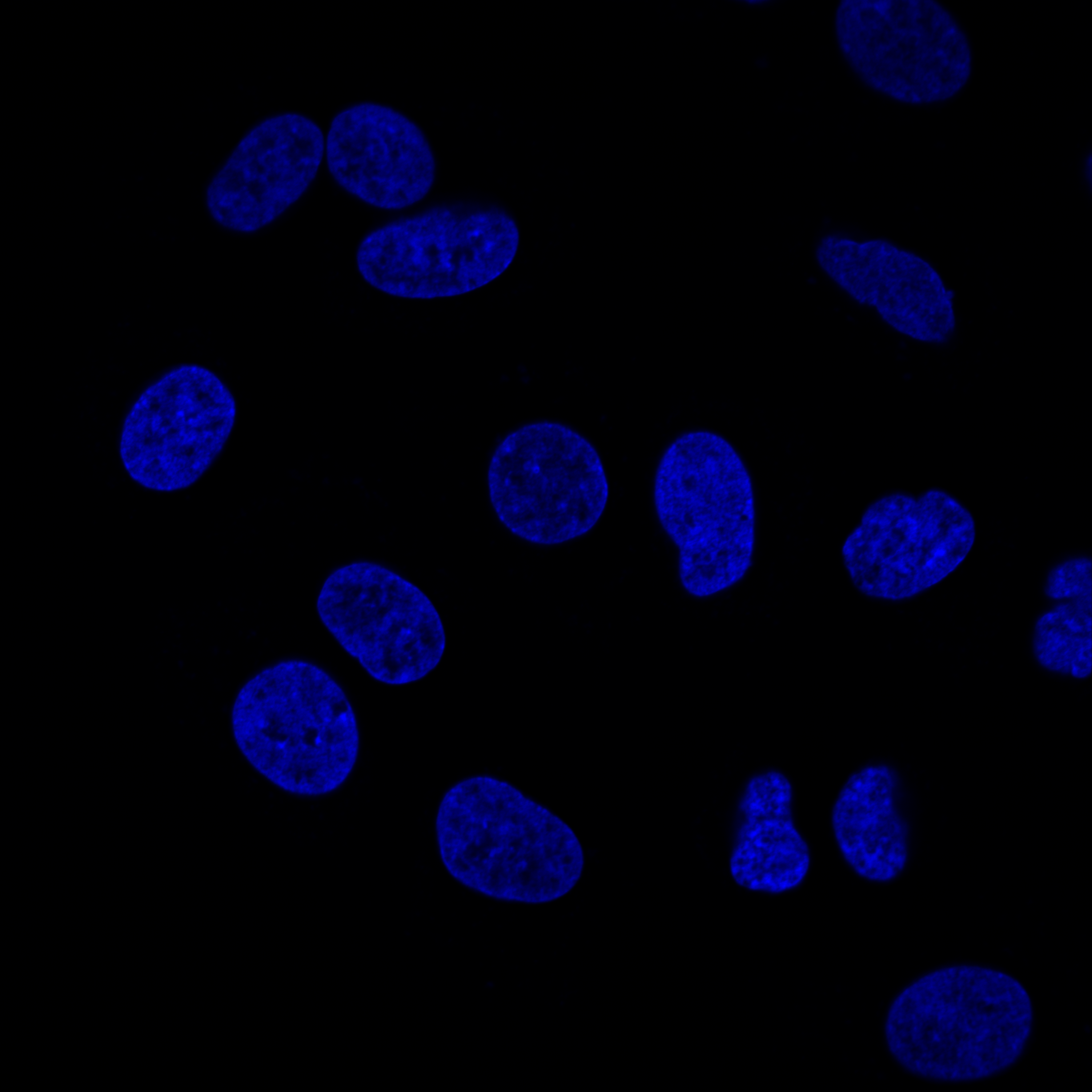

Supplement: Supplementary file 4 — Source data Fig. 3 [file 44318_2026_825_MOESM4_ESM.zip › Figure 3/3B/Figure_3B_IF/A549_p69/A549_p69_DNA_blue.tif]

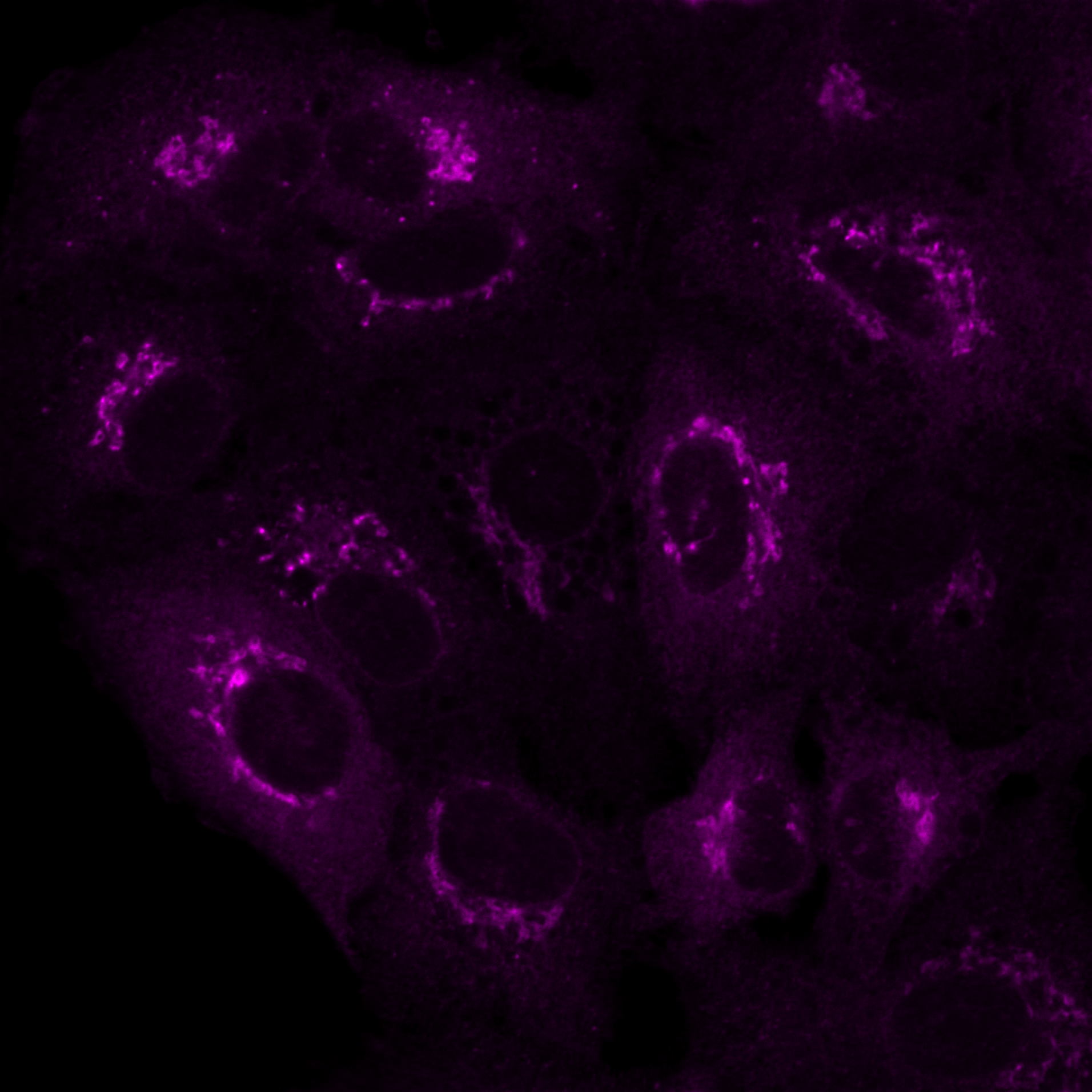

Supplement: Supplementary file 4 — Source data Fig. 3 [file 44318_2026_825_MOESM4_ESM.zip › Figure 3/3B/Figure_3B_IF/A549_p69/A549_p69_OAS2_magenta.tif]

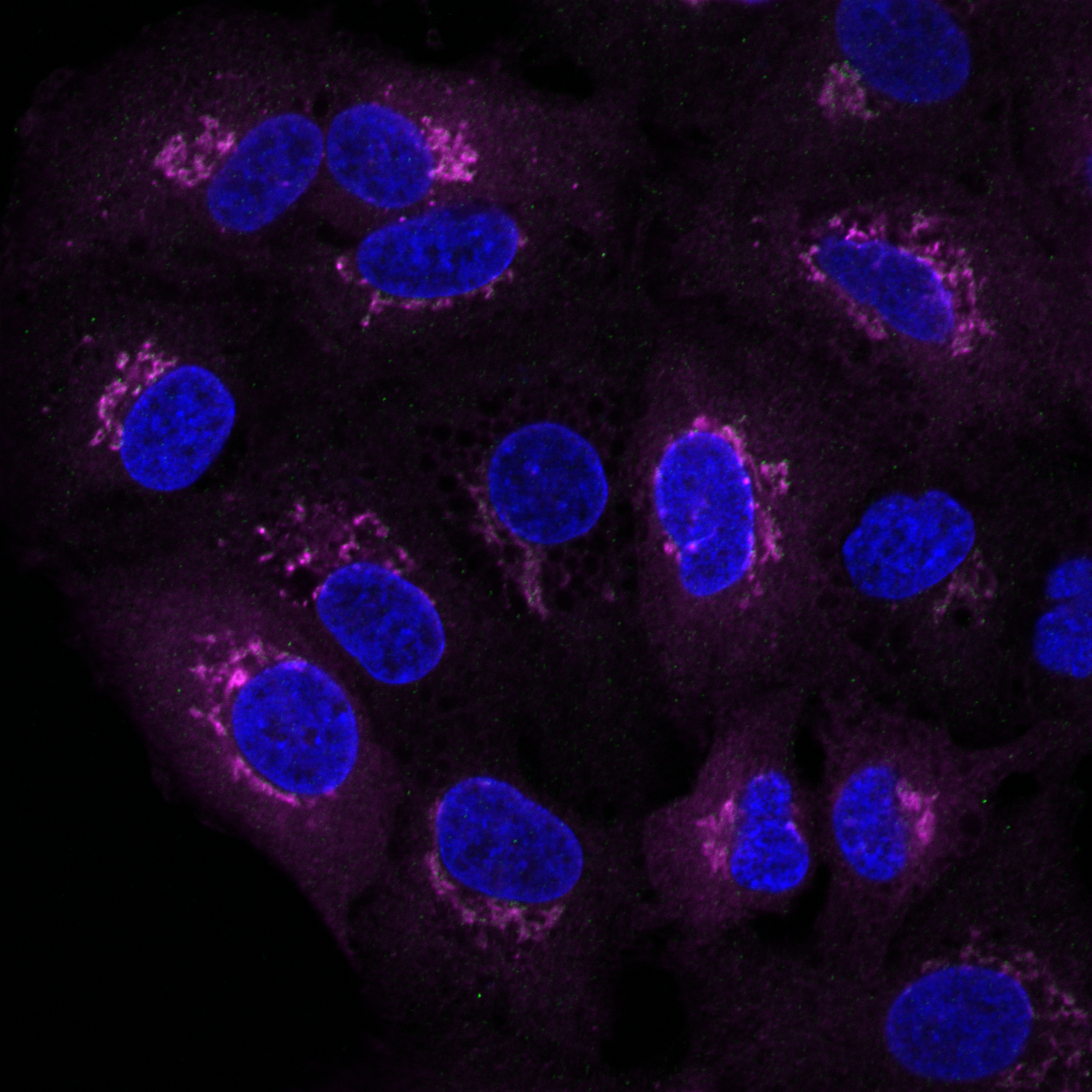

Supplement: Supplementary file 4 — Source data Fig. 3 [file 44318_2026_825_MOESM4_ESM.zip › Figure 3/3B/Figure_3B_IF/A549_p69/A549_p69_merge.tif]

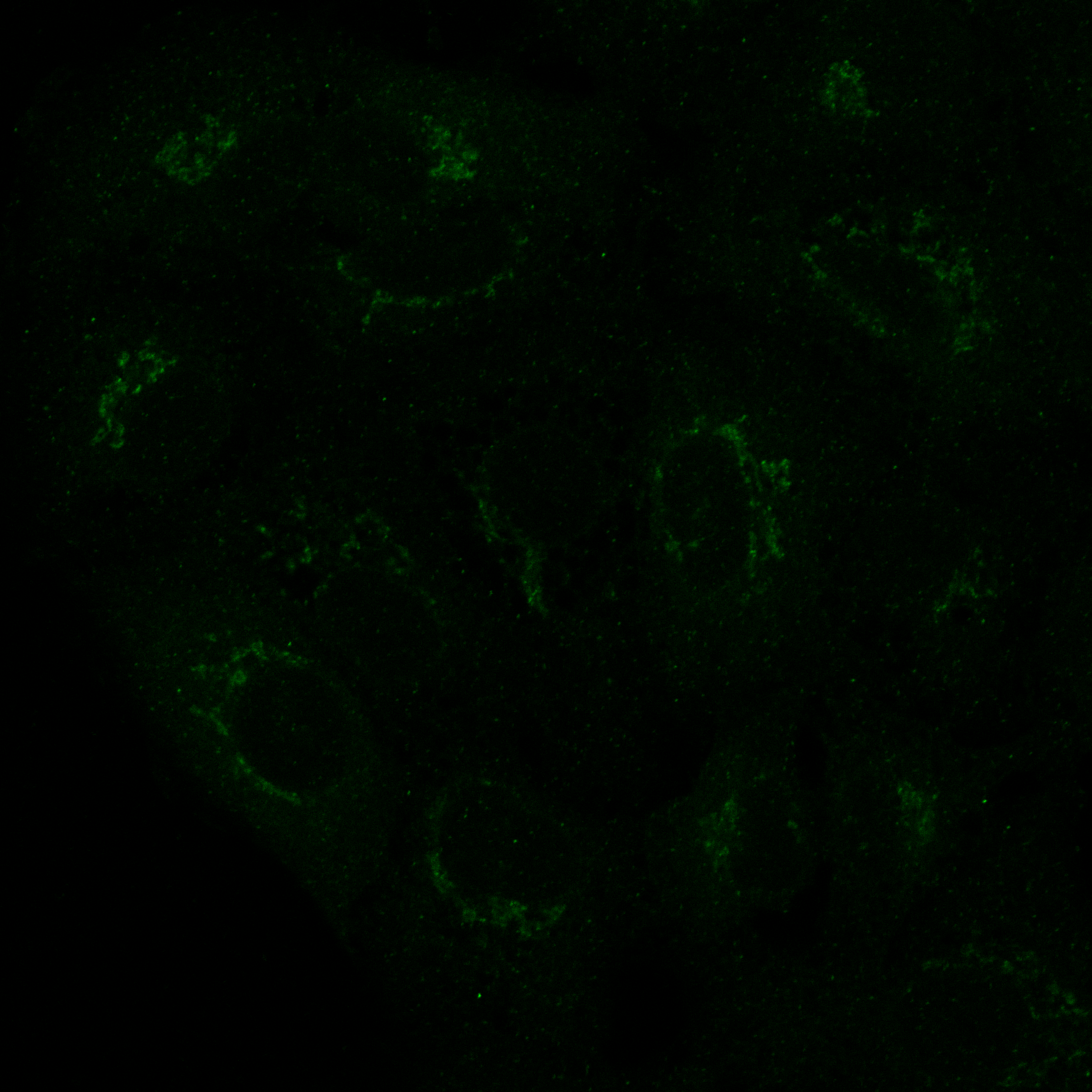

Supplement: Supplementary file 4 — Source data Fig. 3 [file 44318_2026_825_MOESM4_ESM.zip › Figure 3/3B/Figure_3B_IF/A549_p69/A549_p69_58K_green.tif]

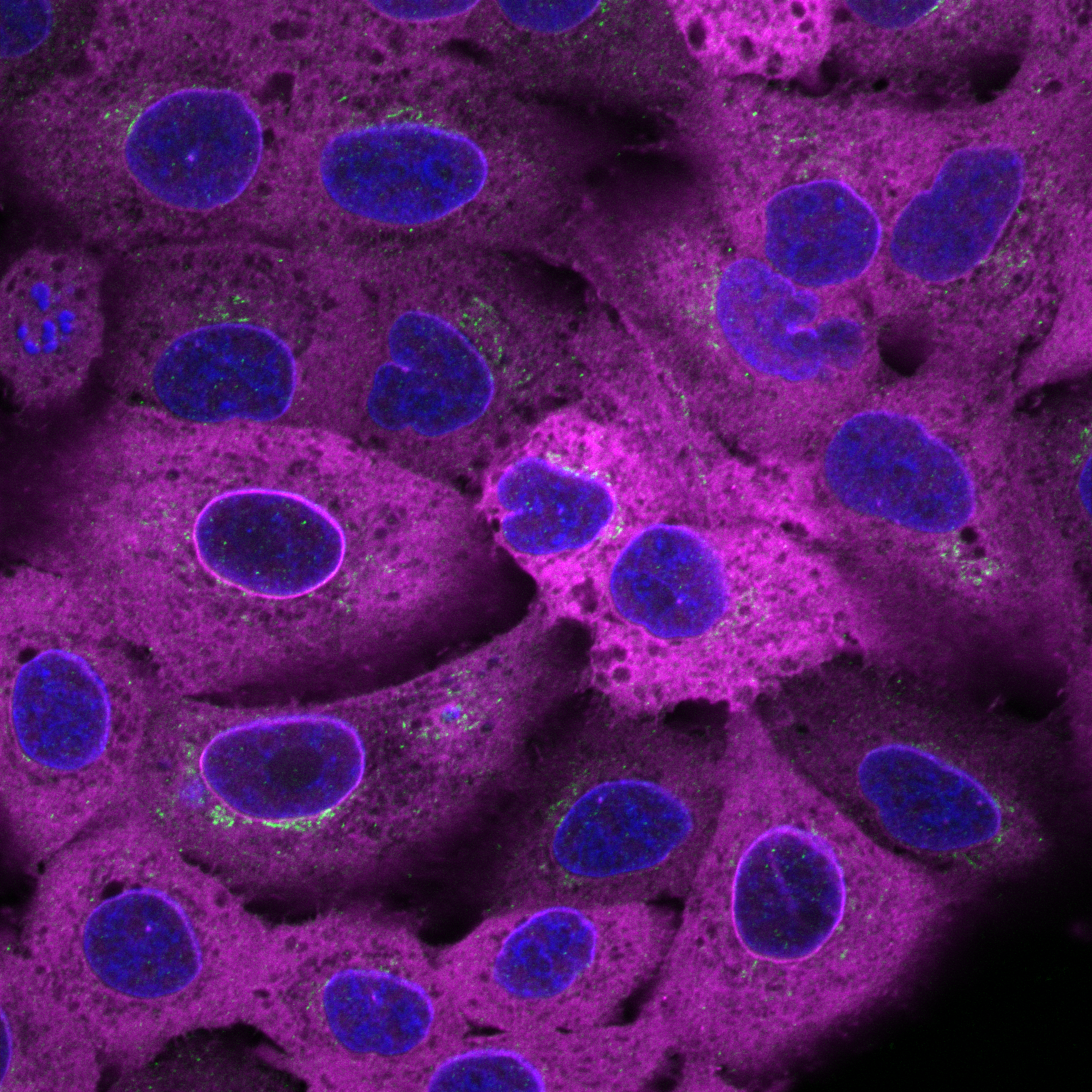

Supplement: Supplementary file 4 — Source data Fig. 3 [file 44318_2026_825_MOESM4_ESM.zip › Figure 3/3B/Figure_3B_IF/A549_p69G2A/A549_p69G2A_merge.tif]

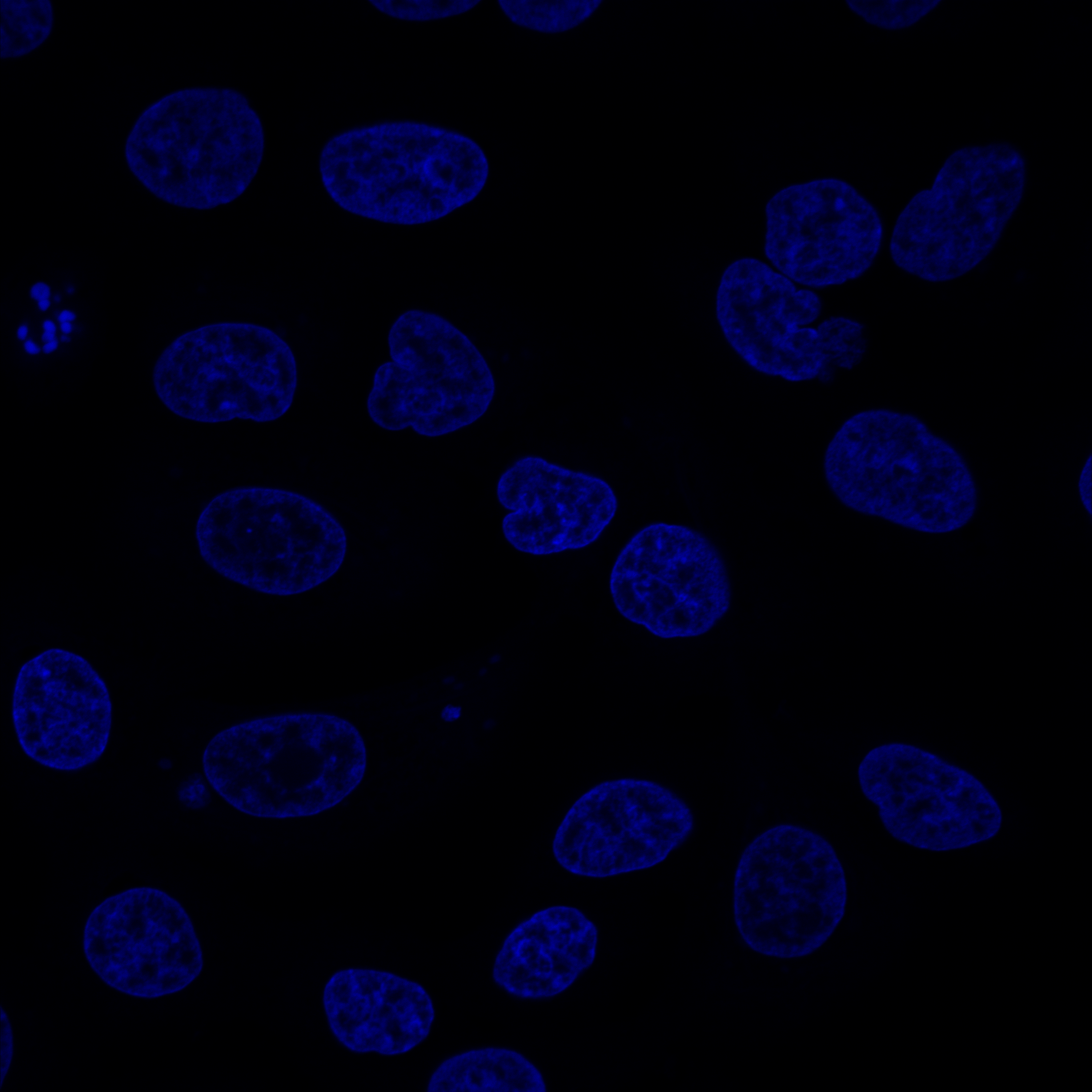

Supplement: Supplementary file 4 — Source data Fig. 3 [file 44318_2026_825_MOESM4_ESM.zip › Figure 3/3B/Figure_3B_IF/A549_p69G2A/A549_p69G2A_DNA_blue.tif]

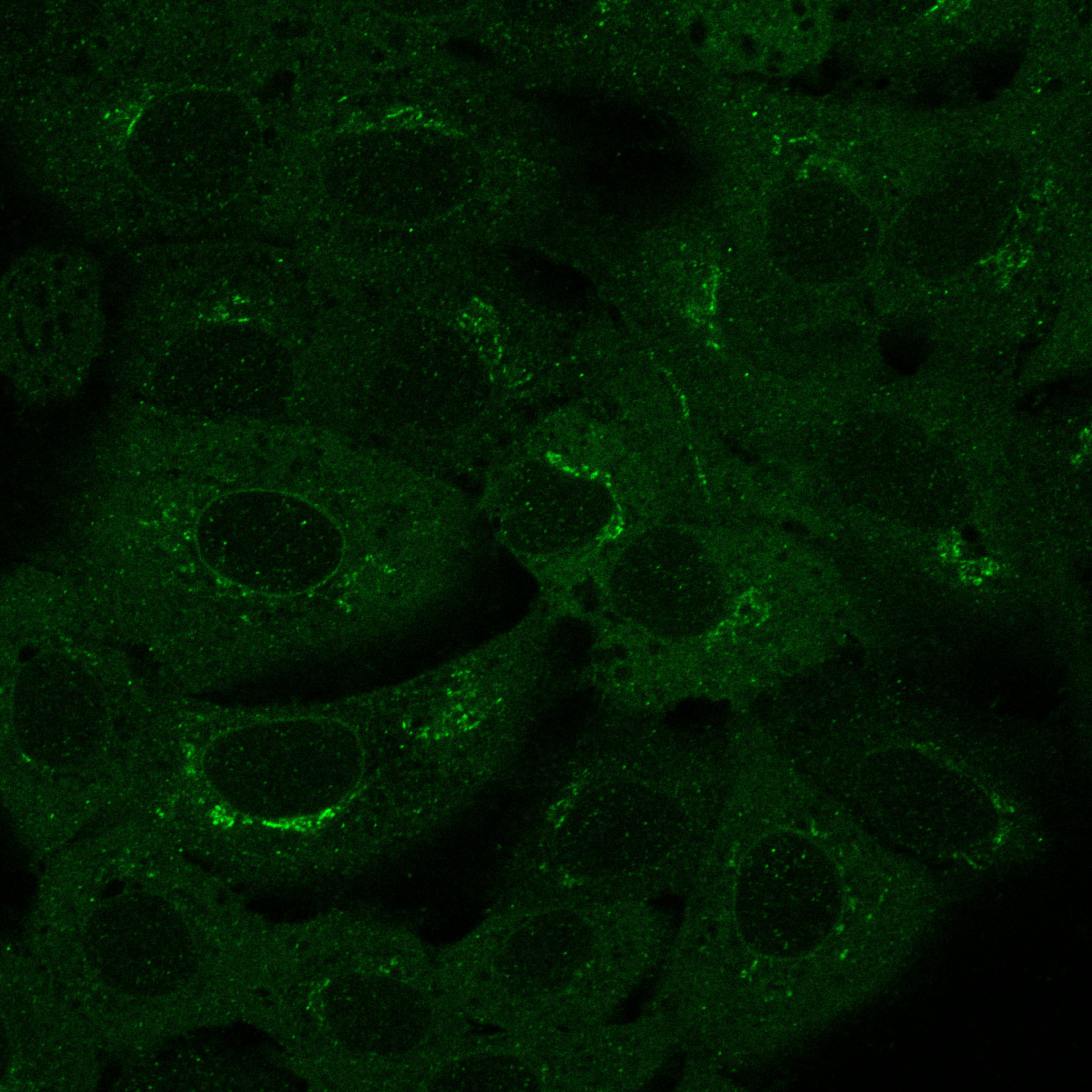

Supplement: Supplementary file 4 — Source data Fig. 3 [file 44318_2026_825_MOESM4_ESM.zip › Figure 3/3B/Figure_3B_IF/A549_p69G2A/A549_p69G2A_58K_green.tif]

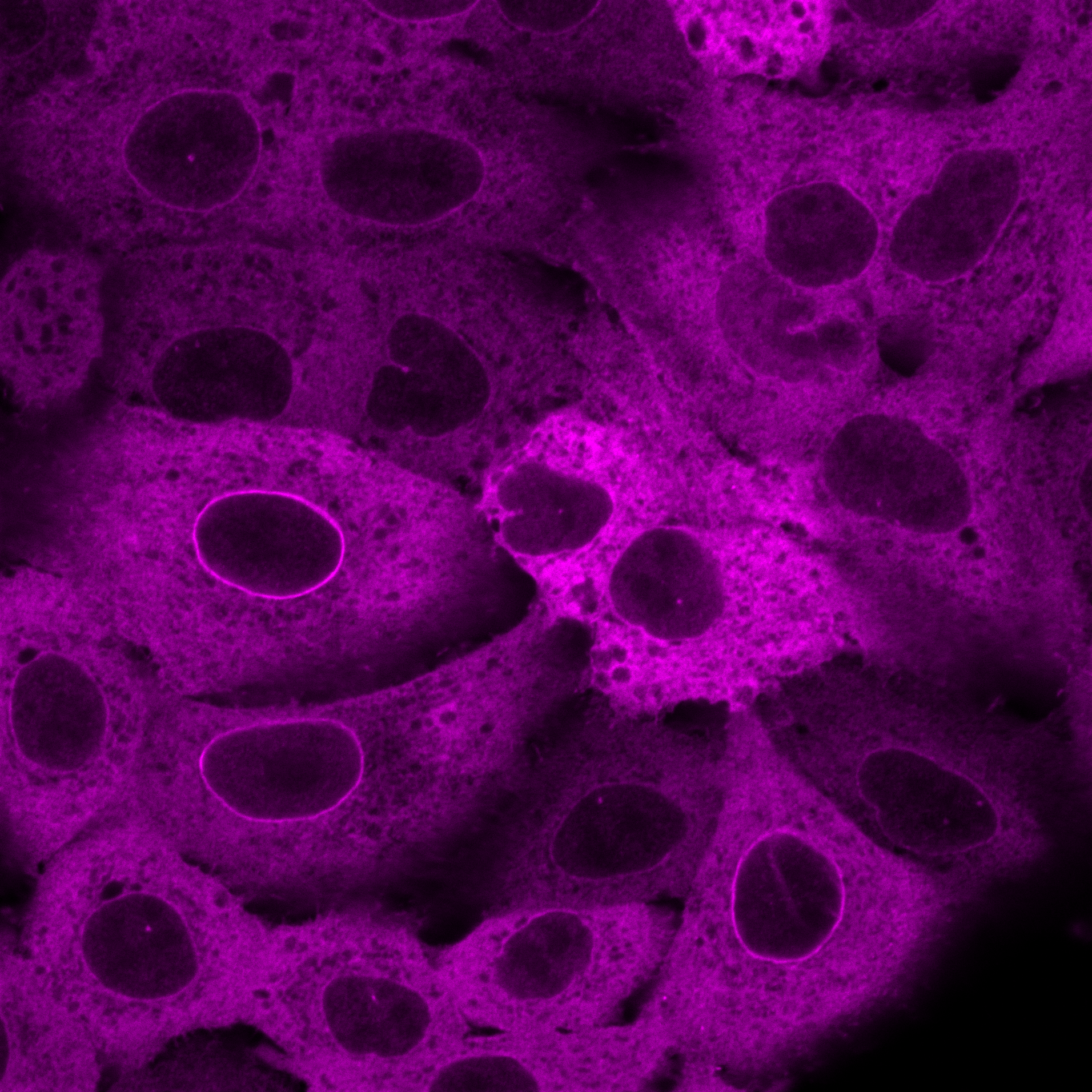

Supplement: Supplementary file 4 — Source data Fig. 3 [file 44318_2026_825_MOESM4_ESM.zip › Figure 3/3B/Figure_3B_IF/A549_p69G2A/A549_p69_OAS2_magenta.tif]

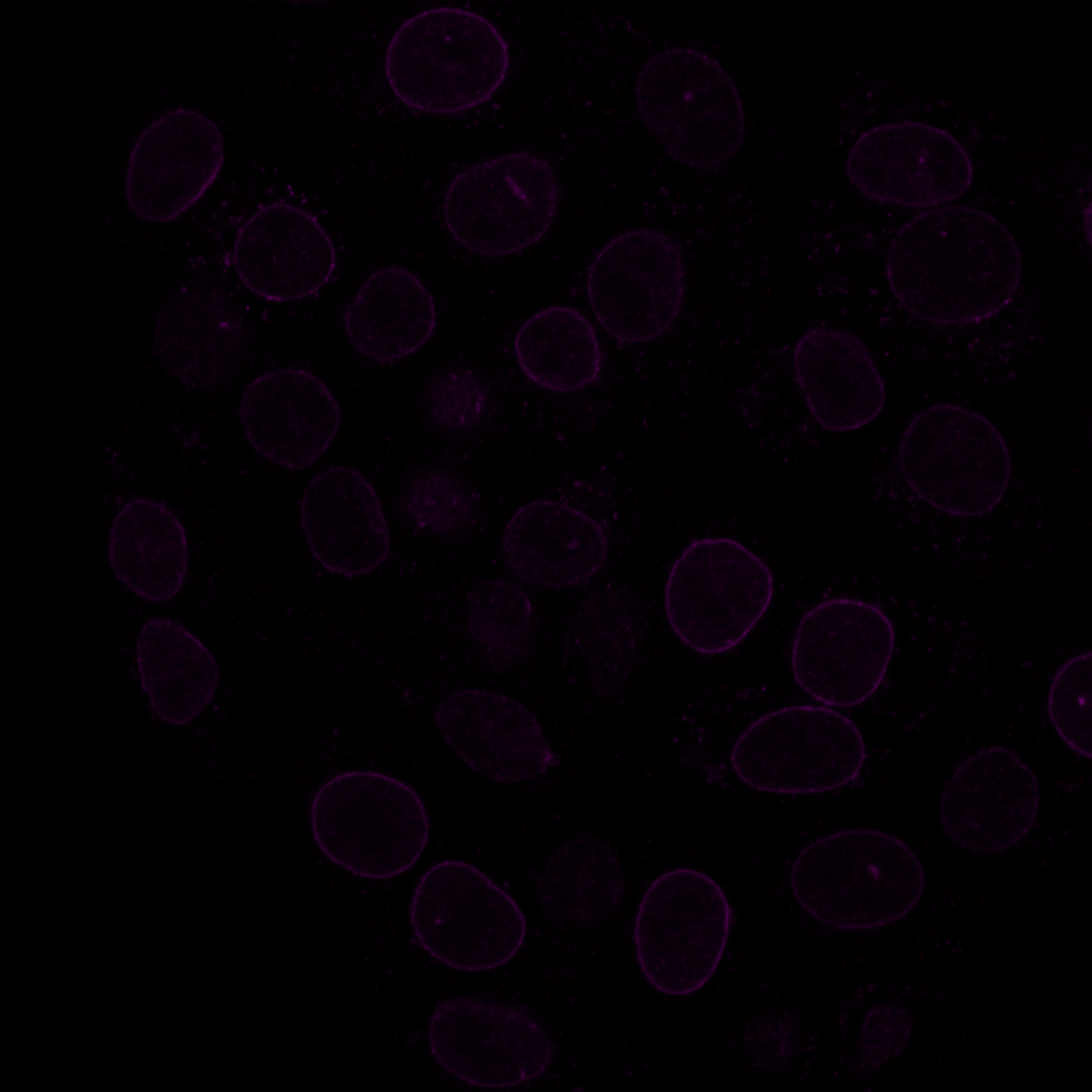

Supplement: Supplementary file 4 — Source data Fig. 3 [file 44318_2026_825_MOESM4_ESM.zip › Figure 3/3B/Figure_3B_IF/A549_untreated/A549_untreated_OAS2_magenta.tif]

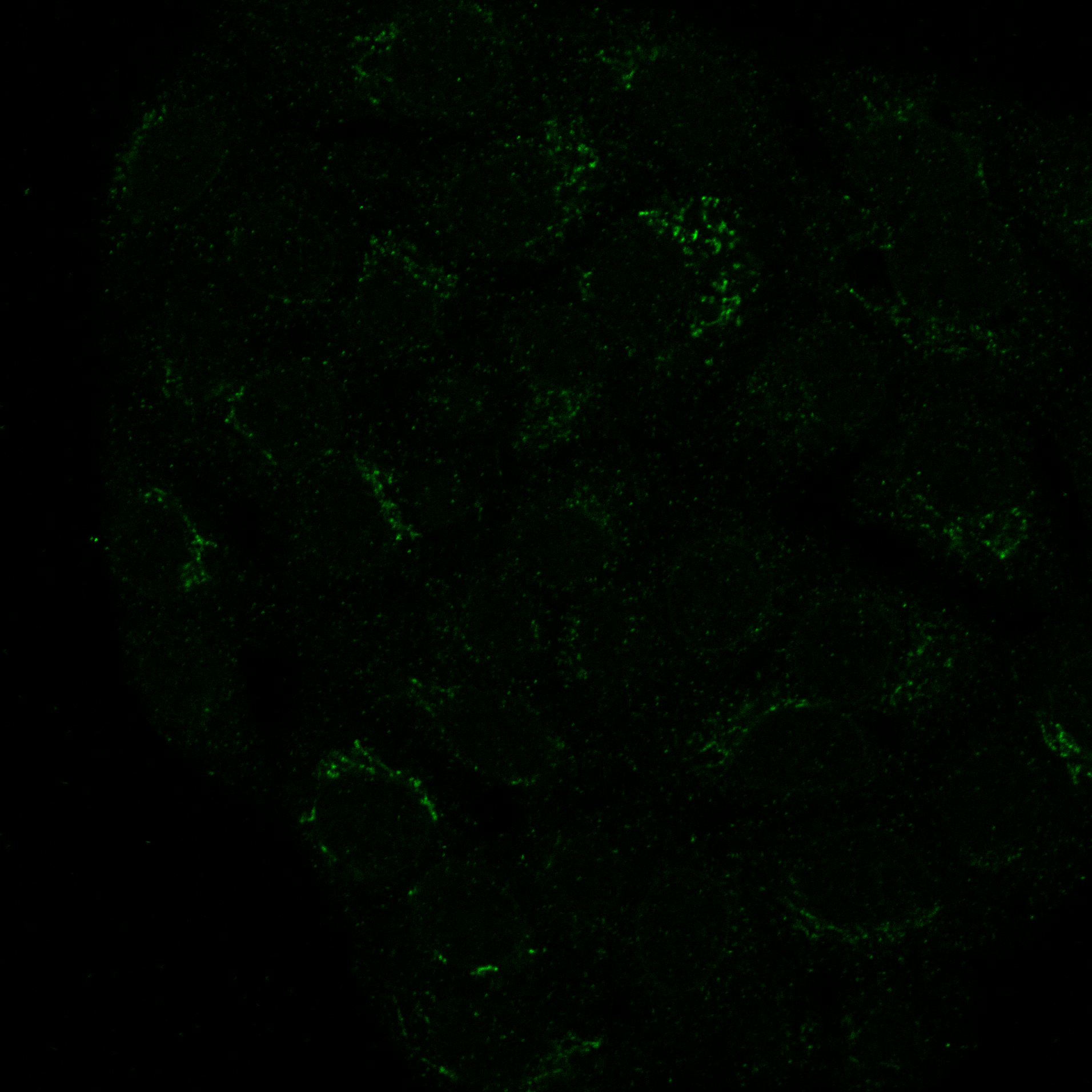

Supplement: Supplementary file 4 — Source data Fig. 3 [file 44318_2026_825_MOESM4_ESM.zip › Figure 3/3B/Figure_3B_IF/A549_untreated/A549_untreated_58K_green.tif]

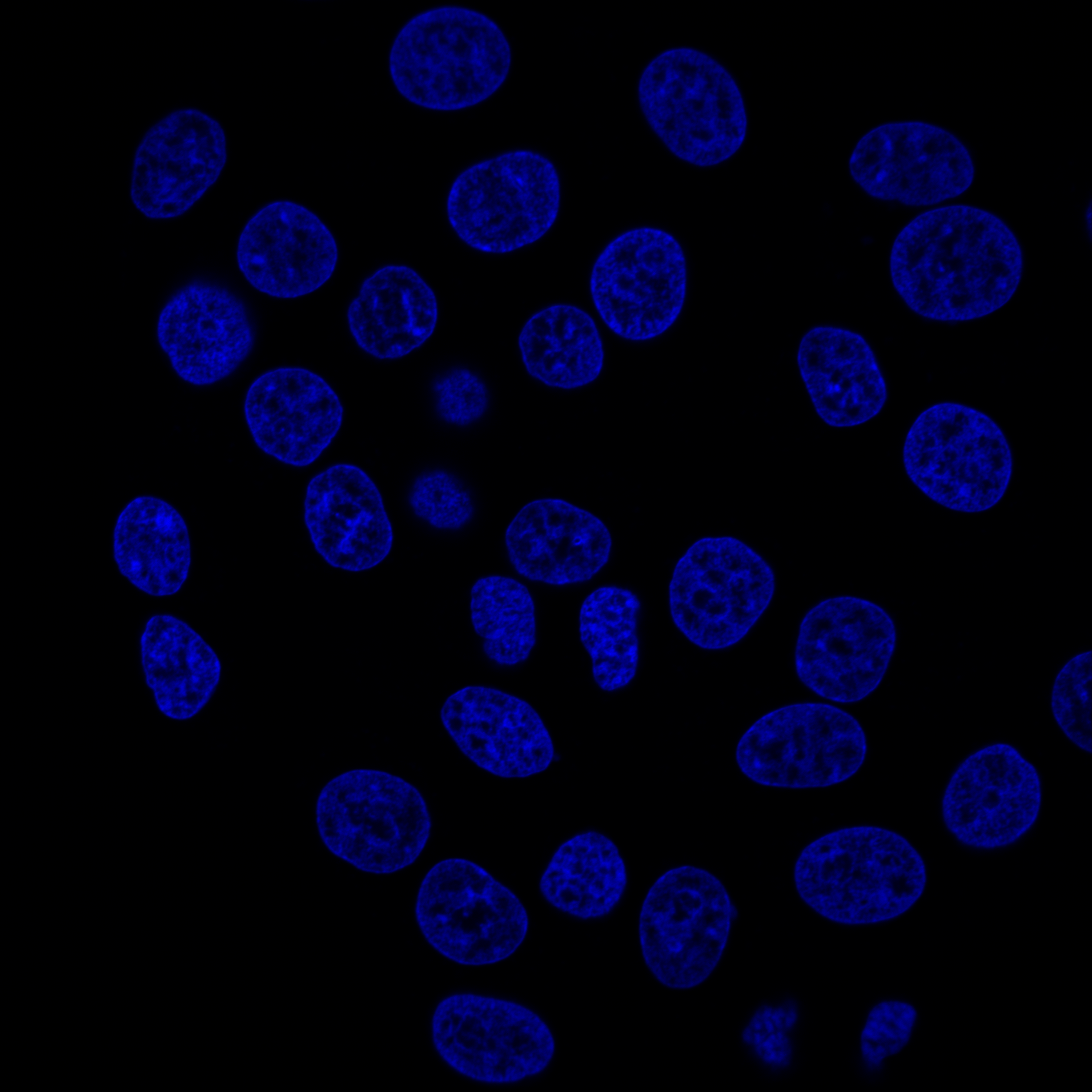

Supplement: Supplementary file 4 — Source data Fig. 3 [file 44318_2026_825_MOESM4_ESM.zip › Figure 3/3B/Figure_3B_IF/A549_untreated/A549_untreated_DNA_blue.tif]

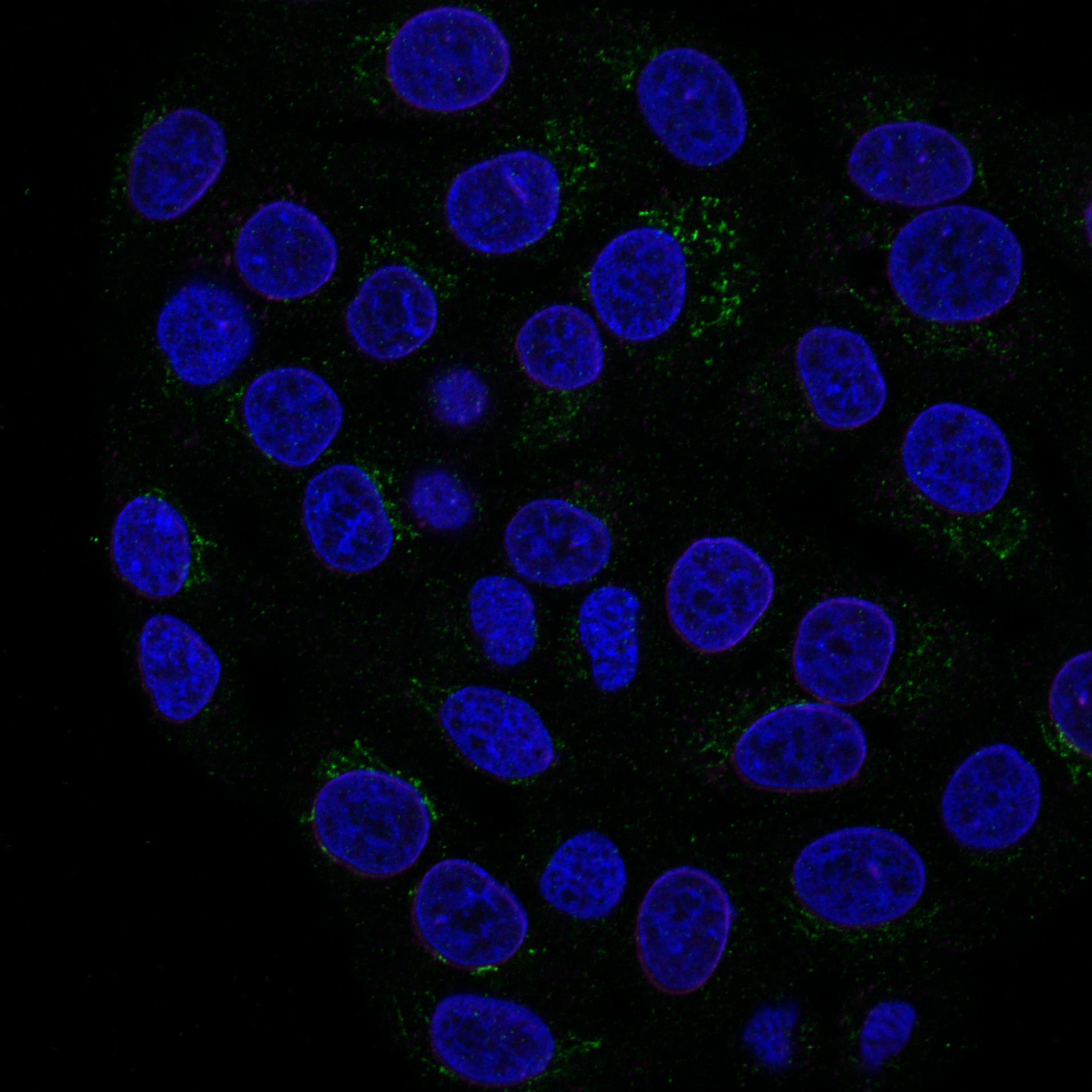

Supplement: Supplementary file 4 — Source data Fig. 3 [file 44318_2026_825_MOESM4_ESM.zip › Figure 3/3B/Figure_3B_IF/A549_untreated/A549_untreated_merge.tif]

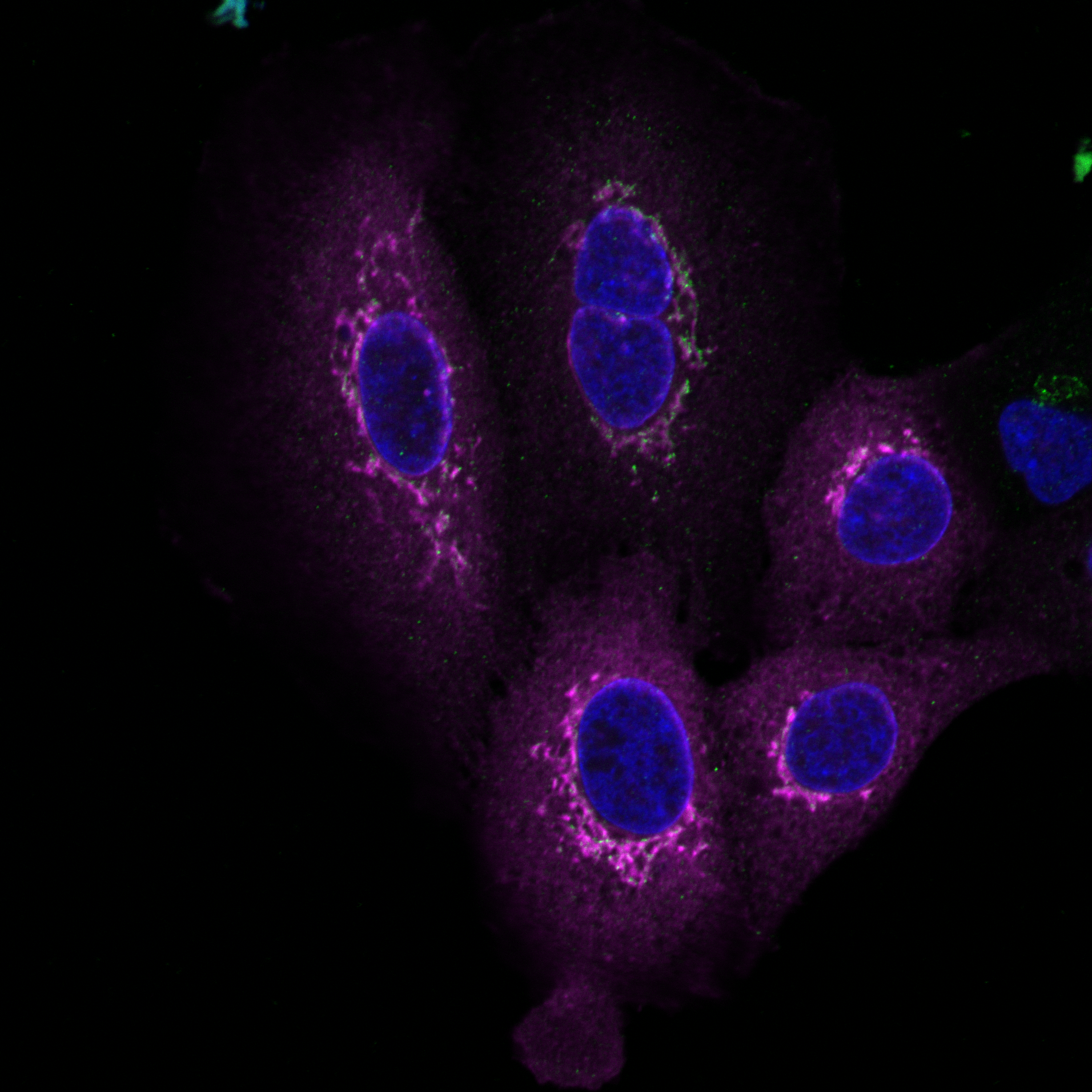

Supplement: Supplementary file 4 — Source data Fig. 3 [file 44318_2026_825_MOESM4_ESM.zip › Figure 3/3B/Figure_3B_IF/A549_p71/A549_p71_merge.tif]

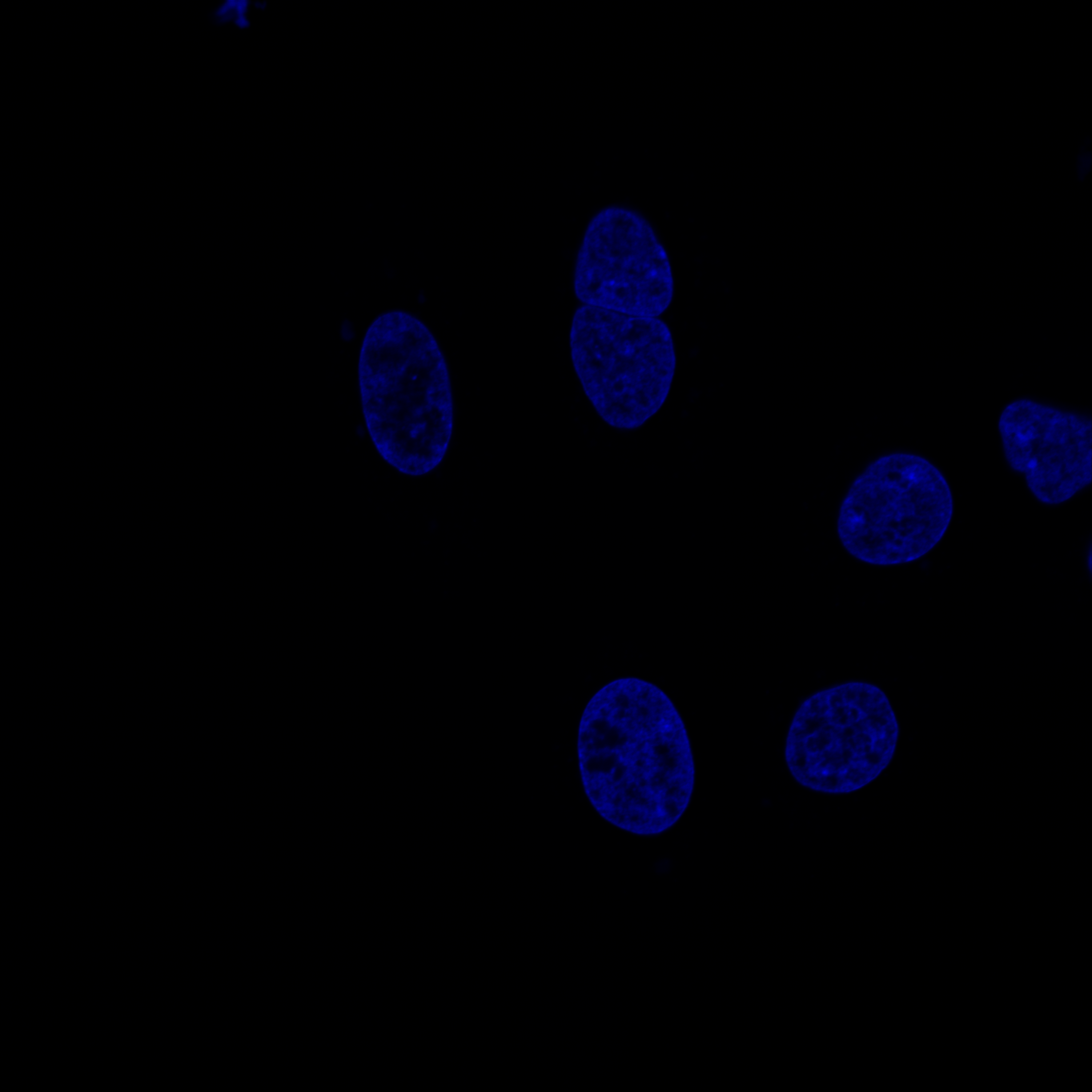

Supplement: Supplementary file 4 — Source data Fig. 3 [file 44318_2026_825_MOESM4_ESM.zip › Figure 3/3B/Figure_3B_IF/A549_p71/A549_p71_DNA_blue.tif]

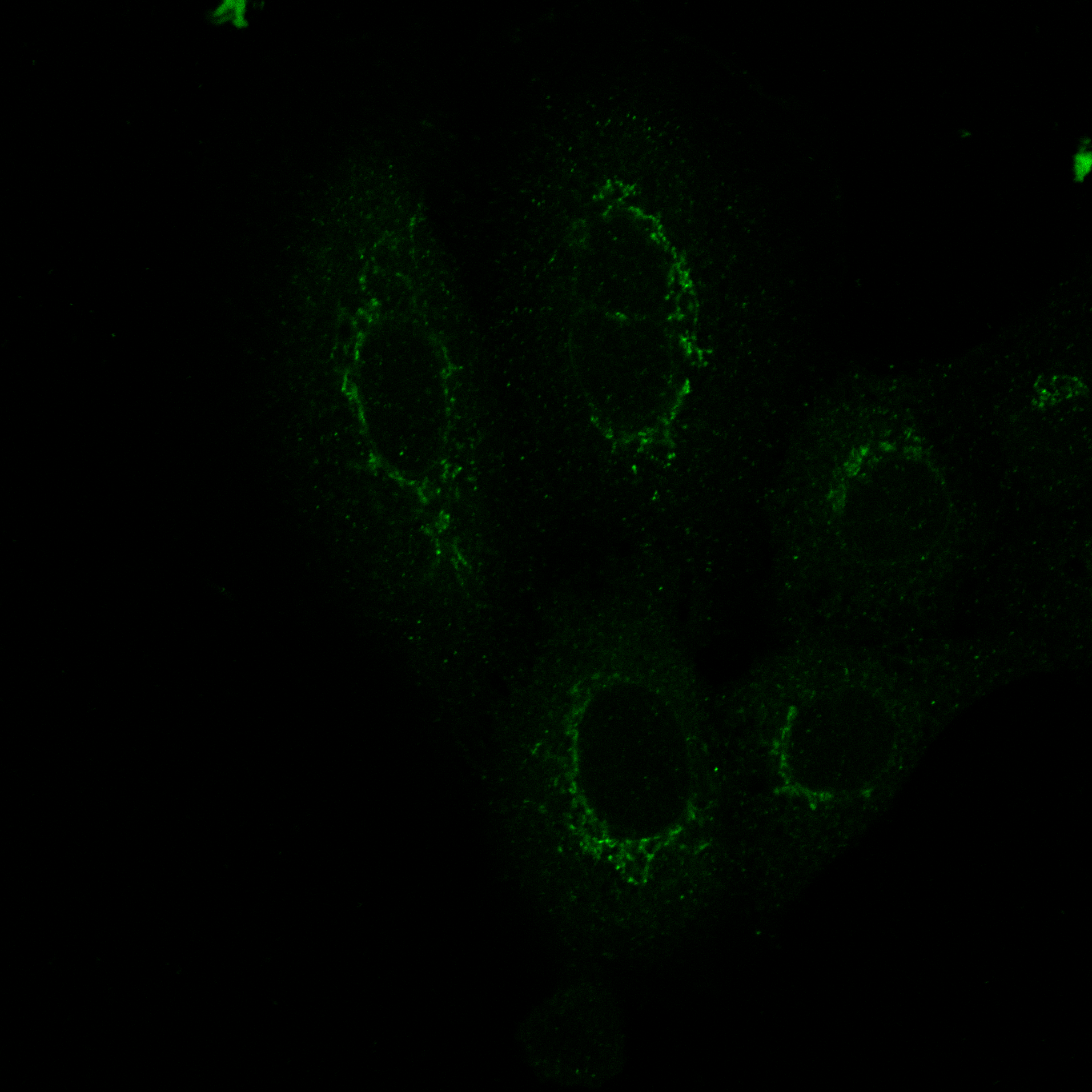

Supplement: Supplementary file 4 — Source data Fig. 3 [file 44318_2026_825_MOESM4_ESM.zip › Figure 3/3B/Figure_3B_IF/A549_p71/A549_p71_58K_green.tif]

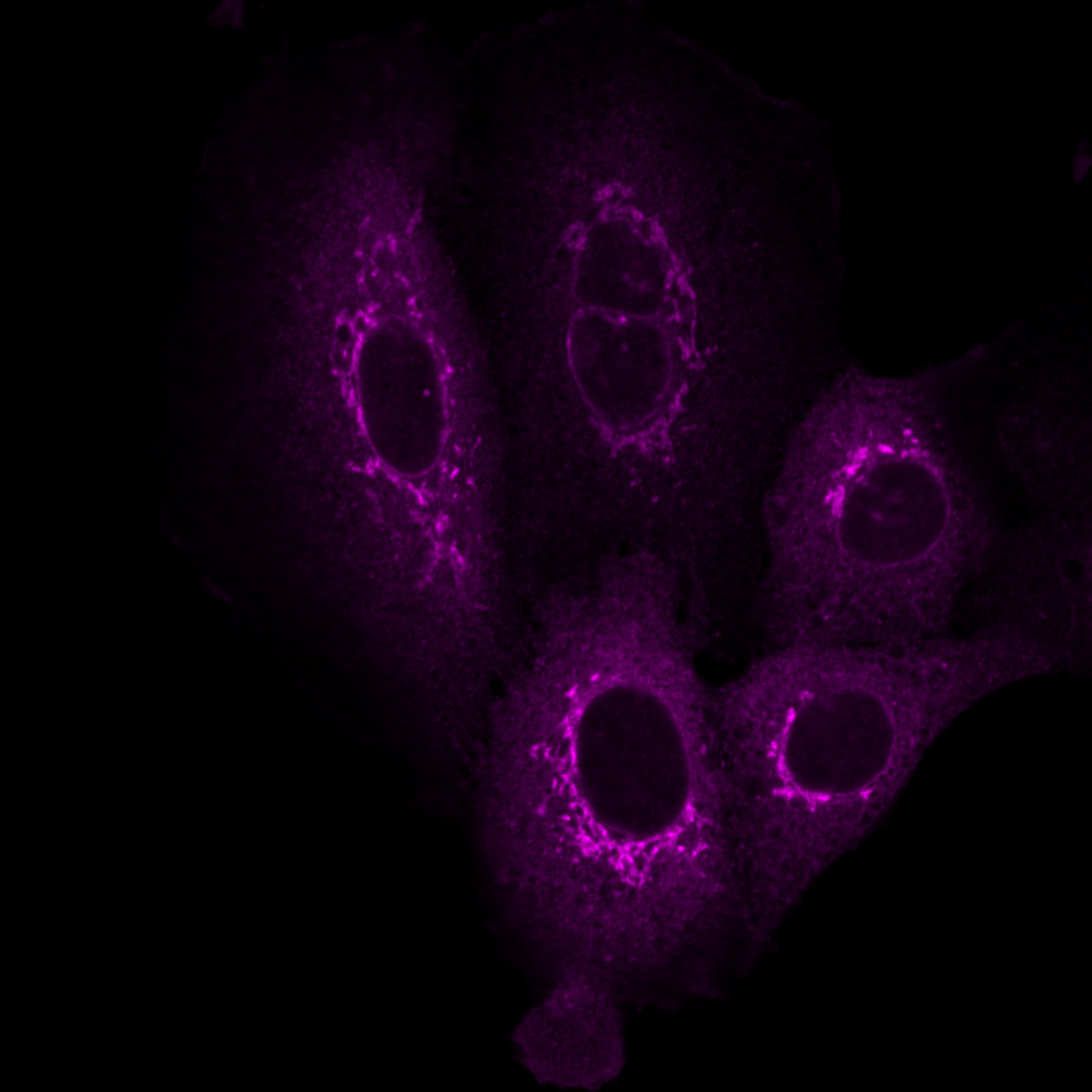

Supplement: Supplementary file 4 — Source data Fig. 3 [file 44318_2026_825_MOESM4_ESM.zip › Figure 3/3B/Figure_3B_IF/A549_p71/A549_p71_OAS2_magenta.tif]

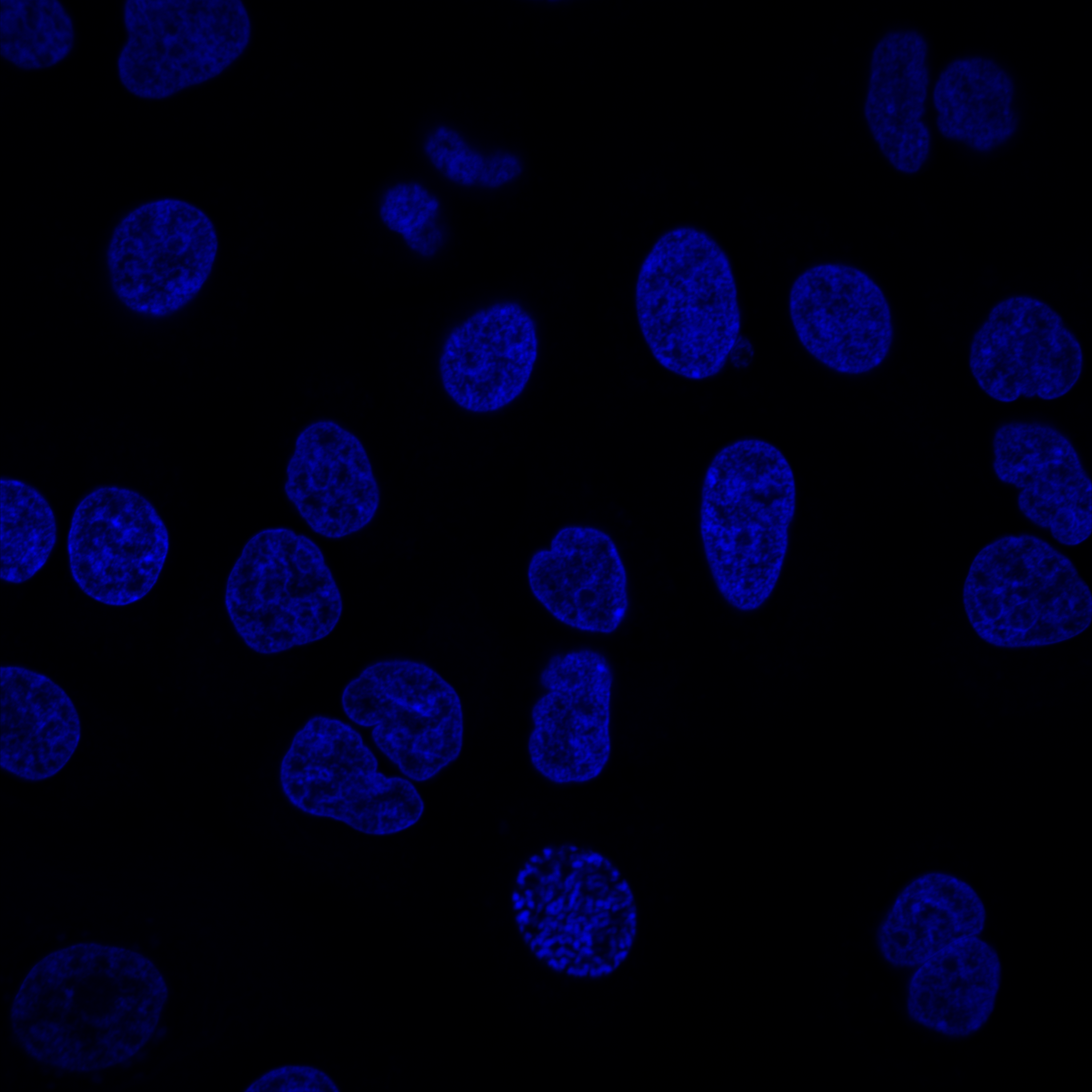

Supplement: Supplementary file 4 — Source data Fig. 3 [file 44318_2026_825_MOESM4_ESM.zip › Figure 3/3B/Figure_3B_IF/A549_p71G2A/A549_p71G2A_DNA_blue.tif]

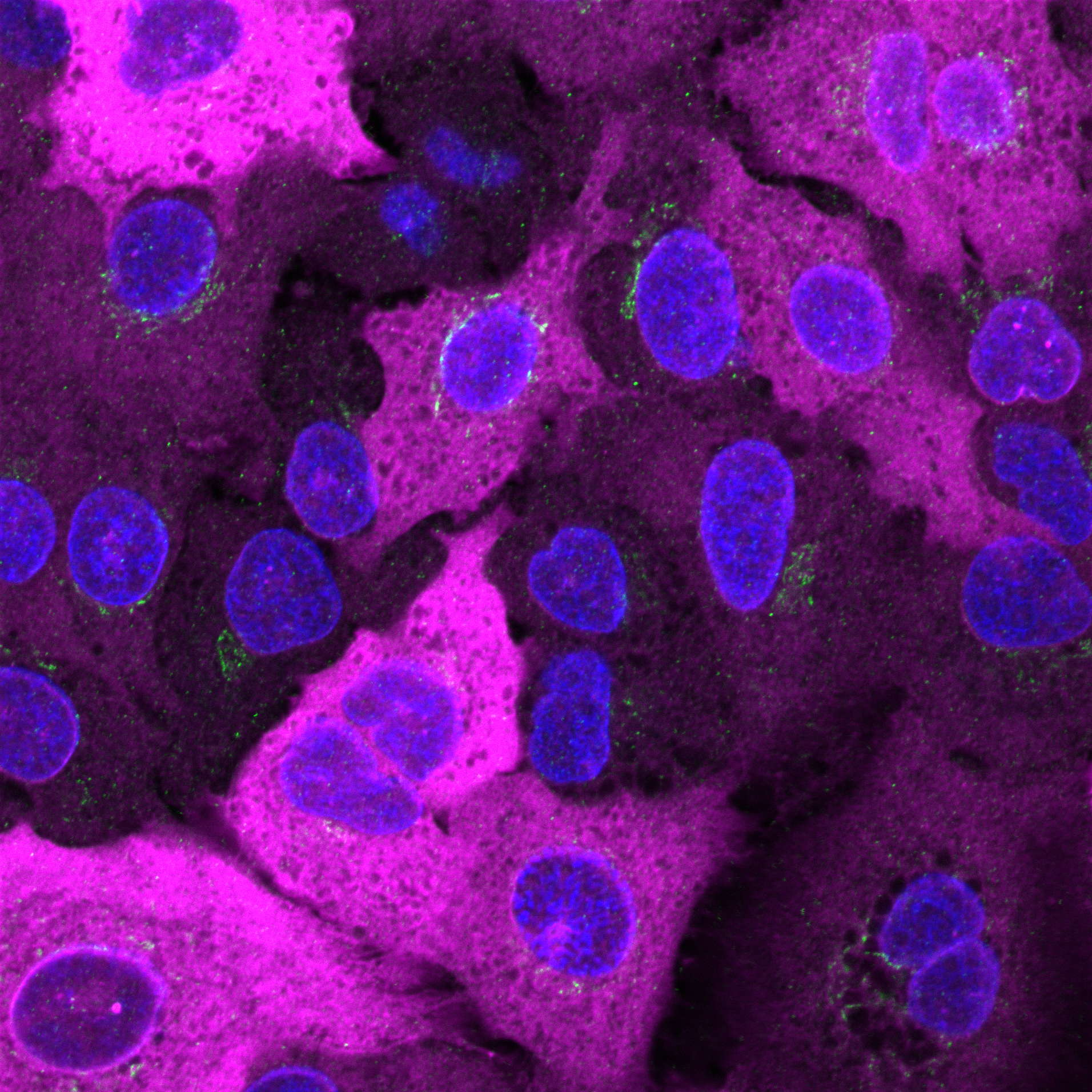

Supplement: Supplementary file 4 — Source data Fig. 3 [file 44318_2026_825_MOESM4_ESM.zip › Figure 3/3B/Figure_3B_IF/A549_p71G2A/A549_p71G2A_merge.tif]

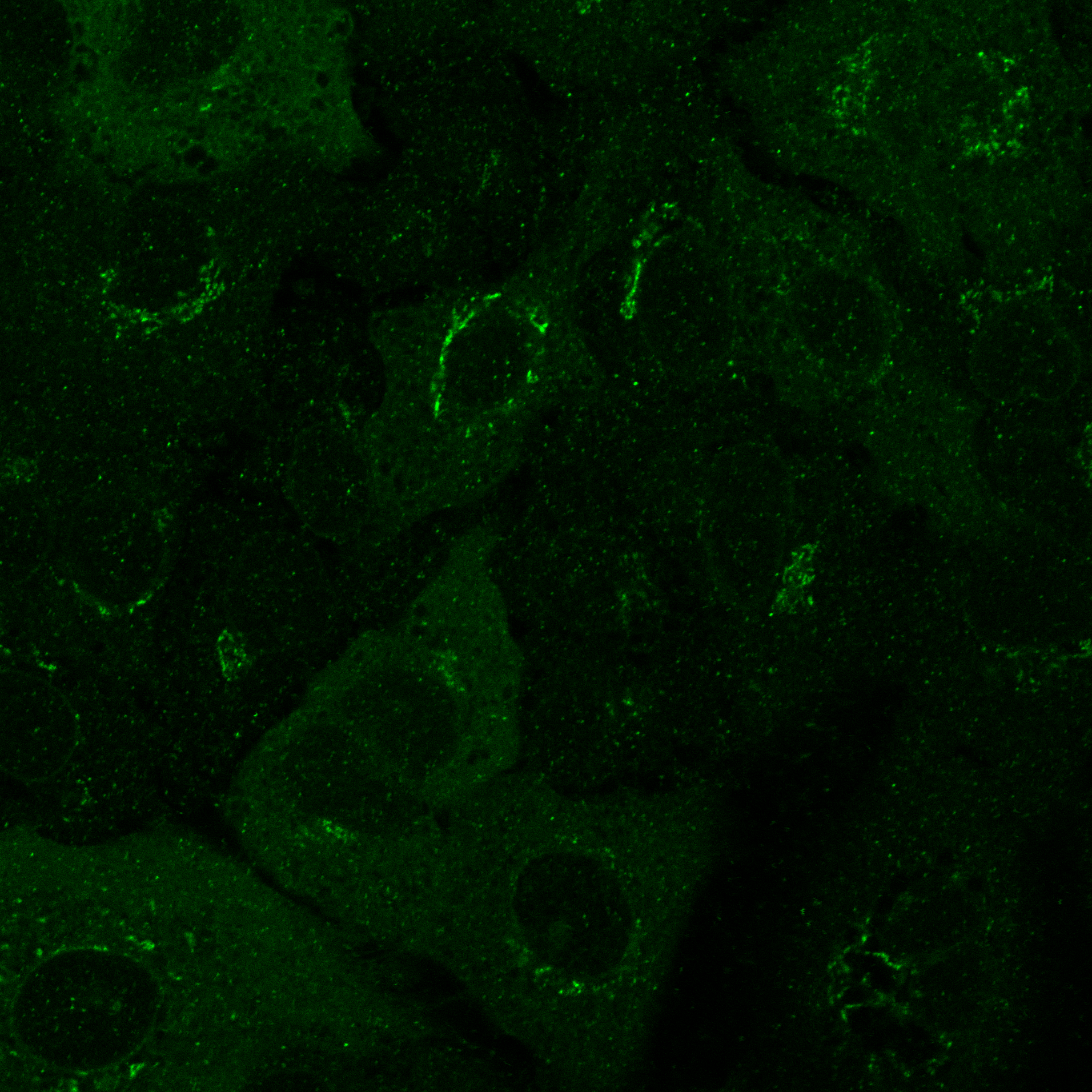

Supplement: Supplementary file 4 — Source data Fig. 3 [file 44318_2026_825_MOESM4_ESM.zip › Figure 3/3B/Figure_3B_IF/A549_p71G2A/A549_p71G2A_58K_green.tif]

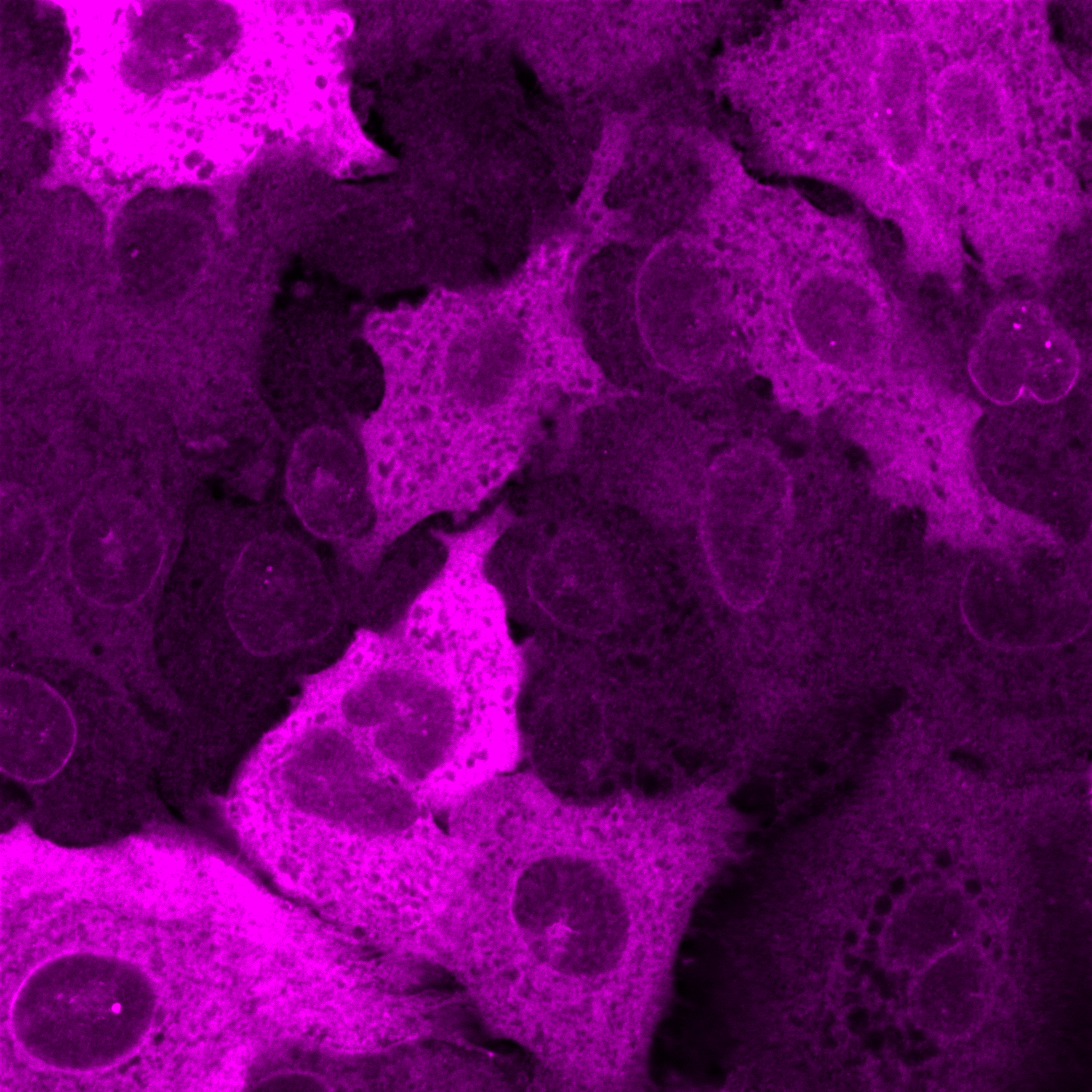

Supplement: Supplementary file 4 — Source data Fig. 3 [file 44318_2026_825_MOESM4_ESM.zip › Figure 3/3B/Figure_3B_IF/A549_p71G2A/A549_p71G2A_OAS2_magenta.tif]

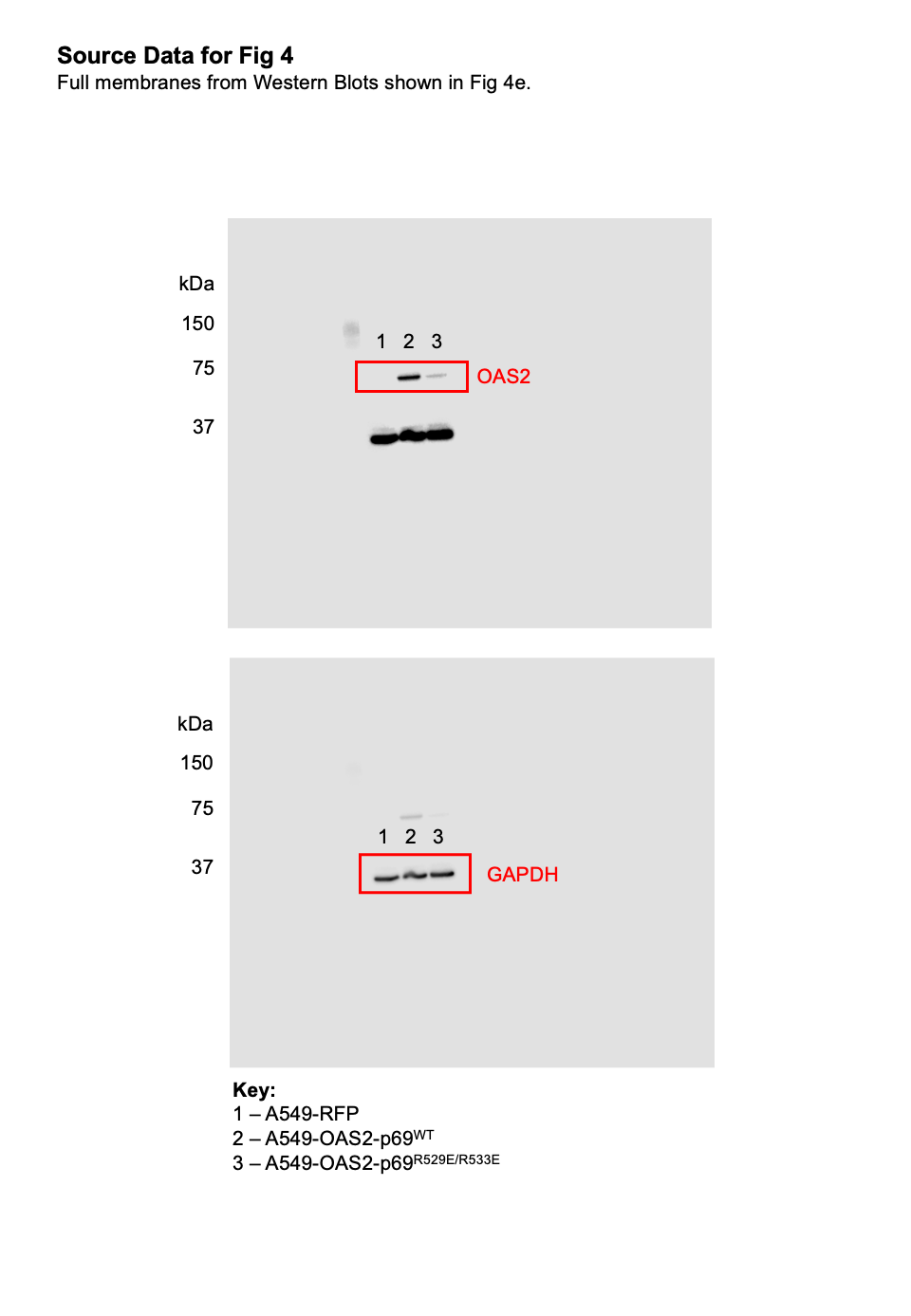

Supplement: Supplementary file 5 — Source data Fig. 4 [file 44318_2026_825_MOESM5_ESM.zip › Figure 4/4E/Figure_4E_WesternBlot.tiff]

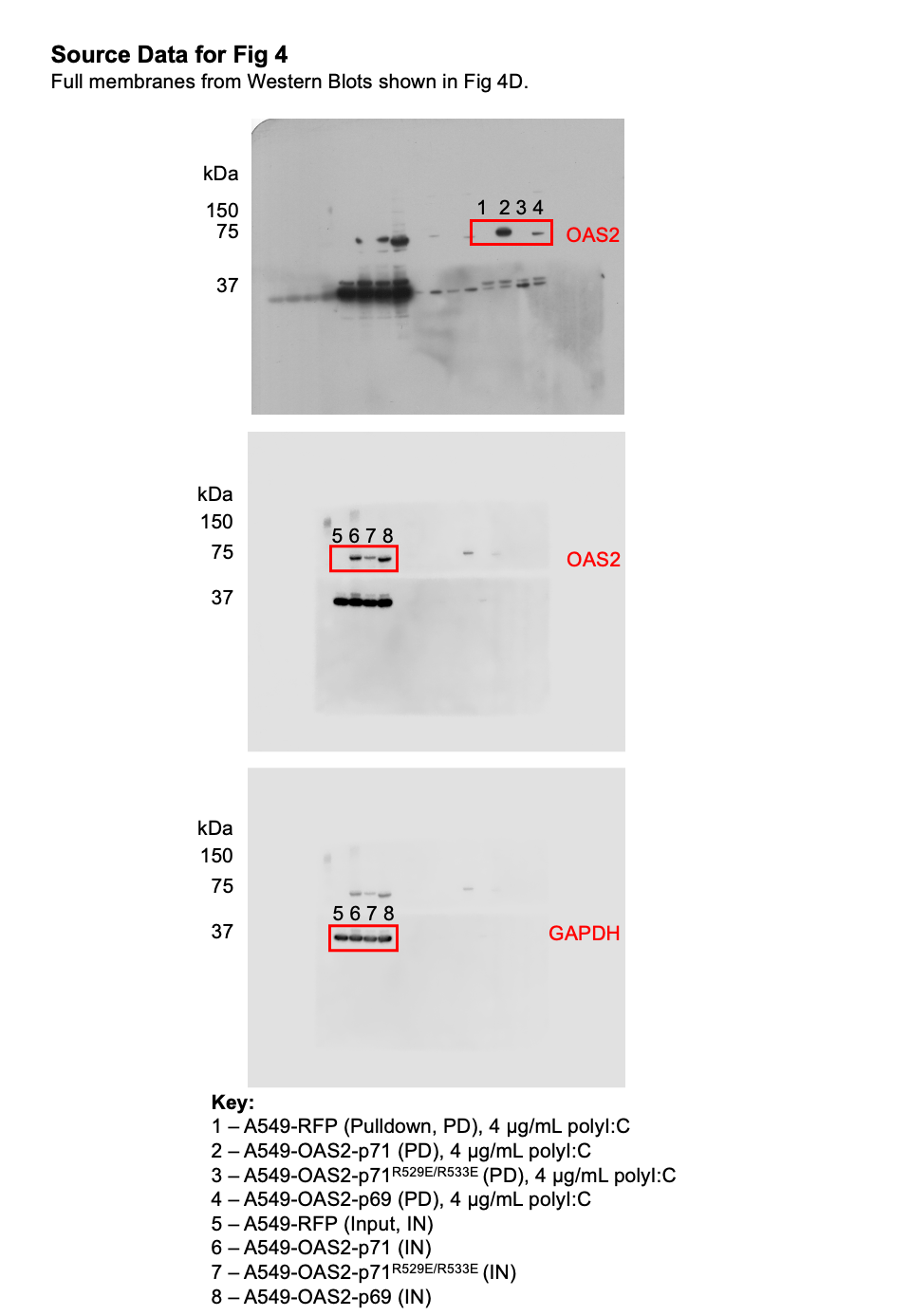

Supplement: Supplementary file 5 — Source data Fig. 4 [file 44318_2026_825_MOESM5_ESM.zip › Figure 4/4D/Figure_4D_WesternBlot.tiff]

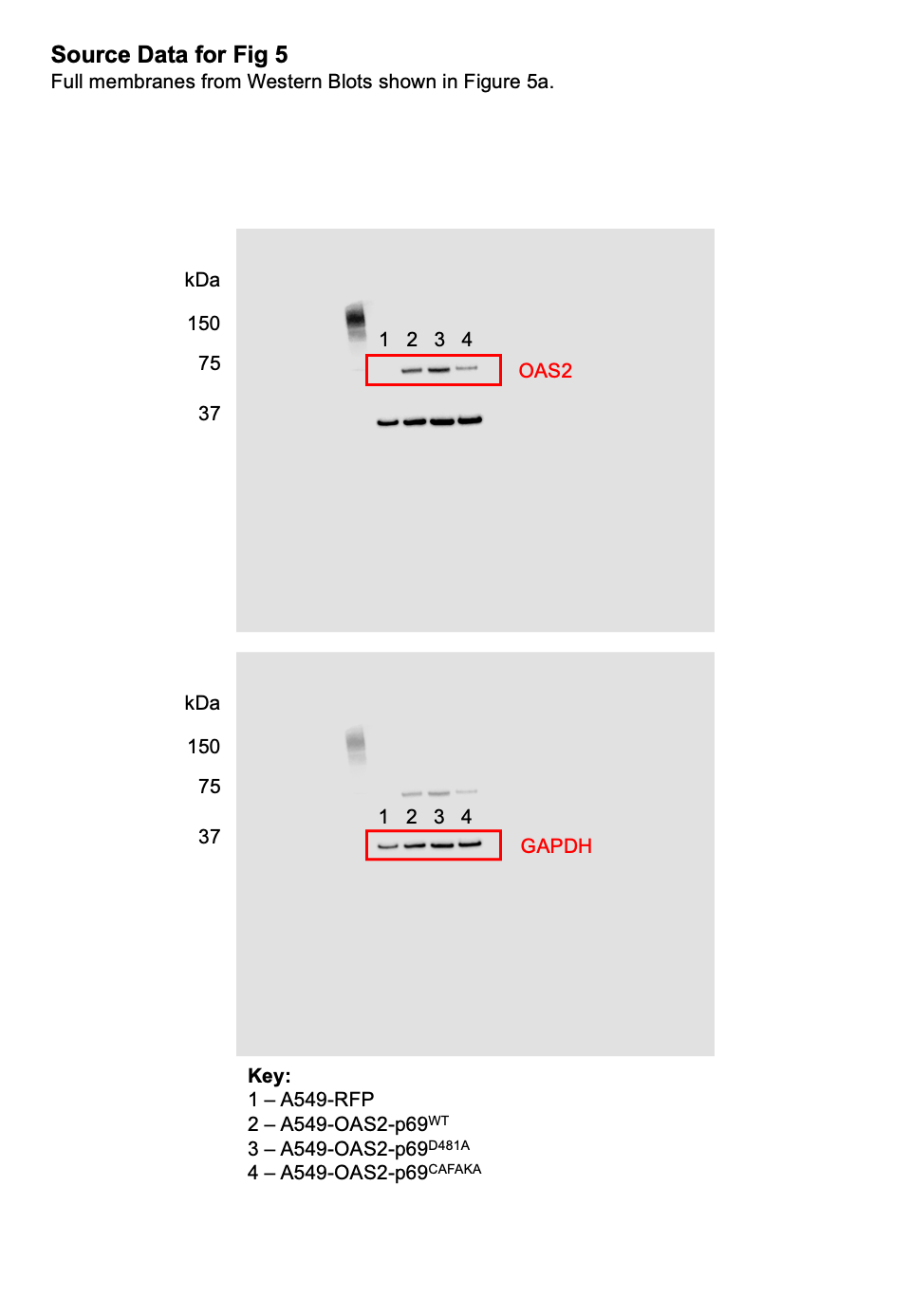

Supplement: Supplementary file 6 — Source data Fig. 5 [file 44318_2026_825_MOESM6_ESM.zip › Figure 5/5A/Figure_5A_WesternBlot.tiff]

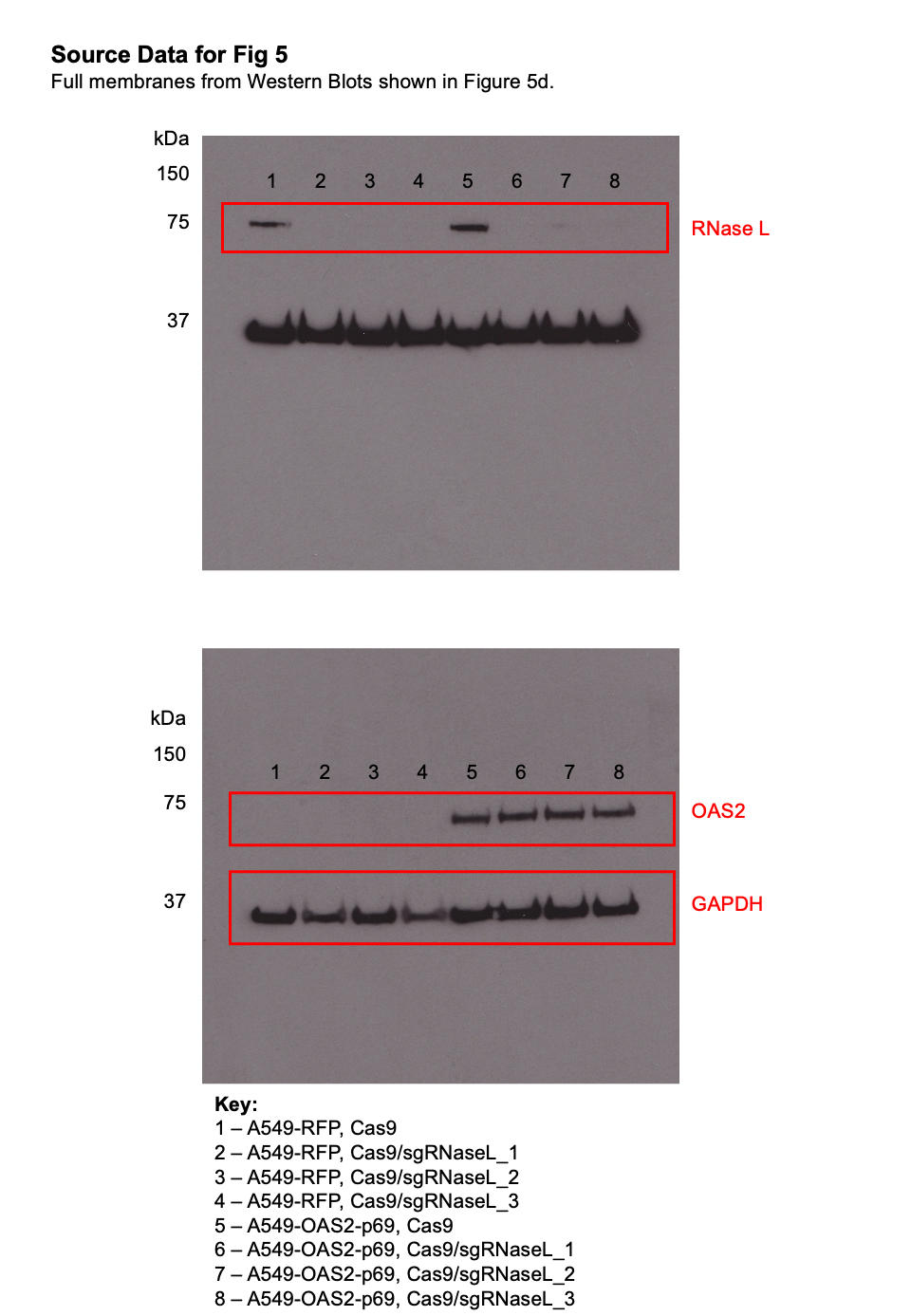

Supplement: Supplementary file 6 — Source data Fig. 5 [file 44318_2026_825_MOESM6_ESM.zip › Figure 5/5D/Figure_5D_WesternBlot.tiff]

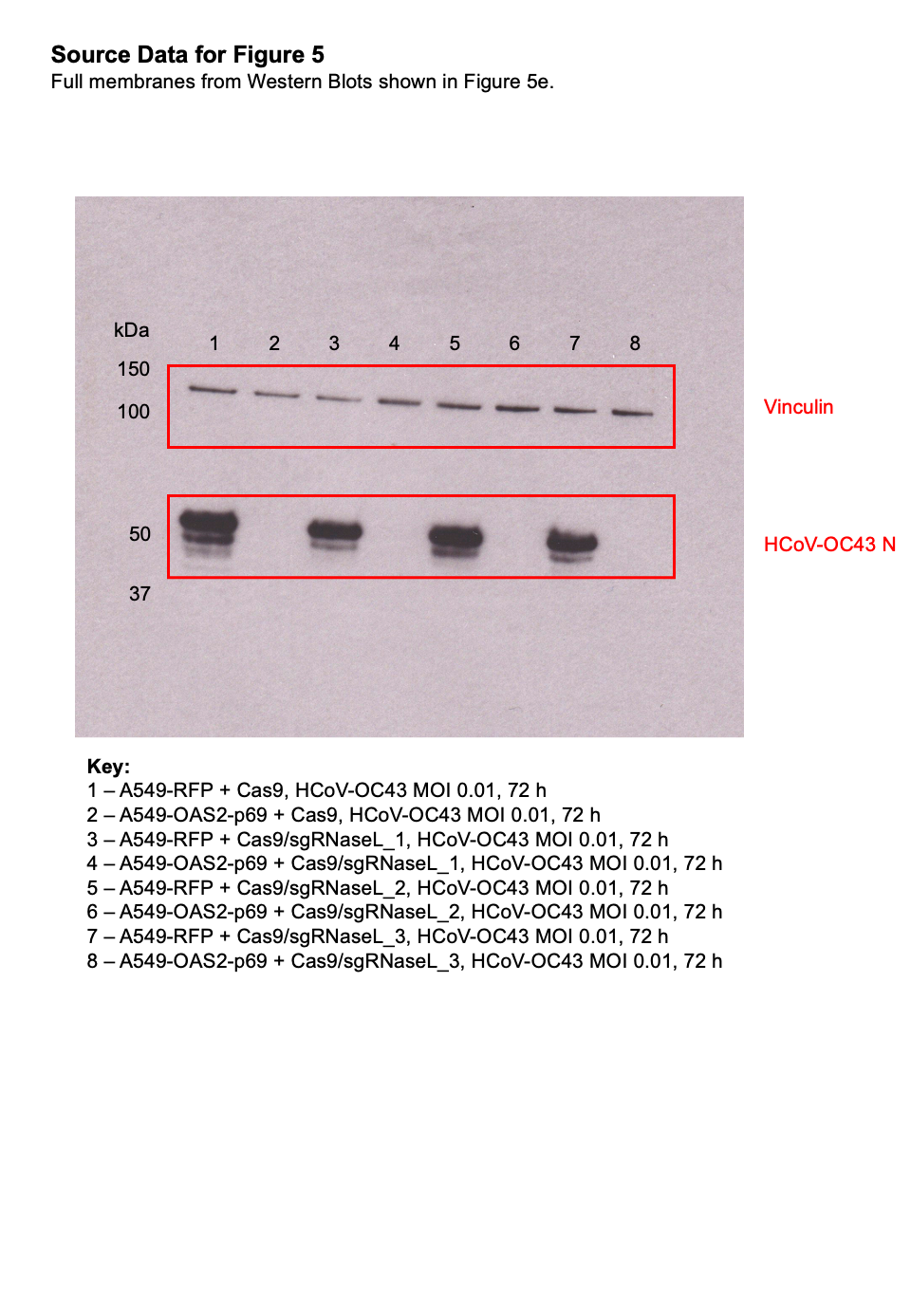

Supplement: Supplementary file 6 — Source data Fig. 5 [file 44318_2026_825_MOESM6_ESM.zip › Figure 5/5E/Figure_5E_WesternBlot.tiff]

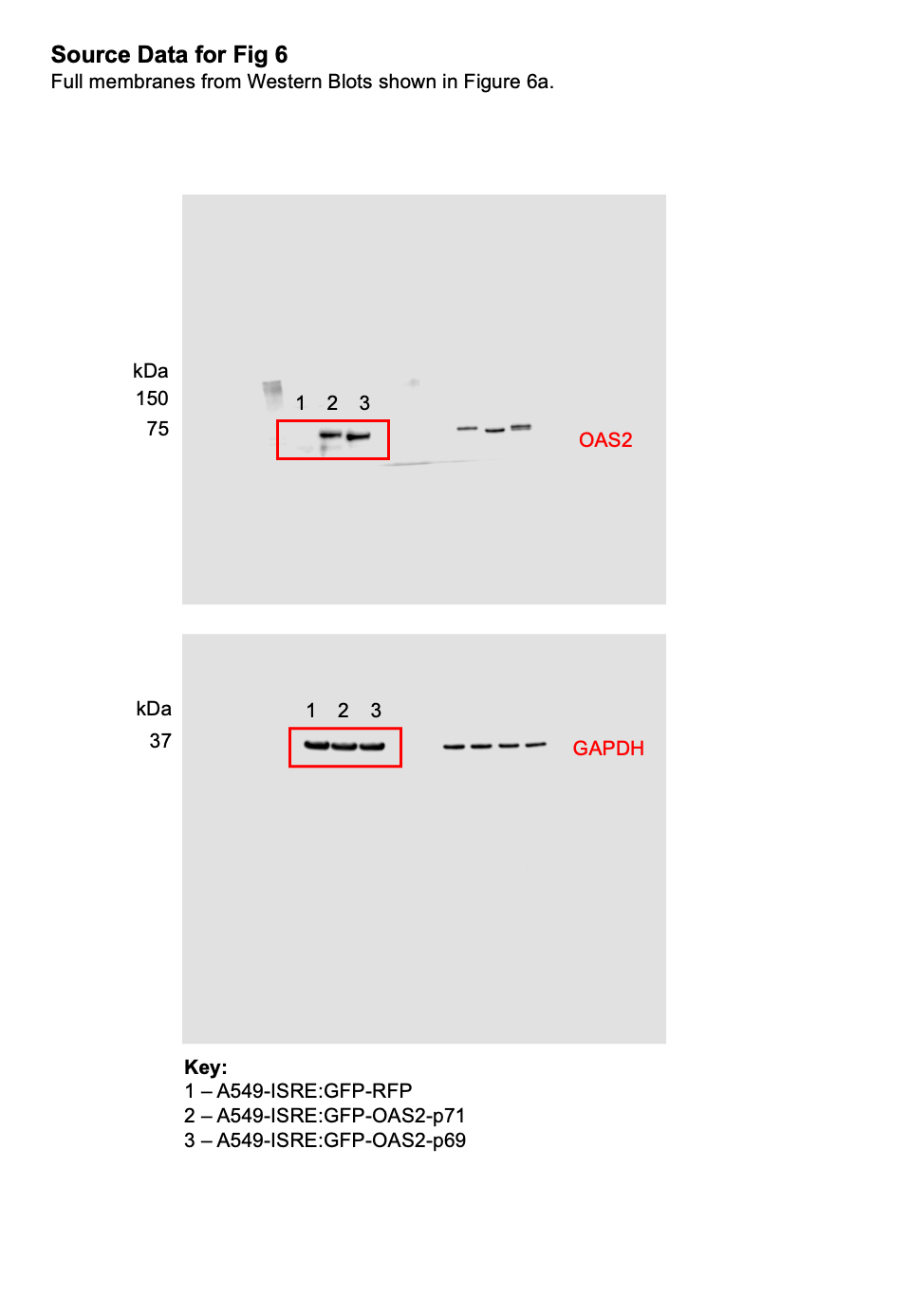

Supplement: Supplementary file 7 — Source data Fig. 6 [file 44318_2026_825_MOESM7_ESM.zip › Figure 6/6A/Figure_6A_WesternBlot.tiff]

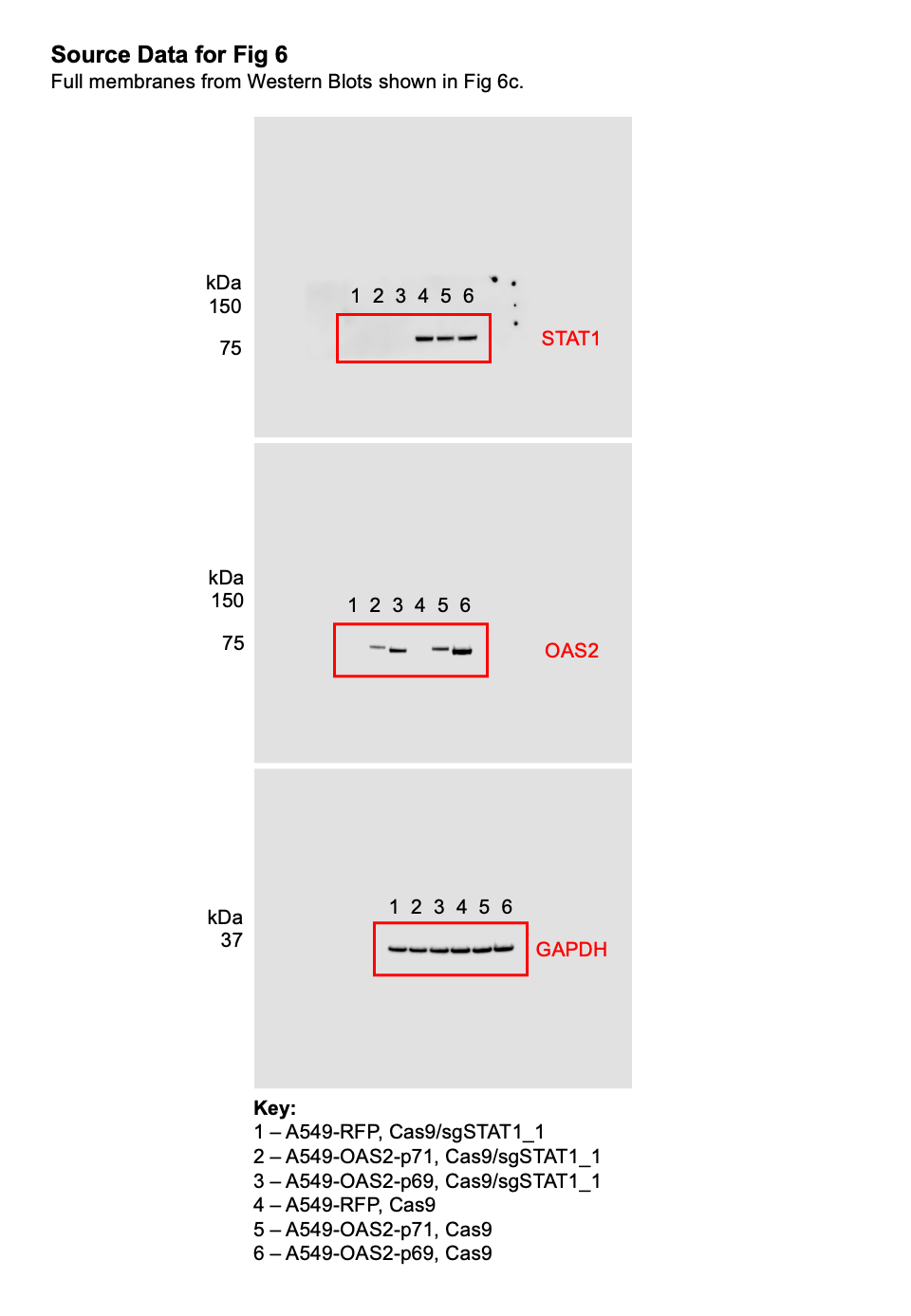

Supplement: Supplementary file 7 — Source data Fig. 6 [file 44318_2026_825_MOESM7_ESM.zip › Figure 6/6C/Figure_6C_WesternBlot.tiff]

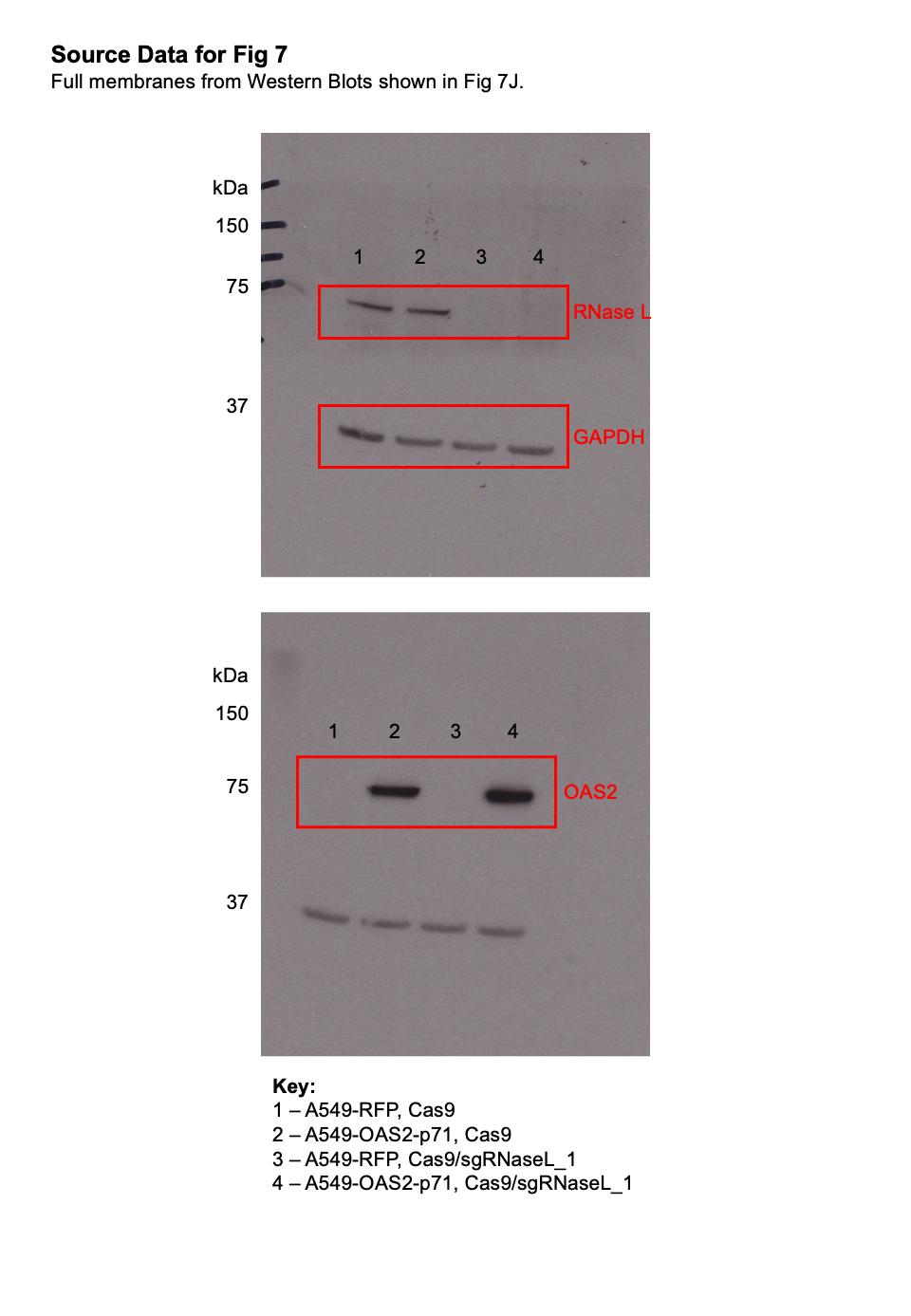

Supplement: Supplementary file 8 — Source data Fig. 7 [file 44318_2026_825_MOESM8_ESM.zip › Figure 7/7J/Figure_7J_WesternBlot.tiff]

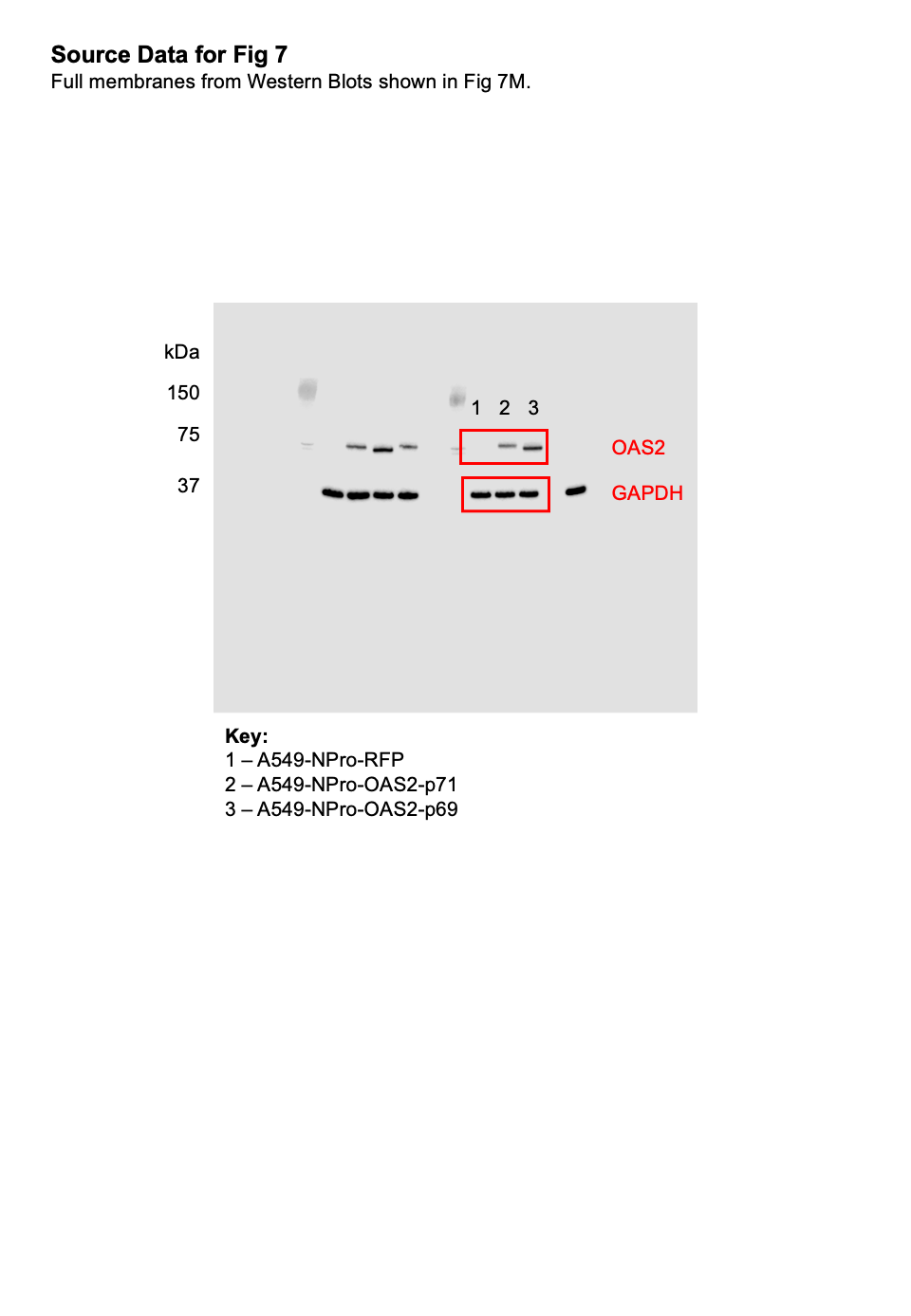

Supplement: Supplementary file 8 — Source data Fig. 7 [file 44318_2026_825_MOESM8_ESM.zip › Figure 7/7M/Figure_7M_WesternBlot.tiff]

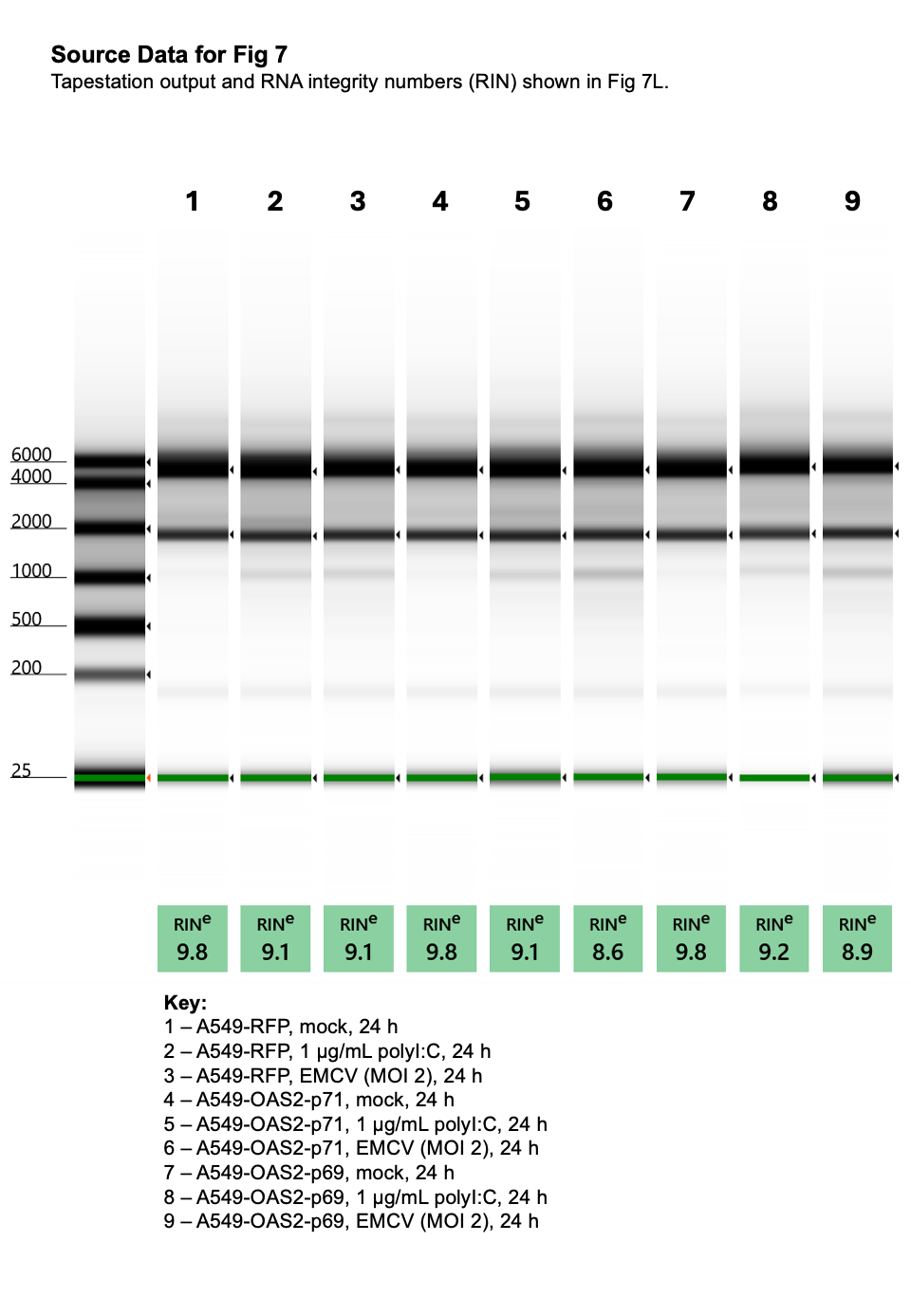

Supplement: Supplementary file 8 — Source data Fig. 7 [file 44318_2026_825_MOESM8_ESM.zip › Figure 7/7L/Figure_7L_TapestationImage.tiff]

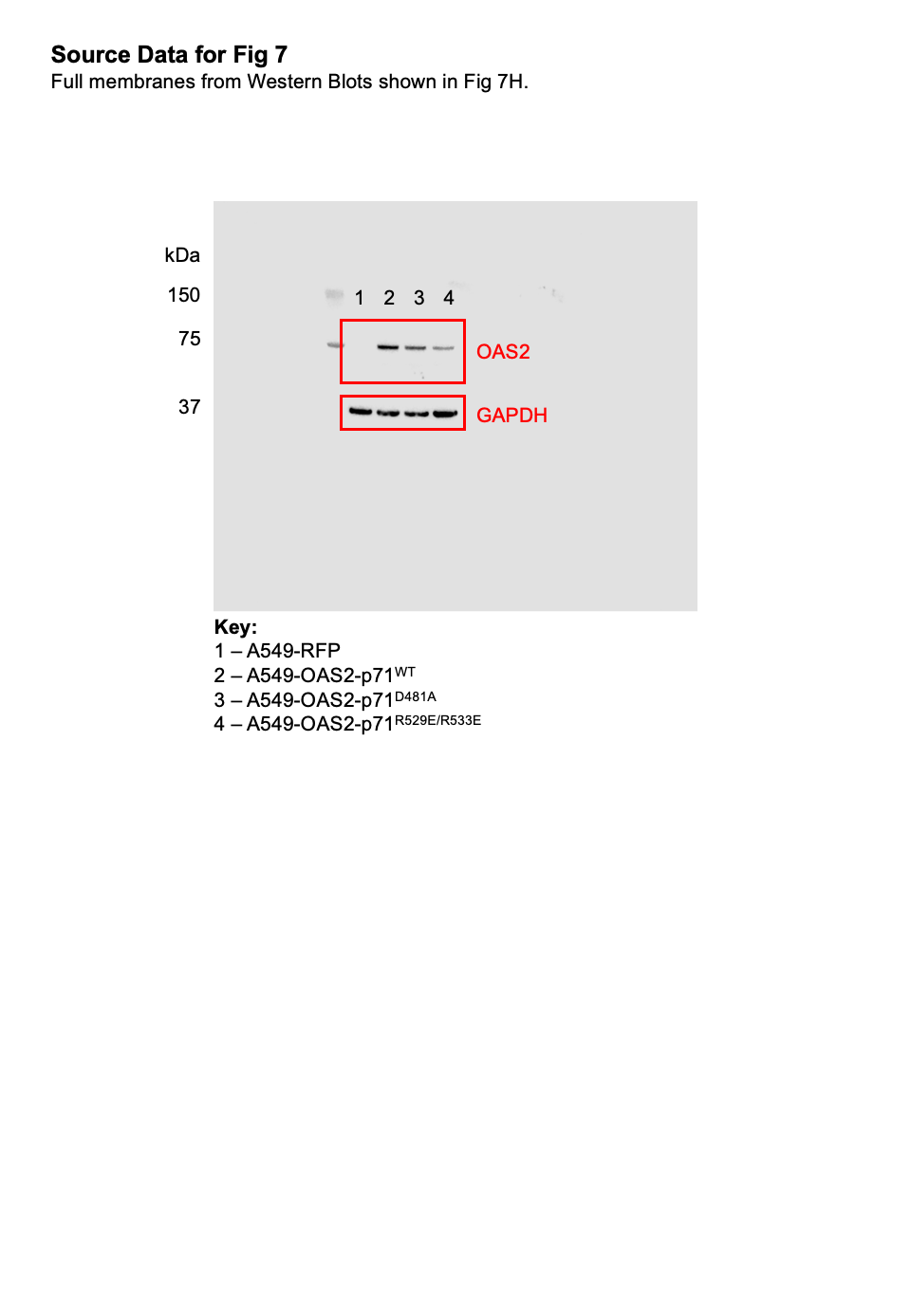

Supplement: Supplementary file 8 — Source data Fig. 7 [file 44318_2026_825_MOESM8_ESM.zip › Figure 7/7H/Figure_7H_WesternBlot.tiff]

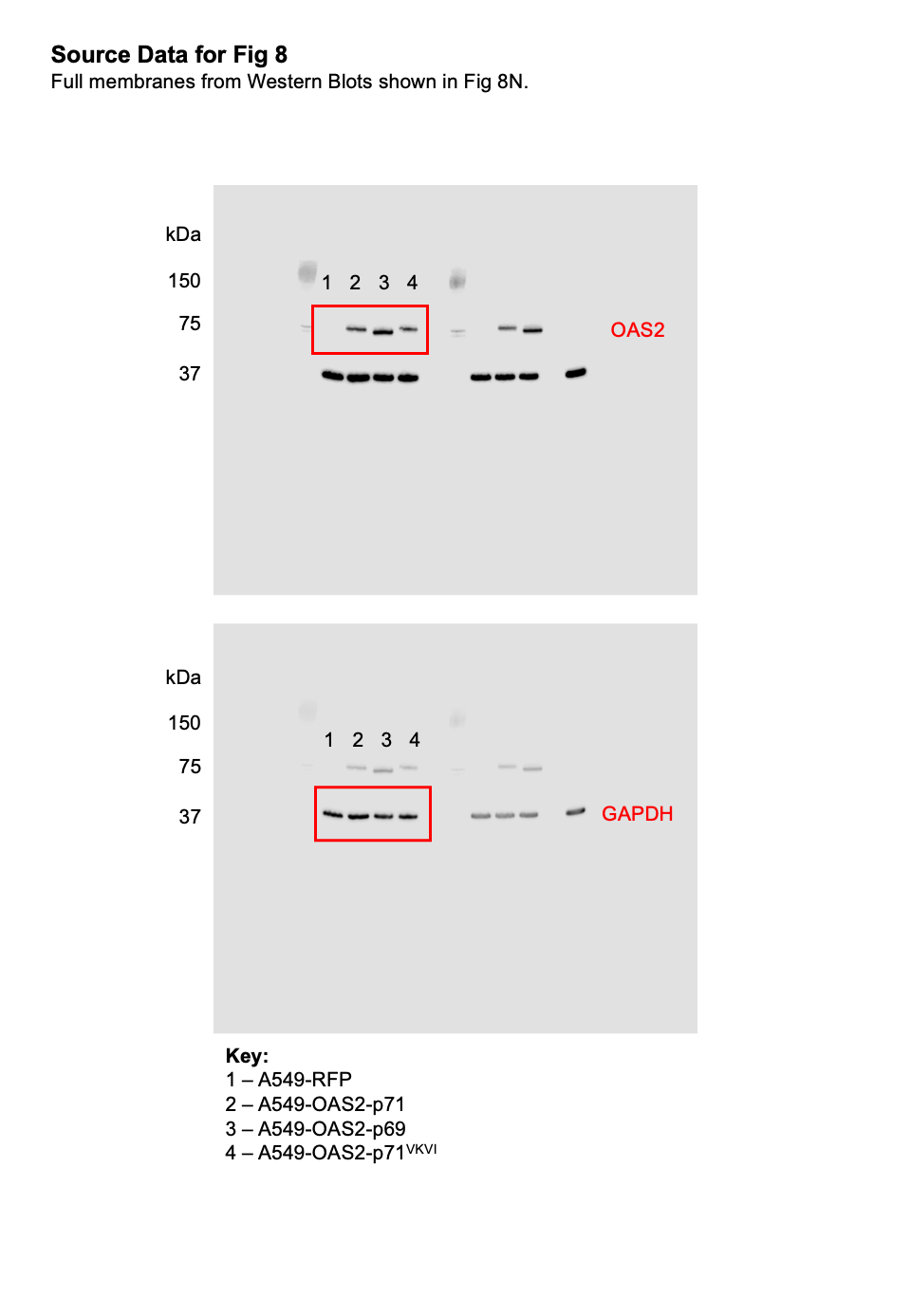

Supplement: Supplementary file 9 — Source data Fig. 8 [file 44318_2026_825_MOESM9_ESM.zip › Figure 8/8N/Figure_8N_WesternBlot.tiff]

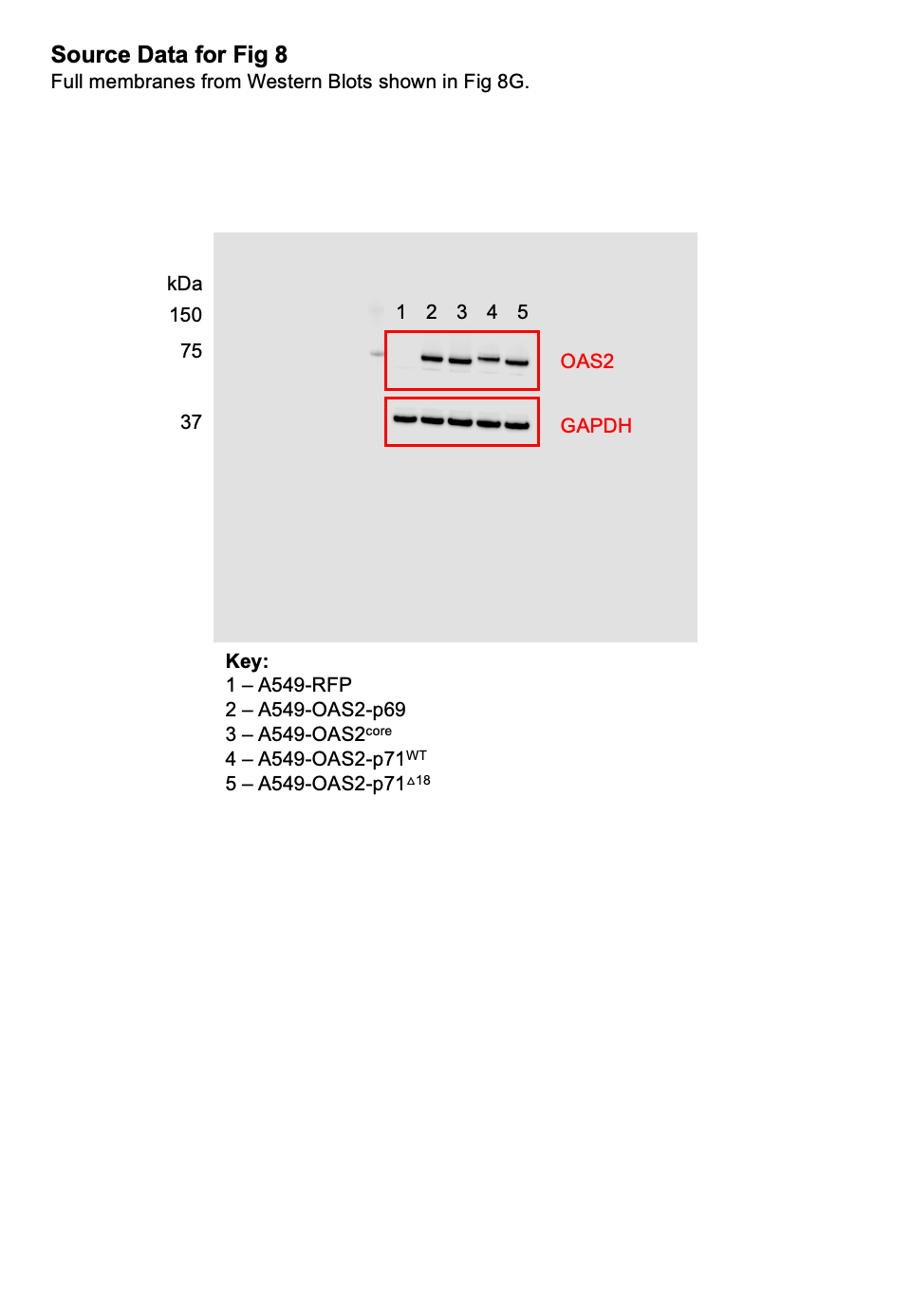

Supplement: Supplementary file 9 — Source data Fig. 8 [file 44318_2026_825_MOESM9_ESM.zip › Figure 8/8G/Figure_8G_WesternBlot.tiff]

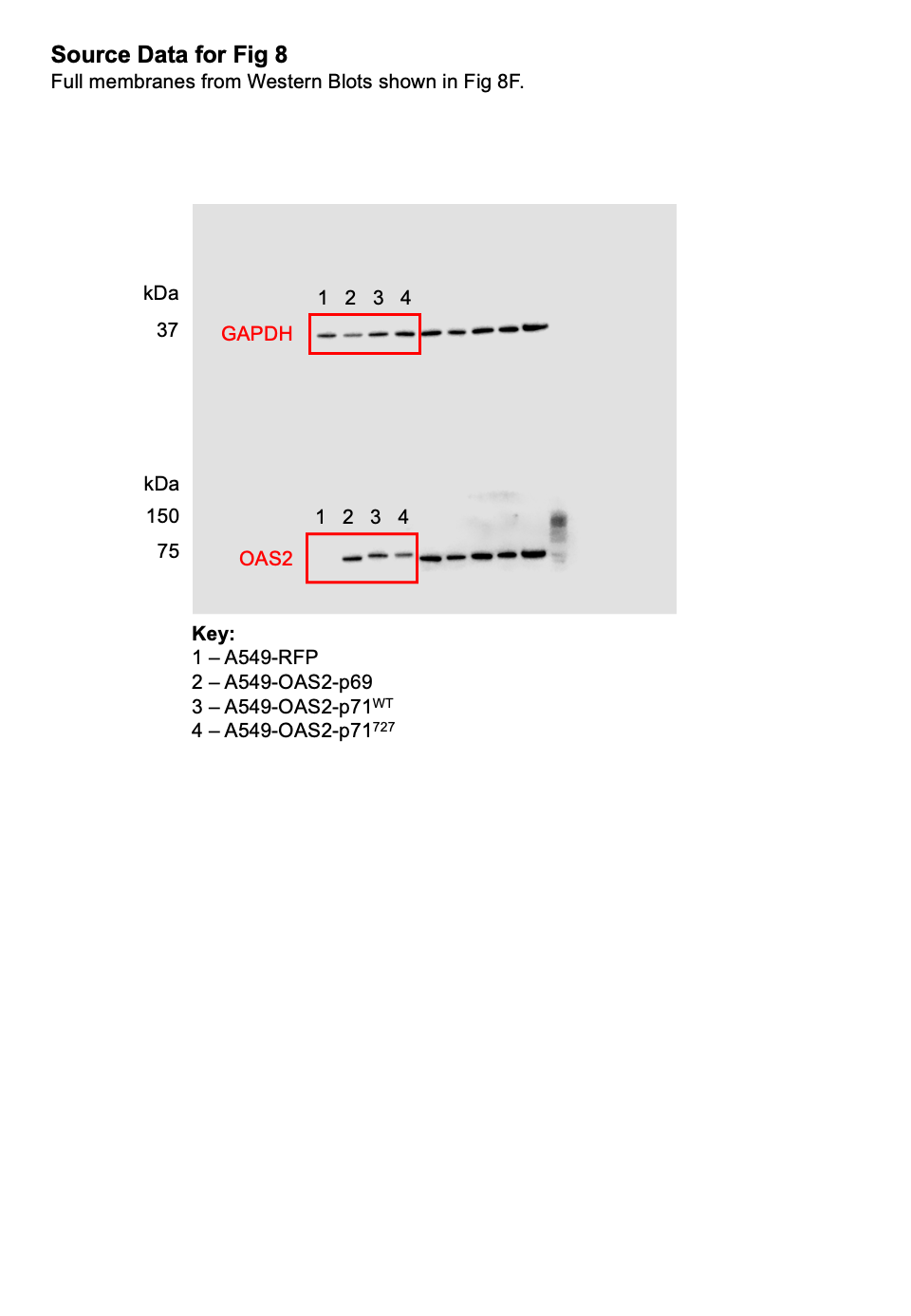

Supplement: Supplementary file 9 — Source data Fig. 8 [file 44318_2026_825_MOESM9_ESM.zip › Figure 8/8F/Figure_8F_WesternBlot.tiff]

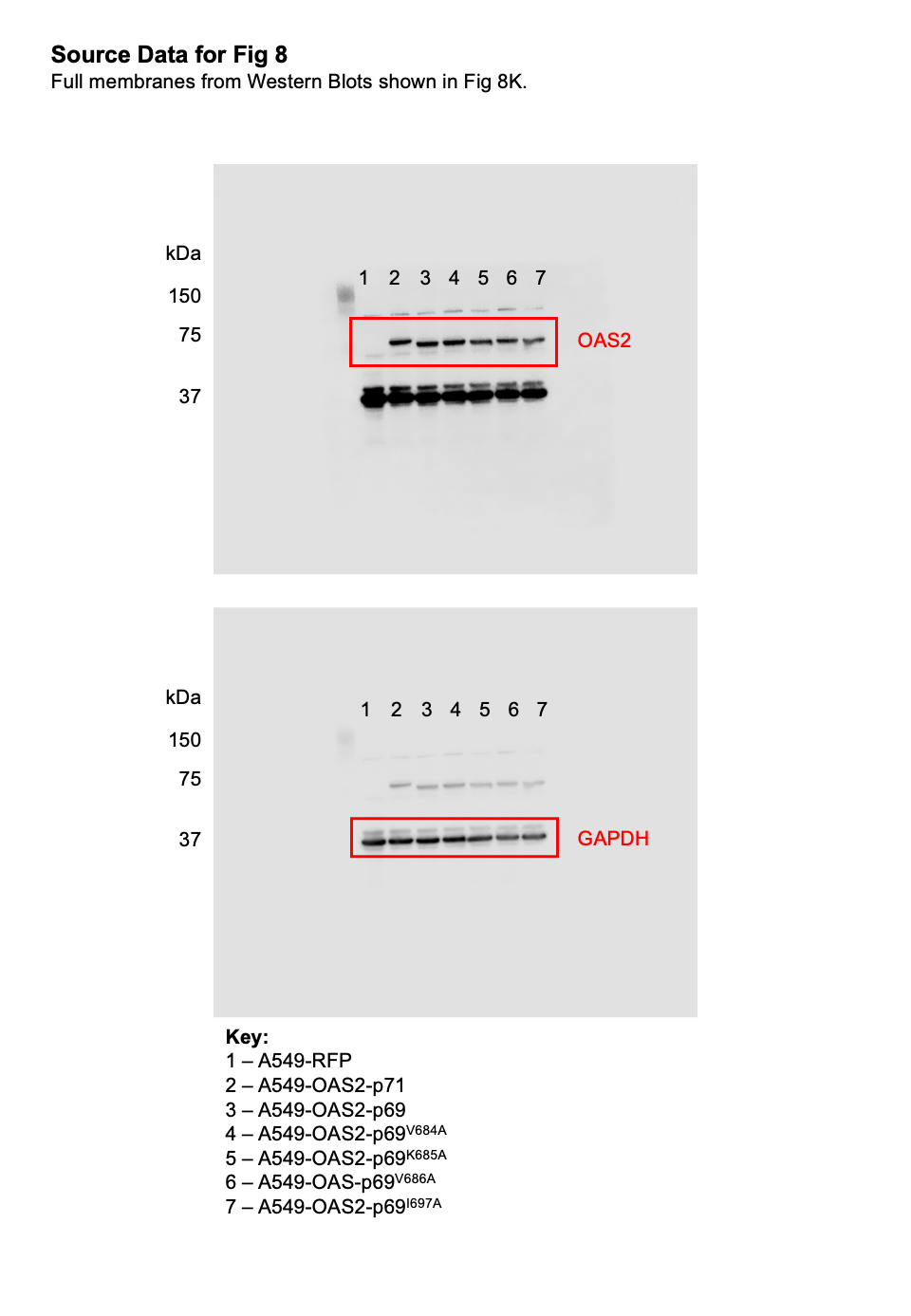

Supplement: Supplementary file 9 — Source data Fig. 8 [file 44318_2026_825_MOESM9_ESM.zip › Figure 8/8K/Figure_8K_WesternBlot.tiff]
