# Supplementary material for: Alternative splicing broadens antiviral diversity at the human OAS2 locus
Source: EMBO J. 2026 Jun 3;45(14):5164–91. doi: 10.1038/s44318-026-00825-w (PMC13372824; doi:10.1038/s44318-026-00825-w)
Supplement: Supplementary file 11 — Appendix [file 44318_2026_825_MOESM11_ESM.pdf]

Appendix for

Alternative splicing broadens antiviral diversity at the human *OAS2* locus

Emma L. Davies[1,*], Alegna Calderon Nuñez[1,†], Allison L. Ward[1,†], Hanna Sowar[1], Eilidh Rivers[1], Arda Balci[1], Daniel Mair[1], Elliot Moorhouse[1], Jake Towers[1], Arthur Wickenhagen[1], Matthew L. Turnbull[1], Massimo Palmarini[1], Sam J. Wilson[2,*], Adam J. Fletcher[1,*]

[1]MRC-University of Glasgow Centre for Virus Research, University of Glasgow, Glasgow, UK

[2]Cambridge Institute of Therapeutic Immunology and Infectious Disease (CITIID), University of Cambridge, Cambridge, UK

[†]These authors contributed equally

[*]Correspondence: emma.davies@glasgow.ac.uk (E.L.D.), sjw58@cam.ac.uk (S.J.W.), and adam.fletcher@glasgow.ac.uk (A.J.F.)

Table of Contents

Appendix Figure S1 - BVDV NPro degrades IRF3 and inhibits IFN signalling.....................2

Appendix Table S1- List of Oligonucleotides used in this study.......................................3

Appendix Table S2 – Exact p-values for main and extended view figures...........................4

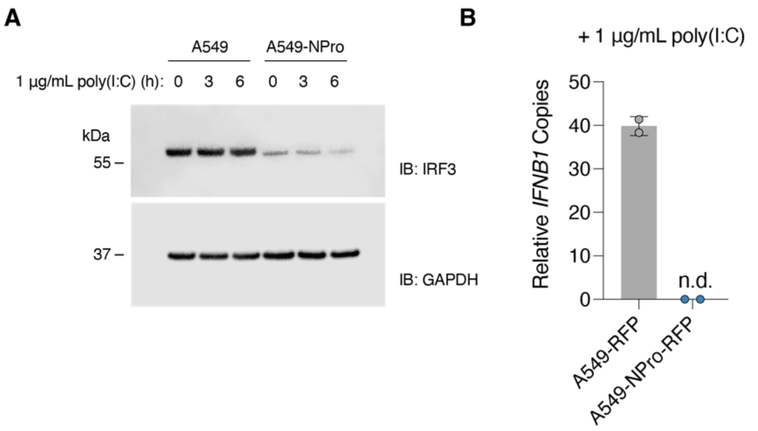

Appendix Figure S1 – BVDV NPro degrades IRF3 and inhibits IFN signalling.

(**A**) A549-RFP and A549-NPro-RFP were transfected with 1 µg/mL poly(I:C), and IRF3 levels were monitored at multiple timepoints, by Western blotting.

(**B**) A549-RFP and A549-NPro-RFP were transfected with 1 µg/mL poly(I:C) and IFNB1 transcript levels were measured at 6 h, by RT-qPCR. n.d., not detected.

Appendix Table S1 - List of oligonucleotides used in this study

Purpose	Target	Sequence
Mutagenesis	OAS2 D481A forward	CCTCAACGAAAGTGTCAGCTTTGCAGTGCTTCCTGCCTTTAATGCACTGGG
Mutagenesis	OAS2 D481A reverse	AAAGCTGACACTTTCGTTGAGGACTTTGG
Mutagenesis	OAS2 CAFAKA forward	GGAATGGTTATCCTCTCCCGCCGCCGCGGATGGGACTGGAAACCCAATACCACC
Mutagenesis	OAS2 CAFAKA reverse	GGGAGAGGATAACCATTCCTTTGCTTC
Mutagenesis	OAS2 R529E/R533E forward	CTGTTTCACAGTCCTGCAGGAAAACTTCATTGAATCCCGGCCCACCAAACTAAAGG
Mutagenesis	OAS2 R529E/R533E reverse	CTGCAGGACTGTGAAACAGGTAGAAAAC
Mutagenesis	OAS2 p71 727 forward	GCCTCAGACAGTGGTTCAAAG
Mutagenesis	OAS2 p71 727 reverse	CCGCCCTCGAGGAATTCGGCCAGAGAGGCCTTAAAAGCGATTGCCAGATGATCTCCAGAAGTTTCTTTTAGAATTATTATTCAG
Mutagenesis	OAS2 p71△18 forward	GCCTCAGACAGTGGTTCAAAG
Mutagenesis	OAS2 p71△18 reverse	CCGCCCTCGAGGAATTCGGCCAGAGAGGCCCTACTCATTGACAATAGGATGGATCCTAGC
Mutagenesis	OAS2 core forward	GCCTCAGACAGTGGTTCAAAG
Mutagenesis	OAS2 core reverse	CCGCCCTCGAGGAATTCGGCCAGAGAGGCCCTACGGCACTTTCCAAGGTGGTATTGGGTTTC
Mutagenesis	OAS2 p71 VKVI forward	CTGAATAATAATTCTAAAAGAAACTTCGTAAAAGTCATCTAGGGCCTCTCTGGCCGAATTCC
Mutagenesis	OAS2 p71 VKVI reverse	GAAGTTTCTTTTAGAATTATTATTCAGGATTCTATGGCTTCTG
Mutagenesis	OAS2 p69 V684A forward	GCCTCAGACAGTGGTTCAAAG
Mutagenesis	OAS2 p69 V684A reverse	CCGCCCTCGAGGAATTCGGCCAGAGAGGCCTTAGATGACTTTTGCCGGCACTTTCCAAGG
Mutagenesis	OAS2 p69 K685A forward	GCCTCAGACAGTGGTTCAAAG
Mutagenesis	OAS2 p69 K685A reverse	CCGCCCTCGAGGAATTCGGCCAGAGAGGCCTTAGATGACTGCTACCGGCACTTTCCAAGG
Mutagenesis	OAS2 p69 V686A forward	GCCTCAGACAGTGGTTCAAAG
Mutagenesis	OAS2 p69 V686A reverse	CCGCCCTCGAGGAATTCGGCCAGAGAGGCCTTAGATTGCTTTTACCGGCACTTTCCAAGG
Mutagenesis	OAS2 p69 I687A forward	GCCTCAGACAGTGGTTCAAAG
Mutagenesis	OAS2 p69 I687A reverse	CCGCCCTCGAGGAATTCGGCCAGAGAGGCCTTATGCGACTTTTACCGGCACTTTCCAAGG
OAS2 KO Generation	sgOAS2_1	GTCTTAAGAGGCAACTCCGA
OAS2 KO Generation	sgOAS2_2	GGACGGAAAACAGTCTTAAG
OAS2 KO Generation	sgOAS2_3	GCTTACTCAGAGCGTTGAAGG
RNase L KO Generation	sgRNaseL_1	GCCGAGTTGCTGTGCAAACG
RNase L KO Generation	sgRNaseL_2	TTATCCTCGCAGCGATTGCG
RNase L KO Generation	sgRNaseL_3	TATAGGACGCTTCGGAATGT
STAT1 KO Generation	sgSTAT1_1	GAGGTCATGAAAACGGATGG
RT-qPCR	HCoV-OC43 ORF1a forward	ATGTGGTGTAAAGCAGGAAC
RT-qPCR	HCoV-OC43 ORF1a reverse	GCAAGAACAGTCCACGGTATA
RT-qPCR	HCoV-OC43 ORF1a Probe	TACTGGTCTGGACGCTGTTATGC
RT-qPCR	HCoV-OC43 N forward	CTACTTCGCGCACATCCAG
RT-qPCR	HCoV-OC43 N reverse	GTCAGGTGTTACACCAGAGG
RT-qPCR	HCoV-OC43 N Probe	AGCCTCTAGTGCAGGATCGCGTAG

Appendix Table S2 - Exact P-values for main and expanded view figures

Figure	Test	Group Compared	P-value	Summary
2D	Welch's t-test	RFP vs. p71 24 h	0.9329	ns
		RFP vs. p69 24 h	0.3427	ns
		RFP vs. p71 48 h	0.8118	ns
		RFP vs. p69 48 h	0.0066	**
		RFP vs. p71 72 h	0.5057	ns
		RFP vs. p69 72 h	<0.0001	****
2E	One-way ANOVA with Dunnett's test	RFP vs. p71	0.6743	ns
		RFP vs. p69	<0.0001	****
2F	One-way ANOVA with Dunnett's test	RFP vs. p71	0.9494	ns
		RFP vs. p69	0.0193	*
2G	One-way ANOVA with Dunnett's test	RFP vs. p71	0.9631	ns
		RFP vs. p69	0.0060	**
2J	Welch's t-test	RFP vs. p71 24 h	0.1543	ns
		RFP vs. p69 24 h	0.0352	*
		p71 vs. p69 24 h	0.5696	ns
		RFP vs. p71 48 h	0.2028	ns
		RFP vs. p69 48 h	0.0364	*
		p71 vs. p69 48 h	0.0137	*
		RFP vs. p71 72 h	0.1099	ns
		RFP vs. p69 72 h	0.0035	**
		p71 vs. p69 48 h	0.0081	ns
2L	Welch's t-test	Cas9/sgOAS1_1 vs Cas9 24 h	0.4617	ns
		Cas9/sgOAS1_1 vs Cas9 48 h	0.5701	ns
		Cas9/sgOAS1_1 vs Cas9 72 h	0.04474	*
2N	Welch's t-test	Cas9/sgOAS1_1 vs Cas9 24 h	0.189	ns
		Cas9/sgOAS1_1 vs Cas9 48 h	0.0924	ns
		Cas9/sgOAS1_1 vs Cas9 72 h	0.0508	ns
3C	One-way ANOVA with Dunnett's test	Untreated vs. p71	<0.0001	****
		Untreated vs. p69	<0.0001	****
3E	One-way ANOVA with Dunnett's test	RFP vs. p71^{WT}	0.9162	ns
		RFP vs. p71^{G2A}	0.9902	ns
		RFP vs. p69^{WT}	<0.0001	****
		RFP vs. p69^{G2A}	>0.9999	ns
EV3C	One-way ANOVA with Dunnett's test	Untreated vs. p71	0.0008	***
		Untreated vs. p69	0.1045	ns
4F	One-way ANOVA with Dunnett's test	RFP vs. p69^{WT}	0.0096	**
		RFP vs. p69$^{R529E/R533E}$	0.5055	ns
4G	One-way ANOVA with Dunnett's test	RFP vs. p69^{WT}	0.0086	**
		RFP vs. p69$^{R529E/R533E}$	0.6653	ns
5B	One-way ANOVA with Dunnett's test	RFP vs. p69^{WT}	<0.0001	****
		RFP vs. p69^{D481A}	<0.0001	****
		RFP vs. p69^{CAFAKA}	<0.0001	****
5C	One-way ANOVA with Dunnett's test	RFP vs. p69^{WT}	<0.0001	****
		RFP vs. p69^{D481A}	<0.0001	****
		RFP vs. p69^{CAFAKA}	<0.0001	****
6D	One-way ANOVA with Tukey's test	Cas9 + RFP vs. Cas9 + p71	>0.9999	ns
		Cas9 + RFP vs. Cas9 + p69	0.0129	*
		Cas9 + RFP vs. Cas9/sgSTAT1 + RFP	0.9991	ns
		Cas9/RFP vs. Cas9/sgSTAT1 + p71	>0.9999	ns
		Cas9 + RFP vs. Cas9/sgSTAT1 + p69	0.0045	**
		Cas9 + p71 vs. Cas9 + p69	0.0128	*
		Cas9 + p71 vs. Cas9/sgSTAT1 + RFP	0.9991	ns
		Cas9 + p71 vs. Cas9/sgSTAT1 + p71	>0.9999	ns
		Cas9 + p71 vs. Cas9/sgSTAT1 + p69	0.0045	**
		Cas9 + p69 vs. Cas9/sgSTAT1 + RFP	0.0051	**
		Cas9 + p69 vs. Cas9/sgSTAT1 + p71	0.0210	*
		Cas9 + p69 vs. Cas9/sgSTAT1 + p69	0.9984	ns
		Cas9/sgSTAT1 + RFP vs. Cas9/sgSTAT1 + p71	0.9930	ns
		Cas9/sgSTAT1 + RFP vs. Cas9/sgSTAT1 + p69	0.0017	**
		Cas9/sgSTAT1 + p71 vs Cas9/sgSTAT1 + p69	0.0075	**

7A	Welch's t-test	RFP vs. p71 8h	0.6210	ns
		RFP vs. p69 8 h	0.5567	ns
		RFP vs. p71 24 h	0.0132	*
		RFP vs. p69 24 h	0.1501	ns
		RFP vs. p71 48 h	0.0631	ns
		RFP vs. p71 48 h	0.5168	ns
7B	Welch's t-test	RFP vs. p71 8 h	0.2937	ns
		RFP vs. p69 8 h	0.3544	ns
		p71 vs. p69 8 h	0.8631	ns
		RFP vs. p71 24 h	0.0302	*
		RFP vs. p69 24 h	0.061	ns
		p71 vs. p69 24 h	0.1443	ns
		RFP vs. p71 48 h	0.0165	*
		RFP vs. p69 48 h	0.01	**
		p71 vs. p69 48 h	0.4419	ns
7D	Welch's t-test	Cas9/sgOAS1_1 vs Cas9 8 h	0.3173	ns
		Cas9/sgOAS1_1 vs Cas9 24 h	0.3255	ns
		Cas9/sgOAS1_1 vs Cas9 48 h	0.8079	ns
7F	Welch's t-test	Cas9/sgOAS1_1 vs Cas9 8 h	0.4141	ns
		Cas9/sgOAS1_1 vs Cas9 24 h	0.1542	ns
		Cas9/sgOAS1_1 vs Cas9 48 h	0.0911	ns
7G	One-way ANOVA with Dunnett's test	RFP vs. p71	0.0030	**
		RFP vs. $p71^{G2A}$	0.9370	ns
		RFP vs. p69	0.7989	ns
		RFP vs. $p69^{G2A}$	0.9963	ns
7I	One-way ANOVA with Dunnett's test	RFP vs. p71	0.0164	*
		RFP vs. $p71^{D481A}$	0.9621	ns
		RFP vs. $p71^{R529E/R533E}$	0.8377	ns
7K	One-way ANOVA with Tukey's test	Cas9 + RFP vs. Cas9 + p71	<0.0001	****
		Cas9 + RFP vs. Cas9/sgRNaseL + RFP	0.0138	*
		Cas9 + RFP vs. Cas9/sgRNaseL + p71	0.0109	*
		Cas9 + p71 vs. Cas9/sgRNaseL + RFP	<0.0001	****
		Cas9 + p71 vs. Cas9/sgRNaseL + p71	<0.0001	****
		Cas9/sgRNaseL + RFP vs. Cas9/sgRNaseL + p71	0.9979	ns
7N	One-way ANOVA with Dunnett's test	$NPro^{RFP}$ vs. $NPro^{p71}$	0.0026	**
		$NPro^{RFP}$ vs. $NPro^{p69}$	0.5758	ns
8H	One-way ANOVA with Dunnett's test	RFP vs. $p71^{727}$	0.7651	ns
		RFP vs. p71	0.8156	ns
		RFP vs. $p71^{\Delta 18}$	0.8733	ns
		RFP vs. p69	0.0004	***
		RFP vs. $OAS2^{core}$	>0.9999	ns
8I	One-way ANOVA with Dunnett's test	RFP vs. $p71^{727}$	0.227	ns
		RFP vs. p71	0.0003	***
		RFP vs. $p71^{\Delta 18}$	0.0281	*
		RFP vs. p69	0.3617	ns
		RFP vs. $OAS2^{core}$	0.4091	ns
8L	One-way ANOVA with Dunnett's test	RFP vs. p71	0.9998	ns
		RFP vs. p69	0.0429	*
		RFP vs. $p69^{V684A}$	0.0307	*
		RFP vs. $p69^{K685A}$	0.2151	ns
		RFP vs. $p69^{V686A}$	>0.9999	ns
		RFP vs. $p69^{I687A}$	0.9979	ns
8O	One-way ANOVA with Dunnett's test	RFP vs. p71	0.9615	ns
		RFP vs. $p71^{VKVI}$	0.9945	ns
		RFP vs. p69	0.0527	ns
EV5F	One-way ANOVA with Dunnett's test	RFP/RFP vs. p71/RFP	0.3476	ns
		RFP/RFP vs. p69/RFP	0.0005	***
		RFP/RFP vs. p71/p69	0.3807	ns
EV5G	One-way ANOVA with Dunnett's test	RFP/RFP vs. p71/RFP	<0.0001	****
		RFP/RFP vs. p69/RFP	0.7944	ns
		RFP/RFP vs. p71/p69	<0.0001	****
