# Supplementary material for: Alternative splicing broadens antiviral diversity at the human OAS2 locus
Source: EMBO J. 2026 Jun 3;45(14):5164–91. doi: 10.1038/s44318-026-00825-w (PMC13372824; doi:10.1038/s44318-026-00825-w)
Supplement: Supplementary file 12 — Expanded View Figures [file 44318_2026_825_MOESM12_ESM.pdf]

# Expanded View Figures

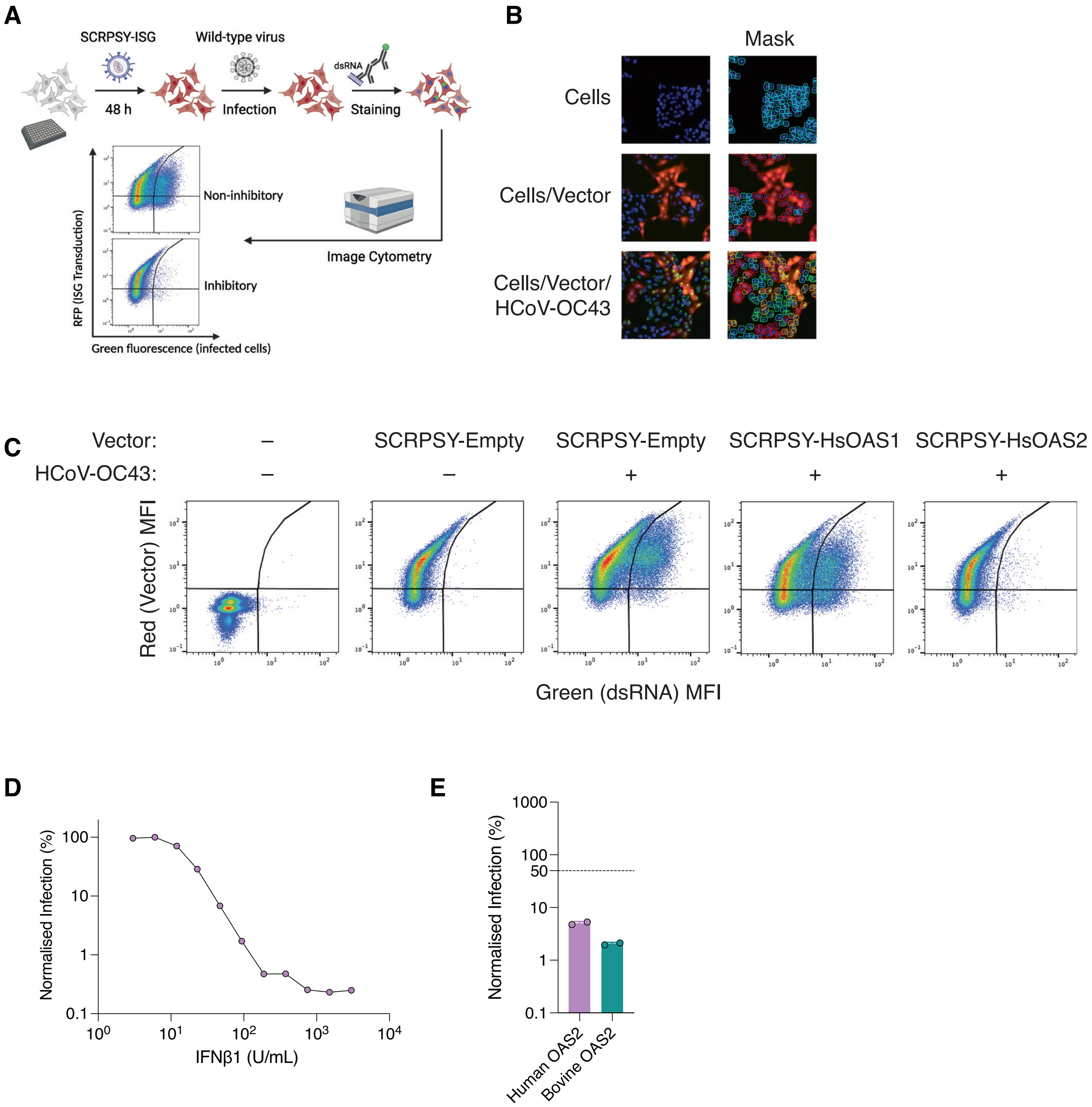


**Figure EV1. Quantifying RNA virus infection by immunostaining.**

(**A**) Schematic diagram of the screening method used for the arrayed ISG expression screens described in (Fig. 1D–G). (**B**) Representative images of control wells in the ISG screen (Fig. 1D) were gated for RFP and dsRNA expression levels using an image cytometer. (**C**) Cell populations from (Fig. 1D) were gated using cell-only and SCRPSY-Empty mock-infected controls. Representative FlowJo analysis plots are shown, including OAS1 and OAS2 as examples of a non-candidate and candidate ISG, respectively. MFI, mean fluorescence intensity. (**D**) A549 cells were pre-treated with IFNβ1 for 24 h, infected with HCoV-OC43 for 72 h, and stained for dsRNA prior to quantifying dsRNA+ cells by image cytometry. (**E**) A549 expressing Human or Bovine OAS2, from the ISG libraries in (Fig. 1D), were infected with HCoV-OC43 for 72 h and stained for dsRNA prior to quantifying dsRNA+ cells by image cytometry. Data normalised to SCRPSY-EMPTY controls.

 © The Author(s)

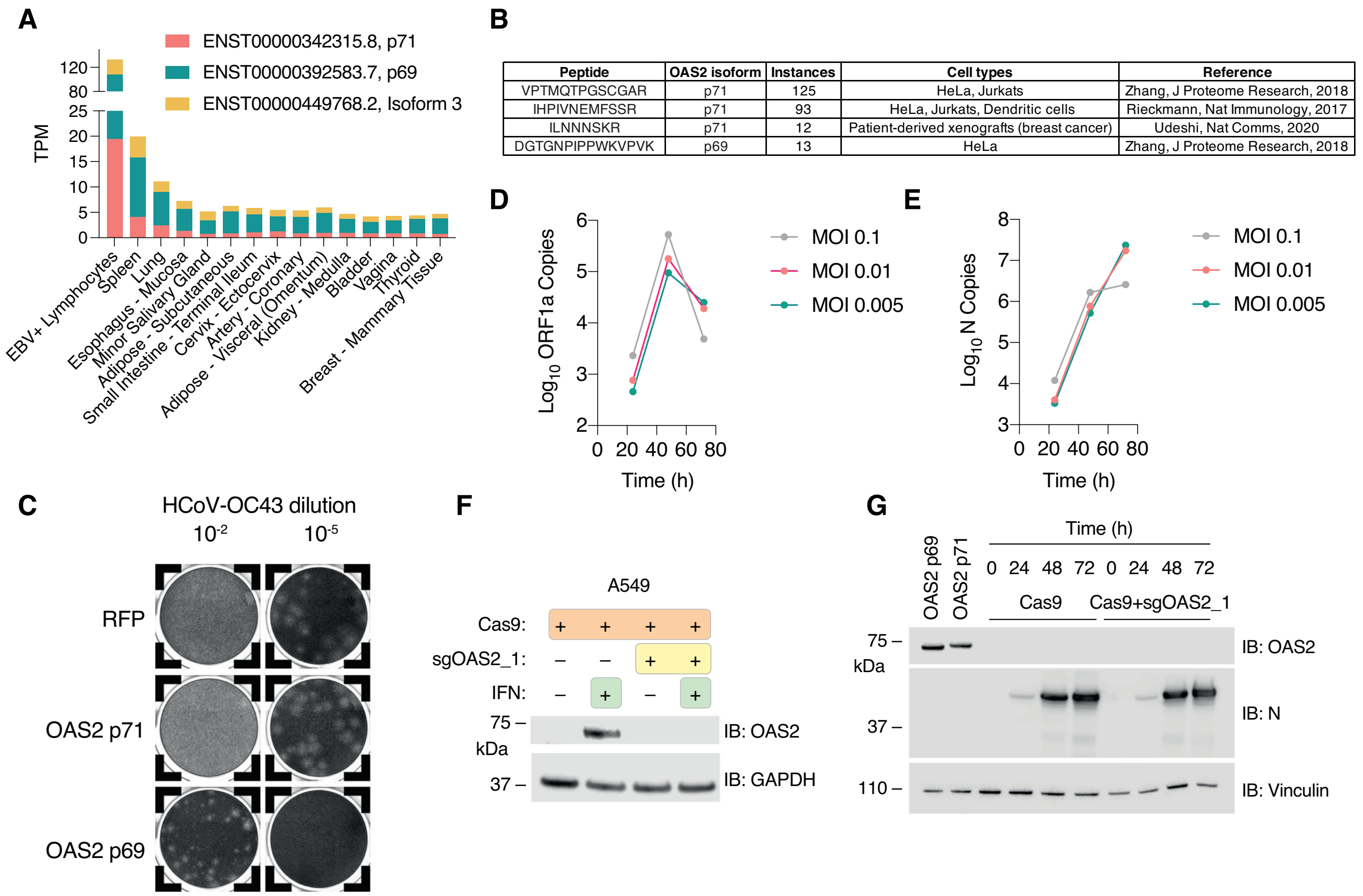


| Peptide | OAS2 isoform | Instances | Cell types | Reference |
|---|---|---|---|---|
| VPTMQTPGSCGAR | p71 | 125 | HeLa, Jurkats | Zhang, J Proteome Research, 2018 |
| IHPIVNEMFSSR | p71 | 93 | HeLa, Jurkats, Dendritic cells | Rieckmann, Nat Immunology, 2017 |
| ILNNNSKR | p71 | 12 | Patient-derived xenografts (breast cancer) | Udeshi, Nat Comms, 2020 |
| DGTGNPIPPWKVPVK | p69 | 13 | HeLa | Zhang, J Proteome Research, 2018 |

**Figure EV2. Presence of endogenous OAS2 isoforms.**

(**A**) Transcript expression of OAS2 isoforms across tissues analysed using the GTEx database. (**B**) The human peptide atlas (https://peptideatlas.org/) was searched for peptides from the OAS2 p71 and p69 C-terminus, generated by trypsin cleavage. (**C**) Representative images of HCoV-OC43 plaques formed in cell lines characterised in (Fig. 2C), 120 hpi. (**D**) HCoV-OC43 *ORF1a* transcript levels in A549 cells infected with HCoV-OC43 were quantified at multiple timepoints, by RT-qPCR. (**E**) HCoV-OC43 *nucleocapsid* transcript levels in A549 cells infected with HCoV-OC43 were quantified at multiple timepoints, by RT-qPCR. (**F**) Endogenous OAS2 levels in A549 cells transduced with a lentiviral vector-derived OAS2 sgRNA, with or without pre-treatment of 100 U/mL IFNβ for 24 h. (**G**) Expression of endogenous OAS2 was monitored in A549 cells from (**F**), infected with HCoV-OC43 (MOI 0.01), at multiple timepoints, by Western blotting.

© The Author(s)

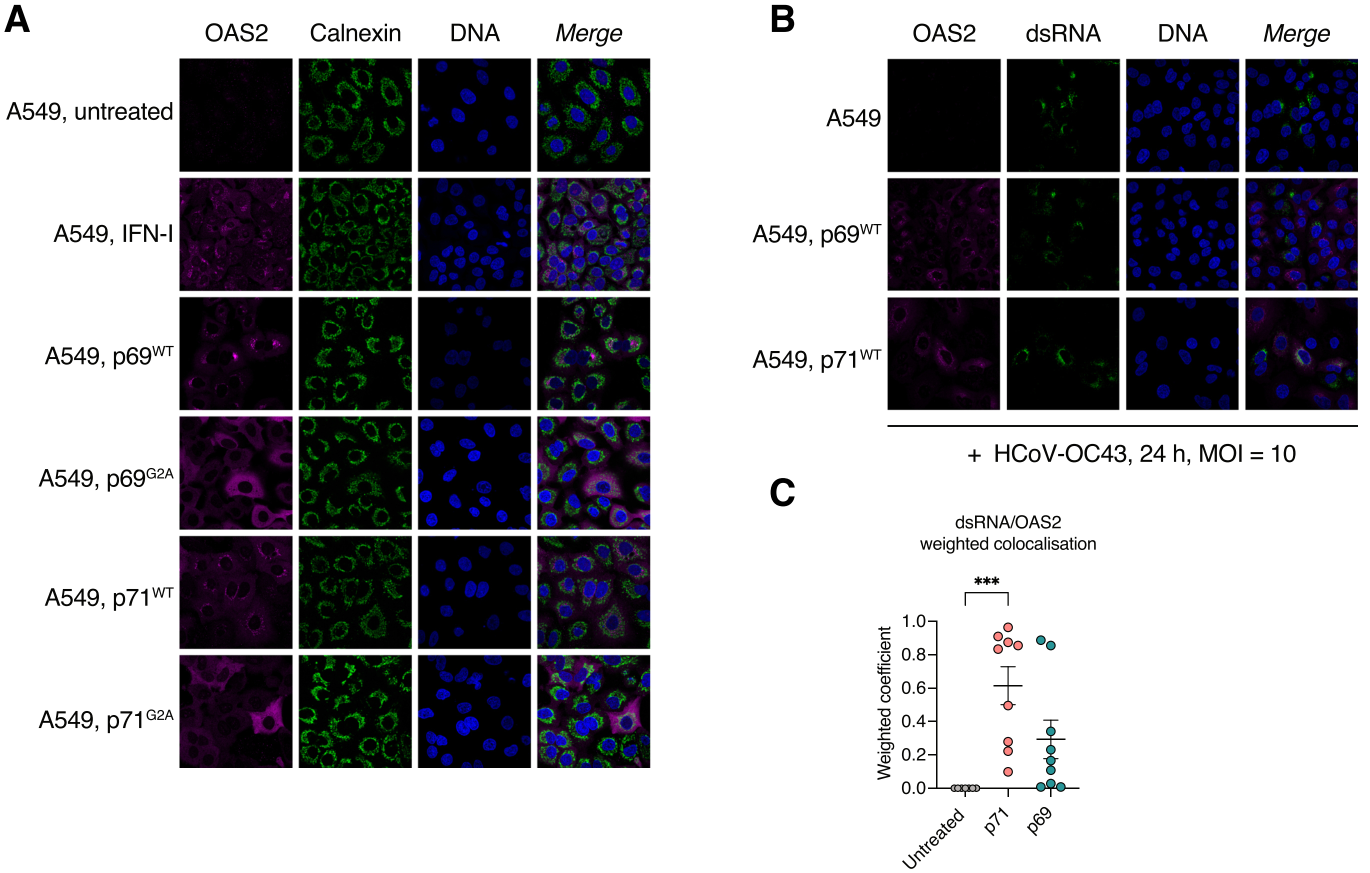


**Figure EV3. N-terminal myristoylation activity is required for antiviral activity.**

(**A**) A549 cells stimulated with 1000 U/mL IFNβ or A549 cells expressing p71, p69, $p71^{G2A}$ or $p69^{G2A}$, were immunostained for OAS2 (magenta), calnexin (green) and DNA (blue). (**B**) Representative immunofluorescence images of A549 cells expressing p71 or p69 infected with HCoV-OC43 (MOI = 10) for 24 h and immunostained for OAS2 (magenta), dsRNA (green) and DNA (blue). (**C**) Weighted coefficients between p71 and p69 isoforms and dsRNA. Each data point represents a separate region of interest from a representative experiment, $n = 2$. Data were presented as the mean ± SEM and analysed by one-way ANOVA with Dunnett's multiple comparison test, $***p = 0.0008$.

 © The Author(s)

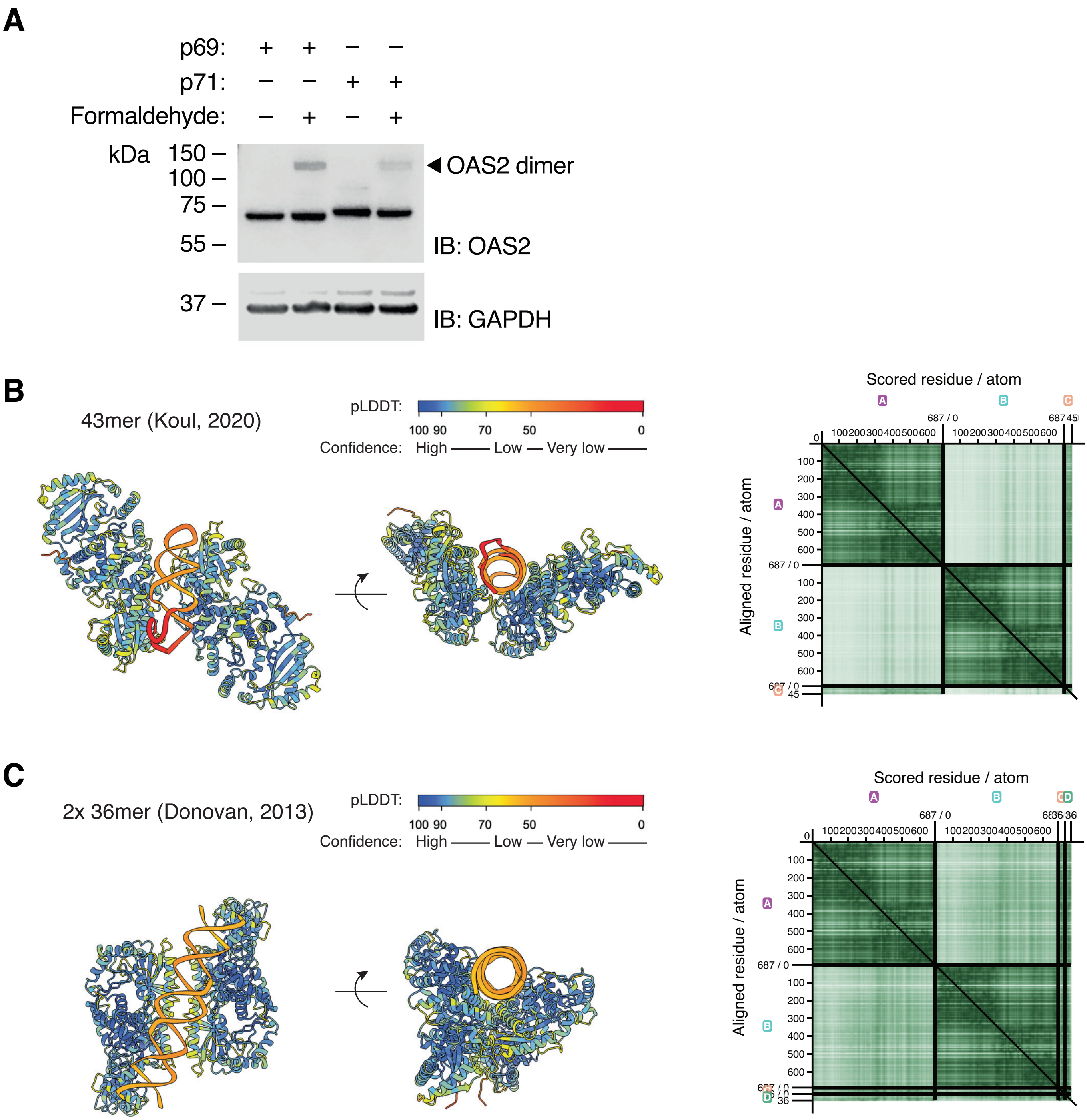


**Figure EV4. AlphaFold 3 models of OAS2:dsRNA.**

(**A**) A549 cells expressing OAS2 p71 or p69, treated with or without 0.5% formaldehyde. OAS2 expression assessed by Western blotting. (**B**, **C**) Top-ranking AlphaFold3 predictions of human OAS2 p69 in complex with dsRNA 43mer palindromic RNA (Koul et al, 2020a) (**B**) or 2x36mer RNA (Donovan et al, 2013) (**C**), coloured according to pLDDT scores as indicated by key. Predicted Aligned Error (PAE) plots for the top-ranking structures are shown, which indicate high confidence in the conformation of individual OAS2 structures, and lower confidence in the relative position of one OAS2 chain to the other.

© The Author(s) 

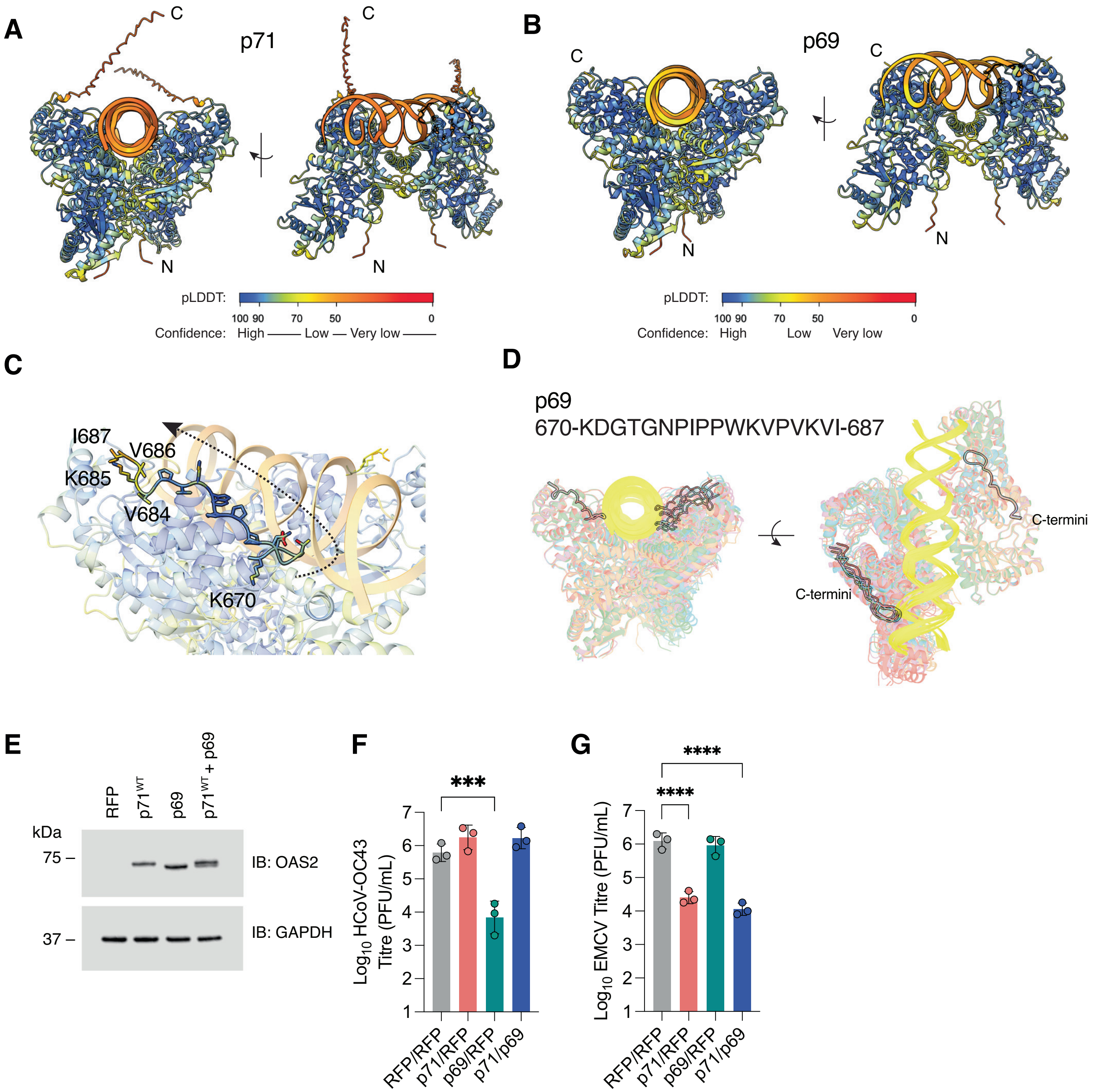


**Figure EV5. OAS2 C-terminal tails shape antiviral activity.**

(**A**, **B**) AlphaFold3 top-ranked models from (Fig. EV4) of p71 (**A**) or p69 (**B**) with dsRNA (Donovan et al, 2013) coloured by pLDDT score, indicated in colour key. OAS2 N- and C-termini are labelled N and C, respectively. (**C**) Close up of OAS2 p69 from structure presented in (**B**), showing side chains of peptide beginning at K670, until terminal residue I687, coloured as in (**B**). The dashed arrow indicates the sequence direction from 670-687. pLDDT scores are high between residues 670-683, and low between residues 684–687. (**D**) Superposition of all 5 OAS2 p69 AlphaFold3 structure predictions in complex with dsRNA (Donovan et al, 2013). The highlighted tail peptides begin at residues K670, as indicated; note that sequence K670-P683 is shared between p69 and p71 isoforms, thus the region highlighted for p69 is also partly present in p71. (**E**) A549 were modified to co-express both p71$^{WT}$ and p69 isoforms. Single isoforms were co-expressed with RFP to control for transgene dosage. OAS2 expression was confirmed by Western blotting. (**F**) Infectious titre of HCoV-OC43 in cells characterised in (**E**), determined by plaque assay at 120 hpi. (**G**) Infectious titre of EMCV in cells characterised in (**E**), determined by plaque assay at 30 hpi. Data information: Data were presented as the mean ± SD. (**F**, **G**) Data were analysed by one-way ANOVA with Dunnett's multiple comparison test (vs. RFP), where $*p < 0.05$, $**p < 0.01$, $***p < 0.001$ and $****p < 0.0001$. (**F**) $***p = 0.0005$. (**G**) $****p < 0.0001$.

 © The Author(s)
