# Supplementary material for: Alternative splicing broadens antiviral diversity at the human OAS2 locus
Source: EMBO J. 2026 Jun 3;45(14):5164–91. doi: 10.1038/s44318-026-00825-w (PMC13372824; doi:10.1038/s44318-026-00825-w)
Supplement: Supplementary file 1 — Peer Review File [file 44318_2026_825_MOESM1_ESM.pdf]

THE EMBO JOURNAL

Alternative splicing broadens antiviral diversity at the human OAS2 locus

Emma Davies, Alegna Calderon Nuñez, Allison Ward, Hanna Sowar, Eilidh Rivers, Arda Balci, Daniel Mair, Elliot Moorhouse, Jake Towers, Arthur Wickenhagen, Matthew Turnbull, Massimo Palmarini, Sam Wilson, and Adam Fletcher

Corresponding author(s): Adam Fletcher (adam.fletcher@glasgow.ac.uk) , Sam Wilson (sjw58@cam.ac.uk), Emma Davies (emma.davies@glasgow.ac.uk)

Review Timeline:

Submission Date:	20th Feb 25
Editorial Decision:	29th Apr 25
Revision Received:	6th Nov 25
Editorial Decision:	5th Feb 26
Appeal Received:	11th Feb 26
Editorial Decision:	7th Apr 26
Revision Received:	17th Apr 26
Accepted:	11th May 26

Editor: Cornelius Schneider

Transaction Report:

(Note: With the exception of the correction of typographical or spelling errors that could be a source of ambiguity, letters and reports are not edited. Depending on transfer agreements, referee reports obtained elsewhere may or may not be included in this compilation. Referee reports are anonymous unless the Referee chooses to sign their reports.)

Dear Dr. Fletcher,

Thank you for submitting your manuscript for consideration by the EMBO Journal. It has now been seen by three referees whose comments are shown below.

As you can see from the reports the referees think that the findings reported in this manuscript are interesting but also voice multiple major concerns regarding the experimental quality and think that extensive revisions are required. Given your availability to fully address the concerns raised by all three referees we invite you to submit a revised version of the manuscript. I should add that it is EMBO Journal policy to allow only a single round of revision, and acceptance of your manuscript will therefore depend on the completeness of your responses in this revised version.

When preparing your letter of response to the referees' comments, please bear in mind that this will form part of the Review Process File, and will therefore be available online to the community. For more details on our Transparent Editorial Process, please visit our website: https://www.embopress.org/page/journal/14602075/authorguide#transparentprocess

We generally allow three months as standard revision time. As a matter of policy, competing manuscripts published during this period will not negatively impact on our assessment of the conceptual advance presented by your study. However, we request that you contact the editor as soon as possible upon publication of any related work, to discuss how to proceed. Should you foresee a problem in meeting this three-month deadline, please let us know in advance and we may be able to grant an extension.

Thank you for the opportunity to consider your work for publication. I look forward to your revision.

Yours sincerely,

Cornelius Schneider, PhD
Editor
The EMBO Journal
c.schneider@embojournal.org

Instructions for preparing your revised manuscript:

Please make sure you upload a letter of response to the referees' comments together with the revised manuscript.

Please also check that the title and abstract of the manuscript are brief, yet explicit, even to non-specialists.

When assembling figures, please refer to our figure preparation guideline in order to ensure proper formatting and readability in print as well as on screen:
https://bit.ly/EMBOPressFigurePreparationGuideline
See also guidelines for figure legends: https://www.embopress.org/page/journal/14602075/authorguide#figureformat

At EMBO Press we ask authors to provide source data for the main manuscript figures. You will receive a separate email with instructions for providing source data with your revised manuscript, including how to upload and organize the files.

IMPORTANT: When you send the revision we will require
- a point-by-point response to the referees' comments, with a detailed description of the changes made (as a word file).
- a word file of the manuscript text.
- individual production quality figure files (one file per figure)
- a complete author checklist, which you can download from our author guidelines (https://www.embopress.org/page/journal/14602075/authorguide).
- Expanded View files (replacing Supplementary Information)
Please see out instructions to authors
https://www.embopress.org/page/journal/14602075/authorguide#expandedview
- a Reagents and Tools Table as part of the Methods section, which can be downloaded from our author guidelines (https://www.embopress.org/page/journal/14602075/authorguide#structuredmethods)

Please remember: Digital image enhancement is acceptable practice, as long as it accurately represents the original data and conforms to community standards. If a figure has been subjected to significant electronic manipulation, this must be noted in the

figure legend or in the 'Materials and Methods' section. The editors reserve the right to request original versions of figures and the original images that were used to assemble the figure.

Further information is available in our Guide For Authors: https://www.embopress.org/page/journal/14602075/authorguide

We realize that it is difficult to revise to a specific deadline. In the interest of protecting the conceptual advance provided by the work, we recommend a revision within 3 months (28th Jul 2025). Please discuss the revision progress ahead of this time with the editor if you require more time to complete the revisions. Use the link below to submit your revision:

https://emboj.msubmit.net/cgi-bin/main.plex

Referee #1:

Davies et al., identified OAS2 as an antiviral gene in a screen against human coronavirus HCoV-OC43. They then investigated the roles of OAS2 isoforms in antiviral activity primarily against human coronavirus HCoV-OC43 and then against the unrelated picornavirus EMCV. They conclude that the OAS2 p69 isoform is antiviral for HCoV-OC43 in an RNase L independent fashion while requiring dsRNA sensing. In contrast, p71 is antiviral against EMCV, and dependent on RNase L. These isoforms are splice variants which are diffentiated by their C terminal tails and colocalization to the Golgi (p69) and/or the ER (p71).

The screen and other molecular biology are well done, but the basic virology is lacking and the data do not convincingly support the conclusions.

1.The conclusions re: OC43 are almost entirely based on overexpressed OAS2 isoforms. This can be misleading and it is important to show the inhibitory effect with endogenous levels of OAS2. The only experiment to address endogenous levels is shown in Figure 2 i,j,k. They have knocked out OAS2 but in order to observe an effect on virus replication they induce OAS with IFN; the IFN which induces many ISG antiviral genes alone produced a 40 fold reduction in virus titer and the KO of OAS2 in those IFN treated cells promotes 1.4- to 2.4-fold increase in virus titer above the 40 fold reduction (without stats to show significance). This is convoluted and not convincing to demonstrate an effect of endogenous levels of OAS2. A simple experiment to address this point would be to perform an OC43 growth curve in WT and OAS2 KO cells (at a defined MOI) and ask if OAS2 is induced during infection and if virus titer is increased in the OAS2 KO cells over time.

The EMCV data are also lacking in that the authors report 2.5 fold reduction in titer in cells with overexpressed OAS2 p71, again without statistical significance shown. In addition, the RNase L KO data are confusing in that it is stated there were 3 guide RNA used to KO but results are shown only for guide 1. Moreover, there is a 10 fold increase in titer with RNase L KO'd but the control cells show 4 fold increased viral titer. The plaque sizes, also referred to are difficult observe and plaque sizes should be quantified if they are to be used to support the conclusions.

2. There are no data addressing whether RNase L is actually activated or not. This is especially important in the case of EMCV, when again OAS2 activity is measured by overexpression only and the effects of RNase L KO are minimal compared to control. It is important to know whether the OAS/RNase L pathway is activated and/or inhibited during infection. In fact the Li et al., 2016 reference shows EMCV does not activate RNase L in A549 cells. Finally, while EMCV infection is assessed only at one time point, activation of RNase L may change over time of infection and with MOI and this is not addressed.

3. The authors identify putative dsRNA binding regions in OAS2 by structural predictions and informatics, but never actually show that dsRNA binding occurs. In fact Fig S3c shows non significant association between OAS p69 and dsRNA. The authors conclude that dsRNA binding is required for inhibitory activity, but OAS2 enzymatic activity is not required. How do they envision an antiviral mechanism that involves dsRNA but not the OAS2 enzymatic activity (see point 5 below)?

4. Virus quantification. Many of the assays used for viral replication involve qPCR for N RNA copies and/or western blot for N protein which do not measure actual infectious virus produced and/or released by the cell. It is not explained why they use these assays when they do use plaque assays for infectious virus replication occasionally in some figures. In some figures rather than designate MOI as the amount of virus used for an infection, they use a dilution of virus stock -Fig 1 for example- which gives the reader no idea how much virus is being put on the cells.

5. Discussion of previous literature is lacking.

There is some discussion of replication organelles and single and double membrane vesicles in OC43 and EMCV infection. CoVs and picorna viruses produce very different replication organelles and this should be incorporated into the discussion. This is relevant to the different localization of the OAS2 isoforms. CoVs induce rearranged ER to form DMVs where viral RNA is replicated, yet OAS2p69 is localized to the Golgi. How do they envision OAS2 p69 inhibiting OC43 at the Golgi (point 3 above); CoVs bud into the ERGIC so by the time virions reach the Golgi they have been assembled and are on the way out of the cell.

There is no mention of the literature showing that OAS3 binds dsRNA more efficiently than the shorter isoforms and that OAS3 is reported to be the major isoform to activate RNase L to restrict replication of several families of viruses.

Referee #2:

Overall this is a highly interesting and well conducted study which should be supported. It uses a well established methodology to identify interferon stimulated genes that restricts OC43 replication and makes the surprising discovery that two different OAS2 splice isoforms have different activities, either direct restriction, or induction of interferon responses. The outcome being that the different splice isoforms vary in particular to restrict different viruses. The major interest of the study is that OAS2 can act as both a restriction factor and an innate sensor due to splice variation. As the authors note, there have been descriptions of restriction factors having a dual role in restriction and sensing, and there are also descriptions of restriction factors having variable function according to splice variation. What is unique here is that sensing vs restriction is affected by splice variation. A further strength of the study is the provision of a dataset which identifies ISGs that are able to restriction the common cold coronavirus OC43, and there is still much interest in understanding immune control of coronaviruses.
Most of my concerns are quite sporadic and are outlined below. At times I do think the rationale for certain experimental approaches needs to be outlined. My one area of concern is that the paper is immunologically quite weak in it's current form (but could readily be corrected). Many of the conclusions about innate sensing or lack of are supported by proxy assays or implication only, and all of the work only done in cell lines. To support the immunological claims of this manuscript in its current form, this aspect would need to be strengthened to be compelling.

Major Concerns

As noted above, the conclusions rest on the idea that the OAS2 p71 splice variant acts in an RNASEL and hence IFN dependent manner, but this only ever implied through RNASEL depletion and not directly shown. There needs to be more control data in figures, 2, 3, 5, 6 showing that IFN expression has or has not been induced. This could be more systematic characterisation of ISG expression (qPCR, Western, RNAseq) or in signalling characterisation leading to IFN production. Ideally, in the first described instance or p71 vs p69 phenotypes, and in critical experiments (e.g. figure 6) both should be performed.

The authors should show lymphocytes or macrophages responding to interferon in the context of p71 and p69 expression, to better connect with the immune response especially given the high lymphocyte expression seen in figure 2a and that ECMV has been described to infect lymphocytes.

Figure 6e:
The data suggest that RNAse-L itself is antiviral, but the explanation in the text (lines 347-349) is currently too vague.
Clarify this point and repeat the assay in a cell line with endogenous OAS knocked out to isolate the effect of overexpressed p71. Ideally consider using CRISPR to remove endogenous OAS2 and repeat the experiment for more robust validation.

Figure 6:
The rationale for using EMCV is unclear-why was this virus chosen? This should be justified in the text.

Use of INFR-KO Cells: The authors should use an INFR-KO line or another immunologically unresponsive cell line to assess the involvement of the IFN pathway when making conclusions in regards RNASEL.

Minor Concerns

Introduction:
Lines 45-46: The justification for choosing OC43 is weak-this should be made clearer.
Lines 55-56: The claim that this is a completely novel finding is perhaps overstated-consider rewording to "poorly described" or similar.

Results:
Figure 1b:
The rationale for using a macaque library is unclear. Since OC43 is stated to originate from rodents, why was a mouse library not used instead? This choice should be justified or rationalised.

Figure 1c:
Show an example of flow data in the supplementary figures for better clarity.

Figure 1h:
Why not perform this experiment in IFN-deficient cells to isolate IFN-independent effects?

Figure 1g:
OAS2 validation is missing-did the authors fail to find it? This should be addressed.

Figure 2a:
Tissue distribution data is confusing-why are the dots connected? This implies a trend, but is that appropriate? I suspect not and the data should be presented differently.

Figure 1:
Four different methods are used to measure OC43 (dsRNA fluorescence, qPCR, Western blot, plaque assay). A correlation analysis of results across these assays should be provided on the same viral stock and presented as a supplement, otherwise it is hard to compare across the analyses.

Line 184:
Incorrect figure reference-correct from Figure 2b to Figure 2c.

Supplementary Figure 4d:
Is there really more basic than acidic residues? A quantification should be included to support this claim because it is hard to tell just by looking.

Figure 4:
The mutants are not validated-there is no direct data confirming they do not bind dsRNA. Experimental validation is required.

Figure 5:
The lack of catalytic activity in the mutants is not experimentally validated. The authors should provide evidence confirming this, or otherwise make it clear that this a claim that is beyond dispute in the literature.

Figure 6:
The choice of EMCV needs better justification in the text.

Figure 7g:
Why did the authors not use ECMV here, despite using it in earlier experiments? This should be approached experimentally or justified.

Text Clarity Issues:
Lines 371-374: The meaning of this section is unclear. The authors should rephrase for clarity.

The authors identify that there are instances or dual restriction factors/sensors, they similarly note instances of splice variants of immune genes having differential functionality, which has been identified in a number of contexts.

Referee #3:

The manuscript by Davies et al investigates the antiviral properties of two distinct isoforms of OAS2. Their findings reveal that the OAS2 p69 isoform effectively inhibits the HCoV-OC43 virus, whereas the p71 isoform exhibits inhibitory effects on various viruses, including EMCV. Additionally, they discovered that the mechanism by which p69 restricts HCoV-OC43 is independent of RNase L, in contrast to the antiviral activity of p71, which relies on RNase L. The growing attention on various OAS proteins underscores the significance of discovering the antiviral effects of p69 that do not rely on RNase L. However, the following points should be addressed for the acceptance of this manuscript.
1. The current literature contains many ambiguous reports on the antiviral activities of OAS proteins across various cell types and viruses. Therefore, it is crucial for the authors to demonstrate the antiviral activity of p69 using a different cell type, ideally a primary cell other than A549.
2. Additionally, the authors should compare virus replication in IFN-untreated cells using both WT and pOAS2-KO cells at a low MOI to establish a more physiologically relevant system that reflects endogenous IFN induction and antiviral activity.
3. Lastly, given the recent discovery of OAS1-mediated enhancement of IFN induction, the authors are encouraged to explore this aspect and elucidate the molecular mechanism underlying the antiviral activity of p69 in the context of HCoV-OC43.

EMBOJ-2025-120553R; Alternative splicing broadens antiviral diversity at the human OAS2 locus

Referee #1 (Report for Author)

Davies et al., identified OAS2 as an antiviral gene in a screen against human coronavirus HCoV-OC43. They then investigated the roles of OAS2 isoforms in antiviral activity primarily against human coronavirus HCoV-OC43 and then against the unrelated picornavirus EMCV. They conclude that the OAS2 p69 isoform is antiviral for HCoV-OC43 in an RNase L independent fashion while requiring dsRNA sensing. In contrast, p71 is antiviral against EMCV, and dependent on RNase L. These isoforms are splice variants which are diffentiated by their C terminal tails and colocalization to the Golgi (p69) and/or the ER (p71).

The screen and other molecular biology are well done, but the basic virology is lacking and the data do not convincingly support the conclusions.

1.The conclusions re: OC43 are almost entirely based on overexpressed OAS2 isoforms. This can be misleading and it is important to show the inhibitory effect with endogenous levels of OAS2. The only experiment to address endogenous levels is shown in Figure 2 i,j,k. They have knocked out OAS2 but in order to observe an effect on virus replication they induce OAS with IFN; the IFN which induces many ISG antiviral genes alone produced a 40 fold reduction in virus titer and the KO of OAS2 in those IFN treated cells promotes 1.4- to 2.4-fold increase in virus titer above the 40 fold reduction (without stats to show significance). This is convoluted and not convincing to demonstrate an effect of endogenous levels of OAS2. A simple experiment to address this point would be to perform an OC43 growth curve in WT and OAS2 KO cells (at a defined MOI) and ask if OAS2 is induced during infection and if virus titer is increased in the OAS2 KO cells over time.

Thanks for the comments. We agree, this was an under-developed aspect of our manuscript. We thank the reviewer for their suggestion of OC43 growth curves. We have now performed a more comprehensive set of experiments, in the presence or absence of exogenous IFN, comparing OC43 replication in control parental and OAS2 KO cells, in both A549 (Fig 2K,L) and THP-1 (Fig 2M,N) cells – the best validated guide was used throughout. MOI 0.01, n = 3 independent experiments. Three observations we'd like to note:

1) In A549, OC43 growth is equivalent in the presence or absence of OAS2. How does this square with OAS2 being antiviral? In parallel Western blots (Fig. EV2G), we see no induction

of OAS2 protein during viral replication (in parental control cells), likely explaining the absence of measurable difference between parental and KO cells in the absence of exogenous IFN. It is interesting that infection alone is insufficient to induce OAS2 expression. We think this is because OC43 is a poor inducer or IFN in these cells (see new Fig. 6B, and PMID: 39869630).

2) In A549, in the presence of exogenous IFN, we see a reduction in IFN antiviral potency at the latest time point (72 hpi) in OAS2 KO cells, compared to control cells. These data therefore support a role for endogenous OAS2 in the IFN-induced control of HCoV-OC43. We think that IFN is necessary to reveal this modest phenotype because OAS2 is not expressed at detectable levels in the absence of IFN (Fig. 2C). As proposed by John Schoggins et al (e.g. PMID 37497756) and others, we envisage that IFN induces the expression of multiple anti-HCoV-OC43 genes simultaneously, which act additively to block infection and of which OAS2 is just one. Plausibly, distinct ISGs that act earlier on viral replication than OAS2 (for example, at viral entry) could mask the effects of OAS2 expression. Indeed, our ISG screening data proposes two such candidate anti-OC43 ISGs – LY6E and CTSS – that have known inhibitory effects on CoV entry (PMIDs: 35421191 and 32704094). Thus, it does become problematic dissecting relative ISG potency from KO experiments during IFN treatment. Nonetheless, our experiments in A549 cells do reveal a contributory role for OAS2 in the IFN response against HCoV-OC43.

In this context, we advocate for the use overexpression as a means of testing the *specificity* of individual genes in isolation of their ISG counterparts. An important caveat is that the relative *potency* of ISG overexpression must not be over-interpreted, and we have taken care not to do this. Indeed, a potent phenotype on overexpression is only useful in so far as providing a large dynamic range in which to test further hypotheses such as cofactor requirement etc. We would like to note too that the level of OAS2 expression we achieve by overexpression is not substantially higher than that achieved by overnight IFN stimulation (Fig. 2C).

3) We also performed the same experiments in THP-1 cells (Fig 2M,N). Here we see the same general trend, with a modest reduction in IFN efficacy in OAS2 depleted cells at late time points of replication. We also see a modest rescue to HCoV-OC43 replication upon OAS2 depletion, in the absence of IFN stimulation.

Combined, these experiments support a role for endogenous OAS2 in HCoV-OC43 replication in both epithelial cells and myeloid cells.

The EMCV data are also lacking in that the authors report 2.5 fold reduction in titer in cells with overexpressed OAS2 p71, again without statistical significance shown. In addition, the RNase L KO data are confusing in that it is stated there were 3 guide RNA used to KO but results are shown only for guide 1. Moreover, there is a 10 fold increase in titer with RNase L KO'd but the control cells show 4 fold increased viral titer. The plaque sizes, also referred to are difficult observe and plaque sizes should be quantified if they are to be used to support the conclusions.

Thanks very much for your comments here – we are grateful to have had the opportunity to improve our experimental design. In performing revision experiments we realised that we had not been analysing EMCV spread at the most appropriate time points. When we fixed cells at an earlier, 30-hour time point, plaques were clearer, more uniform, more countable, and the effect of OAS2 p71 expression was more pronounced. The revealed potency of restriction enabled us to dissect the enzymatic and catalytic requirements of p71 restriction more clearly than previously presented, now presented in a new Fig 7.

In summary of our new figure, comprising entirely new data:

1. We show p71 restricts EMCV replication in a multi-step growth assay in A549 cells (Fig. 7A) and THP-1 cells (Fig. 7B); in the latter, p69 also restricts EMCV replication, revealing that cell background also influences restriction profile.
2. We are unable to reveal a role for endogenous OAS2 in EMCV restriction in A549, with or without IFN stimulation, however this is commensurate with the lack of OAS2 expression in A549 treated with IFN (Fig. 2C) and the induction of p69, but not p71, in A549 cells treated with IFN (Fig. 2C) – in A549 cells, p69 does not restrict EMCV (Fig. 7A). We do see a modest rescue to EMCV replication in THP-1 cells upon OAS2 depletion (Fig. 7E); again, we only see p69 expression in THP-1 cells (Fig. 2I) however in these cells, p69 does exhibit anti-EMCV activity (Fig. 7B). We hypothesise that IFN induces too many additional factors that inhibit EMCV in THP-1 cells, for us to discern any role for endogenous OAS2 under these conditions.
3. In Fig 7 H–L, we demonstrate more clearly now that interruption of p71 catalysis through the well-described D481A substitution, renders p71 inactive against EMCV. Moreover, under our new experimental protocol, we show that p71 restriction of EMCV is entirely RNase L dependent.
4. We show that p71 restriction occurs independently of IFN signaling, as it remains restrictive against EMCV in cells also expressing the BVDV NPro protein (Fig. 7M,N)

that depletes IRF3 and ablates IFN signaling (Appendix). We were also unable to detect IFN signaling in cells expressing p71 infected with EMCV (Fig. 7O).

Regarding statistical tests, we apologise for this omission and have now performed appropriate tests on all relevant experiments. All test details are included in a separate section (Appendix Table S2).

Regarding the three KO guides, we have continued with a single RNase L guide, as while all three guides were effective (Fig. 5D), guide 1 was consistently the most effective by Western blot (e.g. Fig. 7J), so we have predominantly used this guide for experiments.

2. There are no data addressing whether RNase L is actually activated or not. This is especially important in the case of EMCV, when again OAS2 activity is measured by overexpression only and the effects of RNase L KO are minimal compared to control. It is important to know whether the OAS/RNase L pathway is activated and/or inhibited during infection. In fact the Li et al., 2016 reference shows EMCV does not activate RNase L in A549 cells. Finally, while EMCV infection is assessed only at one time point, activation of RNase L may change over time of infection and with MOI and this is not addressed.

Thanks for this comment. We hope that our new RNase L-dependence data (point 3 above) alleviates some of this concern, however we were keen to see whether we could detect RNase L activation during p71 restriction of EMCV replication. To this end, we have monitored total ribosomal RNA integrity, using cells expressing either RFP, OAS2 p69 or OAS2 p71 (Fig. 7L). Cells were either mock transfected, transfected with 1 µg/mL poly(I:C), or infected with EMCV, MOI = 2. After 24 h, purified RNA was assessed using an Agilent TapeStation. The dsRNA mimic poly(I:C) served as a control for RNase L activation (e.g PMID 31494035). Indeed, poly(I:C) and EMCV infection both triggered rRNA degradation, indicated by reduced RNA integrity (RIN) values and the appearance of a peak around 1100 nucleotides (nt). While the response to poly(I:C) was equivalent between all three cell lines, the response to EMCV was greatest in p71-expressing cells, reflected in both the lowest RIN value, and the greatest shift in peak intensity at 1100 nt. These data suggest that both poly(I:C) and EMCV induce RNase L degradation, potentially via OAS3, as previously reported, while OAS2 p71 induces an additional activation of RNase L in response to EMCV.

3. The authors identify putative dsRNA binding regions in OAS2 by structural predictions and informatics, but never actually show that dsRNA binding occurs. In fact Fig S3c shows non significant association between OAS p69 and dsRNA. The authors conclude that dsRNA

binding is required for inhibitory activity, but OAS2 enzymatic activity is not required. How do they envision an antiviral mechanism that involves dsRNA but not the OAS2 enzymatic activity (see point 5 below)?

This is a great question. To clarify this point, we believe that the p69 isoform binds dsRNA but does not require canonical enzymatic activity to levy its block against OC43 replication. Conversely, the p71 isoform does require canonical enzymatic activity. We have clarified this point by introducing the D481A and R529E/R533E substitutions in p71 and showing that these both abolish p71 restriction of EMCV (Fig. 7 H,I). We can now also clarify that p71 restriction of EMCV is entirely RNase L dependent (Fig 7 J,K). If we assume then that p71 works via the textbook mechanism of OAS antiviral function, how does OAS2 p69 restrict in the absence of RNase L? Potentially p69 binds RNA and sequesters it, limiting replication in a non-catalytic manner. OAS2 was very recently reported to recruit to stress granules, for example (PMID 40588419). Alternatively, p69 might be regulating IFN induction during HCoV-OC43 induction, as recently reported for human OAS1, which binds cellular mRNAs, adjusts mRNA stability, cell signalling and West Nile Virus (WNV) permissivity indirectly (Harioudh *et al* PMID: 38423012). We have performed additional experiments to explore a role for IFN, which is reported in a new Fig 6.

We first looked for functional IFN production during infection of A549 cells expressing an ISRE-GFP construct, and co-expressing RFP, OAS2 p71 or p69 (Fig 6A). While soluble IFNβ was able to induce robust ISRE promoter activation as measured by GFP expression, HCoV-OC43 infection was only able to do so in p69-expressing cells, at the latest time point measured (72 hpi). However, the effect was very modest in size (Fig 6B). To ask whether this was a functional induction of IFN that contributes to p69 restriction, we sought to abolish IFN signaling genetically (Fig 6C,D) and pharmacologically (Fig 6E). Neither treatment blunted the antiviral activity of p69 against HCoV-OC43.

In the aforementioned paper from Harioudh *et al.*, IFNB1 mRNA was stabilised by OAS1 overexpression. To test this, we performed the same experiments by stimulating cells with poly(I:C) and measuring IFNB1 expression a time points post transfection. However, p69 expression had no impact on IFNB1 mRNA stability (Fig. 6F).

To demonstrate that OAS2 isoforms bind RNA – a feature we apologise we did not test empirically in our previous submission – we have used biotin-poly(I:C) and shown that both p71 and p69 can be enriched from cell extracts following incubation with the RNA mimic (Fig 4D). However, reproducibly, we observed that p69 bound poly(I:C) more weakly than p71.

Nonetheless, the R529E/R533E substitution we used to test a requirement for RNA binding, ablated binding to poly(I:C) in these enrichment experiments. Supporting this, Merold *et al* (PMID 40412389) recently also identified this double substitution and showed it abolishes 2-5A synthesis in cells, while the mutation has also been characterised in OAS1 and shown to disable RNA binding and catalytic activity (PMID: 14636576).

4. Virus quantification. Many of the assays used for viral replication involve qPCR for N RNA copies and/or western blot for N protein which do not measure actual infectious virus produced and/or released by the cell. It is not explained why they use these assays when they do use plaque assays for infectious virus replication occasionally in some figures. In some figures rather than designate MOI as the amount of virus used for an infection, they use a dilution of virus stock -Fig 1 for example- which gives the reader no idea how much virus is being put on the cells.

Thank you for pointing this out – we agree our decision to use a particular assay was not always justified. The PCR and Western blots were not intended to measure infectious virus, but rather as useful surrogates to compare between genetic backgrounds or variants. We have better clarified this in the text now. We have also performed Fig 2K, J, M to more fully explore endogenous OAS2 using replicating virus readouts, and used replication experiments to better explore the role of the OAS2 C terminal tails in the new Fig 8. We agree our previous labelling of virus dilution in Fig. 1 was unhelpful, and have now converted these values to MOI.

5. Discussion of previous literature is lacking.

There is some discussion of replication organelles and single and double membrane vesicles in OC43 and EMCV infection. CoVs and picorna viruses produce very different replication organelles and this should be incorporated into the discussion. This is relevant to the different localization of the OAS2 isoforms. CoVs induce rearranged ER to form DMVs where viral RNA is replicated, yet OAS2p69 is localized to the Golgi. How do they envision OAS2 p69 inhibiting OC43 at the Golgi (point 3 above); CoVs bud into the ERGIC so by the time virions reach the Golgi they have been assembled and are on the way out of the cell.

Thanks for this detail, this is helpful and we agree we could have clarified this in the discussion. We have added the following sentence in the discussion to elaborate on this:

“The question of precisely where and how membrane-bound OAS2 senses viral RNA remains unclear, although whilst this manuscript was in revision, the requirement of OAS2 anchoring to the Golgi network was confirmed by Merold et al. (Merold et al., 2025). Coronaviruses and cardioviruses such as EMCV have been reported to replicate in structures resembling single or double-membrane vesicles (DMV) (Knoops et al, 2008; Melia et al, 2018). Although the ER has been proposed to be the donor organelle for such structures, OAS1 similarly localises to a Golgi compartment, and can potently restrict other human coronaviruses and EMCV (Soveg et al., 2021; Wickenhagen et al., 2021). Therefore, across OAS systems, the topology of dsRNA sensing remains unclear, although at the resolution provided by our own and others’ experiments so far, OAS2 does not appear to localise to the ER. We note that vRNA can transit through exit pores (Chen et al, 2024), which could facilitate exposure to OAS enzymes anchored to the cytosolic face of the vesicle. Alternatively, anchored OASes could infiltrate the DMV during its biogenesis from the endomembrane system. It is possible that the two OAS2 isoforms exhibit subtly different distribution patterns which better enable their detection of their respective targets, however we cannot discern this at the resolution of our imaging.”

There is no mention of the literature showing that OAS3 binds dsRNA more efficiently than the shorter isoforms and that OAS3 is reported to be the major isoform to activate RNase L to restrict replication of several families of viruses.

Apologies for this omission. We didn’t delve too deeply into the relative dsRNA binding efficiencies of the different OAS genes; in part, because the matter seems not entirely resolved, and we feel is somewhat outwith the focus of our paper. In vitro studies originally suggest OAS3 binds longer RNAs, however recent data from Merold et al suggest that OAS2 is in fact activated by longer RNAs than OAS3 (PMID 40412389). Because we haven’t sought to square RNA length with restriction by either OAS2 isoform, we’re not sure how best to reference OAS3 and long dsRNA, but we have expanded on this in the introduction.

We had previously referenced the Li *et al* (2016) paper, which is the most relevant paper showing OAS3 is the predominant family member to activate RNase L. However, these experiments were performed in A549 cells which predominantly express the p69 isoform, according to our data. Therefore, the importance of the p71 isoform was not thoroughly investigated.

The Li *et al* (2016) paper also focussed on replication of 4 diverse viruses (SINV, CHIKV, IAV △NS1 and VACV△E3L). Many viruses reported to be sensitive to OAS1 and OAS2, such as coronaviruses, have ssRNA genomes. SINV and CHIKV are alphaviruses that utilise endosomes and lysosomes for replication, whereas influenza virus A (IAV) replicates in the nucleus. Given that OAS1 p46 and OAS2 isoforms localise to the endomembrane system, viral dsRNA derived from the above four viruses may not come into proximity to OAS1 and OAS2, and thus RNase L activation is unlikely to occur during infection.

Referee #2 (Report for Author)

Overall this is a highly interesting and well conducted study which should be supported. It uses a well established methodology to identify interferon stimulated genes that restricts OC43 replication and makes the surprising discovery that two different OAS2 splice isoforms have different activities, either direct restriction, or induction of interferon responses. The outcome being that the different splice isoforms vary in particular to restrict different viruses. The major interest of the study is that OAS2 can act as both a restriction factor and an innate sensor due to splice variation. As the authors note, there have been descriptions of restriction factors having a dual role in restriction and sensing, and there are also descriptions of restriction factors having variable function according to splice variation. What is unique here is that sensing vs restriction is affected by splice variation. A further strength of the study is the provision of a dataset which identifies ISGs that are able to restriction the common cold coronavirus OC43, and there is still much interest in understanding immune control of coronaviruses.

We'd like to thank the reviewer for their supportive words; we're delighted that they were excited by these findings. We hope we've now provided sufficient supportive data to satisfy the following concerns.

Most of my concerns are quite sporadic and are outlined below. At times I do think the rationale for certain experimental approaches needs to be outlined. My one area of concern is that the paper is immunologically quite weak in it's current form (but could readily be corrected). Many of the conclusions about innate sensing or lack of are supported by proxy assays or implication only, and all of the work only done in cell lines. To support the

immunological claims of this manuscript in its current form, this aspect would need to be strengthened to be compelling.

Major Concerns

As noted above, the conclusions rest on the idea that the OAS2 p71 splice variant acts in an RNASEL and hence IFN dependent manner, but this only ever implied through RNASEL depletion and not directly shown. There needs to be more control data in figures, 2, 3, 5, 6 showing that IFN expression has or has not been induced. This could be more systematic characterisation of ISG expression (qPCR, Western, RNAseq) or in signalling characterisation leading to IFN production. Ideally, in the first described instance or p71 vs p69 phenotypes, and in critical experiments (e.g. figure 6) both should be performed.

Thanks for this suggestion. We agree that our data does invoke a model whereby p71 acts in a signalling-dependent manner (via 2-5A), although because RNase L is constitutively expressed, we don’t see the how acting via RNase L necessitates an IFN dependent mechanism – other than OAS2 expression being IFN-dependent in these cells. We have addressed this in several ways. First, we directly looked for ISRE promoter activation during EMCV restriction by p71, but find no evidence for this, suggesting IFN had not been induced (Fig. 7O). We also use the BVDV NPro IFN antagonist and show that despite potent IFN signalling inhibition in these cells (Appendix Fig. 1), OAS2 p71 remains effective in restricting EMCV (Fig. 7M,N), suggesting that even if IFN had been produced by OAS2 during EMCV restriction, this had no role in the restriction mechanism itself.

We performed similar experiments for p69 restriction of HCoV-OC43, also failing to collect data that supports a role for IFN in the restriction mechanism (Fig. 6). We note that HCoV-OC43 is adept at evading IFN induction in these cells (PMID: 39869630).

To assess whether 2-5A signalling has taken place during EMCV restriction by p71, we have measured RNA integrity, as elaborated in our response to Referee 1, point 2 above, observing that EMCV infection of p71-expressing cells induces the strongest reduction of total RNA integrity, compared to RFP- or p69-expressing cells. This supports the role for RNase L in p71 restriction of EMCV indicated by our genetic experiments.

The authors should show lymphocytes or macrophages responding to interferon in the context of p71 and p69 expression, to better connect with the immune response especially

given the high lymphocyte expression seen in figure 2a and that ECMV has been described to infect lymphocytes.

Thanks for this comment. We have monitored OAS2 expression in monocytic THP-1 (Fig. 2I) and observed p69 rather than p71 expression upon IFN stimulation. This expression profile supports the restriction data we also observed in THP-1, where the isoform-specificity is more relaxed than in A549. In particular, p69 is able to restrict both HCoV-OC43 and EMCV in THP-1, and at the endogenous level, depletion of OAS2 from THP-1 causes modest rescues to both HCoV-OC43 (Fig. 2) and EMCV (Fig. 7).

Figure 6e:
The data suggest that RNAse-L itself is antiviral, but the explanation in the text (lines 347-349) is currently too vague.
Clarify this point and repeat the assay in a cell line with endogenous OAS knocked out to isolate the effect of overexpressed p71. Ideally consider using CRISPR to remove endogenous OAS2 and repeat the experiment for more robust validation.

We agree this data needed improving. We realised that we had not been analysing EMCV spread at the most appropriate time points. When we fixed cells at an earlier, 30-hour time point, plaques were clearer, more uniform, more countable, and the effect of OAS2 p71 expression was more pronounced. The revealed potency of restriction enabled us to dissect the enzymatic and catalytic requirements of p71 restriction more clearly than previously presented, now presented in a new Fig 7. In Fig 7H-L, we demonstrate more clearly now that interruption of p71 catalysis through the well-described D481A substitution, or RNase L depletion, renders p71 completely inactive against EMCV.

Figure 6:
The rationale for using EMCV is unclear-why was this virus chosen? This should be justified in the text.

Thanks. We have inserted the following sentence to introduce the new Fig 7:

To explore whether OAS2 might execute RNase L-dependent restriction under alternative scenarios, we tested its ability to restrict the picornavirus cardiovirus A (EMCV). Like HCoV-OC43, EMCV replicates in membranous organelles (Romero-Brey & Bartenschlager, 2014) and was shown to be sensitive to OAS2 (Marié et al., 1999) and OAS1 (Wickenhagen et al., 2021).

Use of INFR-KO Cells: The authors should use an INFR-KO line or another immunologically unresponsive cell line to assess the involvement of the IFN pathway when making conclusions in regards RNASEL.

We show that p71 restriction occurs independently of IFN signaling, as it remains restrictive against EMCV in cells also expressing the BVDV NPro protein (Fig. 7M,N) that depletes IRF3 and ablates IFN signaling (Appendix Fig. 1A-B). We were also unable to detect IFN signaling in cells expressing p71 infected with EMCV (Fig. 7O).

Minor Concerns

Introduction:
Lines 45-46: The justification for choosing OC43 is weak-this should be made clearer.

We chose HCoV-OC43 for multiple reasons, that have been mentioned albeit not explicitly in our manuscript:

1. At the time of optimising a dsRNA staining based screen, there were no widely available reporter systems for HCoV-OC43 (lines 69-71)
2. HCoV-OC43 emerged into humans 100-200 years ago and has now become an endemic virus that causes a significant proportion of common cold cases (mentioned lines 46-48). Understanding virus-host dynamics using HCoV-OC43 may help us uncover how emerging coronaviruses, such as SARS-CoV-2, would adapt to human hosts.
3. HCoV-OC43 is a rodent-originating coronavirus. At the time of optimising a dsRNA staining based cDNA screen, cDNA screens had only been performed against bat-originating coronaviruses (SARS-CoV-2, HCoV-229E). We hoped this may shed light on restriction factors of coronaviruses that may have been influenced by their ancestral host.

We have added the qualifying sentence in the text:
“Understanding this evolutionary trajectory and the factors that influence HCoV-OC43-human interaction could aid in understanding how emerging coronaviruses adapt to human hosts.”

Lines 55-56: The claim that this is a completely novel finding is perhaps overstated-consider rewording to "poorly described" or similar.

We have changed our wording from “novel mechanism” to “mechanism”.

Results:

Figure 1b:

The rationale for using a macaque library is unclear. Since OC43 is stated to originate from rodents, why was a mouse library not used instead? This choice should be justified or rationalised.

There are ISGs present in the macaque library, not present in the human library. Therefore, by screening with both libraries, the range of ISGs that are investigated is enhanced. In some instances, a specific ISG from humans is antiviral but the equivalent ISG in macaques is not (or vice versa). This has previously helped to identify key residues that facilitate antiviral activity. We did not have a functioning mouse library to screen with when performing these experiments.

We have clarified this in the text by inserting the following sentence:

“Although not implicated in the emergence of HCoV-OC43, we included the macaque library to increase the range of ISGs screened; the macaque library contains ISGs shared by both humans and macaques, which are not present in the human library”

Figure 1c:

Show an example of flow data in the supplementary figures for better clarity.

We respectfully note that this data is image cytometry data, not flow cytometry data, although gating was performed using FlowJo software. We have now provided examples of this in Fig. EV1.

Figure 1h:

Why not perform this experiment in IFN-deficient cells to isolate IFN-independent effects?

This is a great idea, and a route that we have taken previously (Wickenhagen *et al.,* 2021). However, as our approach in the current manuscript has successfully identified a handful of anti-OC43 restriction factors, one of which we have pursued in depth and found to act as a

direct antiviral effector, we feel the approach we took was sufficient to uncover new antiviral biology.

Figure 1g:
OAS2 validation is missing-did the authors fail to find it? This should be addressed.

Yes, the bovine OAS2 orthologue fell beneath our transduction threshold when we performed the mini screens in the 96 well format. To test whether bovine OAS2 does indeed restrict, we prepared new lentivectors and selected for polyclonal cell populations expression human or bovine OAS2 isoforms. We were unable to confirm expression by Western blot, however when performing either plaque assays or intracellular dsRNA staining (as in Figure 1), we observed robust restriction by the bovine and human orthologues, supporting its initial detection in our bovine ISG screen. This data is now incorporated in Fig EV1.

Figure 2a:
Tissue distribution data is confusing-why are the dots connected? This implies a trend, but is that appropriate? I suspect not and the data should be presented differently.

Agreed. We have corrected this by removing the connecting lines, and moving this data to Fig EV1.

Figure 1:
Four different methods are used to measure OC43 (dsRNA fluorescence, qPCR, Western blot, plaque assay). A correlation analysis of results across these assays should be provided on the same viral stock and presented as a supplement, otherwise it is hard to compare across the analyses.

Thanks for the comments. The viral stock used in the initial ISG screen was different to that used in subsequent figures, so we have converted inoculum dose to MOI in Fig. 1. For a correlation across assays, infection at an MOI of 0.01 provided signals in qPCR at 72 h (Fig. 2F), WB at 72 h (Fig. 2H) and a growth curve titre at 72 h (Fig. 2D). The restriction phenotype (RFP vs p69) was observed across all of these assays.

We have also clarified in the text that these surrogate assays do not supplant nor equate with infectious virus, but rather serve as useful assays to compare restriction between multiple cell lines. We only sporadically use these alternative assays now having first established restriction of virus replication, and have repeated experiments (in triplicate)

previous instances where we had only used qPCR not infectious titre e.g. the new Fig. 8H-I, previous Fig. 7G.

Line 184:
Incorrect figure reference-correct from Figure 2b to Figure 2c.

Thanks for spotting. Corrected.

Supplementary Figure 4d:
Is there really more basic than acidic residues? A quantification should be included to support this claim because it is hard to tell just by looking.

Thanks for this. We have decided to remove this analysis, as there are a very many number of basic residues and it becomes a little woolly given this is only a modelled interaction.

Figure 4:
The mutants are not validated-there is no direct data confirming they do not bind dsRNA. Experimental validation is required.

To demonstrate that OAS2 isoforms bind RNA – a feature we apologise we did not test empirically in our previous submission – we have used biotin-poly(I:C) and shown that both p71 and p69 can be enriched from cell extracts following incubation with the RNA mimic (Fig 4D). However, reproducibly, we observed that p69 bound poly(I:C) more weakly than p71. Nonetheless, the R529E/R533E substitution we used to test RNA binding, ablated binding to poly(I:C) in these enrichment experiments. Supporting this, Merold *et al* (PMID 40412389) recently also identified this same double substitution and showed that it abolishes 2-5A synthesis in cells, while the mutation has also been characterised in OAS1 and shown to disable RNA binding and catalytic activity (PMID: 14636576).

Figure 5:
The lack of catalytic activity in the mutants is not experimentally validated. The authors should provide evidence confirming this, or otherwise make it clear that this a claim that is beyond dispute in the literature.

When the D481A variant is first introduced in the text, we have better emphasised the multiple supporting literature showing its defect in catalysis.

Figure 6:
The choice of EMCV needs better justification in the text.

Agreed, we apologies for not introducing this properly. We have added the following text at the start of Fig 7:

"To explore whether OAS2 might execute RNase L-dependent restriction under alternative scenarios, we tested its ability to restrict the picornavirus cardiovirus A (EMCV). Like HCoV-OC43, EMCV replicates in membranous organelles (Romero-Brey & Bartenschlager, 2014) and was shown to be sensitive to OAS2 (Marié et al., 1999) and OAS1 (Wickenhagen et al., 2021)."

Figure 7g:
Why did the authors not use ECMV here, despite using it in earlier experiments? This should be approached experimentally or justified.

We have now repeated these experiments to measure infectious virus for both HCoV-OC43 and EMCV, for a new Fig. 8 entirely focused on the C-terminal tails. We are very excited by the findings in this figure, which show that p71 restriction activity can be tuned down by shortening tail length, or ablated by making it longer, while p69 restriction activity is abolished by mutation of either of the final two residues in the tail.

Text Clarity Issues:
Lines 371-374: The meaning of this section is unclear. The authors should rephrase for clarity.

This section on the C-terminal OAS2 variants has been restructured due to new experiments, which has improved clarity.

The authors identify that there are instances or dual restriction factors/sensors, they similarly note instances of splice variants of immune genes having differential functionality, which has been identified in a number of contexts.

Referee #3 (Report for Author)

The manuscript by Davies et al investigates the antiviral properties of two distinct isoforms of OAS2. Their findings reveal that the OAS2 p69 isoform effectively inhibits the HCoV-OC43 virus, whereas the p71 isoform exhibits inhibitory effects on various viruses, including EMCV. Additionally, they discovered that the mechanism by which p69 restricts HCoV-OC43 is independent of RNase L, in contrast to the antiviral activity of p71, which relies on RNase L. The growing attention on various OAS proteins underscores the significance of discovering the antiviral effects of p69 that do not rely on RNase L. However, the following points should be addressed for the acceptance of this manuscript.

1. The current literature contains many ambiguous reports on the antiviral activities of OAS proteins across various cell types and viruses. Therefore, it is crucial for the authors to demonstrate the antiviral activity of p69 using a different cell type, ideally a primary cell other than A549.

Thanks for this comment. We have now expressed the OAS2 isoforms in THP-1 cells, performing growth curve experiments for both HCoV-OC43 (Fig 2) and EMCV (Fig 7). Antiviral activity of p69 is maintained in both cell types. However, and suggestive of an important role in cellular context in this gene's function, in THP-1 p71 has gained a modest restriction of HCoV-OC43, while p69 has gained substantial activity against EMCV.

2. Additionally, the authors should compare virus replication in IFN-untreated cells using both WT and pOAS2-KO cells at a low MOI to establish a more physiologically relevant system that reflects endogenous IFN induction and antiviral activity.

Thanks for the comments. We agree, this was an under-developed aspect of our manuscript. We thank the reviewer for their suggestion of low MOI infection and growth curves. We have now performed a more comprehensive set of experiments, in the presence or absence of exogenous IFN, comparing OC43 and EMCV replication in control and OAS2 KO cells, in both A549 (Fig 2K,L, Fig 7C,D) and THP-1 (Fig 2M,N, Fig 7E,F) cells – the best validated guide was used throughout. n = 3 independent experiments in all cases. Three observations we'd like to note:

1) In A549, HCoV-OC43 growth is equivalent in the presence or absence of OAS2. How does this square with OAS2 being antiviral? In parallel Western blots (Fig. EV2G), we see no

induction of OAS2 protein during viral replication (in parental control cells), likely explaining the absence of measurable difference between parental and KO cells in the absence of exogenous IFN. It is interesting that infection alone is insufficient to induce OAS2 expression. We think this is because OC43 is a poor inducer or IFN in these cells (see new Fig. 6B, and PMID: 39869630).

2) In A549, in the presence of exogenous IFN, we see a reduction in IFN antiviral potency at the latest time point (72 hpi) in OAS2 KO cells, compared to control cells. These data therefore support a role for endogenous OAS2 in the IFN-induced control of HCoV-OC43. We think that IFN is necessary to reveal this modest phenotype because OAS2 is not expressed at detectable levels in the absence of IFN (Fig. 2C). As proposed by John Schoggins et al (e.g. PMID 37497756) and others, we envisage that IFN induces the expression of multiple anti-HCoV-OC43 genes simultaneously, which act additively to block infection and of which OAS2 is just one. Plausibly, distinct ISGs that act earlier on viral replication than OAS2 (for example, at viral entry) could mask the effects of OAS2 expression. Indeed, our ISG screening data proposes two such candidate anti-OC43 ISGs – LY6E and CTSS – that have known inhibitory effects on CoV entry (PMIDs: 35421191 and 32704094). Thus, it does become problematic dissecting relative ISG potency from KO experiments during IFN treatment. Nonetheless, our experiments in A549 cells do reveal a contributory role for OAS2 in the IFN response against HCoV-OC43.

In this context, we advocate for the use overexpression as a means of testing the *specificity* of individual genes in isolation of their ISG counterparts. An important caveat is that the relative *potency* of ISG overexpression must not be over-interpreted, and we have taken care not to do this. Indeed, a potent phenotype on overexpression is only useful in so far as providing a large dynamic range in which to test further hypotheses such as cofactor requirement etc. We would like to note too that the level of OAS2 expression we achieve by overexpression is not substantially higher than that achieved by overnight IFN stimulation (Fig. 2C).

3) We also performed HCoV-OC43 growth curves in THP-1 cells (Fig 2M,N). Here we see the same general trend, with a modest reduction in IFN efficacy in OAS2 depleted cells at late time points of replication. We also see a modest rescue to HCoV-OC43 replication upon OAS2 depletion, in the absence of IFN stimulation.

Combined, these experiments support a role for endogenous OAS2 in HCoV-OC43 replication in both epithelial cells and myeloid cells.

We are unable to reveal a role for endogenous OAS2 in EMCV restriction in A549, with or without IFN stimulation, however this is commensurate with the lack of OAS2 expression in A549 treated with IFN (Fig. 2C) and the induction of p69, but not p71, in A549 cells treated with IFN (Fig. 2C) – in A549 cells, p69 does not restrict EMCV (Fig. 7A). We do see a modest rescue to EMCV replication in THP-1 cells upon OAS2 depletion (Fig. 7E); again, we only see p69 expression in THP-1 cells (Fig. 2I) where in these cells, p69 does exhibit anti-EMCV activity (Fig. 7B). We hypothesise that IFN induces too many additional factors that inhibit EMCV in THP-1 cells, for us to discern any role for endogenous OAS2 under these conditions.

3. Lastly, given the recent discovery of OAS1-mediated enhancement of IFN induction, the authors are encouraged to explore this aspect and elucidate the molecular mechanism underlying the antiviral activity of p69 in the context of HCoV-OC43.

We have performed a series of experiments to elucidate how p69 is restricting viral replication. While we don't have a single mechanism yet, our new data provide a fuller picture for how this isoform functions.

In a new Fig. 6, we first looked for functional IFN production during infection of A549 cells expressing an ISRE-GFP construct, and co-expressing RFP, OAS2 p71 or p69 (Fig 6A). While soluble IFNβ was able to induce robust ISRE promoter activation as measured by GFP expression, HCoV-OC43 infection was only able to do so in p69-expressing cells, at the latest time point measured (72 hpi). However, the effect was very modest in size (Fig 6B). To ask whether this was a functional induction of IFN that contributes to p69 restriction, we sought to abolish IFN signaling genetically (Fig 6C,D) and pharmacologically (Fig 6E). Neither treatment blunted the antiviral activity of p69 against HCoV-OC43.

In the aforementioned paper from Harioudh *et al.*, *IFNB1* mRNA was stabilised by OAS1 overexpression. To test this, we performed the same experiments by stimulating cells with poly(I:C) and measuring *IFNB1* expression a time points post transfection. However, p69 expression had no impact on *IFNB1* mRNA stability (Fig. 6F).

We have also expanded on our analysis of the role of the p69 C terminal tail, and performed an alanine scan to find that the final two residues – V686 and I687 – are critical for restriction activity. While we do not yet understand how p69 performs viral restriction independently of RNase L, this finding suggests that the short tail plays a key role in virus targeting and ongoing work is seeking to understand how this can be.

Referee cross-commenting:

Referee #1

Thanks for inviting to comment on the reviews. To me this manuscript lacks two major evidences that multiple reviewers pointed out:

1. Experiment comparing WT OAS3-KO cells to show the difference in viral replication.
2. A plausible molecular mechanism.

Without at least these two results this paper do not establish what it claims. Thanks.

After discussion with Dr Schneider, we have interpreted this as a typographical error and have performed experiments with OAS2, not OAS3. As discussed above, we have provided a series of experiments now to better explore a role for endogenous OAS2 in viral restriction.

Referee #2

I agree that these two points need to be addressed. But I think more data is needed to justify the conclusions. On the first point the authors need to show some data on whether infection actually activates the OAS/RNase L pathway, for example by an RNA degradation assay, widely used in the literature. On the second point the authors should consider/discuss the pre-pandemic literature on OAS-RNase L and viruses and discuss their data in the context of coronavirus biology/replication/life cycle.

The conclusions on EMCV are not justified by the data and should be removed unless substantiated with more data, such as use of KO cells in addition to overexpression.

We’ve addressed RNA degradation during p71 restriction of EMCV, expanded our discussion on OAS-RNase L in the context of CoV and EMCV replication, and we’ve now substantially enhanced the EMCV data, largely through optimising our plaque assay, revealing a more potent restriction phenotype allowing more concise dissection of the catalytic requirements of restriction. This substantive new data is now included in its own Figure (7).

In general, many of the experiments are performed superficially and need to be substantiated with more rigorous assays- plaque assays for infectious virus- not the same as genome copies or N Western blots, quantification of virus stocks and MOI of infections, demonstration of dsRNA binding when discussing dsRNA binding domains, all as I detailed in my review. The authors also need to show statistical significance especially in some data where differences are small.

In my opinion, this manuscript needs very major revisions.

Many thanks for these fair and reasonable comments. By addressing all of these points, we feel our paper is now significantly more compelling.

Referee #3

I agree with the broad points raised, which variously seem to be:

1. There needs to be knockout of OAS2 in in with reviewer suggestions (I'd also add IFNAR or equivalent for some experiments regarding this pathway).

2. The IFN dependent and independent mechanisms need better defined beyond RNAseL knockout (RNA degradation assays, IFN induction/signalling).

3. Viral experiment readouts are too variable and need unified, either by consistently using same assays (plaque or suchlike), or by demonstrating equivalence (or lack of, as I'm not sure they are equivalent really).

Thanks. We agree the different assays are not equivalent (and we apologise if this was implied, either indirectly or through lack of detail, in the previous version). We have repeated key experiments now with infectious virus, which while not altering our conclusions, has provided more compelling data.

4. RNA binding needs to be biochemically determined, not just computationally.

We have demonstrated RNA binding experimentally.

Major revisions all, but not impossible and they should be given the chance (in my opinion).

Thanks for your fair assessment and review, and for recommending we be given an opportunity to respond. We hope our new data are sufficient to allay the above concerns. We are certainly grateful as the experiments have pushed us and allowed us to greatly improve the manuscript and the quality of the data.

Dear Dr. Fletcher,

Thank you for submitting your manuscript for consideration by the EMBO Journal. It has now been re-evaluated by all three referees whose comments are shown below.

As you can see from the reports, referees #2 and #3 consider their concerns to be fully addressed. Referee #1, however, while appreciating the revisions, remains critical and does not feel that the manuscript demonstrates an important role for the OAS2 isoforms in antiviral defense. This referee also maintains that a substantial part of the proposed mechanistic conclusions is not adequately supported by the data presented.

We have re-discussed the manuscript within the editorial team and concluded that the persistent concerns raised by referee #1 are important and cannot be overruled. We have therefore decided that we are unable to offer publication in The EMBO Journal.

While we cannot pursue this manuscript further, we encourage you to transfer your study to our not-for-profit open-access sister journal, Life Science Alliance (LSA). We shared your manuscript and the accompanying reviews with LSA Executive Editor, Tim Fessenden, who is interested in these findings. He is pleased to offer publication of this manuscript at LSA pending the following revisions: - Adjust the abstract, results and discussion where needed to clearly convey that the observed mechanistic plasticity of OAS2 is determined by the cells' IFN signaling state (as is nicely laid out in lines 224-229 already), in view of comments on the overall contribution of OAS2 to innate immunity raised by Reviewer 1. - Clearly state in the results section that the assay shown in Fig 7L used a higher MOI vs elsewhere and discuss the modest degree of RNA degradation shown, as noted by Reviewer 1. Conversely the observation that OAS2 activates RNAse-L is already established in prior literature and could be removed from this figure. We encourage you to contact Dr. Fessenden at t.fessenden@life-science-alliance.org to discuss this work and the revisions requested. You may use the link below to immediately transfer your manuscript to LSA. You do not need to revise the manuscript before transferring it to LSA. Once you transfer, you will receive an invitation to revise and resubmit. Again please contact Dr. Fessenden if you have any questions about the LSA journal, the transfer process, or the revisions requested.

Thank you in any case for the opportunity to consider this manuscript. I am sorry we cannot be more positive on this occasion, but we hope nevertheless that you will find our referees' comments helpful.

Yours sincerely,

Cornelius Schneider, PhD
Editor
The EMBO Journal
c.schneider@embojournal.org

****

Referee #1:

The main conclusion of this manuscript is that the two isoforms of OAS2 have antiviral functions, however utilizing different mechanisms. OAS2 p69 is antiviral for OC43 by an RNase L independent pathway and OASp71 is antiviral for EMCV by an RNase L dependent pathway. The authors have done considerably more experiments to support their conclusions since the first version. While this is an intriguing observation, this reviewer feels like the manuscript still needs more data to support the conclusions. The conclusions, as in the initial submission, that OAS2 is antiviral for OC43 and for EMCV albeit by different mechanism still depend on either overexpression or IFN induced OAS expression. This is problematic. Thus, while the data suggest there may be some antiviral activity, it is a minor effect compared to other IFN induced activities and the authors need to subtract IFN induced antiviral activity from IFN plus OAS2 induced activity This should be acknowledged and discussed. The EMCV and OC43 experiments seem like two separate projects; more data on each project would help support the conclusions for each virus. For example, the demonstration of RNase L activation in EMCV infection needs further data to be conclusive.

OC43 experiments

Author comment: "OC43 growth is equivalent in the presence or absence of OAS2. How does this square with OAS2 being antiviral? In parallel Western blots (Fig. EV2G), we see no induction of OAS2 protein during viral replication (in parental control cells), likely explaining the absence of measurable difference between parental and KO cells in the absence of exogenous IFN. It is interesting that infection alone is insufficient to induce OAS2 expression......Plausibly, distinct ISGs that act earlier on viral

replication than OAS2 (for example, at viral entry) could mask the effects of OAS2 expression."

There is basal OAS2 expressed in A549 cells, at a low level and it is induced by IFN. OC43 like other CoVs has multiple antagonists of IFN induction so it is not surprising that infection does not induce OAS2 or IFN. The data in Figure 2 show that any antiviral effect of OAS2 is minor and seen only late in infections when the level is raised by IFN treatment. Both A549 cells and THP-1 show differences in replication +/- OAS2 only after IFN treatment and this difference is not significant in THP1-cells. THP-1s are myeloid cells and as such likely have more robust innate immune response which may dwarf the effect so OAS2 after IFN treatment.

Author comment: "We would like to note too that the level of OAS2 expression we achieve by overexpression is not substantially higher than that achieved by overnight IFN stimulation (Fig. 2C)."

This statement is confusing since, overexpressed OAS does look to be at a considerably higher level than IFN induced OAS in the western blot in Fig 2C.

EMCV
Author comment: "We are unable to reveal a role for endogenous OAS2 in EMCV restriction in A549, with or without IFN stimulation, however this is commensurate with the lack of OAS2 expression in A549 treated with IFN (Fig. 2C) and the induction of p69, but not p71, in A549 cells treated with IFN (Fig. 2C) (this sentence does not make sense)."

The conclusion regarding RNase L dependent restriction of EMCV by OAS2p71 based on the data presented is problematic. If p71 is not detectable by western blot following IFN treatment, then how can it be important for RNase L dependent restriction?

Fig 7L is the only demonstration of activation of RNase L during EMCV infection. P69 or p71 expression lead to a very small loss of RIN; at moi=2, EMCV infected cells may be destroyed at 24h. The RIN numbers show very little rRNA degradation even for treatment with 1ug polyIC (relatively high dose) used as positive for RNase L and should cause a much more dramatic reduction in RIN with many bands of degraded RNA obvious. pIC acts quickly and 24h hours may be late to observe RNase L activation and rRNA degradation. Most of the EMCV infection in the manuscript before this one were carried out at moi=0.005 pfu/cell while this one was at moi=2. This may be problematic for interpreting the data obtained at such different moi. Also p69 as well as p71 activate RNase L in this assay. For all these reasons activation of RNase L needs to be confirmed with another method. One method in the literature is to quantify PABC1 to the nucleus, a consequent of RNase L activation where activation can be observed on a single cell basis and be quantified (for example PMID: 39196777).

It would be much better to use MAVS or IFNAR KO cells than introducing another viral protein, BVDV NPro IFN antagonist to observe infection in the absence of IFN signaling. As the manuscript concludes and has been shown before in the literature, OAS/RNase L can be induced in the absence of IFN signaling in other viral infections of A549s, indicating that basal OAS's are sufficient to induce RNase L activation. This makes sense because once IFN in induced and other ISG activated, the cell becomes antiviral and OAS/RNase L is no longer a major antiviral factor.

It would be very interesting to know if OAS2 p69 is antiviral against SARS-CoV-2 or other CoVs or if P71 is antiviral against CoVs that do not encode PDEs. These authors have worked on OAS1 activation of RNase L during SARS-CoV-2 infection. Since OC43 and SARS-CoV-2 are CoVs of the same genus it might be expected that p69 would restrict SARS-CoV-2 also by a non-RNase L dependent pathway. A perfect experiment but clearly too much to ask is to repeat the p69/p71 experiments with an NS2 inactive mutant of OC43. Would p71 then activate RNase L and be antiviral against OC43.

Referee #2:

The authors have introduced a large number of experiments to directly address the core of the reviewers' comments. Namely these are further work with OC43 infection and infections in THP1 cells, alongside a number of more minor experiments or changes. They have systematically addressed all unclear points and provided caveats or clarifications. As such I think the work should be supported for publication.

Referee #3:

The authors have reasonably addressed most of the concerns and I support the publication of this manuscript.

** As a service to authors, EMBO Press provides authors with the possibility to transfer a manuscript that one journal cannot offer to publish to another EMBO publication or the open access journal Life Science Alliance launched in partnership between EMBO Press, Rockefeller University Press and Cold Spring Harbor Laboratory Press. The full manuscript and if applicable, reviewers' reports, are automatically sent to the receiving journal to allow for fast handling and a prompt decision on your manuscript. For more details of this service, and to transfer your manuscript please click on Link Not Available. **

Please do not share this URL as it will give anyone who clicks it access to your account.

Referee #1:

The main conclusion of this manuscript is that the two isoforms of OAS2 have antiviral functions, however utilizing different mechanisms. OAS2 p69 is antiviral for OC43 by an RNase L independent pathway and OASp71 is antiviral for EMCV by an RNase L dependent pathway. The authors have done considerably more experiments to support their conclusions since the first version. While this is an intriguing observation, this reviewer feels like the manuscript still needs more data to support the conclusions. The conclusions, as in the initial submission, that OAS2 is antiviral for OC43 and for EMCV albeit by different mechanism still depend on either overexpression or IFN induced OAS expression. This is problematic.

We respectfully question what specifically is problematic. As an ISG, these can only be studied by either IFN-priming cells or expressing. In the former case, we have both induced its expression with IFN, then depleted its expression via CRISPR, showing a reduction in the efficacy of IFN potency.

Fig. 2L, IFN inhibition of HCoV-OC43

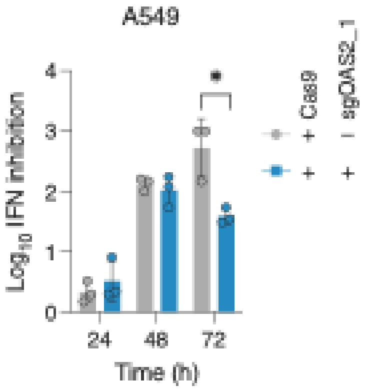

Thus, while the data suggest there may be some antiviral activity, it is a minor effect compared to other IFN induced activities and the authors need to subtract IFN induced antiviral activity from IFN plus OAS2 induced activity This should be acknowledged and discussed.

Indeed, it is entirely expected that the contribution of OAS2 to be just <u>one</u> component of IFN’s arsenal of antiviral activity. The fact that we see any difference between control and KO cells is a result we find astounding. In Fig. 1D, we perform a large genetic screen and identify many human ISGs that are capable of restricting HCoV-OC43 expression (the first of its kind). Thus, IFN could be working through any of these proteins, and therefore depletion of one is not expected to ablate IFN antiviral activity. For an excellent explanation of this, we refer the reviewer to John Schoggins recent review – PMID: 40333690.

The EMCV and OC43 experiments seem like two separate projects; more data on each project would help support the conclusions for each virus.

We have included significantly more data on both viruses – in fact more than was requested by the initial review. We have identified 2 amino acids in the C-terminal tail of the p69 isoform that entirely dictate restriction of HCoV-OC43. And we have clarified the requirement for RNase L in EMCV restriction by the p71 isoform (see below).

For example, the demonstration of RNase L activation in EMCV infection needs further data to be conclusive.

We provide compelling genetic evidence for a functional requirement (see below).

OC43 experiments

Author comment: "OC43 growth is equivalent in the presence or absence of OAS2. How does this square with OAS2 being antiviral? In parallel Western blots (Fig. EV2G), we see no induction of OAS2 protein during viral replication (in parental control cells), likely explaining the absence of measurable difference between parental and KO cells in the absence of exogenous IFN. It is interesting that infection alone is insufficient to induce OAS2 expression......Plausibly, distinct ISGs that act earlier on viral replication than OAS2 (for example, at viral entry) could mask the effects of OAS2 expression."

There is basal OAS2 expressed in A549 cells, at a low level and it is induced by IFN.

We respectfully disagree with this statement; in our cell lines, we fail to detect OAS2 in the absence of IFN stimulation. Which cell lines is the reviewer referring to?

OC43 like other CoVs has multiple antagonists of IFN induction so it is not surprising that infection does not induce OAS2 or IFN.

We completely agree. However, this was an experiment that was requested of us by this same reviewer:

A simple experiment to address this point would be to perform an OC43 growth curve in WT and OAS2 KO cells (at a defined MOI) and ask if OAS2 is induced during infection and if virus titer is increased in the OAS2 KO cells over time.

The data in Figure 2 show that any antiviral effect of OAS2 is minor and seen only late in infections when the level is raised by IFN treatment. Both A549 cells and THP-1 show differences in replication +/- OAS2 only after IFN treatment and this difference is not significant in THP1-cells.

We agree the effect in THP-1 cells just misses the p value threshold of 0.05, however as we state above, this is not entirely surprising, given the many antiviral proteins that IFN works through.

THP-1s are myeloid cells and as such likely have more robust innate immune response which may dwarf the effect so OAS2 after IFN treatment.

Quite. We don't see how this interpretation diminishes the fact that OAS2 is antiviral?

Author comment: "We would like to note too that the level of OAS2 expression we achieve by overexpression is not substantially higher than that achieved by overnight IFN stimulation (Fig. 2C)."

This statement is confusing since, overexpressed OAS does look to be at a considerably higher level than IFN induced OAS in the western blot in Fig 2C.

We agree. The example shown is 3-fold higher expression, which is not so drastically higher. The point we were trying to make by showing this data was that we had not expressed a protein at logs-fold higher expression levels. 3-fold seems relatively minor.

EMCV
Author comment: "We are unable to reveal a role for endogenous OAS2 in EMCV restriction in A549, with or without IFN stimulation, however this is commensurate with the lack of OAS2 expression in A549 treated with IFN (Fig. 2C) and the induction of p69, but not p71, in A549 cells treated with IFN (Fig. 2C) (this sentence does not make sense)."

Apologies for a typo – the sentence in our rebuttal meant to say:

Author comment: "We are unable to reveal a role for endogenous OAS2 in EMCV restriction in A549, with or without IFN stimulation, however this is commensurate with the lack of OAS2 expression in A549 not treated with IFN (Fig. 2C) and the induction of p69, but not p71, in A549 cells treated with IFN (Fig. 2C) (this sentence does not make sense)."

The point we were trying to make in the rebuttal was that a lack of effect of a KO in untreated (no IFN) A549 cells makes sense, as there is undetectable OAS2 expression in untreated (no IFN) cells.

The conclusion regarding RNase L dependent restriction of EMCV by OAS2p71 based on the data presented is problematic. If p71 is not detectable by western blot following IFN treatment, then how can it be important for RNase L dependent restriction?

Unfortunately, we think the reviewer has misunderstood our data. We have not suggested that p71 is required for RNase L-dependent restriction, but the other way around, that RNase L is required for p71 restriction of EMCV. In the absence of RNase L, p71 is non-functional (Fig. 7K).

Fig 7L is the only demonstration of activation of RNase L during EMCV infection. P69 or p71 expression lead to a very small loss of RIN; at moi=2, EMCV infected cells may be destroyed at 24h. The RIN numbers show very little rRNA degradation even for treatment with 1ug polyIC (relatively high dose) used as positive for RNase L and should cause a much more dramatic reduction in RIN with many bands of degraded RNA obvious. pIC acts quickly and 24h hours may be late to observe RNase L activation and rRNA degradation. Most of the EMCV infection in the manuscript

before this one were carried out at moi=0.005 pfu/cell while this one was at moi=2. This may be problematic for interpreting the data obtained at such different moi. Also p69 as well as p71 activate RNase L in this assay. For all these reasons activation of RNase L needs to be confirmed with another method. One method in the literature is to quantify PABC1 to the nucleus, a consequent of RNase L activation where activation can be observed on a single cell basis and be quantified (for example PMID: 39196777).

We thank the reviewer for this comment. We performed these RNA integrity experiments at the request of this same reviewer. Unfortunately, we respectfully disagree with their interpretation. While the effect is admittedly weak, the cells infected with EMCV in the presence of OAS2 p71, <u>show the lowest RIN score of all conditions tested</u>, supporting the model that p71 operates via RNase L.

The most compelling data to support our conclusion that p71, but no 69, operates via RNase L, are the genetic experiments here:

Fig. 7K (EMCV) and Fig. 5G (HCoV-OC43)

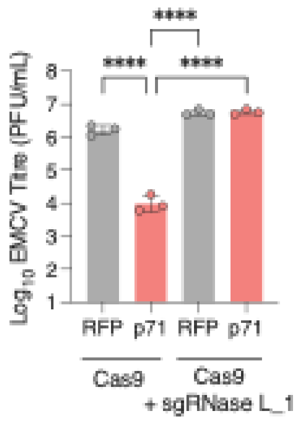

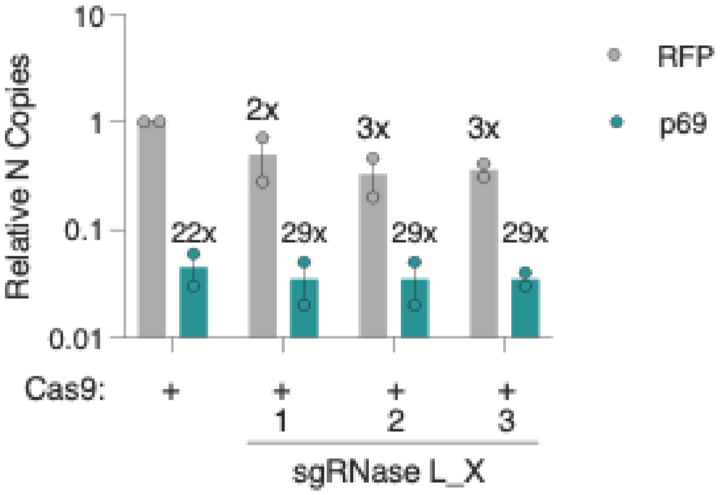

Regarding our use of an MOI for 2 hours, we were informed by the work of others in the field:

- In Li et al 2016 from the Weiss lab (PMID: 26858407) Fig. S1, the authors infected cells at an MOI of 10, for 24 hours.
- In Sorgeloos et al 2013, Fig. 2, the authors infected cells at an MOI of 20 for 9 hours (PMID: 23825954)
- In Han et al, Fig. 4 – the first RNase L structure – used the same RNA integratit assay to dissect mutations of RNase L, and see similarly modest effects on total RNA integrity, suggesting this is not an unprecedented phenomenon (PMID: 24578532).

In summary, we see the most degradation of cellular RNA in cells expressing p71 and infected with EMCV.

It would be much better to use MAVS or IFNAR KO cells than introducing another viral protein, BVDV NPro IFN antagonist to observe infection in the absence of IFN signaling.

We did not rely on one approach to assess the effect of IFN. The author has not acknowledged our additional data where we have used STAT1 KO (no effect on restriction) and used a sensitive IFN reporter, which failed to detect an induction of IFN.

As the manuscript concludes and has been shown before in the literature, OAS/RNase L can be induced in the absence of IFN signaling in other viral infections of A549s, indicating that basal OAS's are sufficient to induce RNase L activation. This makes sense because once IFN in induced and other ISG activated, the cell becomes antiviral and OAS/RNase L is no longer a major antiviral factor.

We agree. However, we would like to point out that RNase L is constitutively expressed in our cells – unlike OAS2. RNase L in our cells does not need IFN stimulation to become expressed.

It would be very interesting to know if OAS2 p69 is antiviral against SARS-CoV-2 or other CoVs or if P71 is antiviral against CoVs that do not encode PDEs.

Here we absolutely agree; our prediction would be that, should OAS2 be able to recognise SARS-CoV-2 RNA, then restriction would be via 2-5A synthesis and RNase L dependent.

These authors have worked on OAS1 activation of RNase L during SARS-CoV-2 infection. Since OC43 and SARS-CoV-2 are CoVs of the same genus it might be expected that p69 would restrict SARS-CoV-2 also by a non-RNase L dependent pathway.

SARS-CoV-2 and HCoV-OC43 belong to the same genius, but do not both encode for a PDE.

A perfect experiment but clearly too much to ask is to repeat the p69/p71 experiments with an NS2 inactive mutant of OC43. Would p71 then activate RNase L and be antiviral against OC43.

We're pleased the reviewer agrees that this NS2-deletion experiment that we propose in our discussion would be worthwhile. Indeed, this might even be the reason for a dual isoform pattern. We think that p69 represents a gain of function that allows for restriction of viruses that encode a PDE.

Dear Dr. Fletcher,

Thank you for submitting a revised version of your manuscript. The study has now been evaluated by an independent arbitrator, who finds that the remaining concerns raised by referee #1 have been satisfactorily addressed and recommends publication pending minor revisions.

We are therefore pleased to invite you to revise your manuscript, addressing the points raised by the arbitrating referee. In addition, there are a few mainly editorial points that will need to be addressed before I can proceed with formal acceptance of the manuscript:

- MANUSCRIPT FORMAT:(no figures!) .docx, no figures, no track changes

- AUTHORS: Authors' request: "I'd like to add Sam Wilson and Emma Davies (cc'd) as co-corresponding authors on our recently resubmitted manuscript. This was an error on my part during resubmission. Both authors have agreed with this decision." - the co-corresponding authors have already been marked in eJP, just need to be labeled in ms as well with their email addressed listed on the title page

- On the abstract page of the manuscript, please include 4-5 general keyword terms to enhance searchability.

- As we are switching from a free-text author contribution statement towards a more formal statement based on Contributor Role Taxonomy (CRediT) terms, please remove the present Author Contribution section and instead specify each author's contribution(s) directly in the Author Information page of our submission system during upload of the final manuscript. See https://casrai.org/credit/ for more information.

- APPENDIX 1 FILE WITH ToC: title page should contain "Appendix for + ms title" and ToC with the page numbers for the listed items; nomenclature should be Appendix Figure Sx and Appendix Table Sx throughout ms and Appendix PDF

- Please provide suggestions for a short 'blurb' text prefacing and summing up the conceptual aspect of the study in two sentences (max. 250 characters), followed by 3-5 one-sentence 'bullet points' with brief factual statements of key results of the paper; they will form the basis of an editor-written 'Synopsis' accompanying the online version of the article. Please also provide an altered synopsis image, making sure that the aspect ratio conforms to our website's format - it should be exactly 550 pixels wide and between 300-600 pixels high.

FIGURE CHECK IN PS: DONE, OK, CR - Wednesday, 12. November 2025.
Some of the blots have no background at all.
Cornelius, I would like to talk to you about how these blots were captured.

- Figure Legends (main + EV):
1. Please define the annotated p values ****/***/**/* as well as provide the exact p-values for the same in the legend of figure 2K as appropriate.

2. Please note that the exact p values are not provided in the legends of figures 2D, E; 3C, E; 5B, C; 7K, EV3 C, EV5 G.

3. Please indicate the statistical test used for data analysis in the legend of figure 2K

4. Please note that information related to n is missing in the legends of figures 2K, 3C, 8H, I, L, O; EV3 C

5. Please note that the error bars are not defined in the legends of figures 2K, EV3 C

- "Materials and Methods" should be renamed to "Methods"

- Sections need to be named and the order should be corrected: Title page - Abstract - Keywords - Introduction - Results - Discussion - Methods - Data Availability - Acknowledgements - Disclosure and Competing Interests Statement - References - Figure Legends - Table(s) - Expanded View Figure Legends.

Please let me know if you have any questions regarding any of these points. You can use the link below to upload the revised files.
Thank you again for giving us the chance to consider your manuscript for The EMBO Journal. I look forward to receiving the final version.
With best regards,
Cornelius Schneider

____
Cornelius Schneider, PhD
Editor | The EMBO Journal
c.schneider@embojournal.org

Please refer to our figure preparation guideline in order to ensure proper formatting and readability in print as well as on screen:

https://link.springer.com/journal/44318/submission-guidelines#cms-Figure-and-data-presentation

Use the link below to submit your revision:

https://emboj.msubmit.net/cgi-bin/main.plex

Referee #4:

I would like to thank the editor for the opportunity to review this manuscript. For context, as an additional reviewer, I was asked by the editor to "review the manuscript, the referee reports, and the authors' point-by-point response, and provide your opinion on the remaining concerns raised by the critical referee, as well as on the overall suitability of the manuscript for publication in The EMBO Journal".

I thoroughly enjoyed reading this manuscript and congratulate the authors on this fine piece of work. Using an overexpression screen of interferon stimulated genes, the authors make the remarkable observation that expression of OAS2 selectively inhibits the seasonal coronavirus HCoV-OC43. Like other OAS family members, OAS2 is alternatively spliced. Interestingly, the p69 isoform controls this coronavirus, whereas the p71 isoform does not. Mechanistically, N-terminal myristoylation and RNA binding are required for virus control, while catalytic activity and RNase L are not. The authors then go on to discover that another virus, EMCV, is sensitive to effects of the p71 isoform but not OAS2 p69. Here, in addition to N-terminal myristoylation and RNA binding, catalytic activity and RNaseL are required for the antiviral effects. The manuscript concludes with intriguing results on the effects of the OAS2 C-terminus on control of these two viruses. Overall, these conclusions are very well supported by the experimental data. The manuscript falls short off describing the precise mechanisms of how HCoV-OC43 is controlled by the p69 isoform, the molecular role of the C-terminus, and why the related SARS-CoV-2 is largely insensitive to OAS2-p69. However, the manuscript already contains a large body of high-quality data and these questions should in my view be addressed in future work.

I have also considered the criticisms raised by reviewer #1 on the revised manuscript, which I understand have led to rejection of the manuscript. Specifically:

• This reviewer is concerned that OAS2 is either overexpressed or induced by recombinant interferon added to cells. I agree that an experiment in which interferon, and consequently OAS2, is naturally induced by the virus, would strengthen the conclusions. I noted that infection of A549 cells with both viruses resulted in identical virus replication in wild type and OAS2 knockout cells (Figs 2K and 7C). In contrast, both viruses replicated better in knockout THP1 cells (Figs 2M and 7E). A key difference between these two cell types is that THP1 cells express MDA5 (the interferon-inducing sensor for both viruses) at baseline whereas A549 cells do not. It is therefore likely that THP1 cells mount an endogenous interferon response that induces OAS2 in wild type cells, which in turn controls the viruses. This could be tested experimentally by western blotting or RT-qPCR; however, in my view adding this consideration in text should be sufficient.
• Reviewer #1 further raises concerns that the effect in figure 2L is minor. Here, I fully agree with the response of the authors.
• I disagree with reviewer #1 that the experiments on the two different viruses "feel like 2 separate projects".
• I agree with the reviewer that the effects in figure 7L are rather small, but I also agree with the authors that these effects are still significant. I suggest that the authors show these data by plotting trace lines in addition to the gel like images shown in figure 7L. For example, the traces from the three conditions involving EMCV could be overlaid. I believe this will nicely show the effect when p71 is overexpressed.
• Where the authors agree with the reviewer but do not have additional data, I would suggest incorporating such points into the discussion and or a "limitations of the study" paragraph.

I have one specific point:
Fig 4F,G - These results may be due to lower expression of mutant p69 protein (4E), which should be acknowledged in text.

In conclusion, in my view this manuscript is very suitable for publication in The EMBO Journal with minor modifications to the text.

I thoroughly enjoyed reading this manuscript and congratulate the authors on this fine piece of work. Using an overexpression screen of interferon stimulated genes, the authors make the remarkable observation that expression of OAS2 selectively inhibits the seasonal coronavirus HCoV-OC43. Like other OAS family members, OAS2 is alternatively spliced. Interestingly, the p69 isoform controls this coronavirus, whereas the p71 isoform does not. Mechanistically, N-terminal myristoylation and RNA binding are required for virus control, while catalytic activity and RNase L are not. The authors then go on to discover that another virus, EMCV, is sensitive to effects of the p71 isoform but not OAS2 p69. Here, in addition to N-terminal myristoylation and RNA binding, catalytic activity and RNaseL are required for the antiviral effects. The manuscript concludes with intriguing results on the effects of the OAS2 C-terminus on control of these two viruses. Overall, these conclusions are very well supported by the experimental data. The manuscript falls short off describing the precise mechanisms of how HCoV-OC43 is controlled by the p69 isoform, the molecular role of the C-terminus, and why the related SARS-CoV-2 is largely insensitive to OAS2-p69. However, the manuscript already contains a large body of high-quality data and these questions should in my view be addressed in future work.

We thank this reviewer very much for taking the time to arbitrate, and for their positive and supportive comments.

I have also considered the criticisms raised by reviewer #1 on the revised manuscript, which I understand have led to rejection of the manuscript. Specifically:
• This reviewer is concerned that OAS2 is either overexpressed or induced by recombinant interferon added to cells. I agree that an experiment in which interferon, and consequently OAS2, is naturally induced by the virus, would strengthen the conclusions. I noted that infection of A549 cells with both viruses resulted in identical virus replication in wild type and OAS2 knockout cells (Figs 2K and 7C). In contrast, both viruses replicated better in knockout THP1 cells (Figs 2M and 7E). A key difference between these two cell types is that THP1 cells express MDA5 (the interferon-inducing sensor for both viruses) at baseline whereas A549 cells do not. It is therefore likely that THP1 cells mount an endogenous interferon response that induces OAS2 in wild type cells, which in turn controls the viruses. This could be tested experimentally by western blotting or RT-qPCR; however, in my view adding this consideration in text should be sufficient.

We agree and have added the following sentences at line 219 relating to Fig. 2:

A key difference between these two cell types is that THP-1 cells likely express higher levels of IFIH1/MDA5 (an interferon-inducing RIG-I-like receptor) at baseline compared to A549 (Lama et al, 2026). It is therefore possible that THP-1 cells mount an endogenous interferon response that induces OAS2 in wild type cells, which in turn controls the virus. However, the lack of a complete rescue to viral replication in OAS2 depleted cells is

And the following sentences at line 399 relating to Fig. 7:

As observed with HCoV-OC43 replication in THP-1 cells (Fig. 2M), EMCV replication in THP-1 cells was modestly rescued by OAS2 depletion in the absence of exogenous IFN (Fig. 7E),

again likely reflecting a higher baseline expression of RLRs like MDA5 in these cells compared to A549 cells (Lama et al., 2026).

We have inserted a new reference (Lama et al., 2026, PMID: 41680413), which compared baseline MDA5 expression between THP-1 and A549 transcriptomic data sets and made the same observation suggested by the reviewer. We hope this is an appropriate reference to include but would change this for another if preferred by the reviewer.

• Reviewer #1 further raises concerns that the effect in figure 2L is minor. Here, I fully agree with the response of the authors.

Thank you for the comment.

• I disagree with reviewer #1 that the experiments on the two different viruses "feel like 2 separate projects".

Thank you for the comment.

• I agree with the reviewer that the effects in figure 7L are rather small, but I also agree with the authors that these effects are still significant. I suggest that the authors show these data by plotting trace lines in addition to the gel like images shown in figure 7L. For example, the traces from the three conditions involving EMCV could be overlaid. I believe this will nicely show the effect when p71 is overexpressed.

Thanks for this helpful suggestion. We have amended Fig. 7 to include a new panel M:

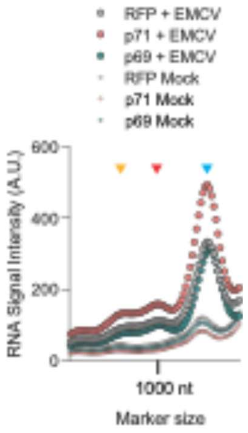

The Figure 7 legend has been amended to accommodate this change, and the legend text updated to reflect the new data presentation.

• Where the authors agree with the reviewer but do not have additional data, I would suggest incorporating such points into the discussion and or a "limitations of the study" paragraph.
Thanks for the suggestion. We feel we'd already incorporated in our discussion, the points that the reviewer 1 mentioned. Specifically, reviewer 1 said:

A perfect experiment but clearly too much to ask is to repeat the p69/p71 experiments with an NS2 inactive mutant of OC43. Would p71 then activate RNase L and be antiviral against OC43.

And we had speculated on this in the discussion:

However, understanding whether restriction versus sensing are strictly split (can p69 restrict a virus via 2-5A synthesis, and p71 via RNase L-independent mechanisms?) will provide a clearer picture of how malleable the OAS2 locus is in humans. Testing the replication of an NS2-deleted HCoV-OC43 (Diefenbacher et al, 2024) in p69- and p71-expressing cells could address whether any residual RNase L-dependent mechanism is indeed antagonised by this viral PDE.

I have one specific point:
Fig 4F,G - These results may be due to lower expression of mutant p69 protein (4E), which should be acknowledged in text.

Thanks for pointing this out. We've added the following qualifier in the text at line 321:

While the lower expression level of the RNA binding mutant might also contribute to the absence of measurable restriction

In conclusion, in my view this manuscript is very suitable for publication in The EMBO Journal with minor modifications to the text.

Thanks for all of your comments.

We have also added one missing reference in the discussion (Fletcher et al., PNAS 2015) related to the topic of restriction/sensing duality on line 503.

2nd Revision - Editorial Decision **11th May 2026**

Dear Dr. Fletcher,

I am pleased to inform you that your manuscript has been accepted for publication in the EMBO Journal.

Your manuscript will be processed for publication by EMBO Press. It will be copy edited and you will receive page proofs prior to publication. Please note that you will be contacted by Springer Nature Author Services to complete licensing and payment information.

You may qualify for financial assistance for your publication charges - either via a Springer Nature fully open access agreement or an EMBO initiative. Check your eligibility: https://link.springer.com/journal/44318/how-to-publish-with-us

Should you be planning a Press Release on your article, please get in contact with embo_production@springernature.com as early as possible in order to coordinate publication and release dates.

If you have any questions, please do not hesitate to contact the Editorial Office. Thank you for your contribution to The EMBO Journal.

Yours sincerely,

Cornelius Schneider, PhD
Editor
The EMBO Journal
c.schneider@embojournal.org

*****
Please note that it is The EMBO Journal policy for the transcript of the editorial process (containing referee reports and your response letters) to be published as an online supplement to each paper. If you should prefer removal of any referee-only figures included in the point-by-point response(s), e.g. because they may still be used for future publication or because they have been reproduced from published work by others, please do let us know immediately via response email.
More information is available here: https://link.springer.com/partners/embo-press/editorial-policies#Peer%20review

** Click here to be directed to your login page: https://emboj.msubmit.net

EMBO Press Author Checklist

Corresponding Author Name: Adam J. Fletcher	
Journal Submitted to: The EMBO Journal	
Manuscript Number: EMBOJ-2025-120553	

USEFUL LINKS FOR COMPLETING THIS FORM
The EMBO Journal - Author Guidelines
EMBO Reports - Author Guidelines
Molecular Systems Biology - Author Guidelines
EMBO Molecular Medicine - Author Guidelines

Reporting Checklist for Life Science Articles (updated January
This checklist is adapted from Materials Design Analysis Reporting (MDAR) Checklist for Authors. MDAR establishes a minimum set of requirements in transparent reporting in the life sciences (see Statement of Task: 10.31222/osf.io/9sm4x). Please follow the journal's guidelines in preparing your
Please note that a copy of this checklist will be published alongside your article.

Abridged guidelines for figures
1. Data
The data shown in figures should satisfy the following conditions:
- the data were obtained and processed according to the field's best practice and are presented to reflect the results of the experiments in an accurate and unbiased manner.
- ideally, figure panels should include only measurements that are directly comparable to each other and obtained with the same assay.
- plots include clearly labeled error bars for independent experiments and sample sizes. Unless justified, error bars should not be shown for technical
- if n<5, the individual data points from each experiment should be plotted. Any statistical test employed should be justified.
- Source Data should be included to report the data underlying figures according to the guidelines set out in the authorship guidelines on Data

2. Captions
Each figure caption should contain the following information, for each panel where they are relevant:
- a specification of the experimental system investigated (eg cell line, species name).
- the assay(s) and method(s) used to carry out the reported observations and measurements.
- an explicit mention of the biological and chemical entity(ies) that are being measured.
- an explicit mention of the biological and chemical entity(ies) that are altered/varied/perturbed in a controlled manner.
- the exact sample size (n) for each experimental group/condition, given as a number, not a range;
- a description of the sample collection allowing the reader to understand whether the samples represent technical or biological replicates (including how many animals, litters, cultures, etc.).
- a statement of how many times the experiment shown was independently replicated in the laboratory.
- definitions of statistical methods and measures:
  - common tests, such as t-test (please specify whether paired vs. unpaired), simple χ2 tests, Wilcoxon and Mann-Whitney tests, can be unambiguously identified by name only, but more complex techniques should be described in the methods section;
  - are tests one-sided or two-sided?
  - are there adjustments for multiple comparisons?
  - exact statistical test results, e.g., P values = x but not P values < x;
  - definition of 'center values' as median or average;
  - definition of error bars as s.d. or s.e.m.

Please complete ALL of the questions below.
Select "Not Applicable" only when the requested information is not relevant for your study.

Materials

Newly Created Materials	Information included in the manuscript?	In which section is the information available? (Reagents and Tools Table, Materials and Methods, Figures, Data Availability Section)
New materials and reagents need to be available; do any restrictions apply?	Yes	Materials and Methods

Antibodies	Information included in the manuscript?	In which section is the information available? (Reagents and Tools Table, Materials and Methods, Figures, Data Availability Section)
For **antibodies** provide the following information:<br>- Commercial antibodies: RRID (if possible) or supplier name, catalogue number and or/clone number<br>- Non-commercial: RRID or citation	Yes	Reagents and Tools Table

DNA and RNA sequences	Information included in the manuscript?	In which section is the information available? (Reagents and Tools Table, Materials and Methods, Figures, Data Availability Section)
**Short novel DNA or RNA including primers, probes**: provide the sequences.	Yes	Appendix Table S1

Cell materials	Information included in the manuscript?	In which section is the information available? (Reagents and Tools Table, Materials and Methods, Figures, Data Availability Section)
**Cell lines:** Provide species information, strain. Provide accession number in repository **OR** supplier name, catalog number, clone number, and/**OR** RRID.	Yes	Reagents and Tools Table
**Primary cultures:** Provide species, strain, sex of origin, genetic modification status.	Not Applicable	
Report if the cell lines were recently **authenticated** (e.g., by STR profiling) and tested for mycoplasma contamination.	Yes	

Experimental animals	Information included in the manuscript?	In which section is the information available? (Reagents and Tools Table, Materials and Methods, Figures, Data Availability Section)
**Laboratory animals or Model organisms:** Provide species, strain, sex, age, genetic modification status. Provide accession number in repository **OR** supplier name, catalog number, clone number, **OR** RRID.	Not Applicable	
**Animal observed in or captured from the field:** Provide species, sex, and age where possible.	Not Applicable	
Please detail **housing and husbandry conditions**.	Not Applicable	

Plants and microbes	Information included in the manuscript?	In which section is the information available? (Reagents and Tools Table, Materials and Methods, Figures, Data Availability Section)
**Plants:** provide species and strain, ecotype and cultivar where relevant, unique accession number if available, and source (including location for collected wild specimens).	Not Applicable	
**Microbes:** provide species and strain, unique accession number if available, and source.	Yes	Reagents and Tools Table

Human research participants	Information included in the manuscript?	In which section is the information available? (Reagents and Tools Table, Materials and Methods, Figures, Data Availability Section)
If collected and within the bounds of privacy constraints report on age, sex and gender or ethnicity for all study participants.	Not Applicable	

Core facilities	Information included in the manuscript?	In which section is the information available? (Reagents and Tools Table, Materials and Methods, Figures, Data Availability Section)
If your work benefited from core facilities, was their service mentioned in the acknowledgments section?	Yes	Acknowledgements

Design

Study protocol	Information included in the manuscript?	In which section is the information available? (Reagents and Tools Table, Materials and Methods, Figures, Data Availability Section)
If study protocol has been **pre-registered, provide DOI in the manuscript**. For clinical trials, provide the trial registration number **OR** cite DOI.	Not Applicable	
Report the **clinical trial registration number** (at ClinicalTrials.gov or equivalent), where applicable.	Not Applicable	

Laboratory protocol	Information included in the manuscript?	In which section is the information available? (Reagents and Tools Table, Materials and Methods, Figures, Data Availability Section)
Provide DOI OR other citation details if **external detailed step-by-step protocols** are available.	Yes	Materials and Methods

Experimental study design and statistics	Information included in the manuscript?	In which section is the information available? (Reagents and Tools Table, Materials and Methods, Figures, Data Availability Section)
Include a statement about **sample size** estimate even if no statistical methods were used.	Yes	Figures
Were any steps taken to minimize the effects of subjective bias when allocating animals/samples to treatment (e.g. **randomization procedure**)? If yes, have they been described?	Not Applicable	
Include a statement about **blinding** even if no blinding was done.	Yes	Cell culture experiments were not blinded; samples were analysed with the cell genotype or experimental condition known.
Describe **inclusion/exclusion criteria** if samples or animals were excluded from the analysis. Were the criteria pre-established?<br><br>If sample or data points were omitted from analysis, report if this was due to attrition or intentional exclusion and provide justification.	Yes	Data points were not excluded during analysis
For every figure, are **statistical tests** justified as appropriate? Do the data meet the assumptions of the tests (e.g., normal distribution)? Describe any methods used to assess it. Is there an estimate of variation within each group of data? Is the variance similar between the groups that are being statistically compared?	Yes	

Sample definition and in-laboratory replication	Information included in the manuscript?	In which section is the information available? (Reagents and Tools Table, Materials and Methods, Figures, Data Availability Section)
In the figure legends: state number of times the experiment was **replicated** in laboratory.	Yes	Figures
In the figure legends: define whether data describe **technical or biological replicates**.	Yes	Figures

Ethics

Ethics	Information included in the manuscript?	In which section is the information available? (Reagents and Tools Table, Materials and Methods, Figures, Data Availability Section)
Studies involving **human participants**: State details of **authority granting ethics approval** (IRB or equivalent committee(s), provide reference number for approval.	Not Applicable	
Studies involving **human participants**: Include a statement confirming that **informed consent** was obtained from all subjects and that the experiments conformed to the principles set out in the WMA Declaration of Helsinki and the Department of Health and Human Services Belmont Report.	Not Applicable	
Studies involving **human participants:** For publication of **patient photos**, include a statement confirming that consent to publish was obtained.	Not Applicable	
Studies involving experimental **animals**: State details of **authority granting ethics approval** (IRB or equivalent committee(s), provide reference number for approval. Include a statement of compliance with ethical regulations.	Not Applicable	
Studies involving **specimen and field samples:** State if relevant **permits** obtained, provide details of authority approving study; if none were required, explain why.	Not Applicable	

Dual Use Research of Concern (DURC)	Information included in the manuscript?	In which section is the information available? (Reagents and Tools Table, Materials and Methods, Figures, Data Availability Section)
Could your study fall under dual use research restrictions? Please check biosecurity documents and list of **select agents and toxins** (CDC): https://www.selectagents.gov/sat/list.htm	Not Applicable	
If you used a select agent, is the security level of the lab appropriate and reported in the manuscript?	Not Applicable	
If a study is subject to dual use research of concern regulations, is the name of the **authority granting approval and reference number** for the regulatory approval provided in the manuscript?	Not Applicable	

Reporting

The MDAR framework recommends adoption of discipline-specific guidelines, established and endorsed through community initiatives. Journals have their own policy about requiring specific guidelines and recommendations to complement MDAR.

Adherence to community standards	Information included in the manuscript?	In which section is the information available? (Reagents and Tools Table, Materials and Methods, Figures, Data Availability Section)
State if relevant guidelines or checklists (e.g., **ICMJE, MIBBI, ARRIVE, PRISMA**) have been followed or provided.	Not Applicable	
For **tumor marker prognostic studies,** we recommend that you follow the **REMARK** reporting guidelines (see link list at top right). See author guidelines, under 'Reporting Guidelines'. Please confirm you have followed these guidelines.	Not Applicable	
For **phase II and III randomized controlled trials**, please refer to the **CONSORT** flow diagram (see link list at top right) and submit the CONSORT checklist (see link list at top right) with your submission. See author guidelines, under 'Reporting Guidelines'. Please confirm you have submitted this list.	Not Applicable	

Data Availability

Data availability	Information included in the manuscript?	In which section is the information available? (Reagents and Tools Table, Materials and Methods, Figures, Data Availability Section)
Have **primary datasets** been deposited according to the journal's guidelines (see 'Data Deposition' section) and the respective accession numbers provided in the Data Availability Section?	Not Applicable	
Were **human clinical and genomic datasets** deposited in a public access-controlled repository in accordance to ethical obligations to the patients and to the applicable consent agreement?	Not Applicable	
Are **computational models** that are central and integral to a study available without restrictions in a machine-readable form? Were the relevant accession numbers or links provided?	Not Applicable	
If publicly available data were reused, provide the respective **data citations in the reference list**.	Not Applicable	
